# Chemoenzymatic Synthesis of Complex Phenylpropanoid Derivatives by the *Botrytis cinerea* Secretome and Evaluation of Their Wnt Inhibition Activity

**DOI:** 10.3389/fpls.2021.805610

**Published:** 2022-01-13

**Authors:** Robin Huber, Laurence Marcourt, Alexey Koval, Sylvain Schnee, Davide Righi, Emilie Michellod, Vladimir L. Katanaev, Jean-Luc Wolfender, Katia Gindro, Emerson Ferreira Queiroz

**Affiliations:** ^1^School of Pharmaceutical Sciences, Centre Médical Universitaire (CMU), University of Geneva, Geneva, Switzerland; ^2^Institute of Pharmaceutical Sciences of Western Switzerland, Centre Médical Universitaire (CMU), University of Geneva, Geneva, Switzerland; ^3^Department of Cell Physiology and Metabolism, Translational Research Centre in Oncohaematology, Faculty of Medicine, Centre Médical Universitaire (CMU), University of Geneva, Geneva, Switzerland; ^4^Mycology Group, Research Department Plant Protection, Agroscope, Nyon, Switzerland; ^5^School of Biomedicine, Far Eastern Federal University, Vladivostok, Russia

**Keywords:** chemoenzymatic synthesis, biotransformation, *Botrytis cinerea*, enzymatic secretome, phenylpropanoids, high-resolution semi-preparative HPLC, dry load introduction, Wnt inhibition

## Abstract

In this study, a series of complex phenylpropanoid derivatives were obtained by chemoenzymatic biotransformation of ferulic acid, caffeic acid, and a mixture of both acids using the enzymatic secretome of *Botrytis cinerea.* These substrates were incubated with fungal enzymes, and the reactions were monitored using state-of-the-art analytical methods. Under such conditions, a series of dimers, trimers, and tetramers were generated. The reactions were optimized and scaled up. The resulting mixtures were purified by high-resolution semi-preparative HPLC combined with dry load introduction. This approach generated a series of 23 phenylpropanoid derivatives, 11 of which are described here for the first time. These compounds are divided into 12 dimers, 9 trimers (including a completely new structural scaffold), and 2 tetramers. Elucidation of their structures was performed with classical spectroscopic methods such as NMR and HRESIMS analyses. The resulting compound series were analyzed for anti-Wnt activity in TNBC cells, with several derivatives demonstrating specific inhibition.

## Introduction

Throughout history, natural products (NPs) have been a source of inspiration for the development of a large number of therapeutics (Newman and Cragg, 2020). New chemical entities based on NP “scaffolds” continue to be used as the basis for the development of a large number of approved drugs and drug candidates to treat many diseases (Newman and Cragg, 2020). The reason for this success in drug discovery can probably be explained by the great chemical diversity of NPs and the fact that these compounds are the result of natural selection where living organisms produce them to interact with biological targets (Harvey et al., 2015). A series of studies revealed that NPs have a greater number of chiral centers and increased steric complexity than synthetic drugs (Rodrigues et al., 2016). Because of their broad chemical diversity, NP collections are known to better represent the “chemical space” of drug-like molecules than synthetic compounds (Larsson et al., 2007).

The classical way of obtaining bioactive NPs is based on the bioassay-guided fractionation of an active extract from plant or microbial origin (Bucar et al., 2013). This strategy is based on the separation of constituents using different chromatographic steps combined with biological assays, aiming at the isolation and identification of the compound(s) responsible for a given biological activity (Bucar et al., 2013). While being very successful for the discovery of important drugs, this approach is challenging because of low yields of isolated compounds, limited supply of natural sources that produce them, and difficulty of total synthesis or targeted structural modification due to structural complexity (Li and Vederas, 2009). The constitution of pure NP chemical libraries for biological screening using this approach is a time-consuming and expensive process. In this regard, efforts have been made to screen natural fraction libraries more compatible with high-content screening platforms (Harvey et al., 2015; Thornburg et al., 2018).

In this context, chemoenzymatic methods using common NPs as substrates could be an interesting option to generate chemically diverse and well-characterized NP derivative libraries. Biotransformation reactions represent an interesting tool for chemo-diversification of NPs covering a large chemical space from sustainable sources (Li and Lou, 2018). Rather than using an intact microorganism or a pure enzyme to perform biotransformation, we have developed an original concept to biotransform NPs using the secretome of given fungi (mixture of their enzymes) to obtain chemically diverse NP derivatives using enzymatic reactions. The use of secretome takes advantage of the catalytic promiscuity of different enzymes (Copley, 2003). This approach was successfully used for the generation of specific libraries of complex stilbene dimers with chiral carbons (Gindro et al., 2017; Righi et al., 2020). Some of these compounds presented remarkable antifungal and antibacterial properties (Gindro et al., 2017; Righi et al., 2020). After having successfully applied this biotransformation process on stilbenes, we decided to use this methodology to another family of molecules, and phenylpropanoids were selected.

Phenylpropanoids are indeed ubiquitous secondary metabolites in the plant kingdom. The core structure of these compounds consists of a phenyl group attached to a small chain of three carbons. This carbon skeleton is obtained from the amino acid phenylalanine by enzymatic deamination (Vanholme et al., 2019). Phenylpropanoids are the main component of lignin, the second most abundant polymer in vascular plants (Boerjan et al., 2003). Lignin is synthesized from the combinatorial oxidative coupling of *p*-hydroxycinnamyl alcohol monomers and related compounds (Boerjan et al., 2003). Phenylpropanoids also play an important role in plant responses toward biotic and abiotic stimuli (Vogt, 2010; Arbona et al., 2013). They are also relevant for human health because of their antioxidant (Sharma et al., 2016), antiviral (Liu et al., 2015), and anticancer properties (Gindro et al., 2012; Hasan et al., 2017; Hematpoor et al., 2018). Phenylpropanoid derivatives such as caffeic and ferulic acids represent important substrates considered as relevant building blocks in drug discovery (Touaibia et al., 2011; Kumar and Pruthi, 2014; Davison and Brimble, 2019). Previous studies have demonstrated the successful use of these compounds as the starting material for the generation of more complex compounds through a biotransformation process using a purified enzyme (Wan et al., 2008; Saliu et al., 2011; Constantin et al., 2012). The resulting novel compounds could have therapeutic potential for major diseases.

Breast cancer (BC) is responsible for 627,000 deaths worldwide in 2018, being the most widespread cancer type in women. In triple-negative breast cancer (TNBC), expression of the estrogen and progesterone receptors, as well as human epidermal growth factor receptor 2 (HER2) is missing, making it unsusceptible to the targeted treatment and thus resulting in a drastically poor prognosis and a strong risk of relapse in the first 5 years following surgery (Lee et al., 2019).

The Wnt signaling pathway is reported in many sources to be a target for TNBC (Shaw et al., 2019b). The signaling cascade is initiated by Wnt ligands through the Frizzled receptor, which culminates in the deactivation of a destruction complex consisting of proteins axin, APC, and GSK3β, which together phosphorylate and thus reduce cytoplasmic β-catenin by promoting its proteasomal degradation. In the absence of a destruction complex activity, β-catenin rapidly accumulates and translocates in the nucleus where it exerts its activity as a transcriptional co-factor for a variety of pro-proliferative genes. This so-called “canonical” branch of Wnt signaling was the first discovered and plays the most significant role in TNBC and many other cancers. Despite its long story, the pathway remains undrugged to the present day and the solution to the quest of finding specific and selective inhibitors for the pathway activity promises to save millions of patients’ lives worldwide.

In this study, the aim was to perform biotransformations of these representative phenylpropanoid units using the *B. cinerea* secretome to obtain a series of more complex structures for evaluation of their Wnt inhibition properties.

## Results and Discussion

### Generation of Phenylpropanoid Derivatives Using the Enzymatic Secretome of *Botrytis cinerea*

**FIGURE 1:**
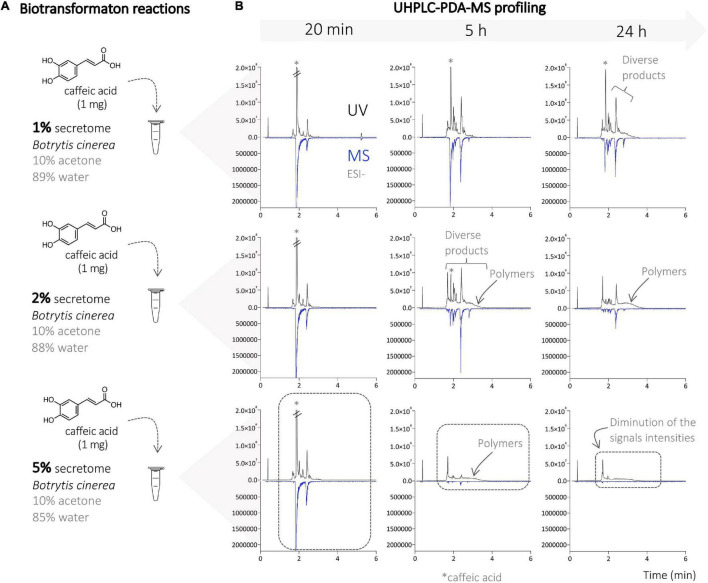
**(A)** Biotransformation reactions of caffeic acid with different amounts of the enzymatic secretome of *Botrytis cinerea* (1, 2, and 5%). **(B)** UHPLC-PDA-MS profile of the different reactions on the analytical scale at different times (20 min, and 5 and 24 h). A strong decrease in signal intensity is observed with 5% secretome (at 5 and 24 h) due to the strong polymerization process leading to the generation of insoluble compounds (polymers). The chromatograms obtained at 10% corresponded to the ones obtained at 5% and are, therefore, not shown.

**FIGURE 2:**
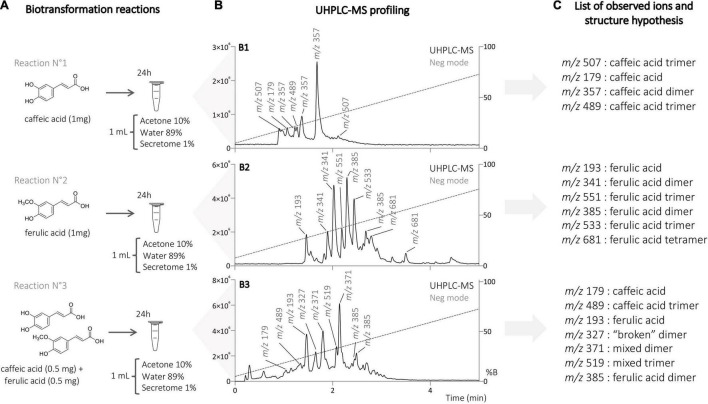
**(A)** Biotransformation reactions on caffeic acid, ferulic acid, and mixture of both acids with the enzymatic secretome of *B. cinerea*. **(B)** UHPLC-MS profiling of the different reactions on the analytical scale with the top-scan *m/z* for the main peaks. **(C)** List of the observed ions and subsequent hypotheses about their chemical identity.

First, a series of biotransformation reactions were performed on an analytical scale, using caffeic acid (1 mg), ferulic acid (1 mg), and a mixture of both (0.5 mg of each) acids as substrates. The compounds were first solubilized in acetone, then diluted in water, and the *B. cinerea* secretome was added last. The amount of substrate, acetone, and water remained constant, but that of the secretome varied at 1, 5, and 10% (Figure 1). The substrates were incubated for 24 h, and samples were collected after 20 min, 5 h, and 24 h. The reaction mixtures were submitted to metabolite profiling by UHPLC-PDA-ELSD-MS. The electrospray ionization mass spectrometry (ESI-MS) detection showed a series of molecular ions, suggesting the presence of dimer (*m/z* 300–400), trimer (*m/z* 450–600), and tetramer (>*m/z* 680) derivatives (Figure 2). UV trace profiling of these reactions showed under certain conditions (higher times or higher secretome amounts) the presence of a broad unresolved peak resulting in baseline deformation (“unsolved hump”), suggesting the presence of polymers (Figure 1) (Queiroz et al., 2014). Generation of polymers during the incubation of phenylpropanoids with laccases had already been previously reported (Adelakun et al., 2012). The almost complete absence of MS signals at 5 h and 24 h using 5% enzymatic secretome also supports this fact, with the upper detection limit of the instrument being *m/z* 1,250. These reactions also presented an insoluble brown precipitate attributed to the presence of polymers. Based on this information, the reaction conditions (time and amount of secretome) were, therefore, carefully adjusted to optimize the consumption of starting materials while avoiding polymer generation (Figure 2).

The metabolite profiles obtained under optimal conditions revealed the presence of a large number of generated compounds (Figure 2). The reactions were, therefore, scaled up to isolate and identify them. The three biotransformation reactions with caffeic acid, ferulic acid, and a mixture of both were performed on a large scale with 100 mg of total substrate amount and using the optimized conditions mentioned above (see “Experimental” section).

**FIGURE 3:**
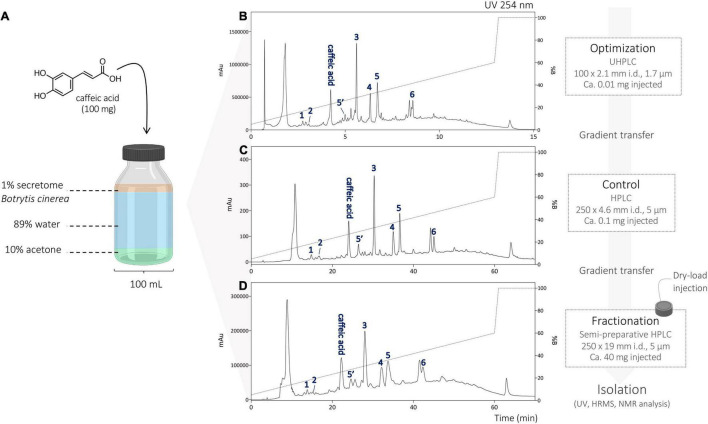
**(A)** Scaled up biotransformation reaction of caffeic acid with the enzymatic secretome of *B. cinerea* and 10% of acetone. **(B)** UHPLC-PDA analysis with optimized chromatographic conditions. **(C)** HPLC-PDA analysis using the transferred optimized chromatographic conditions. **(D)** Semi-preparative HPLC-UV analysis using the transferred optimized chromatographic conditions, and isolation of the generated compounds.

After monitoring the crude reaction mixtures, the separation conditions were optimized on the UHPLC scale. The optimized chromatographic conditions were transferred to the analytical (as a control) and semi-preparative HPLC scale by geometric gradient transfer (Guillarme et al., 2008). The stationary-phase chemistry of the columns used on each instrument was the same to maintain the same chromatographic selectivity (Figure 3). The shift in retention time between the UHPLC and HPLC scale can be explained by the difference in temperature of these analyses; UHPLC was performed at 40°C to decrease solvent viscosity and pressure, while HPLC analysis was performed at room temperature (25°C). Semi-preparative injections were performed using a dry load method, allowing to preserve a better resolution compared to a classical loop injection, according to a protocol recently developed in our laboratory (Queiroz et al., 2019). The geometrical transfer of the chromatographic conditions allowed for the preservation of good separation of the compound and thus isolation of the majority of the compounds in a single step. Purification of these reaction mixtures yielded a total of 24 compounds; compounds 1–6 from the reaction with caffeic acid, 7–15 from the reaction with ferulic acid, and 16–24 from the mixed reaction (Figure 3 and Supplementary Figures 1, 2).

### Structure Elucidation of the Generated Compounds

**FIGURE 4:**
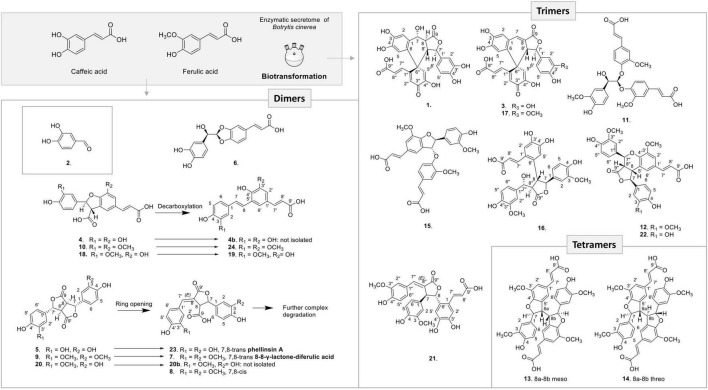
Compounds generated by the three biotransformation reactions with caffeic acid, ferulic acid, and mixture of both acids with the enzymatic secretome of *B. cinerea.*

From the 3 biotransformation reactions, 24 compounds were isolated and fully characterized by HRMS and NMR including 12 dimer, 9 trimer, and 2 tetramer derivatives (Figure 4). In addition, a degraded aromatic compound was identified as 3,4-dihydroxybenzaldehyde (2) (Kang et al., 2004). Each class of compounds is described in the next paragraphs.

Among the 12 dimers produced, 9 were already known and 3 are new derivatives. Compound 6 resulted from an 8-O-4′ oxidative coupling between two caffeic acid units (Supplementary Figure 3). This compound was already described by Wan et al. (2008) as *rel*-(2*E*)-3-[(2*S*)-2-[(*R*)-(3,4-dihydroxyphenyl)hydroxymethyl]-1,3-benzodioxol-5-yl]-2-pro penoic acid from the biotransformation of caffeic acid with H_2_O_2_ and a *Momordica charantia* peroxidase.

**FIGURE 5:**
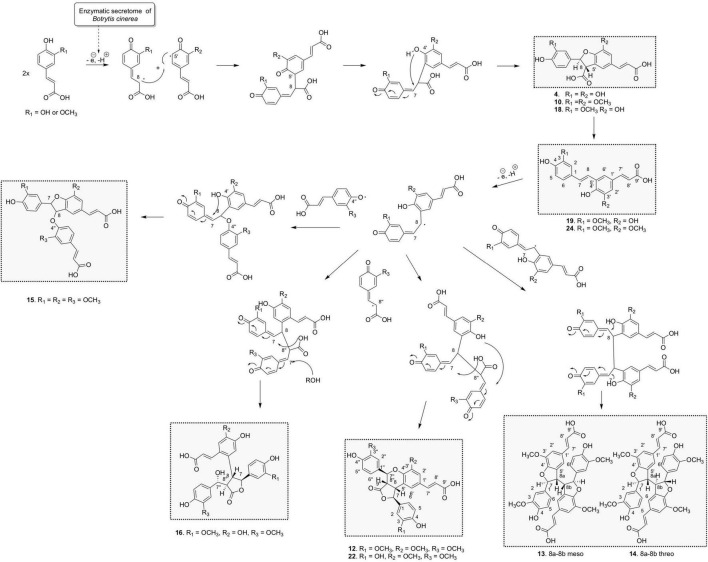
Proposed chemoenzymatic pathway for the formation of compounds 4, 10, 12-16, 18, 19, 22, and 24 by 8-5′ phenoxy radical coupling.

**FIGURE 6:**
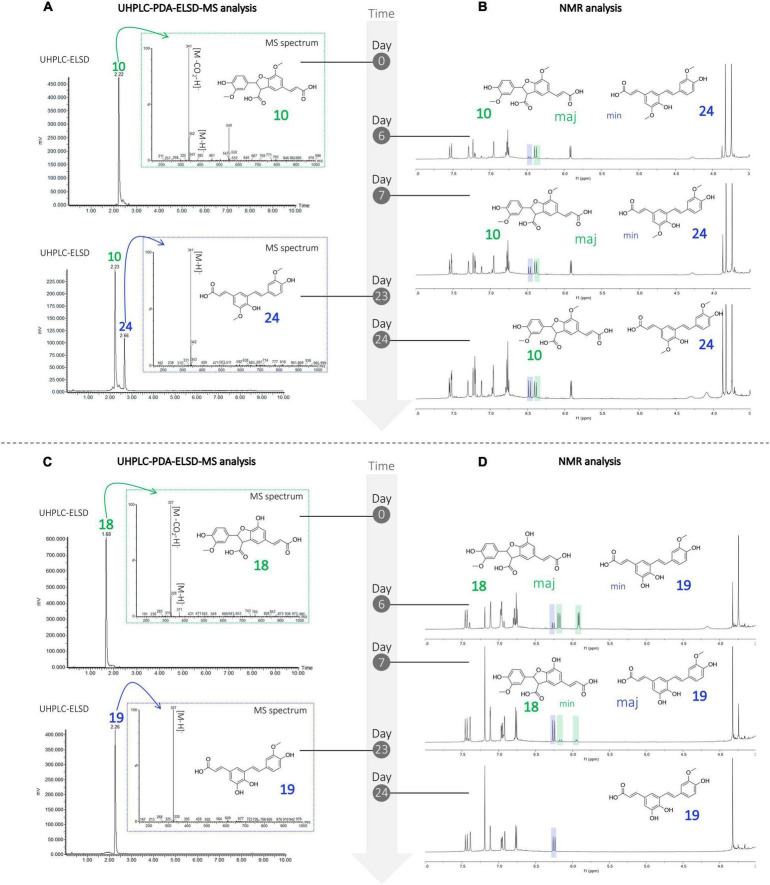
Monitoring of the degradation of the compounds undergoing decarboxylation **(A)** UHPLC-PDA-ELSD-MS and **(B)** NMR analysis showing the degradation of compound 10 in 24, **(C)** UHPLC-PDA-ELSD-MS and **(D)** NMR analysis showing the degradation of compound 18 in 19.

Three 8-5′-benzofuran dimers formed by 8-5′ oxidative coupling followed by 4′-O-7 cyclization (Figure 5) were identified. The first one, 8-5′-benzofuran-dicaffeic acid (4), has been described from the peroxidase-catalyzed polymerization of caffeic acid (Arrieta-Baez and Stark, 2006). The second one, 8-5′-benzofuran-diferulic acid (10), was identified from lignin peroxidase polymerization of ferulic acid (Ward et al., 2001). The third one, an 8-5′-benzofuran dimer formed between caffeic acid and ferulic acid (18), was also described by Arrieta-Baez and Stark (2006). Under our conditions, these three 8-5′-benzofuran dimers are decarboxylated but in different ways. The di-caffeic acid dimer (4) decarboxylated so slowly that the corresponding decarboxylated compound (4b) could not be identified. The decarboxylation of a di-ferulic acid dimer (10) was not total even after 24 days and gave off poacic acid (24) (Figure 6), an antifungal compound (Yue et al., 2017) described as a constituent of cell walls of various Gramineae (Ralph et al., 1994). In the NMR tube in DMSO-*d*_6_, compound 18 fully decarboxylated rapidly (less than 24 h) to give off a new derivative (19) (Figure 6).

The ESI(-)-HRMS analysis of compound 19 showed a molecular ion at *m/z* 327.0872 [M – H]^–^ corresponding to a molecular formula of C_18_H_15_O_6_ (calcd. for C_18_H_15_O_6_, 327.0869, Δ = 1.1 ppm), confirming the dimerization of caffeic and ferulic acids. The position of the methoxy group was deduced from the HMBC correlation of this methoxyl at δ_*H*_ 3.83 (3H, s, 3OCH_3_) to C-3 at δ_*C*_ 147.8 and from H-5 at δ_*H*_ 6.77 (1H, d, *J* = 8.2 Hz) to C-1 (δ_*C*_ 129.2) and C-3. Compound 19 was thus identified as (*E*)-3-(3,4-dihydroxy-5-((*E*)-4-hydroxy-3-methoxystyryl)phenyl)acrylic acid.

**FIGURE 7:**
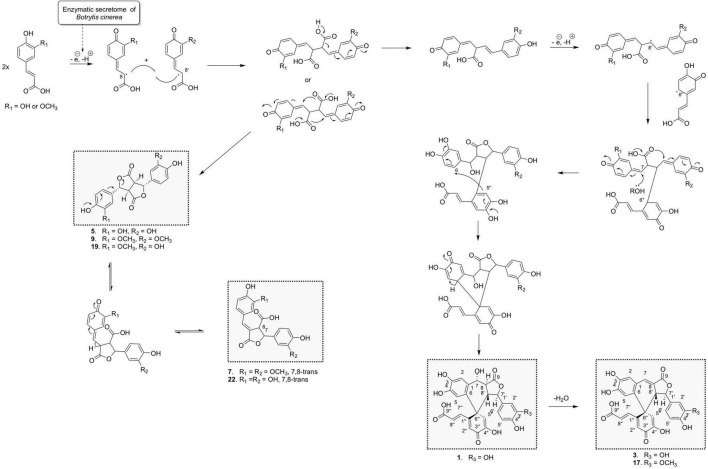
Proposed chemoenzymatic pathway for the formation of compounds 1, 3, 6, 7, 9, 17, 20, and 23 by 8-8′ phenoxy radical coupling.

The biotransformation reactions also generated dilactone dimers. For example, the dehydrodicaffeic acid dilactone (5) and dehydrodiferulic acid dilactone (9) resulted from an 8-8′ coupling (Figure 7), 5 was known to be produced by cultured mushroom, *Inonotus* sp. (Kumada et al., 1976), and 9 was known to be present in cell walls of wheat and barley (Turner et al., 1993). A new dilactone (20) formed by an 8-8′ coupling between caffeic acid and ferulic acid was identified. In contrast to compounds 5 and 9 in which only monomer signals (three aromatic and two methine signals) are observed in NMR, the signals of compound 20 corresponding to the caffeic moiety were observed at δ_*H*_ 6.81 (1H, d, *J* = 2.2 Hz, H-2), 6.77 (1H, d, *J* = 8.1 Hz, H-5), 6.71 (1H, dd, *J* = 8.1, 2.2 Hz, H-6), 5.67 (1H, d, *J* = 2.9 Hz, H-7), and 4.15 (1H, dd, *J* = 9.8, 2.9 Hz, H-8), whereas those of ferulic acid were observed at δ_*H*_ 6.99 (1H, d, *J* = 2.2 Hz, H-2′), 6.86 (1H, dd, *J* = 8.2, 2.1 Hz, H-6′), 6.79 (1H, d, *J* = 8.2 Hz, H-5′), 5.72 (1H, d, *J* = 3.0 Hz, H-7′), 4.08 (1H, dd, *J* = 9.8, 3 Hz, H-8′), and 3.8 (3H, s, 3′OCH_3_) because of loss of symmetry. This compound was named dehydrocaffeicferulic acid dilactone (20).

Surprisingly, in each reaction, dilactones were accompanied by a more polar and less intense peak: 5′ for the caffeic acid reaction (Figure 2), 9′ for the ferulic acid reaction (Supplementary Figure 1), and 20′ for the caffeic acid and ferulic acid reaction (Supplementary Figure 2). After isolation and analysis by UHPLC-MS and NMR, these compounds were found to be the same as the dilactones 5, 9, and 20, respectively (Figure 4). The caffeic acid biotransformation reaction was repeated to isolate compounds 5 and 5′, which were carefully dried without heating under nitrogen flow. Both compounds were analyzed directly after drying, and it was found that 5′ had already been re-transformed into 5. Because of this rapid transformation, the initial identity of compounds 5′, 9′, and 20′ could not be defined.

Furthermore, a degradation process also occurred under our conditions for these dilactones. For example, we observed that one of the lactone rings can open to give off almost quantitatively phellinsin A (23) in the case of dehydrodicaffeic acid dilactone (5) (Figure 4 and Supplementary Figure 4). The same behavior seems to occur with the dehydrodiferulic acid dilactone (9) since we were able to isolate the 8-8-γ-lactone-diferulic acid (7). The mixed dilactone 20 also appeared to undergo the same ring opening, but the compound formed was not obtained pure enough to be characterized. Indeed, unlike the decarboxylation products 19 and 24, the compounds generated by the ring opening underwent further degradation and gave off complex mixtures (Figure 4). These observations are confirmed by stability studies conducted on phellinsin A by Nemadziva et al. (2018) who showed that this type of compound is unstable when the temperature increases or at a pH higher than 7.5, and that it is sensitive to light.

The 7,8-*cis* isomer of compound 7 was also isolated as 8 and had never been described. On the ROESY spectrum, both 7 and 8 showed correlations between H-8 and H-2′/H-6′, indicating an *E* configuration of the double bond. For 7 the coupling constant between H-7 and H-8 was 2.9 Hz and an ROE correlation was observed between H-8 and H-2, confirming the *trans* configuration of H-7 and H-8 protons, whereas 8 *cis* configuration was indicated by the ROE correlation between H-7 and H-8 and a ^3^*J*_*H*7–H8_ value of 7.6 Hz.

Concerning the 9 trimers formed, 3 were already described. Compounds 11 and 15 were isolated by Ward et al. (2001) from ferulic acid polymerization by lignin peroxidase. The first one is (2*E*,2′*E*)-3,3′-[[2-hydroxy-2-(4-hydroxy-3-methoxyphen yl)ethylidene]bis[oxy(3-methoxy-4,1-phenylene)]]bis-2-propen oic acid (11) formed through two successive 8-O-4′ couplings and H_2_O addition (Supplementary Figure 3). The second one is *rel*-(2*E*)-3-[4-[[(2*R*,3*S*)-5-[(1*Z*)-2-Carboxyethenyl]-2,3-dihyd ro-2-(4-hydroxy-3-methoxyphenyl)-7-methoxy-3-benzofuranyl] oxy]-3-methoxyphenyl]-2-propenoic acid (15) that resulted from 8-5′ coupling followed by 8-O-4′′ coupling from compound 24 (Figure 5). The third one was *rel*-(2*E*) -3-[(1*R*,3a*R*,4*R*,9b*S*)-1,3a,4,9b-Tetrahydro-1,4-bis(4-hydroxy-3 -methoxyphenyl)-6-methoxy-3-oxo-3H-furo[3,4-c][1]benzopyr an-8-yl]-2-propenoic acid (12) already identified by Liu et al. (2005) from the biotransformation of ferulic acid by *Momordica charantia* peroxidase. It was formed by an 8-5′ coupling of two ferulic acids followed by 8-8′′ coupling with a third ferulic acid (Figure 5). Our reaction between caffeic acid and ferulic acid produced the same type of trimer, but with a caffeic acid group replacing one of the ferulic acid groups. This new derivative (22) showed a molecular ion in ESI(-)-HRMS at *m/z* 519.1291 [M – H]^–^ in agreement with a trimer structure. The NMR data of 22 showed closed similarities to those of 12 except that two methoxy groups were observed for 22 while three were observed for 12. The ROESY correlations from the aromatic H-2′ to the ethylenic H-8′ and the methoxyl 3′-OCH_3_, from the aromatic H-2′′ to the oxymethine H-7′′, the methine H-8′′ and the methoxyl 3′′-OCH_3_ in addition to the HMBC correlation of H-7′′ and H-2′ to C-4′ allowed to positioned the two methoxy groups. Compound 22 was thus defined as *rel*-(*E*)-3-((1*S*,3a*S*,4*S*,9b*R*)-1-(3,4-dihydroxyphenyl)-4-(4-hydroxy-3-methoxyphenyl)-6-methoxy-3-oxo-1,3a,4,9b-tet rahydro-3H-furo[3,4-c]chromen-8-yl)acrylic acid.

Compound 16 is a trimer, as indicated by its ESI(-)-HRMS ion at *m/z* 537.1401 [M – H]^–^, (calcd. for C_28_H_25_O_11_, 537.1397, Δ = 0.7 ppm). The ^1^H and COSY NMR data indicated the presence of two 1,3,4-trisubstituted aromatic cycles at δ_*H*_ 6.35 (1H, dd, *J* = 8, 2 Hz, H-6), 6.47 (1H, d, *J* = 2 Hz, H-2), and 6.57 (1H, d, *J* = 8 Hz, H-5) for the first one and δ_*H*_ 6.64 (1H, d, *J* = 8 Hz, H-5′′), 6.73 (1H, dd, *J* = 8, 1.9 Hz, H-6′′), and 6.82 (1H, d, *J* = 1.9 Hz, H-2′′) for the second one. The presence of two methoxy groups at δ_*H*_ 3.71 (3H, s, 3OCH_3_) and 3.73 (3H, s, 3′′OCH_3_) indicated that the trimer resulted from the coupling of two ferulic acids and one caffeic acid. The ROE correlations between 3OCH_3_ and H-2 and between 3′′OCH_3_ and H-2′′ positioned these methoxy groups on the two 1,3,4-trisubstituted aromatic cycles. The HMBC correlations from H-2′′ and H-6′′ to the oxymethine H-7′′ at δ_*H*_ 5.14 (1H, d, *J* = 5.4 Hz), from H-2 and H-6 to the oxymethine H-7 at δ_*H*_ 5.01 (1H, d, *J* = 9.5 Hz) and the COSY correlations from H-7 to H-8, H-8 to H-8′′, and H-8′′ to H-7′′ allowed to link these two ferulic entities. The HMBC correlation from the aromatic singlet H-5′ at δ_*H*_ 6.97 to H-8 and the second aromatic singlet H-2′ at δ_*H*_ 6.79 to the ethylenic proton H-7′ at δ_*H*_ 7.19 (1H, d, *J* = 15.5 Hz) positioned the caffeic acid group. Finally, a 7-O-9′′ cyclization made it possible to match the structure to the HRMS data. The NMR spectra recorded in DMSO-*d*_6_ showed a broad singlet at δ_*H*_ 5.41, which gives ROE correlation with H-7′ confirming the position of the hydroxy group. The *trans*–*trans* relative configuration of γ-butyrolactone was given by the ROE correlation from H-2 and H-6 to H-7 and H-8, from H-5′ to H-8, H-7 and H-8′′, and from H-7′′ to H-8 and H-8′′. The relative configuration in C-7′ was defined thanks to the following ROE correlations from H-7′′ to H-8′′ and H-8 and from H2′′/H-6′′ to H-8 and H-7′ (Supplementary Figure 5). This compound was formed by 8-5′ coupling between ferulic acid and caffeic acid, which give off 19, followed by an 8-8′′ coupling with a second ferulic acid, cyclization between the acid group, and C-7 and H_2_O addition in C-7′′ (Figure 5). Compound 16 was thus identified as *rel*-(*E*)-3-(4,5-dihydroxy-2-((2*S*,3*R*,4*R*)-4-((*R*)-hydroxy(4-hydroxy-3-methoxyphenyl)methyl)-2-(4-hydroxy-3-methoxyphenyl)-5-oxotetrahydrofuran-3-yl)phenyl)acrylic acid.

The ESI(-)-HRMS of compound 21 displayed a [M – H]^–^ ion at *m/z* 519.1294 corresponding to a molecular formula of C_28_H_23_O_10_ (calcd. for C_28_H_23_O_10_, 519.1291, Δ = 0.6 ppm) and thus to a trimer. The ^1^H and edited HSQC confirmed the presence of two *trans* olefinic protons at δ_*H*_ 6.15 (1H, d, *J* = 15.4 Hz, H-8′) and 7.62 (1H, d, *J* = 15.4 Hz, H-7′), as well as three aromatic groups with the same substitution as that of 16, i.e., two 3-methoxy-4-hydroxyphenyl and one caffeic acid substituted in C-6. In addition, the following signals were observed: an oxymethine at δ_*H*_ 5.61 (1H, d, *J* = 2.6 Hz, H-8) with HMBC correlations to C-5′ and C-6′, a methine at δ_*H*_ 4.38 (1H, t, *J* = 2.6, 2.1 Hz, H-7) with HMBC correlations to C-1 and C-2, and a downfield olefinic proton at δ_*H*_ 7.65 (1H, d, *J* = 2.1 Hz, H-7′′) with HMBC correlations to C-2′′ and C-6′′. A 3-methylenedihydrofuranone was identified thanks to the HMBC correlations from H-7, H-8, and H-7′′ to the carbonyl C-9′′ at δ_*C*_ 172.3. The ROE correlations from H-6′′ to H-7 defined the double bond as (*E*) and those from H-7 to H-5′ and from H-8 to H-2 a *trans* configuration of H-7 and H-8 protons. This compound should be formed by an 8-6′ coupling between ferulic acid and caffeic acid followed by decarboxylation. Then a 7-8′′ coupling with another ferulic acid followed by cyclization between the acid and C-8 should occur (Supplementary Figure 6). Compound 21 was thus identified as *rel*-(*E*)-3-(4,5-dihydroxy-2-((2*S*,3*S*)-4-((*E*)-4-hydroxy-3-meth oxybenzylidene)-3-(4-hydroxy-3-methoxyphenyl)-5-oxotetrahy drofuran-2-yl)phenyl)acrylic acid.

**FIGURE 8:**
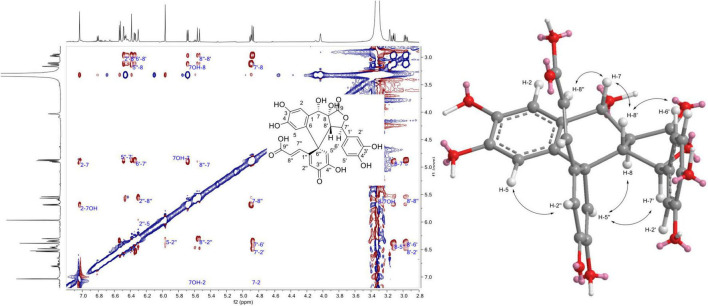
^1^H-^1^H Rotating frame Overhauser Effect SpectroscopY (ROESY) correlations observed for compound 1.

The ^1^H NMR data of 1, 3, and 17 showed that they share the same aromatic profile: four singlet protons, three protons belonging to a 3,4-dihydroxyphenyl group, and two *trans* olefinic protons. Compound 1 displayed an ESI(-)-HRMS ion at *m/z* 507.0943 [M - H]^–^, (calcd. for C_26_H_19_O_11_, 507.0927, Δ = 3.2 ppm) indicating a trimer. The ^1^H and edited-HSQC indicated that in addition to the aromatic signals, the following signals were observed: an oxymethine proton at δ_*H*_ 4.89 (1H, t, *J* = 9.9, 7.9 Hz, H-7) with its OH proton at δ_*H*_ 5.69 (1H, d, *J* = 7.9 Hz, 7-OH), an oxymethine at δ_*H*_ 4.88 (1H, d, *J* = 10.2 Hz, H-7′), and two methine protons at δ_*H*_ 3.12 (1H, dd, *J* = 14.2, 9.9 Hz, H-8), and 2.96 (1H, dd, *J* = 14.2, 10.2 Hz, H-8′). These protons were linked together scalar coupling from 7-OH to H-7, H-8, H-8′, and H-7′. The 3,4-dihydroxyphenyl group at δ_*H*_ 6.34 (1H, dd, *J* = 8.1, 2.1 Hz, H-6′), 6.48 (1H, d, *J* = 2.1 Hz, H-2′), and 6.53 (1H, d, *J* = 8.1 Hz, H-5′) were linked to H-7′ thanks to the HMBC correlations of H-2′ and H-6′ to C-7′ at δ_*C*_ 81.4 (C-7′). The HMBC correlations of H-7, H-8, and H-7′ to the carbonyl at δ_*C*_ 173.5 (C-9) allowed to form butyrolactone. The correlations from H-2 and H-5 to the same *sp*^2^ carbon at δ_*C*_ 125 (C-6), 132.2 (C-1), 145 (C-4), and 145.1 (C-3) showed that they belonged to the same aromatic group and the correlation from H-2 to C-7 at δ_*C*_ 65.9 (C-7) linked this 3,4-dihydroxyphenyl group substituted in C-6 to C-7. The correlations from both H-2′′ at δ_*H*_ 6.3 (1H, s, H-2′′) and H-5′′δ_*H*_ 6.39 (1H, s, H-5′′) to the hydroxy *sp*^2^ carbon C-4′′ at δ_*C*_ 146.3 and the quaternary carbon C-6′′ at δ_*C*_ 47.7 positioned H-2′′ and H-5′′ on the same ring but also indicated a 2-hydroxycyclohexa-2,5-dien-1-one ring, as confirmed by the correlation of H-5′′ to the carbonyl C-3′′ at δ_*C*_ 181.1. The *trans* olefinic protons were linked to C-1′′ because of the HMBC correlation of H-8′′ at δ_*H*_ 5.55 (1H, d, *J* = 16 Hz, H-8′′) with C-1′′ at δ_*C*_ 156.9. Finally, the correlation between H-5 and C-6′′ allowed to establish the structure of 1 (Figure 4). The relative configuration was not easy to establish because of a strong TOCSY effect between H-8 and H-8′ in the ROESY experiment as indicated by the peak with the same phase as the diagonal between these two protons. These protons became thus a good relay *via* TOCSY and can give false ROEs. However, the ROEs from H-5′′ to H-7′ and H-8, from H-2′′ to H-5, from H-7 to H-8′′, and H-8′ and from H-2′/H-6′ to H-8′ were considered as key to defining the relative configuration of this compound (Figure 8). Compound 1 was named spiro caffeic acid trimer.

The ESI(-)-HRMS of 3 displayed a [M – H]^–^ ion at *m/z* 489.0839 corresponding to a molecular formula of C_26_H_17_O_10_, (calcd. for C_26_H_17_O_10_ 489.0822, Δ = 3.4 ppm), which indicated that the compound obtained was the result of decarboxylation and e fusion of three units of caffeic acid. The NMR data of 3 showed close similarities to those of compound 1, except that the oxymethine proton H-7 with its OH proton and the methine proton H-8 of compound 1 was not present in 3. On the contrary, an additional olefinic proton was detected in 3 at δ_*H*_ 7.53 (1H, d, *J* = 3.3 Hz, H-7). The HMBC correlations of this proton to C-8′ at δ_*C*_ 48.6, C-2 at δ_*C*_ 118.1, C-6 at δ_*C*_ 127.1, and C9 at δ_*C*_ 167.5 allowed to position this proton and to elucidate 3 as the new biotransformed form of caffeic acid described in Figure 4. The ROE correlations from H-7′ to H-5′′ and from H-8′ to H-7′′, H-2′ and H-6′ provided indications on the relative configuration of compound 3 (Supplementary Figure 7). Compound 3 was named 7-hydroxyspirocaffeic acid trimer.

The NMR data of 17 showed close similarities to those of 3, except that a methoxy group at δ_*H*_ 3.65 (3H, s, 3′OCH_3_) was observed in 17. The methoxy was positioned in C-3′ thanks to its ROE correlation with H-2′ at δ_*H*_ 6.9 (1H, d, *J* = 2 Hz, H-2′). All the NMR and HRMS data agree with the structure depicted in Figure 4. Compound 17 was named 2′-*O*-methylspirocaffeic acid trimer.

The ESI(-)-HRMS of compounds 13 and 14 displayed [M – H]^–^ ions at *m/z* 681.2004 and 681.2005, respectively, corresponding to a molecular formula of C_38_H_33_O_12_ and thus to tetramers. The NMR data of these compounds showed protons corresponding to a 3-methoxy,4-hydoxypenyl group, two meta-coupled aromatic protons, two *trans* olefinic protons, and an oxymethine and methine protons. These data were thus closed to those of 8-5′-benzofuran diferulic acid (10), except that only one carbonyl was observed at δ_*C*_ 171 for 13 and 171.3 for 14. This carbonyl was assigned to the acid linked to the *trans* olefinic carbons and thus no acid was detected in C-8. This information, combined with the HRMS, data led to the conclusion that 13 and 14 are symmetric dimers. The ^3^*J* coupling constant between H-7 and H-8 was 5.6 Hz for 13 and 3.4 Hz for 14, but both compounds showed ROE correlations from H-8 to H2/H-6, suggesting a *trans* configuration between H-7 and H-8 in both molecules. The difference should thus be the configuration between H-8a and H-8b (Figure 4). The *meso* form was assigned to 13 and the *threo* to 14 by comparison with the bisbenzofuran obtained by Chong et al. (2017) from anodic oxidation of 3,4-dimethoxy-2′hydroxystilbene. Their *meso-*bisbenzofuran was characterized by a ^3^*J*_*H–*7–H–8_ value of 6.9 Hz (3.4 Hz for the *threo* form) and more deshielded aromatic H-2, H-3, and H-6 protons. Compounds 13 and 14 were named *meso*-8-5′-benzofuran tetra-ferulic acid and *threo*-8-5′-benzofuran tetra-ferulic acid, respectively.

Overall, the three biotransformation reactions afforded a total of 24 compounds. All of these compounds are most likely produced by an oxidative coupling reaction produced by the laccase present in the *Botrytis cinerea* secretome (Pezet, 1998). In the case of caffeic and ferulic acid, the phenoxy radicals produced could form different types of coupling through delocalization of the radical. From the 8-5′ coupling (Figure 5), five dimers (4, 10, 18, 19, and 24), four trimers (15, 16, 12, and 22), and two tetramers (13 and 14) were formed. Among these compounds, the scaffold of one trimer (16), as well as that of the tetramers, is new. From the 8-8′ coupling (Figure 7), six dimers (5, 7–9, 20, and 23) and three tetramers (1, 3, and 17) were produced including a completely new scaffold with a spiro carbon. From the 8-O-4′ coupling (Supplementary Figure 3), a dimer (6) and a trimer (11) already described were isolated. Finally, a new trimer (21), which should be formed by 8-6′ coupling (Supplementary Figure 6) followed by decarboxylation, then a 7-8′′ coupling with another unit followed by cyclization gave off a new scaffold.

### Biological Screening of the Generated Compounds on Wnt Inhibition

The biotransformation products were analyzed for their activity against the Wnt pathway (Table 1). To this end, we have employed a BT-20 triple-negative breast cancer cell line stably transfected by an 8× TopFlash reporter and stimulated by a purified Wnt3a ligand (Koval et al., 2014; Shaw et al., 2019a). The cells were additionally transfected by a CMV-driven *Renilla* luciferase construct, which is expressed constitutively and allows us to distinguish specific inhibition of the pathway from the reduction of the signal caused by acute cytotoxic or unspecific (e.g., inhibition of transcription/translation) activity. Interestingly, we have found that biotransformation resulted in the generation of several low-potency Wnt inhibition-specific scaffolds (compounds 3, 6, 21, 14, 23, and 24) and that the original compounds possessed no specific activity.

**TABLE 1:**
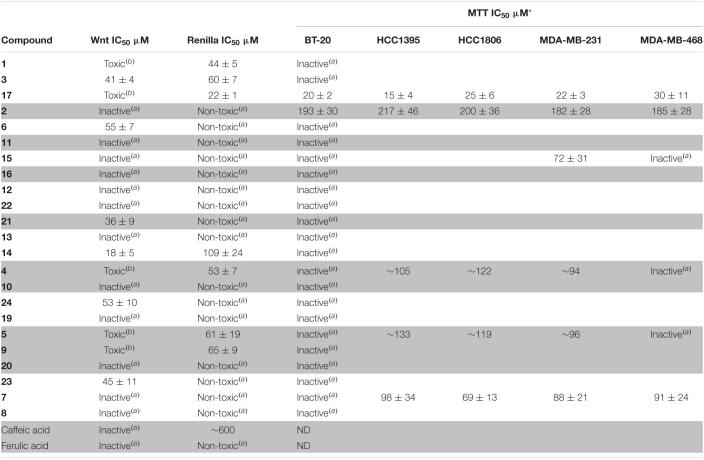
Inhibition of the Wnt pathway with the biotransformation products.

*Gray/white background highlights compounds with similar scaffold.*

*The cell lines used were chosen to represent a broad variety of TNBC histotypes: luminal-like ductal (BT-20), squamous (HCC1806), and adenocarcinoma (MDA-MB-468); basal-like ductal (HCC1395) and adenocarcinoma (MDA-MB-231).

*^(a)^Compounds are considered inactive and non-toxic if the highest concentration tested does not show a 20% reduction compared to the control.*

*^(b)^Compounds are considered “toxic” if the IC_50_ value against Renilla luciferase is less than 1.7-fold of the estimated TopFlash one (i.e., SI < 1.7), indicating that the decrease in observed signal in the TopFlash is due to or strongly affected by the toxic effect.*

None of these compounds have ever been reported to possess a Wnt inhibitory activity before. Surprisingly, we did not observe any long-term inhibition of TNBC cell proliferation in a 3-day MTT assay by the Wnt-inhibiting compounds. On the other hand, the majority of the compounds (except 17) that showed toxicity in the Renilla assay (24 h) demonstrated comparatively less potency in inhibition of cell proliferation over 3 days. Combined with a similar trend for compounds inhibiting Wnt signaling, this suggests that these derivatives are sufficiently stable under culture conditions for only 24 h and that the cells recover quickly from the initial impact.

## Experimental Section

### General Experimental Procedures

The UV and ECD spectra were recorded on a JASCO J-815 spectrometer (Loveland, CO, United States) in MeOH or MeCN using a 1-cm cell. Scan speed was set at 200 nm/min in continuous mode between 600 and 190 nm. NMR spectroscopic data were recorded on a Bruker Avance Neo 600 MHz NMR spectrometer equipped with a QCI 5-mm cryoprobe and a SampleJet automated sample changer (Bruker BioSpin, Rheinstetten, Germany). One-dimensional (1D) and 2D NMR experiments (^1^H, COSY, ROESY, HSQC, and HMBC) were recorded in DMSO-*d*_6_ or CD_3_OD. Chemical shifts were reported in parts per million (δ) and coupling constants (*J*) in Hz. The residual DMSO-*d*_6_ signal (δ_*H*_ 2.5; δ_*C*_ 39.5) and CD_3_OD signal (δ_*H*_ 3.31; δ_*C*_ 49) were used as internal standards for ^1^H and ^13^C NMR, respectively. *Trans*-caffeic acid (reference C0625-25 g, >98% purity) was purchased from Sigma-Aldrich (Steinheim, Germany), and *trans*-ferulic acid (reference 9936, 99% purity) was purchased from Roth (Karlsruhe, Germany).

### Secretome Isolation From *Botrytis cinerea* Culture

*Botrytis cinerea* Pers.:Fr., isolate K16, was obtained from naturally sporulated grape berries from Changins Agroscope experimental vineyards in 2015. The strain was purified, determined phenotypically as well as molecular tools (sequencing of ITS regions), and maintained by regular transplanting. The fungus was grown in an oatmeal agar medium (Difco), and conidia were sampled by vacuum aspiration and stored dry at −80°C until use. *B. cinerea* was cultivated in a 1.5-L liquid medium (in 5 L bottles) at 22°C under alternating light and dark (12 h/12 h). The medium was filtrated through a double layer of folded filter paper (diameter 500 mm; Prat Dumas, Couze-St-Front, France). The filtrate was brought to 80% saturation [(NH_4_)_2_SO_4_, 24 h at 4°C] and centrifuged (4,200 *g*, 4°C, 2 h). The supernatant was discarded, and the resulting pellet was solubilized in nanopure water (4.21 μS cm^–1^; Evoqua Water Technologies, Pittsburgh, PA, United States) (the protein crude extract) and submitted to dialysis (spectra/por 1 dialysis membrane, 6–8 kDa, diameter 14.6 mm) against nanopure water overnight at 4°C. The resulting protein extract was concentrated against polyethylene glycol beads (PEG 20,000) in the dialyzed tube. Protein content was determined by the method of Bradford, using a Bio-Rad protein assay kit with BSA as a standard. The volume of the extract was adjusted to obtain a final protein concentration of 2 μg/μl. The resulting extract (considered as secretome) was aliquoted to 1 ml and stored at −20°C until use.

### Analytical Scale Biotransformation Reactions

The biotransformation reactions of caffeic acid, ferulic acid, and the mixture of both were performed on an analytical scale in 2-ml Eppendorf tubes. The total volume of the reactions was 1 ml, with a total substrate concentration of 1 mg/ml (0.5 mg/ml of each compound for the mixture) and an acetone volume of 10% (100 μl). The secretome was always added last, and its amount varied from 1 to 10% (10–100 μl). Water volume was adapted accordingly to fit the total volume (890–800 μl). Once prepared, the mixtures were incubated at room temperature in the dark under constant gentle agitation. Aliquots were collected after 15 min and 5 h and dried under vacuum in a centrifugal evaporator. The reactions were stopped after 24 h by removing the solvent under vacuum in a centrifugal evaporator. A total of 1 ml of MeOH was then added to the dry deposit. The sample was centrifugated, and the supernatants were analyzed by UHPLC-PDA-ELSD-MS.

### UHPLC-PDA-ELSD-MS Analysis

The crude reaction mixtures were analyzed on an ultra-high-performance liquid chromatography system equipped with a photodiode array, an evaporative light-scattering detector, and a single quadrupole detector by heated electrospray ionization (UHPLC-PDA-ELSD-MS) (Waters, Milford, MA, United States). ESI parameters were the following: capillary voltage 800 V, cone voltage 15 V, source temperature 120°C, and probe temperature 600°C. The acquisition was performed in negative ionization (NI) mode with an *m/z* range of 150–1,000 Da. Chromatographic separation was performed on an Acquity UPLC BEH C18 column (50 mm × 2.1 mm i.d., 1.7 μm; Waters, Milford, MA, United States) at 6 ml/min, 40°C with H_2_O (A) and MeCN (B), both containing.1% formic acid, as solvents. The gradient was carried out as follows: 5–100% B in 7 min, 1 min at 100% B, and a re-equilibration step at 5% B for 2 min. The ELSD temperature was fixed at 45°C, with a gain of 9. The PDA data were acquired from 190 to 500 nm, with a resolution of 1.2 nm. The sampling rate was set at 20 points/s. Data were processed with the MassLynx software (Waters, Milford, MA, United States).

### UHPLC-PDA-CAD-HRMS Analysis

The pure compounds were analyzed on a Waters Acquity UHPLC system equipped with a Q-Exactive Focus mass spectrometer (Thermo Fisher Scientific, Bremen, Germany), using a heated electrospray ionization source (HESI-II). Chromatographic separation was carried out on an Acquity UPLC BEH C18 column (50 mm × 2.1 mm i.d., 1.7 μm; Waters) at 6 ml/min, 40°C with H_2_O (A) and MeCN (B), both containing 1% formic acid, as solvents. The gradient was carried out as follows: 5–100% B in 7 min, 1 min at 100% B, and a re-equilibration step at 5% B in 2 min. The ionization parameters were the same as those used in Rutz et al. (2019).

### Large Scale Biotransformation Reactions

The biotransformation reactions were performed on a large scale by keeping the most efficient parameters tested on the analytical scale. The biotransformation reaction of caffeic acid was performed in a 100-ml Schott bottle with 100 mg of starting material and a total volume of 100 ml (1 mg/ml substrate concentration) for 24 h. The percentage of acetone was kept at 10% (10 ml), the amount of secretome was 1% (1 ml), and the water volume was 89 ml. The biotransformation reaction of ferulic acid was performed in a 100-mL Schott bottle with 100 mg of starting material and a total volume of 100 ml (1 mg/ml substrate concentration) for 24 h. The percentage of acetone was kept at 10% (10 ml), the amount of secretome was 1% (1 ml), and the water volume was 89 ml. The biotransformation reaction of caffeic acid/ferulic acid mixture was performed in a 100-ml Schott bottle with 100 mg of starting material (50 mg of caffeic acid and 50 mg of ferulic acid) and a total volume of 100 ml (1 mg/ml substrate concentration) for 24 h. The percentage of acetone was kept at 10% (10 ml), the amount of secretome was 1% (1 ml), and the water volume was 89 ml. The reaction mixtures were incubated at room temperature in the dark under constant gentle magnetic stirring. After evaporation of the solvent with a rotatory evaporator, the dry deposit was resuspended in MeOH and filtered through.45-μm filters (13-mm syringe filter, PVDF; BGB Analytik, Böckten, Switzerland). These crude reaction mixtures were analyzed by UHPLC-PDA-ELSD-MS for comparison with analytical scale reactions before moving to gradient optimization and semi-preparative HPLC fractionation.

### Purification of the Crude Reaction Mixtures by Semi-Preparative HPLC-UV

The separation conditions of the three crude reaction mixtures were optimized on the UHPLC-PDA-ELSD-MS system with an Acquity UPLC BEH C18 column (100 mm × 2.1 mm i.d., 1.7 μm; Waters, Milford, MA, United States) at 4 ml/min, 40°C with H_2_O (A) and MeOH (B), both containing 1% formic acid, as solvents. The optimized gradient conditions for each reaction were geometrically transferred by gradient transfer to the analytical and semi-preparative HPLC scale (Supplementary Table 1, for each chromatographic condition). The geometrically transferred gradients were first tested on an HP 1260 Agilent High-Performance liquid chromatography device equipped with a photodiode array detector (HPLC-PDA) (Agilent Technologies, Santa Clara, CA, United States). Chromatographic separation was performed on an XBridge C18 column (250 mm × 4.6 mm i.d., 5 μm; Waters, Milford, MA, United States) equipped with a C18 pre-column at 1 ml/min, with H_2_O (A) and MeOH (B), both containing 1% formic acid, as solvents. UV absorbance was measured at 210 and 254 nm, and UV-PDA spectra were recorded between 190 and 600 nm (step 2 nm). The geometrically transferred gradients were used on the semi-preparative scale in a Shimadzu system equipped with an LC-20 A module pump, an SPD-20 A UV/VIS, a 7725I Rheodyne^®^ valve, and an FRC-40 fraction collector (Shimadzu, Kyoto, Japan). Separation was performed on a Waters XBridge C18 column (250 mm × 19 mm i.d., 5 μm) equipped with a Waters C18 pre-column cartridge holder (10 mm × 19 mm i.d., 5 μm) at 17 ml/min, with H_2_O (A) and MeOH (B), both containing 1% formic acid, as solvents. UV detection was set at 210 and 254 nm. The mixtures were injected on the semi-preparative HPLC column using a dry load methodology developed in our laboratory (Queiroz et al., 2019).

### Fractionation of the Caffeic Acid Biotransformation Reaction Crude Mixture

Two semi-preparative HPLC injections of about 40 mg each yielded 50 fractions each. The fractions were analyzed by UHPLC-PDA-ELSD-MS and mixed together according to their composition. Six compounds were obtained pure: 1 (0.6 mg, *t*_*R*_ 14 min), 2 (0.7 mg, *t*_*R*_ 15.9 min), 3 (3 mg, *t*_*R*_ 27.2 min), 4 (2.7 mg, *t*_*R*_ 33.4 min), 5 (12.2 mg, *t*_*R*_ 34.2 min), and 6 (2.1 mg, *t*_*R*_ 42.7 min).

### Fractionation of the Ferulic Acid Biotransformation Reaction Crude Mixture

Two semi-preparative HPLC injections of about 40 mg each yielded 45 fractions each. The fractions were analyzed by UHPLC-PDA-ELSD-MS and mixed together according to their composition. Nine compounds were obtained pure: 7 (1.6 mg, *t*_*R*_ 15.5 min), 8 (0.2 mg, *t*_*R*_ 19.3 min), 9 (5.7 mg, *t*_*R*_ 20.6 min), 10 (2 mg, *t*_*R*_ 22.8 min), 11 (0.9 mg, *t*_*R*_ 27.2 min), 12 (3.1 mg, *t*_*R*_ 28.4 min), 13 (0.7 mg, *t*_*R*_ 34.3 min), 14 (0.6 mg, *t*_*R*_ 39.4 min), and 15 (1.5 mg, *t*_*R*_ 41.3 min).

### Fractionation of the Caffeic Acid/Ferulic Acid Biotransformation Reaction Crude Mixture

Two semi-preparative HPLC injections of about 45 mg each yielded 55 fractions each. The fractions were analyzed by UHPLC-PDA-ELSD-MS and mixed according to their composition. Six compounds were obtained pure: 16 (0.7 mg, *t*_*R*_ 15.8 min), 17 (0.1 mg, *t*_*R*_ 20.5 min), 19 (1 mg, *t*_*R*_ 28 min), 20 (10.9 mg, *t*_*R*_ 29.5 min), 21 (0.6 mg, *t*_*R*_ 35.1 min), and 22 (0.8 mg, *t*_*R*_ 42.7 min). Two compounds were isolated first as 5 (3.6 mg, *t*_*R*_ 22.2 min) and as 10 (1.2 mg, *t*_*R*_ 40.7 min), but ring opening occurred during the NMR analysis of 5 that led to compound 23, and decarboxylation occurred during the NMR analysis of 10 that led to compound 24.

### Description of the Isolated Compounds

Spiro caffeic acid trimer (1). UV (MeOH) λmax (log ε) 237 (sh) (4.05), 287 (3.91), 330 (3.59) nm. ^1^H NMR (DMSO-*d*_6_, 600 MHz) δ 2.96 (1H, dd, *J* = 14.2, 10.2 Hz, H-8′), 3.12 (1H, dd, *J* = 14.2, 9.9 Hz, H-8), 4.88 (1H, d, *J* = 10.2 Hz, H-7′), 4.89 (1H, t, *J* = 9.9, 7.9 Hz, H-7), 5.55 (1H, d, *J* = 16 Hz, H-8′′), 5.69 (1H, d, *J* = 7.9 Hz, 7OH), 5.97 (1H, s, H-5), 6.3 (1H, s, H-2′′), 6.34 (1H, dd, *J* = 8.1, 2.1 Hz, H-6′), 6.39 (1H, s, H-5′′), 6.44 (1H, d, *J* = 16 Hz, H-7′′), 6.48 (1H, d, *J* = 2.1 Hz, H-2′), 6.53 (1H, d, *J* = 8.1 Hz, H-5′), 7.03 (1H, s, H-2), 8.9 (1H, s, 4OH), 9.03 (1H, s, 4′′OH), 9.06 (1H, s, 4′OH), 9.07 (1H, s, 3OH); ^13^C NMR (DMSO-*d*_6_, 151 MHz) δ 44.6 (C-8), 47.7 (C-6′′), 49.6 (C-8′), 65.9 (C-7), 81.4 (C-7′), 112.2 (C-5), 114.5 (C-5′), 114.8 (C-2, C-2′, C-7′′), 118.7 (C-6′), 120.3 (C-5′′), 125 (C-6), 126.3 (C-1′), 127.8 (C-2′′), 132.2 (C-1), 144.7 (C-3′), 145 (C-4), 145.1 (C-3), 145.9 (C-4′), 146.3 (C-4′′), 156.9 (C-1′′), 173.5 (C-9), 181.1 (C-3′′). ESI(-)-HRMS *m/z* 507.0943 [M – H]^–^, (calcd. for C_26_H_19_O_11_, 507.0927, Δ = 3.2 ppm). MS/MS spectrum: CCMSLIB00006718008.

3,4-Dihydroxybenzaldehyde (2) (Kang et al., 2004). ^1^H NMR (DMSO-*d*_6_, 600 MHz) δ 6.9 (1H, d, *J* = 8.1 Hz, H-5), 7.23 (1H, d, *J* = 2 Hz, H-2), 7.27 (1H, dd, *J* = 8.1, 2 Hz, H-2, H-6), 9.7 (1H, s, H-7); ^13^C NMR (DMSO-*d*_6_, 151 MHz) δ 113.9 (C-2), 115.2 (C-5), 124.1 (C-6), 128.4 (C-1), 145.5 (C-3), 151.8 (C-4), 190.6 (C-7). ESI(-)-HRMS *m/z* 137.0245 [M – H]^–^, (calcd. for C_7_H_5_O_3_, 137.0239, Δ = 4.3 ppm).

7-Hydroxyspirocaffeic acid trimer (3). UV (MeOH) λmax (log ε) 264 (4.13), 318 (3.83), 350 (3.74) nm. ^1^H NMR (DMSO-*d*_6_, 600 MHz) δ 3.73 (1H, dd, *J* = 8.5, 3.3 Hz, H-8′), 4.89 (1H, d, *J* = 8.5 Hz, H-7′), 5.74 (1H, d, *J* = 15.9 Hz, H-8′′), 6.03 (1H, s, H-5′′), 6.22 (1H, s, H-5), 6.43 (1H, dd, *J* = 8.1, 2.2 Hz, H-6′), 6.54 (1H, d, *J* = 2.2 Hz, H-2′), 6.6 (1H, d, *J* = 8.1 Hz, H-5′), 6.73 (1H, s, H-2′′), 6.87 (1H, d, *J* = 15.9 Hz, H-7′′), 7.02 (1H, s, H-2), 7.53 (1H, d, *J* = 3.3 Hz, H-7); ^13^C NMR (DMSO-*d*_6_, 151 MHz) δ 48.6 (C-8′), 48.9 (C-6′′), 81.5 (C-7′), 113.1 (C-5), 114.7 (C-2′), 115 (C-5′), 118.1 (C-5′′), 118.1 (C-2), 118.6 (C-6′), 123 (C-8), 123.7 (C-1), 126.8 (C-8′′), 127.1 (C-6), 128 (C-1′), 130.8 (C-2′′), 131.8 (C-7), 138.2 (C-7′′), 145 (C-3′), 145.3 (C-3), 146 (C-4′), 147.5 (C-4′′), 148.8 (C-4), 155.2 (C-1′′), 166.3 (C-9′′), 167.5 (C-9), 181 (C-3′′). ESI(-)-HRMS *m/z* 489.0839 [M – H]^–^, (calcd for C_26_H_17_O_10_, 489.0822, Δ = 3.4 ppm). MS/MS spectrum: CCMSLIB00006717996.

8-5′-Benzofuran-dicaffeic acid (4) (Tazaki et al., 2001; Arrieta-Baez and Stark, 2006). ^1^H NMR (DMSO-*d*_6_, 600 MHz) δ 4.18 (1H, d, *J* = 7.1 Hz, H-8), 5.84 (1H, d, *J* = 7.1 Hz, H-7), 6.2 (1H, d, *J* = 15.9 Hz, H-8′), 6.65 (1H, dd, *J* = 8.1, 2.1 Hz, H-6), 6.71 (1H, d, *J* = 8.1 Hz, H-5), 6.74 (1H, d, *J* = 2.1 Hz, H-2), 7 (1H, d, *J* = 1.7 Hz, H-2′), 7.1 (1H, d, *J* = 1.7 Hz, H-6′), 7.45 (1H, d, *J* = 15.9 Hz, H-7′); ^13^C NMR (DMSO-*d*_6_, 151 MHz) δ 55.6 (C-8), 87 (C-7), 113.2 (C-2), 115.5 (C-5), 115.8 (C-8′), 115.9 (C2′), 116.2 (C-6′), 117.2 (C-6), 127.6 (-1′), 131.2 (C-1), 144.3 (C-7′), 145.3 (C3), 145.5 (C-4), 148.7 (C-4′), 167.7 (C-9′), 171.7 (C-9). ESI(-)-HRMS *m/z* 357.0611 [M – H]^–^, (calcd for C_18_H_13_O_8_, 357.061, Δ = 0.3 ppm). MS/MS spectrum: CCMSLIB00006718010.

Dehydrodicaffeic acid dilactone (5) (Kumada et al., 1976). ^1^H NMR (DMSO-*d*_6_, 600 MHz) δ 4.03 (2H, d, *J* = 1.5 Hz, H-8, H-8′), 5.66 (2H, d, *J* = 1.8 Hz, H-7, H-7′), 6.72 (2H, dd, *J* = 8.2, 2.2 Hz, H-6, H-6′), 6.76 (2H, d, *J* = 8.2 Hz, H-5, H-5′), 6.8 (2H, d, *J* = 2.2 Hz, H-2, H-2′), 9.09 (2H, s, OH), 9.2 (2H, s, OH); ^13^C NMR (DMSO-*d*_6_, 151 MHz) δ 48.3 (C-8, C-8′), 81.9 (C-7, C-7′), 113.7 (C-2, C-2′), 115.7 (C-5, C-5′), 117.4 (C-6, C-6′), 129.1 (C-1, C-1′), 145.5 (C-3, C-3′), 146.1 (C-4, C-4′), 175.3 (C-9, C-9′). ESI(-)-HRMS *m/z* 357.0621 [M – H]^–^, (calcd for C_18_H_13_O_8_, 357.061, Δ = 3.1 ppm). MS/MS spectrum: CCMSLIB00006718000.

*rel*-(2E)-3-[(2S)-2-[(R)-(3,4-Dihydroxyphenyl)hydroxymeth yl]-1,3-benzodioxol-5-yl]-2-propenoic acid (6) (Wan et al., 2008). UV (MeOH) λmax (log ε) 234 (sh) (4), 287 (3.87), 326 (3.86) nm. ^1^H NMR (DMSO-*d*_6_, 600 MHz) δ 4.64 (1H, t, *J* = 5, 3.6 Hz, H-7), 5.79 (1H, d, *J* = 5 Hz, 7OH), 6.26 (1H, d, *J* = 3.6 Hz, H-8), 6.35 (1H, d, *J* = 15.9 Hz, H-8′), 6.68 (2H, s, H-5, H-6), 6.83 (1H, d, *J* = 8 Hz, H-5′), 6.85 (1H, d, *J* = 1.4 Hz, H-2), 7.08 (1H, dd, *J* = 8, 1.7 Hz, H-6′), 7.25 (1H, d, *J* = 1.7 Hz, H-2′), 7.47 (1H, d, *J* = 15.9 Hz, H-7′); ^13^C NMR (DMSO-*d*_6_, 151 MHz) δ 72.3 (C-7), 105.8 (C-2′), 107.6 (C-5′), 112.8 (C-8), 114.7 (C-2), 115 (C-5), 116.6 (C-8′), 118.2 (C-6), 124.2 (C-6′), 128.1 (C-1′), 129.8 (C-1), 143.9 (C-7′), 144.8 (C-4), 148.5 (C-3′), 149.6 (C-4′), 167.8 (C-9′). ESI(-)-HRMS *m/z* 329.0671 [M – H]^–^, (calcd for C_17_H_13_O_7_, 329.0661, Δ = 3 ppm). MS/MS spectrum: CCMSLIB00006717997.

7,8-*trans*-8-8-γ-Lactone-diferulic acid (7) (Kim et al., 2005). ^1^H NMR (CD_3_OD, 600 MHz) δ 3.82 (3H, s, 3OCH_3_), 3.88 (3H, s, 3′OCH_3_), 4.16 (1H, t, *J* = 2.9, 2.3 Hz, H-8), 5.71 (1H, d, *J* = 2.9 Hz, H-7), 6.77 (1H, dd, *J* = 8.1, 2 Hz, H-6), 6.8 (1H, d, *J* = 8.1 Hz, H-5), 6.85 (1H, d, *J* = 8.2 Hz, H-5′), 6.89 (1H, d, *J* = 2 Hz, H-2), 7.14 (1H, dd, *J* = 8.2, 2.1 Hz, H-6′), 7.29 (1H, d, *J* = 2.1 Hz, H-2′), 7.65 (1H, d, *J* = 2.3 Hz, H-7′); ^13^C NMR (CD_3_OD, 151 MHz) δ 55.3 (C-8), 56.4 (3O CH_3_), 56.5 (3′O CH_3_), 83 (C-7), 110.2 (C-2), 114 (C-2′), 116.5 (C-5), 116.6 (C-5′), 119.3 (C-6), 120.6 (C-8′), 126.8 (1′), 127.2 (C-6′), 132.6 (C-1), 141.8 (C-7′), 148.3 (C-4), 149.3 (C-3), 149.4 (C-3′), 150.9 (C-4′), 174.1 (C-9). ESI(-)-HRMS *m/z* 385.0932 [M – H]^–^, (calcd for C_20_H_17_O_8_, 385.0923, Δ = 2.3 ppm). MS/MS spectrum: CCMSLIB00006718011.

7,8-*cis*-8-8-γ-Lactone-diferulic acid (8).^1^H NMR (CD_3_OD, 600 MHz) δ 3.87 (3H, s, 3OCH_3_), 3.89 (3H, s, 3′OCH_3_), 4.51 (1H, d, *J* = 7.6 Hz, H-8), 5.72 (1H, d, *J* = 7.6 Hz, H-7), 6.79 (1H, d, *J* = 8.1 Hz, H-5), 6.86 (1H, d, *J* = 8.2 Hz, H-5′), 6.91 (1H, dd, *J* = 8.1, 2 Hz, H-6), 7.06 (1H, d, *J* = 2 Hz, H-2), 7.12 (1H, dd, *J* = 8.2, 2 Hz, H-6′), 7.25 (1H, d, *J* = 2 Hz, H-2′), 7.57 (1H, d, *J* = 1.8 Hz, H-7′); ^13^C NMR (CD_3_OD, 151 MHz) δ 54.2 (C-8), 55.9 (3OCH_3_), 56.3 (3′OCH_3_), 81.6 (C-7), 110.8 (C-2), 113.4 (C-2′), 115.5 (C-5), 116.2 (C-5′), 120.3 (C-6), 123.0 (C-8′), 126.8 (C-6′), 127.1 (C-1′), 128.1 (C-1), 139.6 (C-7′), 147.7 (C-4), 148.5 (C-3), 149 (C-3′), 150.4 (C-4′), 173.3 (C-9), 174.2 (C-9′). ESI(-)-HRMS *m/z* 385.0932 [M – H]^–^, (calcd for C_20_H_17_O_8_, 385.0923, Δ = 2.3 ppm). MS/MS spectrum: CCMSLIB00006718012.

Dehydrodiferulic acid dilactone (9) (Ou et al., 2003; Hirata et al., 2005).^1^H NMR (DMSO-*d*_6_, 600 MHz) δ 3.8 (6H, s, 3′OCH_3_, 3OCH_3_), 4.2 (2H, t, *J* = 1.4 Hz, H-8, H-8′), 5.73 (2H, d, *J* = 1.5 Hz, H-7, H-7′), 6.8 (2H, d, *J* = 8.1 Hz, H-5, H-5′), 6.87 (2H, dd, *J* = 8.1, 2.1 Hz, H-6, H-6′), 7 (2H, d, *J* = 2.1 Hz, H-2, H-2′), 9.26 (2H, s, 4′OH, 4OH); ^13^C NMR (DMSO-*d*_6_, 151 MHz) δ 48.1 (C-8, C-8′), 55.8 (3′OCH_3_, 3OCH_3_), 82.1 (C-7, C-7′), 110.6 (C-2, C-2′), 115.4 (C-5, C-5′), 119.2 (C-6, C-6′), 129 (C-1, C-1′), 147.4 (C-4, C-4′), 147.9 (C-3, C-3′), 175.4 (C-9, C-9′). ESI(-)-HRMS *m/z* 385.0933 [M – H]^–^, (calcd for C_20_H_17_O_8_, 385.0923, Δ = 2.6 ppm). MS/MS spectrum: CCMSLIB00006717999.

8-5′-Benzofuran-diferulic acid (10) (Ralph et al., 1994; Ward et al., 2001). ^1^H NMR (CD_3_OD, 600 MHz) δ 3.83 (3H, s, 3OCH_3_), 3.91 (3H, s, 3′OCH_3_), 4.31 (1H, d, *J* = 7.6 Hz, H-8), 6.02 (1H, d, *J* = 7.6 Hz, H-7), 6.36 (1H, d, *J* = 15.8 Hz, H-8′), 6.8 (1H, d, *J* = 8.1 Hz, H-5), 6.85 (2H, dd, *J* = 8.1, 2 Hz, H-6), 6.96 (1H, d, *J* = 2 Hz, H-2), 7.2 (1H, d, *J* = 1.5 Hz, H-2′), 7.27 (1H, d, *J* = 1.5 Hz, H-6′), 7.63 (1H, d, *J* = 15.8 Hz, H-7′); ^13^C NMR (CD_3_OD, 151 MHz) δ 56.4 (3OCH_3_), 56.8 (3′OCH_3_), 56.9 (C-8), 89.1 (C-7), 110.6 (C-2), 113.9 (C-2′), 116.3 (C-5), 116.9 (C-8′), 119.2 (C-6′), 119.9 (C-6), 128.2 (C-5′), 130 (C-1′), 132.9 (C-1), 146.1 (C-3′), 146.5 (C-7′), 148.1 (C-4), 149.3 (C-3), 151.4 (C-4′), 170.8 (C-9′), 174.0 (C-9). ESI(-)-HRMS *m/z* 385.0933 [M – H]^–^, (calcd for C_20_H_17_O_8_, 385.0923, Δ = 2.6 ppm). MS/MS spectrum: CCMSLIB00006717995.

(2E,2′E)-3,3′-[[2-hydroxy-2-(4-hydroxy-3-methoxyphenyl) ethylidene]bis[oxy(3-methoxy-4,1-phenylene)]]bis-2-Propenoic acid (11) (Ward et al., 2001). UV (MeOH) λmax (log ε) 233 (sh) (4.46), 287 (4.45), 319 (4.36) nm. ^1^H NMR (CD_3_OD, 600 MHz) δ 3.68 (3H, s, 3′OCH_3_), 3.78 (3H, s, 3′′OCH_3_), 3.84 (3H, s, 3OCH_3_), 4.95 (1H, d, *J* = 5.5 Hz, H-7), 6 (1H, d, *J* = 5.5 Hz, H-8), 6.33 (1H, d, *J* = 15.9 Hz, H-8′), 6.37 (1H, d, *J* = 15.9 Hz, H-8′′), 6.74 (1H, d, *J* = 8.3 Hz, H-5′), 6.77 (1H, d, *J* = 8.2 Hz, H-5), 6.94 (1H, d, *J* = 8.3 Hz, H-5′′), 6.96 (2H, m, H-6′, H-6), 7.03 (1H, dd, *J* = 8.3, 2 Hz, H-6′′), 7.08 (1H, d, *J* = 2 Hz, H-2′), 7.14 (1H, d, *J* = 1.9 Hz, H-2), 7.16 (1H, d, *J* = 2 Hz, H-2′′), 7.52 (1H, d, *J* = 15.9 Hz, H-7′), 7.55 (1H, d, *J* = 15.9 Hz, H-7′′); ^13^C NMR (CD_3_OD, 151 MHz) δ 56.4 (3OCH_3_, 3′OCH_3_), 56.5 (3′′OCH_3_), 75.8 (C-7), 106.3 (C-8), 112.6 (C-2′′, C-2′), 112.6 (C-2), 115.7 (C-5), 118.5 (C-8′), 118.6 (C-8′′), 120.2 (C-5′), 120.8 (C-5′′), 121.7 (C-6), 122.6 (C-6′), 122.7 (C-6′′), 131.6 (C-1′), 131.8 (C-1′′), 132.2 (C-1), 145.6 (C-7′′, C-7′), 147.4 (C-4), 148.7 (C-3), 149.1 (C-4′), 149.3 (C-4′′), 152.1 (C-3′), 152.3 (C-3′′), 170.8 (C-9′′, C-9′). ESI(-)-HRMS *m/z* 551.1558 [M – H]^–^, (calcd for C_29_H_27_O_11_, 551.1558, Δ = 0.9 ppm). MS/MS spectrum: CCMSLIB00006717991.

(2E)-*rel*-3-[(1R,3aR,4R,9bS)-1,3a,4,9b-tetrahydro-1,4-bis(4-h ydroxy-3-methoxyphenyl)-6-methoxy-3-oxo-3H-furo[3,4-c][1] benzopyran-8-yl]-2-Propenoic acid (12) (Liu et al., 2005). UV (MeCN) λmax (log ε) 237 (sh) (4.42), 289 (4.26), 324 (4.22) nm. ^1^H NMR (CD_3_OD, 600 MHz) δ 3.5 (1H, t, *J* = 9.2, 7.5 Hz, H-8′′), 3.83 (3H, s, 3′′OCH_3_), 3.85 (3H, s, 3′OCH_3_), 3.87 (1H, t, *J* = 8, 7.5 Hz, H-8), 3.89 (3H, s, 3OCH_3_), 5.23 (1H, d, *J* = 9.2 Hz, H-7′′), 5.42 (1H, d, *J* = 8 Hz, H-7), 6.18 (1H, d, *J* = 15.9 Hz, H-8′), 6.51 (1H, d, *J* = 2.2 Hz, H-6′), 6.8 (1H, d, *J* = 8.2 Hz, H-5′′), 6.9 (1H, dd, *J* = 8.2, 2.1 Hz, H-6′′), 6.92 (2H, m, H-5, H-6), 7.07 (1H, d, *J* = 2.1 Hz, H-2′′), 7.09 (1H, s, H-2), 7.11 (1H, d, *J* = 2.2 Hz, H-2′), 7.4 (1H, d, *J* = 15.9 Hz, H-7′); ^13^C NMR (CD_3_OD, 151 MHz) δ 44.7 (C-8), 46.2 (C-8′′), 75.8 (C-7′′), 88.5 (C-7), 110.6 (C-2′), 111.7 (C-2), 112.3 (C-2′′), 116 (C-5′′), 116.4 (C-5), 118 (C-8′), 121.6 (C-6), 121.6 (C-6′′), 122.2 (C-5′), 123.1 (C-6′), 129.1 (C-1′), 129.9 (C-1), 130.1 (C-1′′), 145.7 (C-7′), 147.5 (C-4′), 148.2 (C-4′′), 148.9 (C-4), 149 (C-3′′), 149.6 (C-3), 150.7 (C-3′), 170.8 (C-9′), 176 (C-9′′). ESI(-)-HRMS *m/z* 533.1455 [M – H]^–^, (calcd for C_29_H_25_O_10_, 533.1448, Δ = 1.4 ppm). MS/MS spectrum: CCMSLIB00006717994.

*meso*-8-5′-Benzofuran tetra-ferulic acid (13). UV (MeOH) λmax (log ε) 234 (sh) (4.53), 290 (4.39), 324 (4.44) nm. ^1^H NMR (CD_3_OD, 600 MHz) δ 3.78 (6H, s, 3OCH_3_), 3.91 (6H, s, 3′OCH_3_), 4.06 (2H, d, *J* = 5.6 Hz, H-8), 5.45 (2H, d, *J* = 5.6 Hz, H-7), 6.2 (2H, d, *J* = 15.9 Hz, H-8′), 6.68 (2H, d, *J* = 1.6 Hz, H-6′), 6.78 (2H, dd, *J* = 8.1, 1.8 Hz, H-6), 6.8 (2H, d, *J* = 8.1 Hz, H-5), 6.82 (2H, d, *J* = 1.8 Hz, H-2), 7.2 (2H, d, *J* = 1.6 Hz, H-2′), 7.54 (2H, d, *J* = 15.9 Hz, H-7′); ^13^C NMR (CD_3_OD, 151 MHz) δ 54.4 (C-8), 56.4 (3OCH_3_), 56.8 (3′OCH_3_), 89.5 (C-7), 110.9 (C-2), 114.1 (C-2′), 116.5 (C-5), 117.2 (C-8′), 118.7 (C-6′), 120.3 (C-6), 130.2 (C-1′), 130.4 (C-5′), 133.3 (C-1), 146.1 (C-3′), 146.3 (C-7′), 148.3 (C-4), 149.3 (C-3), 152.3 (C-4′), 171 (C-9′). ESI(-)-HRMS *m/z* 681.2004 [M – H]^–^, (calcd for C_38_H_33_O_12_, 681.1972, Δ = 4.7 ppm). MS/MS spectrum: CCMSLIB00006717998.

*threo*-8-5′-Benzofuran tetra-ferulic acid (14). UV (MeOH) λmax (log ε) 232 (sh) (4.61), 289 (4.48), 323 (4.52) nm. ^1^H NMR (CD_3_OD, 600 MHz) δ 3.64 (6H, s, 3OCH_3_), 3.98 (6H, s, 3′OCH_3_), 4.07 (2H, d, *J* = 4 Hz, H-8), 5.44 (2H, d, *J* = 4 Hz, H-7), 6.23 (2H, d, *J* = 2 Hz, H-2), 6.33 (2H, d, *J* = 15.8 Hz, H-8′), 6.42 (2H, dd, *J* = 8.1, 2 Hz, H-6), 6.63 (2H, d, *J* = 8.1 Hz, H-5), 7.12 (2H, d, *J* = 1.5 Hz, H-6′), 7.23 (2H, d, *J* = 1.5 Hz, H-2′), 7.56 (2H, d, *J* = 15.8 Hz, H-7′); ^13^C NMR (CD_3_OD, 151 MHz) δ 56.2 (C-8), 56.3 (3OCH_3_), 56.9 (3′OCH_3_), 88.3 (C-7), 109.6 (C-2), 113.8 (C-2′), 116.1 (C-5), 117.9 (C-8′), 118.5 (C-6), 119.4 (C-6′), 130.2 (C-5′), 130.5 (C-1′), 134.3 (C-1), 145.8 (C-7′), 146.2 (C-3′), 147.5 (C-4), 149 (C-3), 152.3 (C-4′), 171.3 (C-9′). ESI(-)-HRMS *m/z* 681.2005 [M – H]^–^, (calcd for C_38_H_33_O_12_, 681.1972, Δ = 4.8 ppm). MS/MS spectrum: CCMSLIB00006717993.

*rel*-(2E)-3-[4-[[5-(2-Carboxyethenyl)-2,3-dihydro-2-(4-hydr oxy-3-methoxyphenyl)-7-methoxy-3-benzofuranyl]oxy]-3-meth oxyphenyl]-2-propenoic acid (15) (Ward et al., 2001). UV (MeOH) λmax (log ε) 231 (sh) (4.52), 292 (4.48), 322 (4.49) nm. ^1^H NMR (CD_3_OD, 600 MHz) δ 3.74 (3H, s, 3OCH_3_), 3.86 (3H, s, 3′′OCH_3_), 3.95 (3H, s, 3′OCH_3_), 5.76 (1H, d, *J* = 2.7 Hz, H-7), 5.91 (1H, d, *J* = 2.7 Hz, H-8), 6.34 (1H, d, *J* = 15.9 Hz, H-8′), 6.43 (1H, d, *J* = 15.9 Hz, H-8′′), 6.7 (1H, dd, *J* = 8.1, 2.0 Hz, H-6), 6.75 (1H, d, *J* = 2 Hz, H-2), 6.76 (1H, d, *J* = 8.1 Hz, H-5), 7.02 (1H, d, *J* = 8.2 Hz, H-5′′), 7.16 (1H, dd, *J* = 8.2, 2 Hz, H-6′′), 7.2 (1H, d, *J* = 1.9 Hz, H-6′), 7.29 (1H, d, *J* = 1.9 Hz, H-2′), 7.3 (1H, d, *J* = 2 Hz, H-2′′), 7.61 (1H, d, *J* = 15.9 Hz, H-7′), 7.63 (1H, d, *J* = 15.9 Hz, H-7′′); ^13^C NMR (CD_3_OD, 151 MHz) δ 56.4 (3OCH_3_), 56.5 (3′′OCH_3_), 56.9 (3′OCH_3_), 87.9 (C-8), 92 (C-7), 110.4 (C-2), 112.7 (C-2′′), 114.8 (C-2′), 116.5 (C-5), 117.5 (C-8′), 118.6 (C-8′′), 119.6 (C-6), 120.2 (C-5′′), 120.6 (C-6′), 123.0 (C-6′′), 128 (C-5′), 130.3 (C-1′), 131.1 (C-1), 131.6 (C-1′′), 145.7 (C-7′′), 146.1 (C-7′), 146.5 (C-3′), 148.1 (C-4), 149.3 (C-3), 149.6 (C-4′′), 152.6 (C-3′′), 153.2 (C-4′), 170.8 (C-9′′), 170.9 (C-9′). ESI(-)-HRMS *m/z* 533.1455 [M – H]^–^, (calcd for C_29_H_25_O_10_, 533.1448, Δ = 1.3 ppm). MS/MS spectrum: CCMSLIB00006717992.

*rel*-(E)-3-(4,5-Dihydroxy-2-((2S,3R,4R)-4-((R)-hydroxy(4-hy droxy-3-methoxyphenyl)methyl)-2-(4-hydroxy-3-methoxyphen yl)-5-oxotetrahydrofuran-3-yl)phenyl)acrylic acid (16). UV (MeOH) λmax (log ε) 232 (sh) (4.37), 284 (4.15), 328 (3.98) nm. ^1^H NMR (CD_3_OD, 600 MHz) δ 3.67 (1H, dd, *J* = 11.3, 5.4 Hz, H-8′′), 3.71 (3H, s, 3OCH_3_), 3.73 (3H, s, 3′′OCH_3_), 3.78 (1H, t, *J* = 11.3, 9.5 Hz, H-8), 5.01 (1H, d, *J* = 9.5 Hz, H-7), 5.14 (1H, d, *J* = 5.4 Hz, H-7′′), 5.71 (1H, dd, *J* = 10.5, 5.4 Hz, H-8′), 6.35 (1H, dd, *J* = 8, 2 Hz, H-6), 6.47 (1H, d, *J* = 2 Hz, H-2), 6.57 (1H, d, *J* = 8 Hz, H-5), 6.64 (1H, d, *J* = 8 Hz, H-5′′), 6.73 (1H, dd, *J* = 8, 1.9 Hz, H-6′′), 6.79 (1H, s, H-2′), 6.82 (1H, d, *J* = 1.9 Hz, H-2′′), 6.97 (1H, s, H-5′), 7.19 (1H, d, *J* = 15.5 Hz, H-7′); ^13^C NMR (CD_3_OD, 151 MHz) δ 48.3 (C-8), 56.1 (3′′OCH_3_), 56.4 (3OCH_3_), 56.4 (C-8′′), 74.3 (C-7′′), 88.9 (C-7), 109.9 (C-2), 111.3 (C-2′′), 113.7 (C-2′), 114.8 (C-5′), 115.6 (C-5), 115.8 (C-5′′), 117.8 (C-8′), 120.7 (C-6′′), 121.2 (C-6), 127.2 (C-1′), 129.3 (C-1), 130.9 (C-6′), 133.3 (C-1′′), 143.2 (C-7′), 145.8 (C-3′), 147.2 (C-4′′), 148.3 (C-4), 148.6 (C-3′′), 149.4 (C-4′), 149.4 (C-3), 170.7 (C-9′), 178.4 (C-9′′). ESI(-)-HRMS *m/z* 537.1401 [M – H]^–^, (calcd for C_28_H_25_O_11_, 537.1397, Δ = 0.7 ppm). MS/MS spectrum: CCMSLIB00006718002.

2′-*O*-Methylspirocaffeic acid trimer (17). UV (MeOH) λmax (log ε) 237 (sh) (4.72), 267 (4.76), 318 (4.48), 356 (4.35) nm. ^1^H NMR (DMSO-*d*_6_, 600 MHz) δ 3.65 (3H, s, 3′OCH_3_), 3.96 (1H, dd, *J* = 8.9, 3.3 Hz, H-8′), 4.97 (1H, d, *J* = 8.9 Hz, H-7′), 5.71 (1H, d, *J* = 16 Hz, H-8′′), 6.05 (1H, s, H-5′′), 6.21 (1H, s, H-5), 6.53 (1H, dd, *J* = 8, 2 Hz, H-6′), 6.63 (1H, d, *J* = 8 Hz, H-5′), 6.71 (1H, s, H-2′′), 6.9 (1H, d, *J* = 2 Hz, H-2′), 6.93 (1H, d, *J* = 16 Hz, H-7′′), 7.03 (1H, s, H-2), 7.53 (1H, d, *J* = 3.3 Hz, H-7), 9.11 (1H, s, 4′OH), 9.17 (1H, s, 4′′OH), 9.36 (1H, s, 3OH), 9.67 (1H, s, 4OH), 12.57 (1H, s, COOH); ^13^C NMR (DMSO-*d*_6_, 151 MHz) δ 55.2 (3′OCH_3_), 48.8 (C-6′′), 48.5 (C-8′), 81.6 (C-7′), 111.1 (C-2′), 112.9 (C-5), 114.3 (C-5′), 117.8 (C-2), 118.3 (C-5′′), 120.8 (C-6′), 123.6 (C-1), 125.6 (C-8′′), 126.8 (C-6), 127.4 (C-1′), 130.8 (C-2′′), 131 (C-7), 138.8 (C-7′′), 145 (C-3), 147 (C-4′), 147.2 (C-3′, C-4′′), 148.6 (C-4), 154.6 (C-1′′), 165.9 (C-9′′), 167.2 (C-9), 180.7 (C-3′′). ESI(-)-HRMS *m/z* 503.0981 [M – H]^–^, (calcd for C_27_H_19_O_10_, 503.0978, Δ = 0.6 ppm). MS/MS spectrum: CCMSLIB00006718001.

5-[(E)-2-carboxyvinyl]-7-hydroxy-2-(4-hydroxy-3-methoxyp henyl)-2,3-dihydro-1-benzofuran-3-carboxylic acid (8-5′-ben zofuran-caffeic-ferulic acid dimer) (18). ^1^H NMR (DMSO-*d*_6_, 600 MHz) δ 3.75 (3H, s, 3O CH_3_), 4.17 (1H, brs, H-8), 5.92 (1H, d, *J* = 7.8 Hz, H-7), 6.18 (1H, d, *J* = 15.9 Hz, H-8′), 6.76 (1H, d, *J* = 8.1 Hz, H-5), 6.79 (1H, dd, *J* = 8.1, 2.0 Hz, H-6), 6.96 (1H, d, *J* = 2 Hz, H-2), 6.97 (1H, d, *J* = 2.2 Hz, H-2′), 7.11 (1H, d, *J* = 2.2 Hz, H-6′), 7.44 (1H, d, *J* = 15.9 Hz, H-7′).

(E)-3-(3,4-dihydroxy-5-((E)-4-hydroxy-3-methoxystyryl)phe nyl)acrylic acid (19). UV (MeOH) λmax (log ε) 240 (sh) (4.1), 294 (4.12), 321 (4.17) nm. ^1^H NMR (DMSO-*d*_6_, 600 MHz) δ 3.83 (3H, s, 3OCH_3_), 6.25 (1H, d, *J* = 15.9 Hz, H-8′), 6.77 (1H, d, *J* = 8.2 Hz, H-5), 6.92 (1H, d, *J* = 2 Hz, H-2′), 6.96 (1H, dd, *J* = 8.2, 2 Hz, H-6), 7.12 (1H, d, *J* = 2 Hz, H-2), 7.19 (2H, s, H-7, H-8), 7.39 (1H, d, *J* = 2 Hz, H-6′), 7.44 (1H, d, *J* = 15.9 Hz, H-7′); ^13^C NMR (DMSO-*d*_6_, 151 MHz) δ 55.5 (3OCH_3_), 109.7 (C-2), 112.4 (C-2′), 115.6 (C-5, C-8′), 118 (C-6′), 119.8 (C-6), 119.9 (C-8), 124.9 (C-1′), 125 (C-5′), 129 (C-7), 129.2 (C-1), 144.6 (C-7′), 145.7 (C-4′), 146.5 (C-4), 147.8 (C-3), 168 (C-9′). ESI(-)-HRMS *m/z* 327.0872 [M – H]^–^, (calcd for C_18_H_15_O_6_, 327.0869, Δ = 1.1 ppm). MS/MS spectrum: CCMSLIB00006718006.

Dehydrocaffeicferulic acid dilactone (20). ^1^H NMR (DMSO-*d*_6_, 600 MHz) δ 3.8 (3H, s, 3′OCH_3_), 4.08 (1H, dd, *J* = 9.8, 3 Hz, H-8′), 4.15 (1H, dd, *J* = 9.8, 2.9 Hz, H-8), 5.67 (1H, d, *J* = 2.9 Hz, H-7), 5.72 (1H, d, *J* = 3 Hz, H-7′), 6.71 (1H, dd, *J* = 8.1, 2.2 Hz, H-6), 6.77 (1H, d, *J* = 8.1 Hz, H-5), 6.79 (1H, d, *J* = 8.2 Hz, H-5′), 6.81 (1H, d, *J* = 2.2 Hz, H-2), 6.86 (1H, dd, *J* = 8.2, 2.1 Hz, H-6′), 6.99 (1H, d, *J* = 2.2 Hz, H-2′); ^13^C NMR (DMSO-*d*_6_, 151 MHz) δ 48.1 (C-8′), 48.2 (C-8), 55.8 (3′OCH_3_), 81.9 (C-7), 82.1 (C-7′), 110.6 (C-2′), 113.7 (C-2), 115.4 (C-5′), 115.7 (C-5), 117.3 (C-6), 119.3 (C-6′), 129 (C-1′), 129.1 (C-1), 145.5 (C-3), 146.1 (C-4), 147.4 (C-4′), 147.9 (C-3′), 175.3 (C-9), 175.4 (C-9′). ESI(-)-HRMS *m/z* 371.0769 [M – H]^–^, (calcd for C_19_H_15_O_8_, 371.0767, Δ = 0.5 ppm). MS/MS spectrum: CCMSLIB00006718007.

*rel*-(E)-3-(4,5-dihydroxy-2-((2S,3S)-4-((E)-4-hydroxy-3-met hoxybenzylidene)-3-(4-hydroxy-3-methoxyphenyl)-5-oxotetrah ydrofuran-2-yl)phenyl)acrylic acid (21). UV (MeOH) λmax (log ε) 237 (sh) (4.31), 288 (4.16), 328 (4.12) nm. ^1^H NMR (DMSO-*d*_6_, 600 MHz) δ 3.51 (3H, s, 3′′O CH_3_), 3.68 (3H, s, 3OCH_3_), 4.38 (1H, t, *J* = 2.6, 2.1 Hz, H-7), 5.61 (1H, d, *J* = 2.6 Hz, H-8), 6.15 (1H, d, *J* = 15.4 Hz, H-8′), 6.57 (1H, dd, *J* = 8.1, 2.1 Hz, H-6), 6.64 (1H, s, H-5′), 6.71 (1H, d, *J* = 8.1 Hz, H-5), 6.71 (1H, d, *J* = 8.3 Hz, H-5′′), 6.85 (2H, m, H-2, H-2′′), 7.01 (1H, dd, *J* = 8.3, 2 Hz, H-6′′), 7.11 (1H, s, H-2′), 7.62 (1H, d, *J* = 15.4 Hz, H-7′), 7.65 (1H, d, *J* = 2.1 Hz, H-7′′); ^13^C NMR (DMSO-*d*_6_, 151 MHz) δ 51.7 (C-7), 55.2 (3′′OCH_3_), 55.4 (3OCH_3_), 82.3 (C-8), 111.5 (C-2), 112 (C-5′), 113.6 (C-2, C-2′′), 115.5 (C-5′′), 116.3 (C-5), 118.1 (C-8′), 118.8 (C-6), 122.9 (C-1′), 124.5 (C-1′′), 126.1 (C-6′′), 131 (C-1), 131.2 (C-6′), 139.2 (C-7′′), 139.9 (C-7′), 145.6 (C-3′), 145.8 (C-4), 147.4 (C-3′′), 147.7 (C-3), 148 (C-4′), 149.2 (C-4′′), 167.4 (C-9′), 172.3 (C-9′′). ESI(-)-HRMS *m/z* 519.1294 [M – H]^–^, (calcd for C_28_H_23_O_10_, 519.1291, Δ = 0.6 ppm). MS/MS spectrum: CCMSLIB00006718003.

*rel*-(E)-3-((1S,3aS,4S,9bR)-1-(3,4-dihydroxyphenyl)-4-(4-hyd roxy-3-methoxyphenyl)-6-methoxy-3-oxo-1,3a,4,9b-tetrahydro-3H-furo[3,4-c]chromen-8-yl)acrylic acid (22). UV (MeOH) λmax (log ε) 236 (sh) (4.29), 288 (4.15), 322 (4.05) nm. ^1^H NMR (DMSO-*d*_6_, 600 MHz) δ 3.56 (1H, dd, *J* = 9.1, 7.6 Hz, H-8′′), 3.75 (3H, s, 3′′OCH_3_), 3.78 (3H, s, 3′OCH_3_), 3.81 (1H, t, *J* = 7.9, 7.6 Hz, H-8), 5.29 (1H, d, *J* = 9.1 Hz, H-7′′), 5.44 (1H, d, *J* = 7.9 Hz, H-7), 6.26 (1H, d, *J* = 15.9 Hz, H-8′), 6.45 (1H, d, *J* = 1.9 Hz, H-6′), 6.76 (1H, d, *J* = 8.1 Hz, H-5′′), 6.78 (1H, dd, *J* = 8.1, 2.2 Hz, H-6), 6.84 (1H, d, *J* = 8.1 Hz, H-5), 6.85 (1H, dd, *J* = 8.1, 2 Hz, H-6′′), 6.91 (1H, d, *J* = 2.2 Hz, H-2), 7.04 (1H, d, *J* = 2 Hz, H-2′′), 7.24 (1H, d, *J* = 1.9 Hz, H-2′), 7.28 (1H, d, *J* = 15.9 Hz, H-7′); ^13^C NMR (DMSO-*d*_6_, 151 MHz) δ 42.2 (C-8), 43.7 (C-8′′), 55.6 (3′OCH_3_), 55.7 (3′′OCH_3_), 73.5 (C-7′′), 85.7 (C-7), 109.2 (C-2′), 111.8 (C-2′′), 114.6 (C-2), 115.1 (C-5′′), 115.6 (C-5), 117.4 (C-8′), 118.7 (C-6), 120.5 (C-6′′), 120.8 (C-5′), 121.7 (C-6′), 127 (C-1′), 128 (C-1), 128.3 (C-1′′), 143.7 (C-7′), 145.6 (C-4′), 146.2 (C-4), 146.9 (C-4′′), 147.4 (C-3′′), 148.8 (C-3′), 167.7 (C-9′), 173.6 (C-9′′). ESI(-)-HRMS *m/z* 519.1291 [M – H]^–^, (calcd for C_28_H_23_O_10_, 519.1291, Δ = 0 ppm). MS/MS spectrum: CCMSLIB00006718005.

*rel*-(2R,3R,4E)-2-(3,4-Dihydroxyphenyl)-4-[(3,4-dihydroxyp henyl)methylene]tetrahydro-5-oxo-3-furancarboxylic acid = phe llinsin A (23) (Hwang et al., 2000; Nemadziva et al., 2018). ^1^H NMR (DMSO-*d*_6_, 600 MHz) δ 4.41 (1H, dd, *J* = 7.6, 1.7 Hz, H-8), 5.66 (1H, d, *J* = 7.6 Hz, H-7), 6.64 (1H, dd, *J* = 8.2, 2.1 Hz, H-6), 6.7 (1H, d, *J* = 8.2 Hz, H-5), 6.79 (1H, d, *J* = 2.1 Hz, H-2), 6.81 (1H, d, *J* = 8.3 Hz, H-5′), 6.97 (1H, dd, *J* = 8.3, 2.2 Hz, H-6′), 7.03 (1H, d, *J* = 2.2 Hz, H-2′), 7.36 (1H, d, *J* = 1.7 Hz, H-7′); ^13^C NMR (DMSO-*d*_6_, 151 MHz) δ 51.5 (C-8), 79 (C-7), 113.5 (C-2), 115.1 (C-5), 115.8 (C-5′), 117.3 (C-6), 117.5 (C-2′), 121.5 (C-8′), 123.2 (C-6′), 125.1 (C-1′), 126.5 (C-1), 137.6 (C-7′), 144.9 (C-3), 145.3 (C-4), 145.6 (C-3′), 148.4 (C-4′), 170.3 (C-9), 171.2 (C-9′). ESI(-)-HRMS *m/z* 357.0617 [M – H]^–^, (calcd for C_18_H_13_O_8_, 357.061, Δ = 1.8 ppm). MS/MS spectrum: CCMSLIB00006718013.

(2E)-3-[4-Hydroxy-3-[(1E)-2-(4-hydroxy-3-methoxyphenyl) ethenyl]-5-methoxyphenyl]-2-propenoic acid = poacic acid (24) (Ralph et al., 1994). UV (MeOH) λmax (log ε) 235 (sh) (4.26), 290 (4.16), 323 (4.24) nm. ^1^H NMR (DMSO-*d*_6_, 600 MHz) δ 3.83 (3H, s, 3′OCH_3_), 3.87 (3H, s, 3OCH_3_), 6.47 (1H, d, *J* = 15.9 Hz, H-8′), 6.75 (1H, d, *J* = 8.1 Hz, H-5), 6.97 (1H, dd, *J* = 8.1, 1.9 Hz, H-6), 7.12 (1H, d, *J* = 1.9 Hz, H-2), 7.21 (3H, m, H-2′, H-7, H-8), 7.52 (1H, s, H-6′), 7.53 (1H, d, *J* = 15.8 Hz, H-7′); ^13^C NMR (DMSO-*d*_6_, 151 MHz) δ 55.5 (3OCH_3_), 56.1 (3′OCH_3_), 109 (C-2′), 109.7 (C-2), 115.7 (C-5), 116.2 (C-8′), 119.4 (C-8), 119.6 (C-6′), 119.9 (C-6), 125.4 (C-1′), 129.4 (C-7), 130.5 (C-1), 144.7 (C-7′), 145.9 (C-4′), 146.5 (C-4), 147.8 (C-3), 148 (C-3′), 167.8 (C-9). ESI(-)-HRMS *m/z* 341.1029 [M – H]^–^, (calcd for C_19_H_17_O_6_, 341.1025, Δ = 1.1 ppm). MS/MS spectrum: CCMSLIB00006718004.

### MTT Assay

Indicated cell lines were detached and resuspended at 60,000 cells/ml and added to each well of a transparent 384-well plate in the final volume of 25 μl/well. The cells were maintained in DMEM containing 10% FBS at 37°C, 5% CO_2_ overnight. The next day, the medium in each well was replaced with 50 μl of the fresh one containing the indicated concentrations of compounds. After incubation for 3 days, the medium in each well was replaced with 25 μl of.5 mg/ml thiazolyl blue solution in 1× PBS followed by incubation for 3 h at 37°C. Then, the solution was removed, and 25 μl DMSO was added to each well. Absorbance at 510 nm was measured in a Tecan Infinite M200 PRO plate reader.

### Wnt Pathway Activity Measurements

The Wnt3a-induced luciferase activity was analyzed as described (Koval et al., 2014; Shaw et al., 2019b). The BT-20 TNBC cell line stably transfected with TopFlash reporter plasmid was seeded at 150,000 cells/ml in a white opaque 384-well plate in the final volume of 25 μl of a DMEM medium supplemented with 10% FCS. The cells were maintained incubated at 37°C, 5% CO_2_ overnight for attachment. Subsequently, they were transfected by a plasmid encoding constitutively expressed Renilla luciferase under the CMV promoter as described in the protocol of the manufacturer using 12 μg/ml of DNA and 40 μl/ml XtremeGENE 9 reagent. The next day, the medium in each well was replaced with 20 μl of fresh medium containing the compound of interest; after 1 h of preincubation of the compound, Wnt3a was added to a final concentration of 2.5 μg/ml (Koval and Katanaev, 2011). After overnight incubation, the supernatant in each well was removed, and luciferase activity was measured as described (Koval et al., 2014). The culture medium was completely removed from all wells of the plate. Next, the luciferase activity of the firefly and Renilla luciferases were detected sequentially in individual wells of a 384-well plate through injection of corresponding measurement solutions in Infinite M Plex multifunctional plate reader with injection module (Dyer et al., 2000).

## Conclusion

It is well known in the literature that biotransformation reactions carried out on caffeic acid, ferulic acid, or the mixture of both with purified enzymes can generate very different compounds depending on the experimental conditions used. Indeed, conditions such as nature of the solvent or co-solvent and its proportion, concentration of the starting material, the value of pH (Carunchio et al., 2001; Adelakun et al., 2012), or presence of a surfactant (Larsen et al., 2001) allow to form some compounds more than others and thus orientate the reactions. In our case, we were able to generate most of the dimers and trimers already described except for 5-5′ dimer like diferulic acid (Larsen et al., 2001) or the series with a 1,4-benzodioxane skeleton like *trans*-3-[6-[6-[(*E*)-2-carb oxy-ethenyl]-3(3,4-dihydroxyphenyl)-2,3-dihydro-2-(1,4-ben-z odioxin)]3,4-dihy-droxyphe-nyl]-(*E*)-2-propenoic acid, the *trans* -3-[4-[[6-[(E)-2-carboxy-ethenyl]-3-(3,4-di-hydroxyphenyl)-2,3 -dihydro-2-(1,4-benzodioxin)]oxy]-3-hydroxyphenyl](E)-2-pro penoic acid obtained by Wan et al. (2008), or the (2*R*,3*S*)-6-[(1*E*)-2-Carboxyethenyl]-3-(3,4-dihydroxyphenyl)-2,3-dihydro-1,4-benzodioxin-2-carboxylic acid described by Matsumoto et al. (1999).

More surprisingly, our reactions allowed us to biosynthesize 11 new phenylpropanoid derivatives, including 4 novel scaffolds. Several hypotheses could explain this like the experimental conditions used (optimized amount of secretome, incubation time, and percentage of organic solvent) or the use of an enzymatic secretome instead of a purified enzyme. Indeed, this enzymatic pool could explain that certain reactions are not only due to laccases but other enzymes. A more detailed study of this precise point will be carried out.

Careful monitoring of the biotransformation reactions by UHPLC coupled to a triple detection system (ELSD-PDA-MS) allowed us to find the best reaction conditions. On the other hand, an innovative compound isolation process (gradient transfer from UHPLC to analytical and preparative HPLC as well as a dry load sample injection system developed in our laboratory) allowed for the isolation of a large number of compounds including the minor ones.

From a drug discovery point of view, the advantages of this approach to generate libraries of natural product derivatives seem obvious to us: the reactions can be easily carried out using a mixture of enzymes (fungal secretome) as a catalyst, which is non-toxic and environmentally friendly. Furthermore, there is no need to use a cofactor, since laccases catalyze the process using oxygen as an oxidant (Constantin et al., 2012). Finally, the reactions can be performed at room temperature in an aqueous solvent system.

Some of the generated compounds have complex structures, and some of the new structural skeletons are described here for the first time. This exemplifies the potential of this approach for the chemo-diversification of common NPs leading to the generation of libraries of unusual derivatives for biological screening. Having reproducible reactions allows a particular compound to be reproduced in case of interesting biological activities and the need for further studies.

While the starting phenylpropanoids (caffeic acid and ferulic acid) showed no specific Wnt inhibition activity, compounds 3, 6, 14, 21, 23, and 24 exhibited a moderate Wnt inhibition that has never been reported. However, the lack of long-term inhibition of TNBC cell proliferation in the compounds and the potential relationship of this to their instability suggest that this method can be considered as an excellent first step in the identification of an active pharmacophore, but that the products require further optimization to become stable, selective, and efficient.

## Data Availability Statement

The raw data supporting the conclusions of this article are available on the UNIGE website Yaretta at doi: https://doi.org/10.26037/yareta:gccdworqzfejhffuukqr4moexu. The MS/MS spectrum of each isolated compound has its own accession number from CCMSLIB00006718008 to CCMSLIB00006718013 on the Global Natural Product Social Molecular Networking (GNPS) (accessed via: https://gnps.ucsd.edu/ProteoSAFe/static/gnps-splash.jsp).

## Author Contributions

EFQ, KG, RH, SS, LM, AK, and VK contributed to the conception and design of the study. RH, LM, EM, and AK organized and performed the experiments. EFQ, KG, SS, LM, RH, and AK performed the data analysis. LM, RH, AK, VK, J-LW, KG, SS, and EFQ wrote the first draft of the article and edited the further versions. All authors read, reviewed, and approved the article.

## Conflict of Interest

The authors declare that the research was conducted in the absence of any commercial or financial relationships that could be construed as a potential conflict of interest.

## Publisher’s Note

All claims expressed in this article are solely those of the authors and do not necessarily represent those of their affiliated organizations, or those of the publisher, the editors and the reviewers. Any product that may be evaluated in this article, or claim that may be made by its manufacturer, is not guaranteed or endorsed by the publisher.
