# Supplementary material for: Chemoenzymatic Synthesis of Complex Phenylpropanoid Derivatives by the Botrytis cinerea Secretome and Evaluation of Their Wnt Inhibition Activity
Source: Front Plant Sci. 2022 Jan 13;12:805610. doi: 10.3389/fpls.2021.805610 (PMC8792767; doi:10.3389/fpls.2021.805610)
Supplement: Supplementary file 1 [file Data_Sheet_1.PDF]

frontiers

Supplementary Material

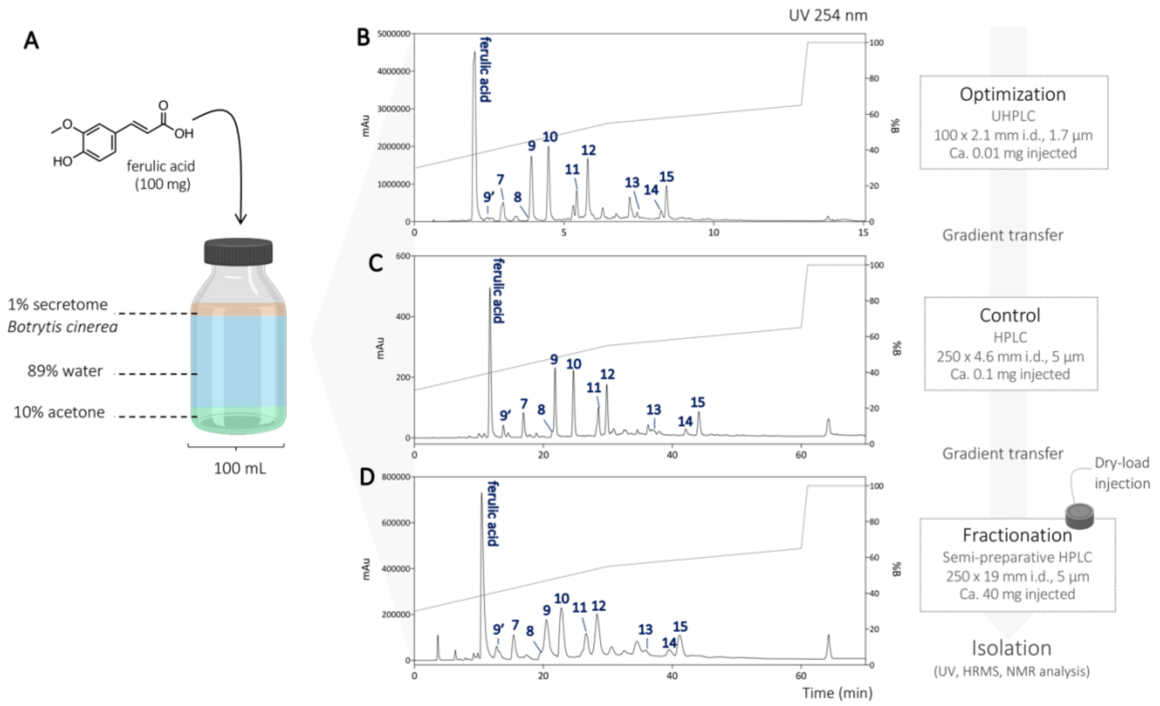

Supplementary Figure 1. (A) Scaled up biotransformation reaction of ferulic acid with the enzymatic secretome of *Botrytis cinerea* and 10% of acetone. (B) UHPLC-PDA analysis with optimized chromatographic conditions. (C) HPLC-PDA analysis using the transferred optimized chromatographic conditions. (D) Semi-preparative HPLC-UV analysis using the transferred optimized chromatographic conditions and isolation of the generated compounds.

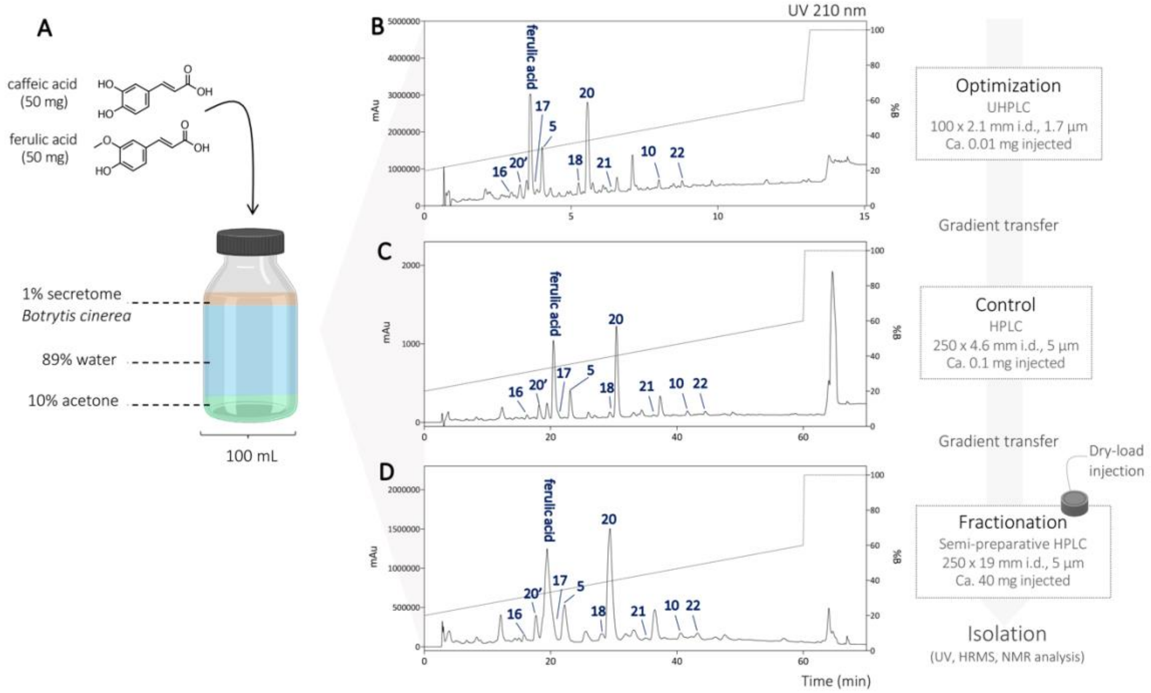

Supplementary Figure 2. (A) Scaled up biotransformation reaction of caffeic acid with the enzymatic secretome of *Botrytis cinerea* and 10% of acetone. (B) UHPLC-PDA analysis with optimized chromatographic conditions. (C) HPLC-PDA analysis using the transferred optimized chromatographic conditions. (D) Semi-preparative HPLC-UV analysis using the transferred optimized chromatographic conditions and isolation of the generated compounds.

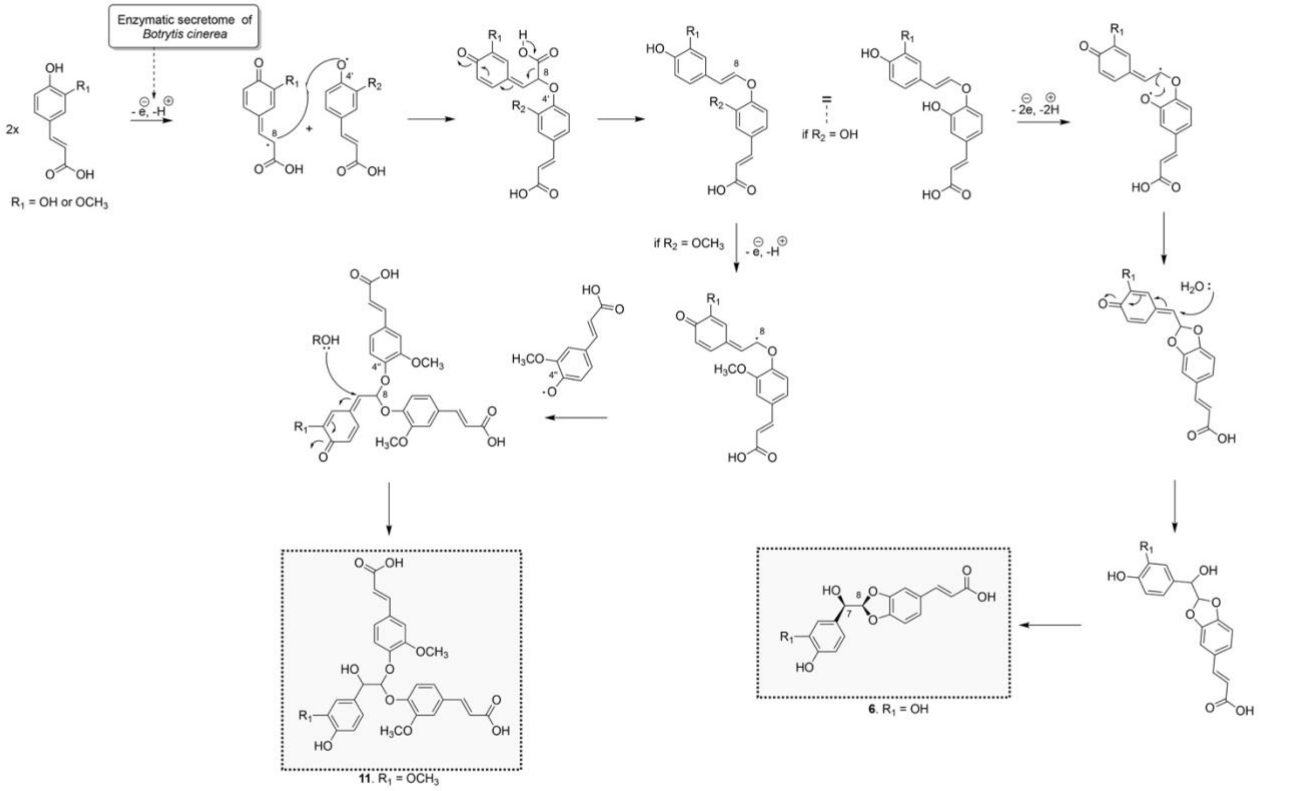

Supplementary Figure 3. Proposed chemo-enzymatic pathway for the formation of compounds **6** and **11** through 8-O-4' phenoxy radical coupling.

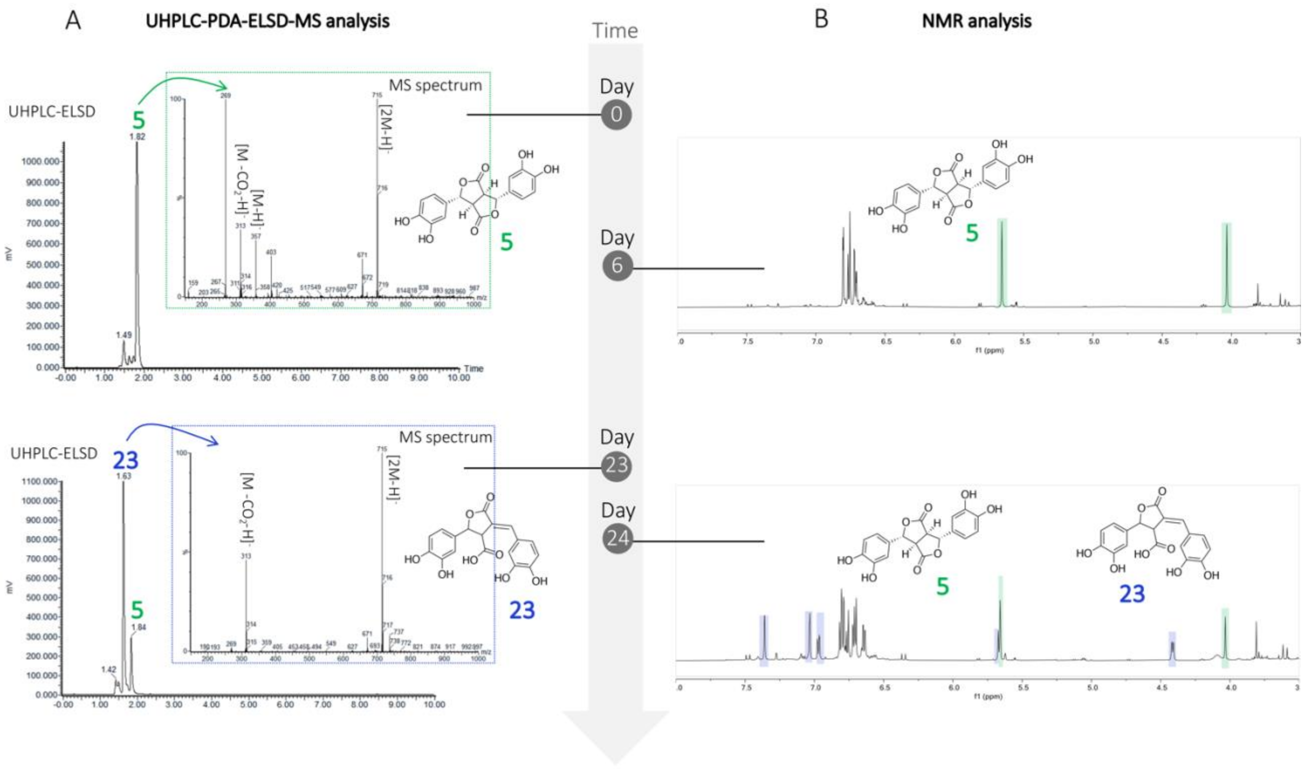

Supplementary Figure 4. (A) UHPLC-PDA-ELSD-MS and (B) NMR analysis showing the degradation of the compound **5** into **23**.

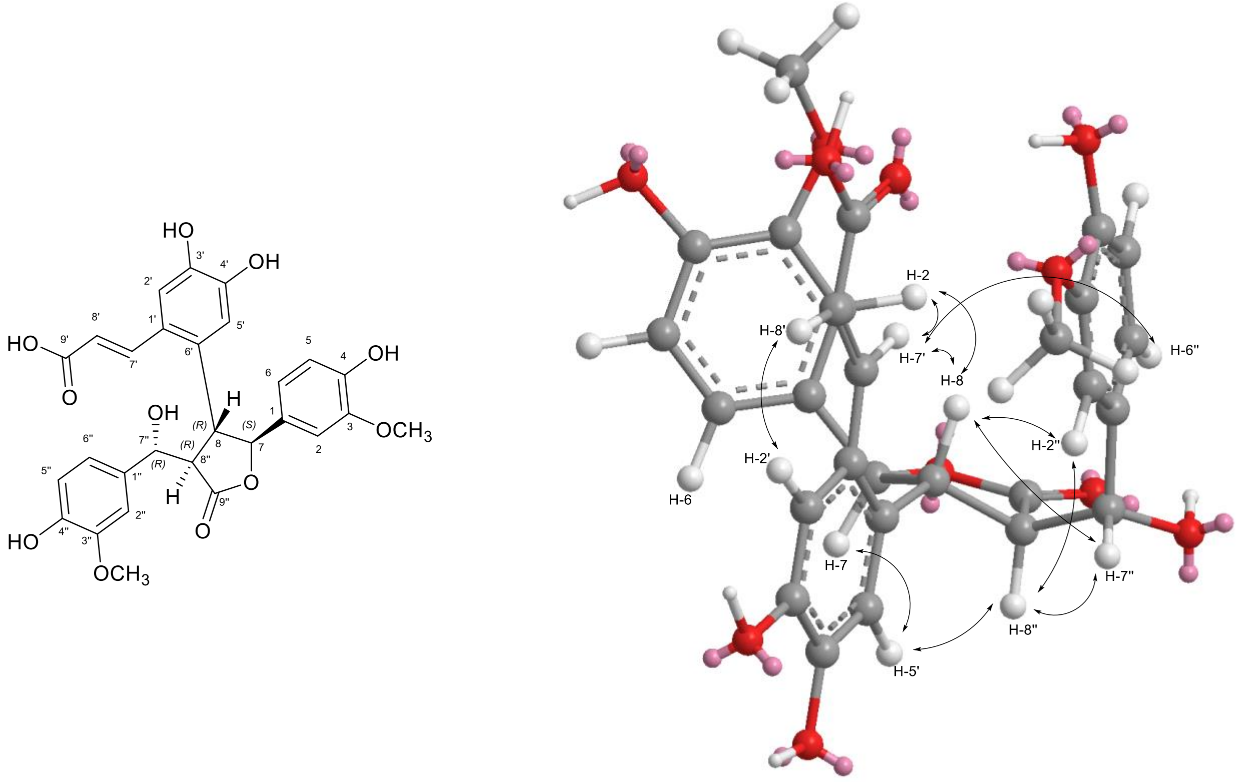

Supplementary Figure 5. Structure of compound **16** and its key ROESY correlations.

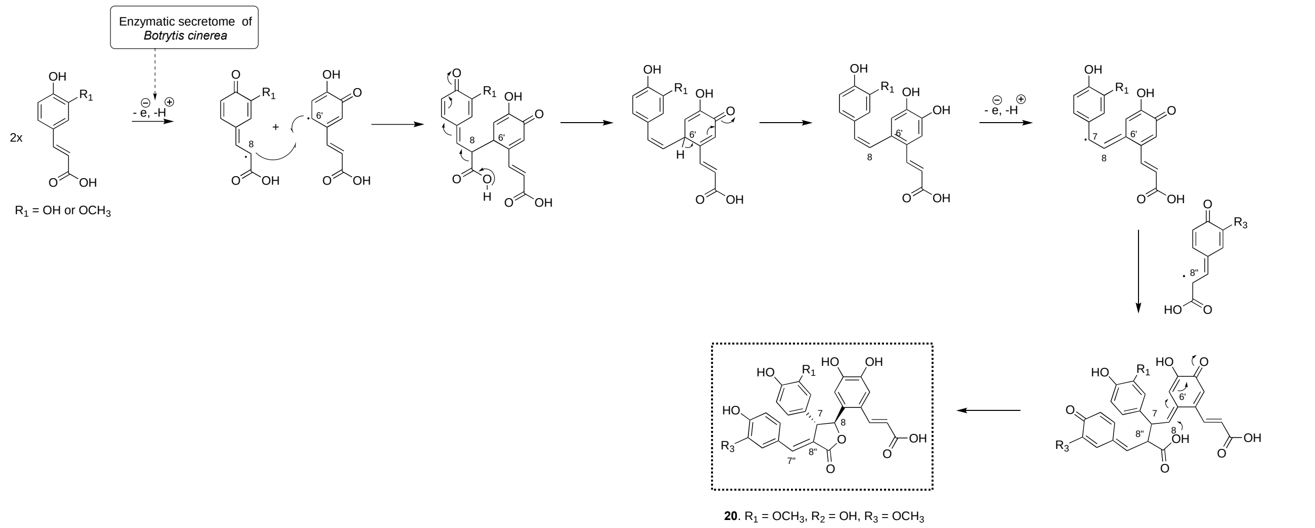

Supplementary Figure 6. Proposed chemo-enzymatic pathway for the formation of compounds **21** through 8-6' phenoxy radical coupling.

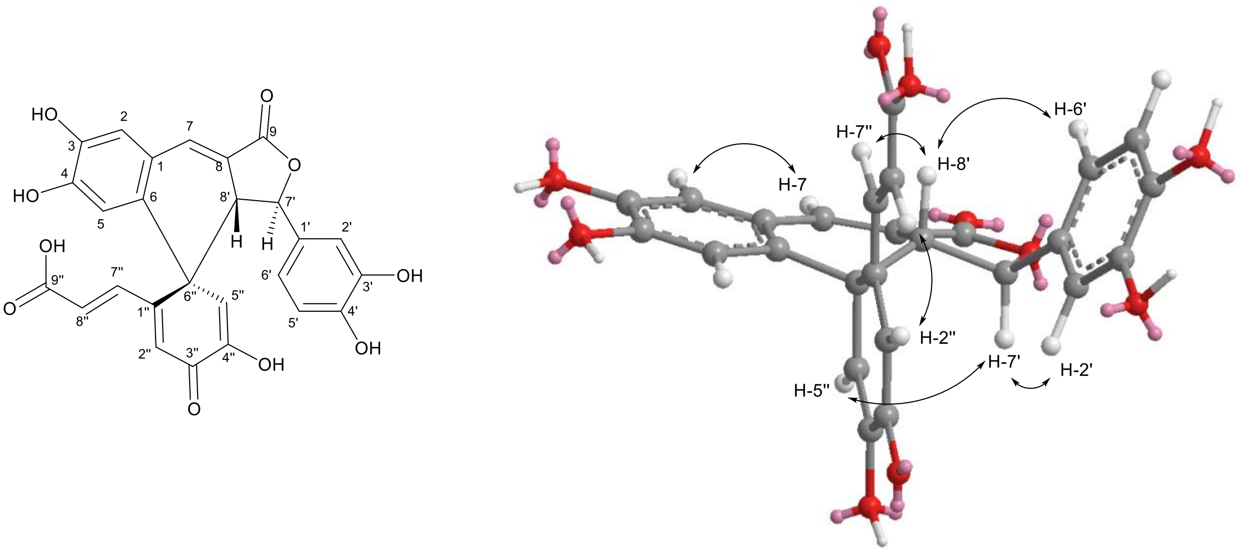

Supplementary Figure 7. Structure of compound **3** and its key ROESY correlations.

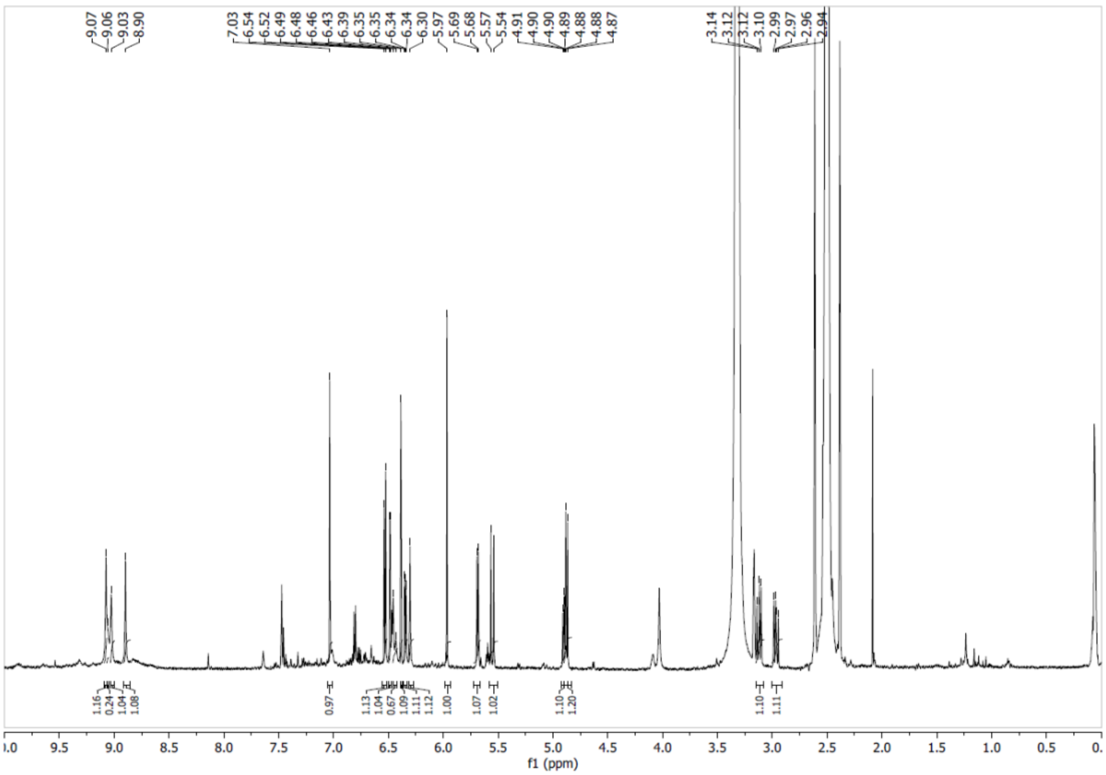

Supplementary Figure 8. ^{1}H NMR spectrum of compound **1** in DMSO-d_6 at 600 Mhz.

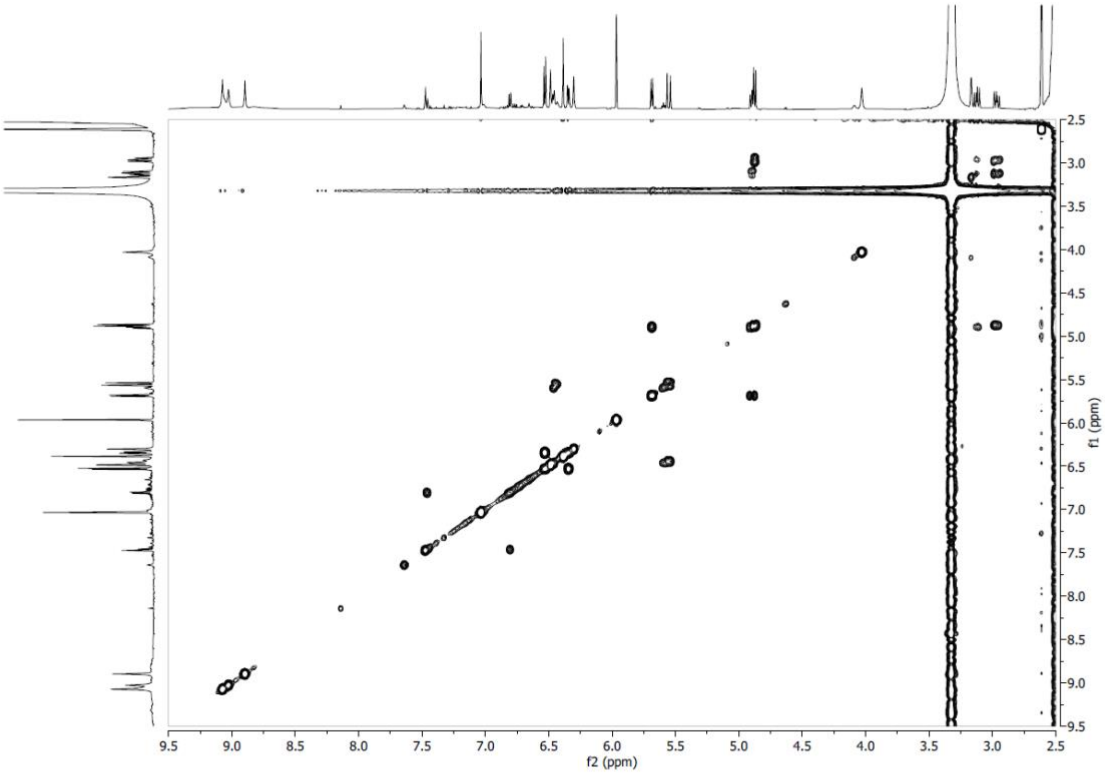

Supplementary Figure 9. COSY NMR spectrum of compound **1** in DMSO-d_6

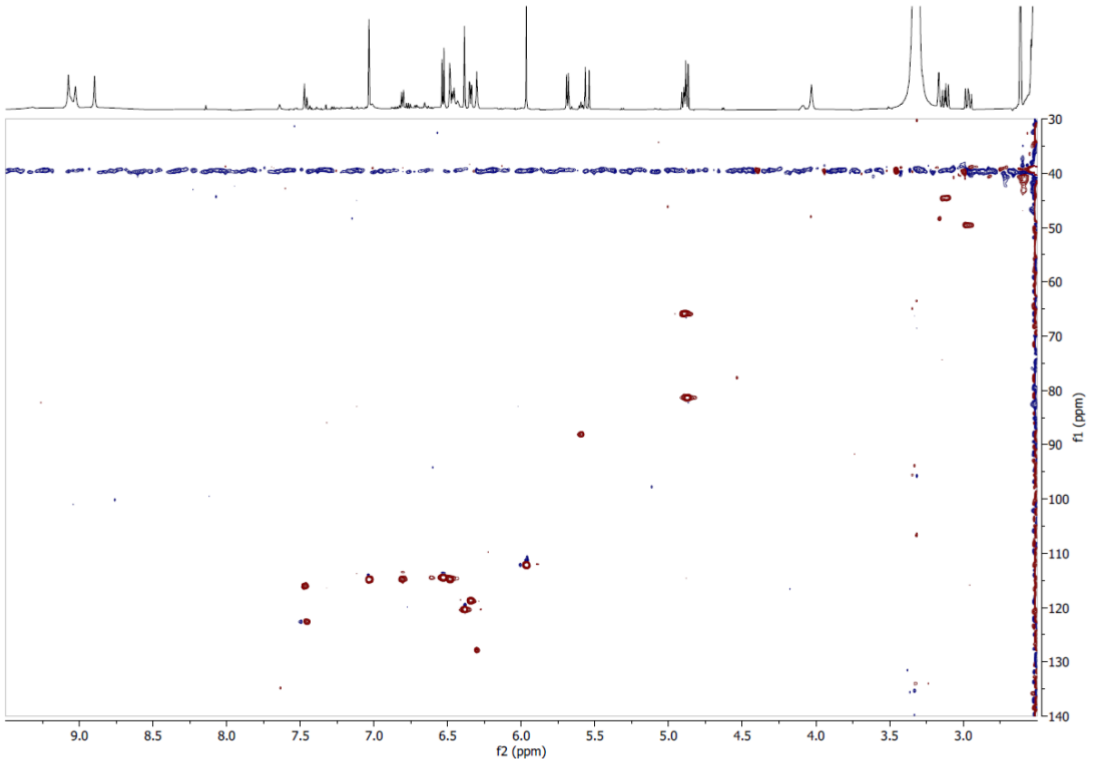

Supplementary Figure 10. Edited-HSQC NMR spectrum of compound **1** in DMSO-d_6

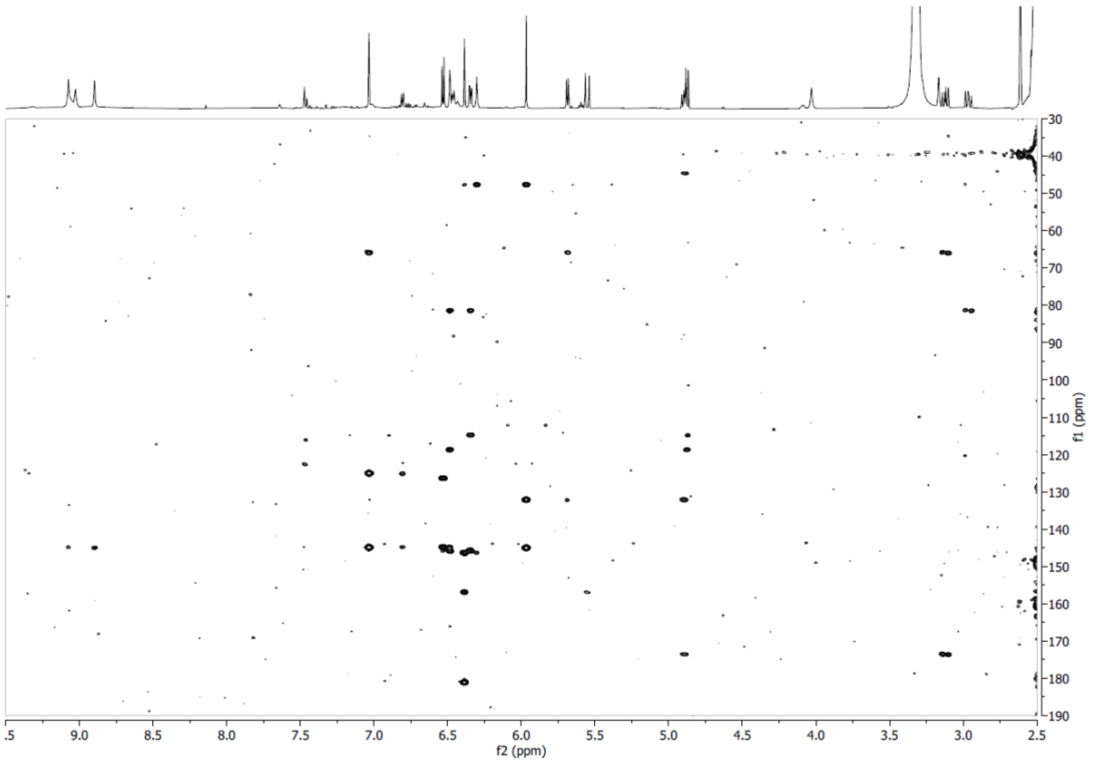

Supplementary Figure 11. HMBC NMR spectrum of compound **1** in DMSO-d_6

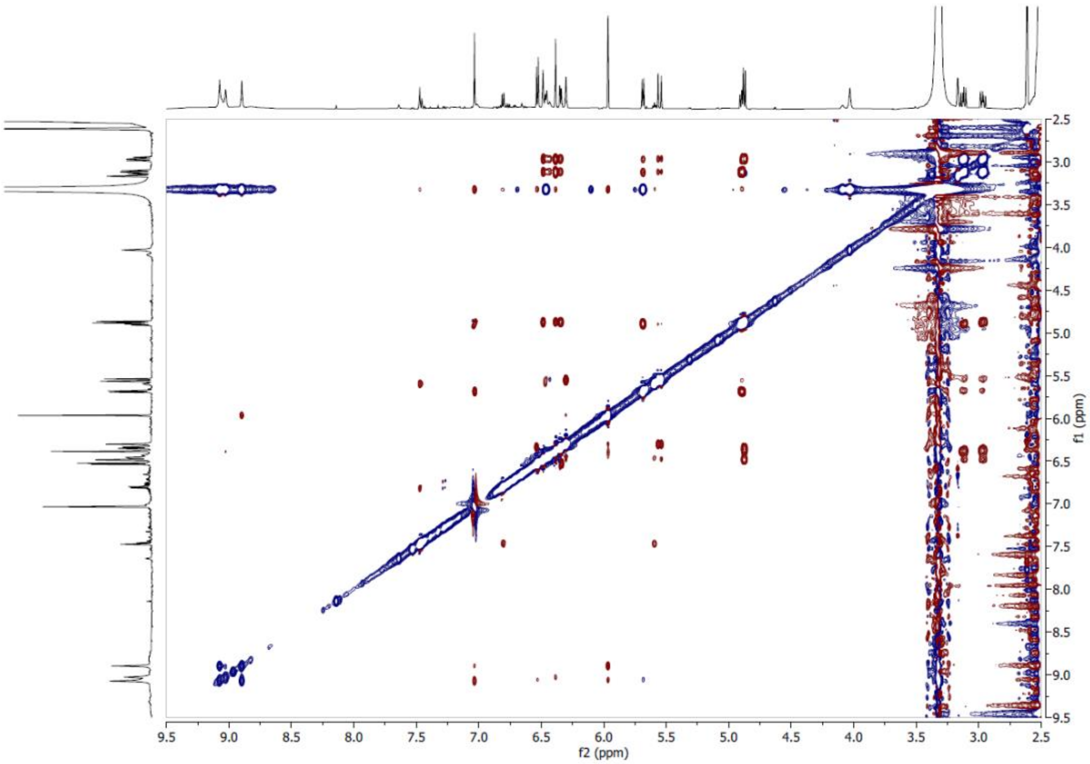

Supplementary Figure 12. ROESY NMR spectrum of compound **1** in DMSO-d_6

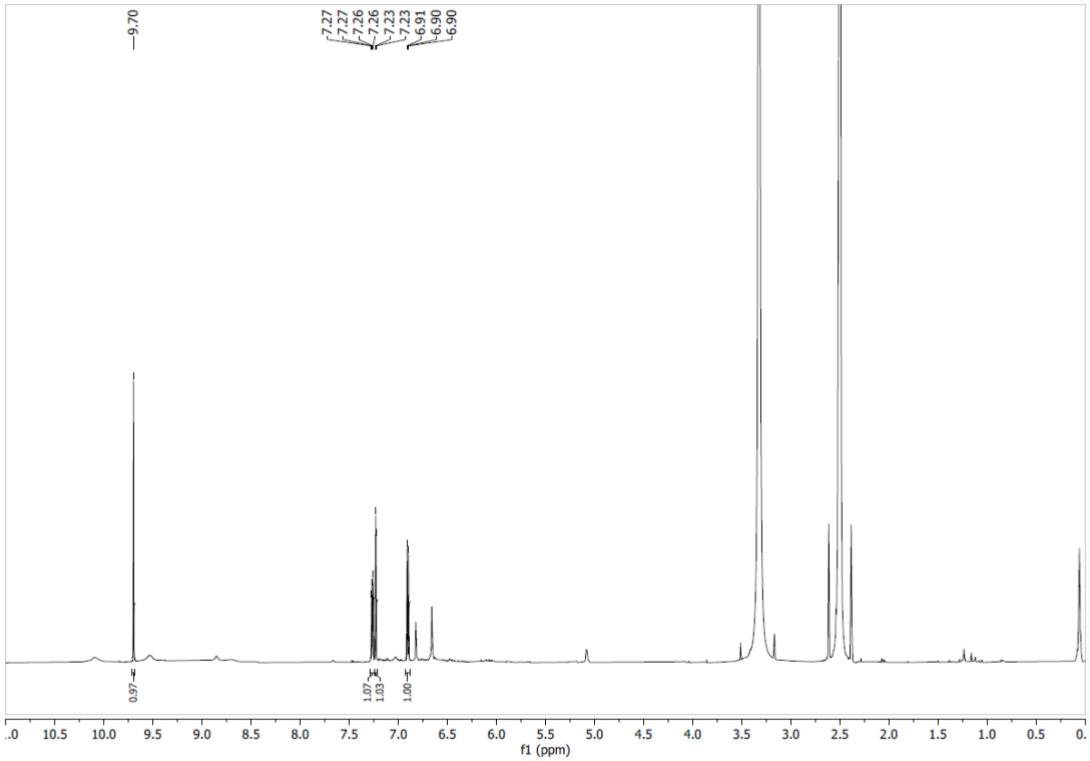

Supplementary Figure 13. ^{1}H NMR spectrum of compound **2** in DMSO-d_6 at 600 MHz

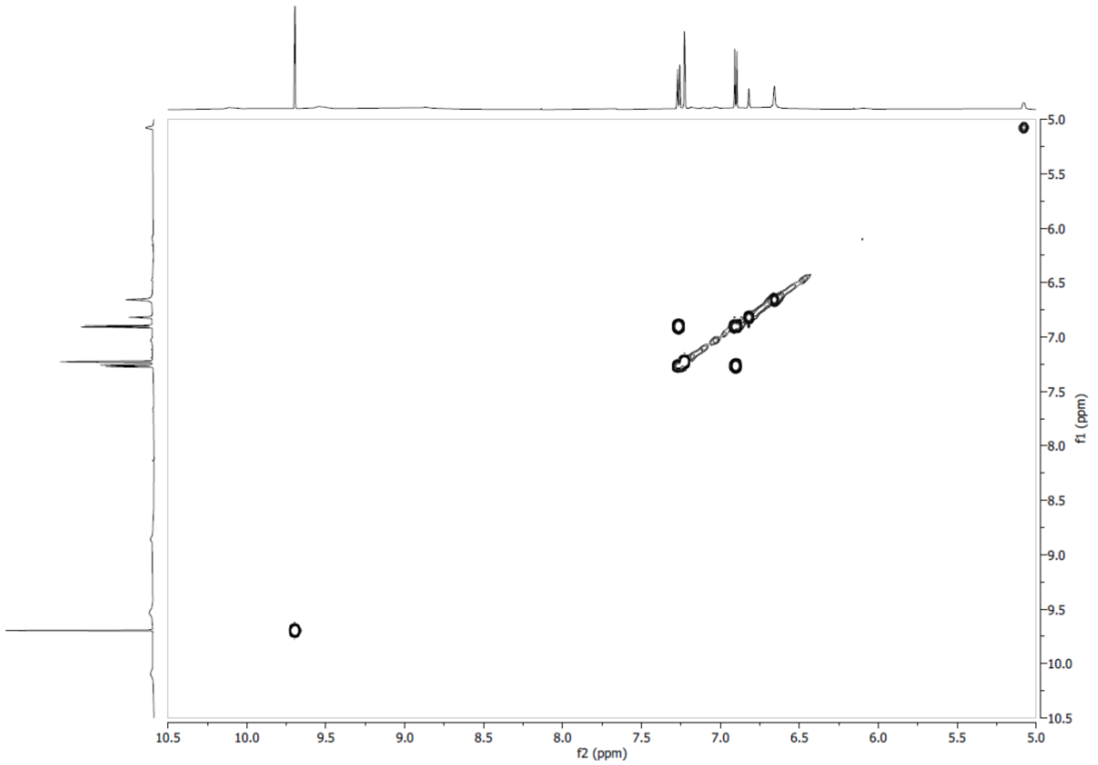

Supplementary Figure 14. COSY NMR spectrum of compound **2** in DMSO-d_6

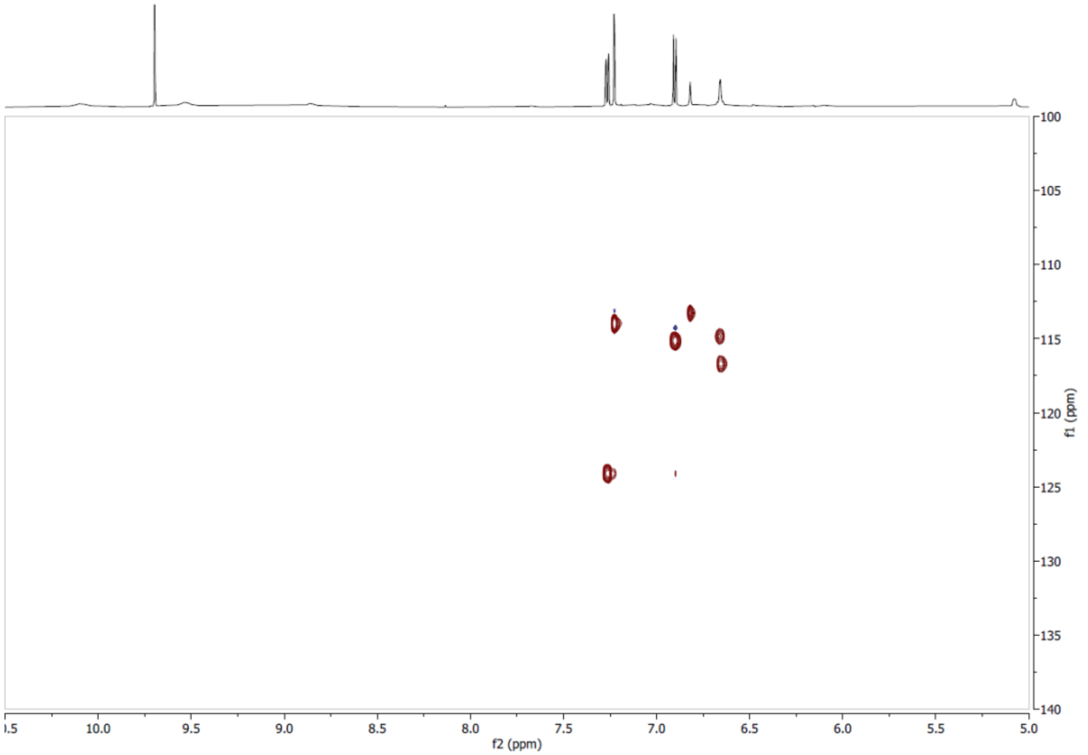

Supplementary Figure 15. Edited-HSQC NMR spectrum of compound **2** in DMSO-d_6

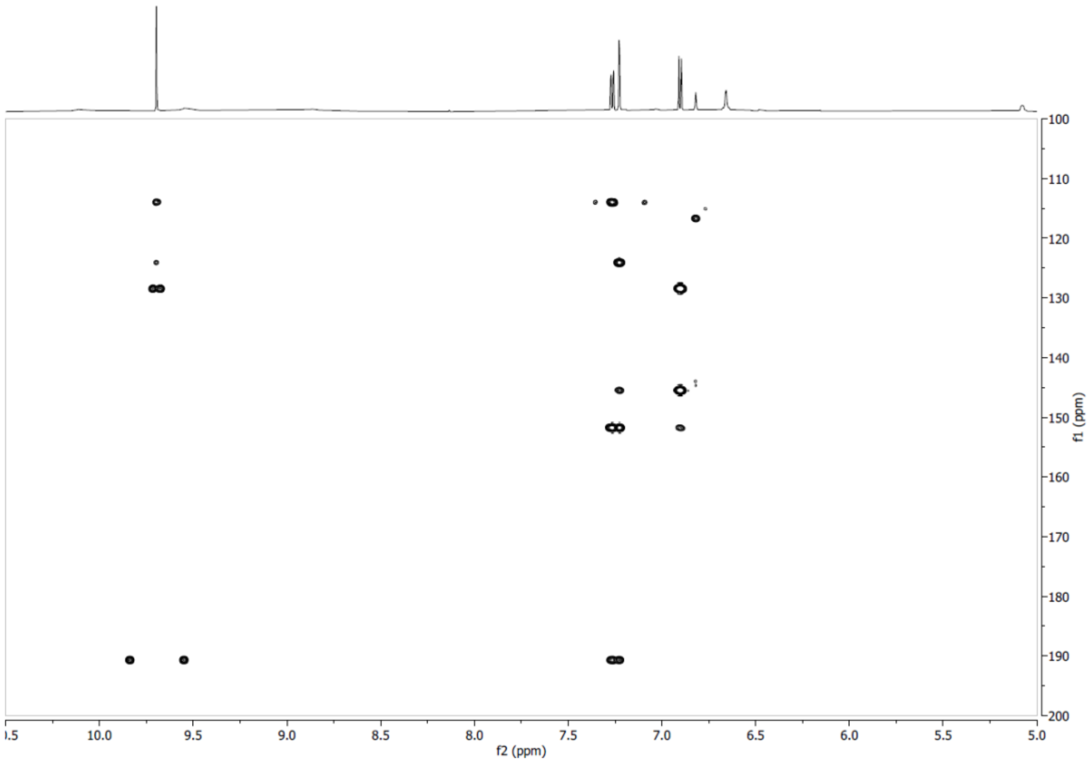

Supplementary Figure 16. HMBC NMR spectrum of compound **2** in DMSO-d_6

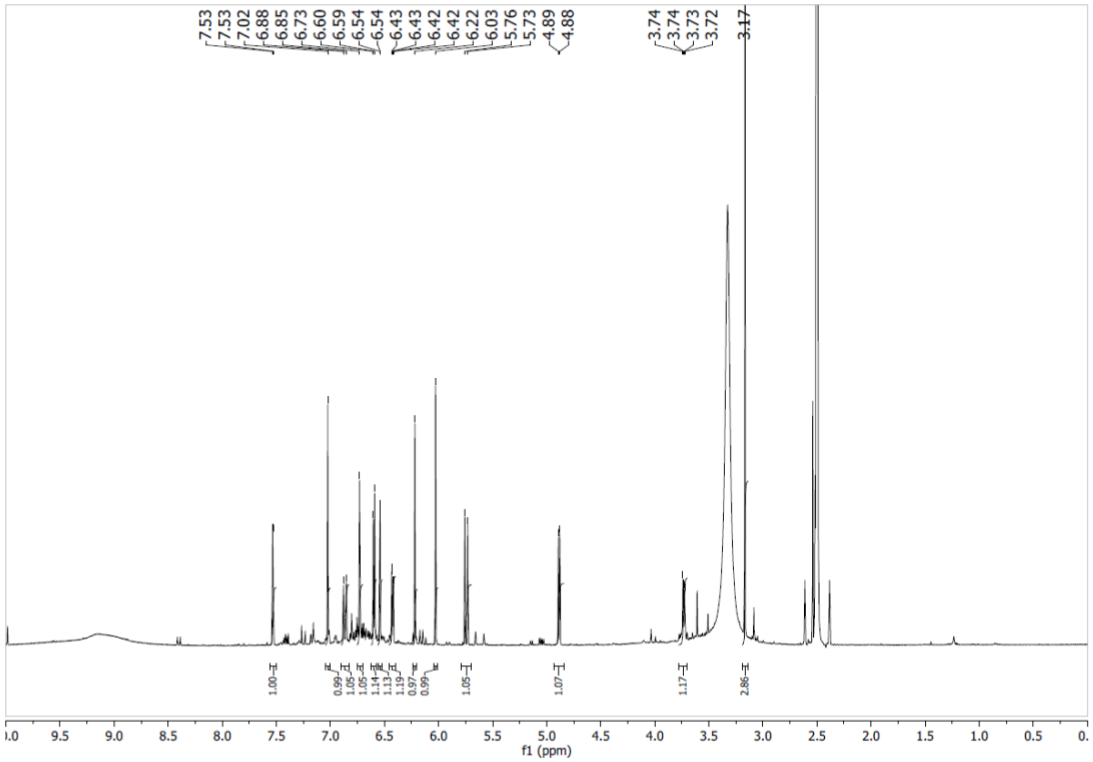

Supplementary Figure 17. ^{1}H NMR spectrum of compound **3** in DMSO-d_6 at 600 MHz

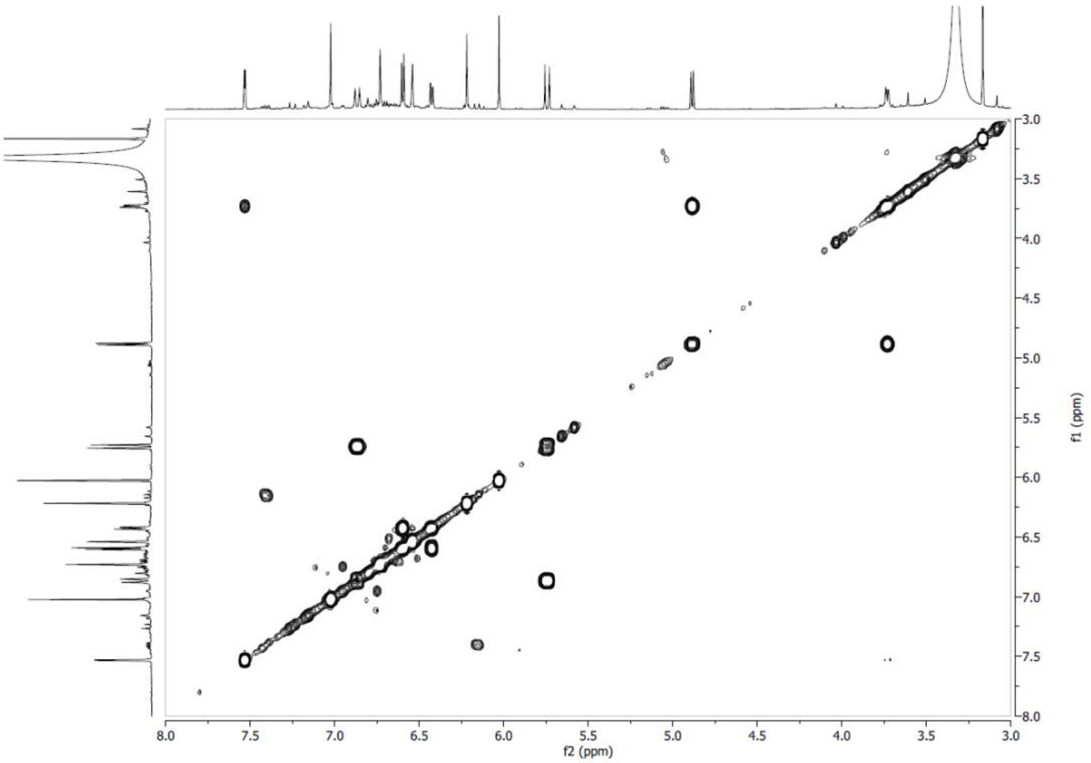

Supplementary Figure 18. COSY NMR spectrum of compound **3** in DMSO-d_6

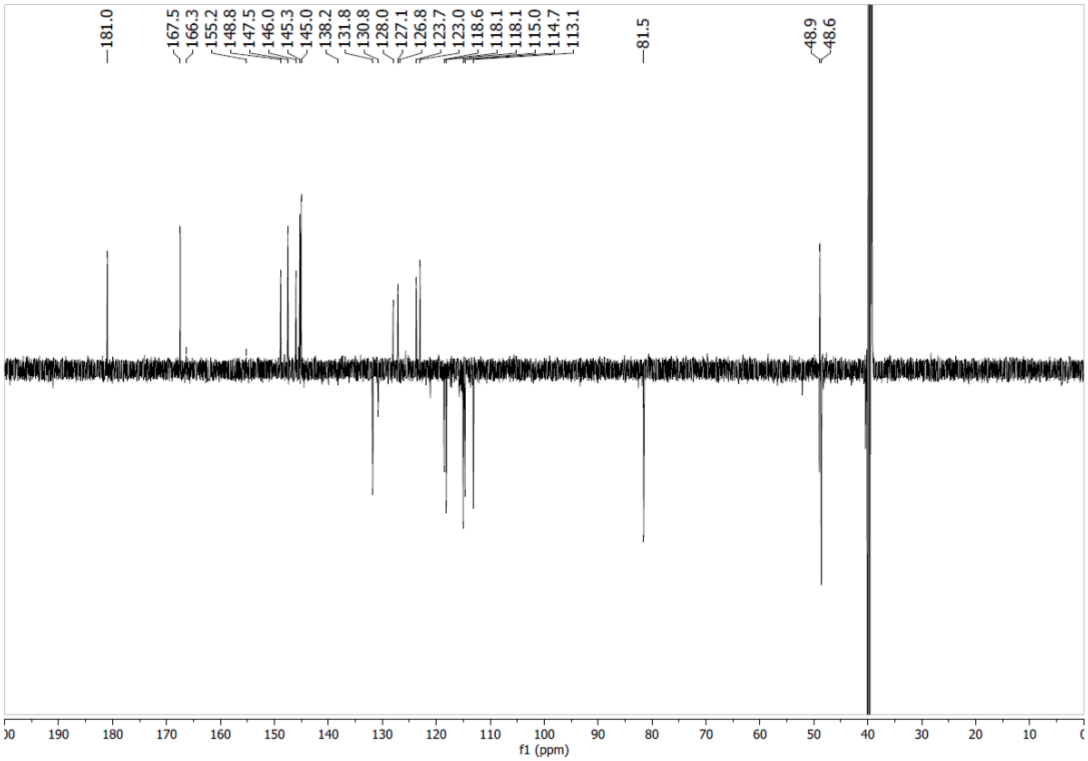

Supplementary Figure 19. ^{13}C-DEPTQ NMR spectrum of compound **3** in DMSO-d_6 at 151 MHz

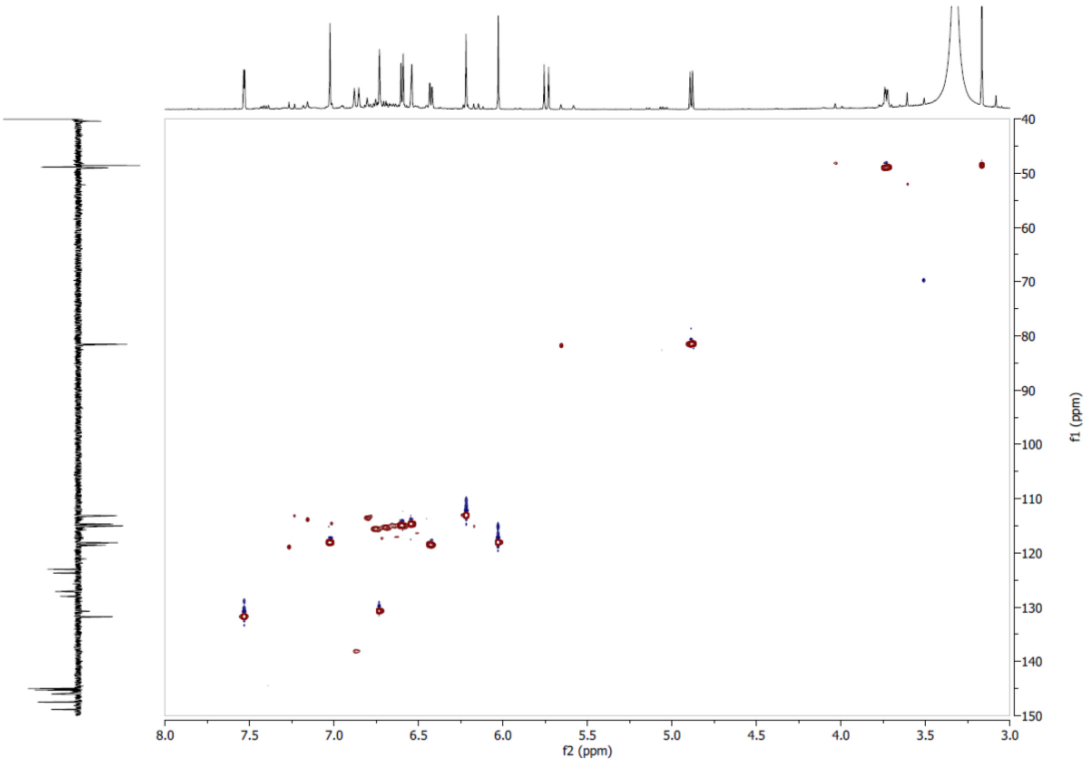

Supplementary Figure 20. Edited-HSQC NMR spectrum of compound **3** in DMSO-d_6

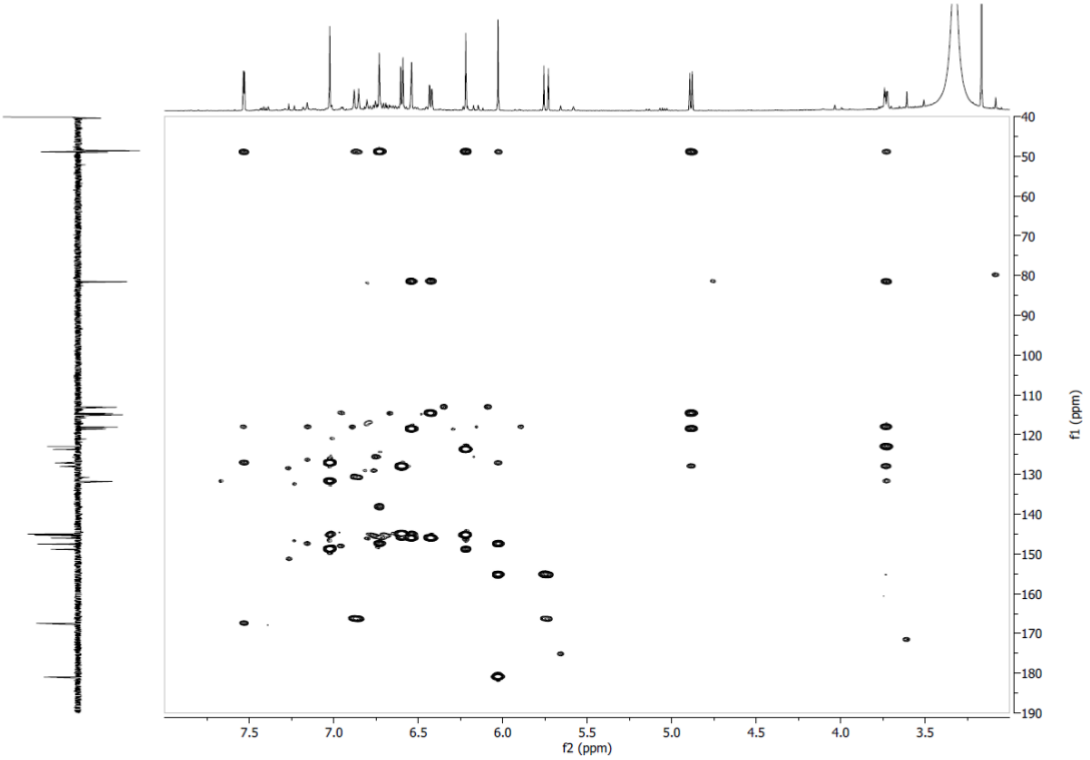

Supplementary Figure 21. HMBC NMR spectrum of compound **3** in DMSO-d_6

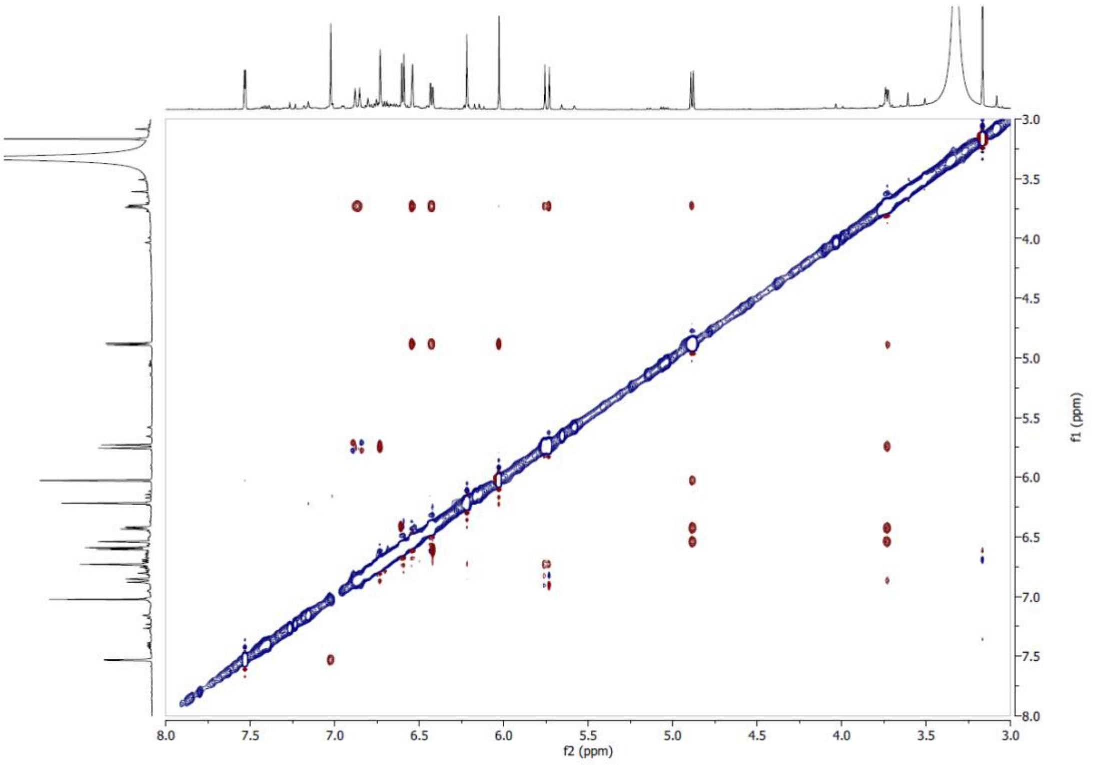

Supplementary Figure 22. ROESY NMR spectrum of compound **3** in DMSO-d_6

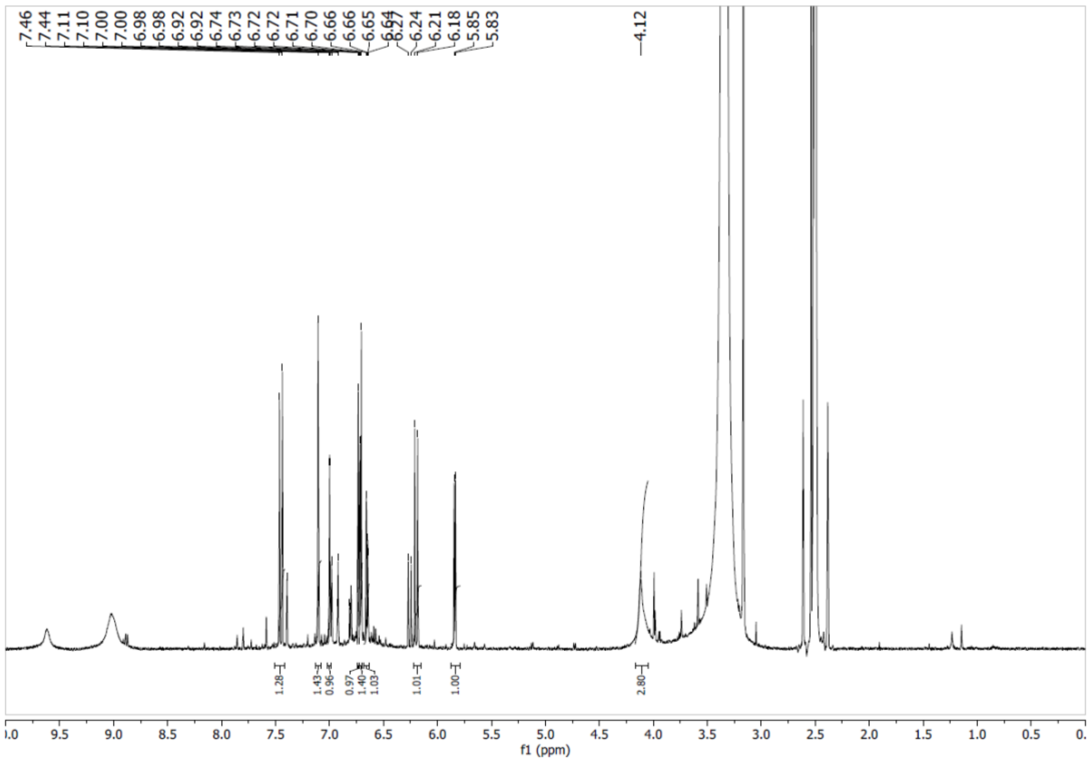

Supplementary Figure 23. ^{1}H NMR spectrum of compound **4** in DMSO-d_6 at 600 MHz

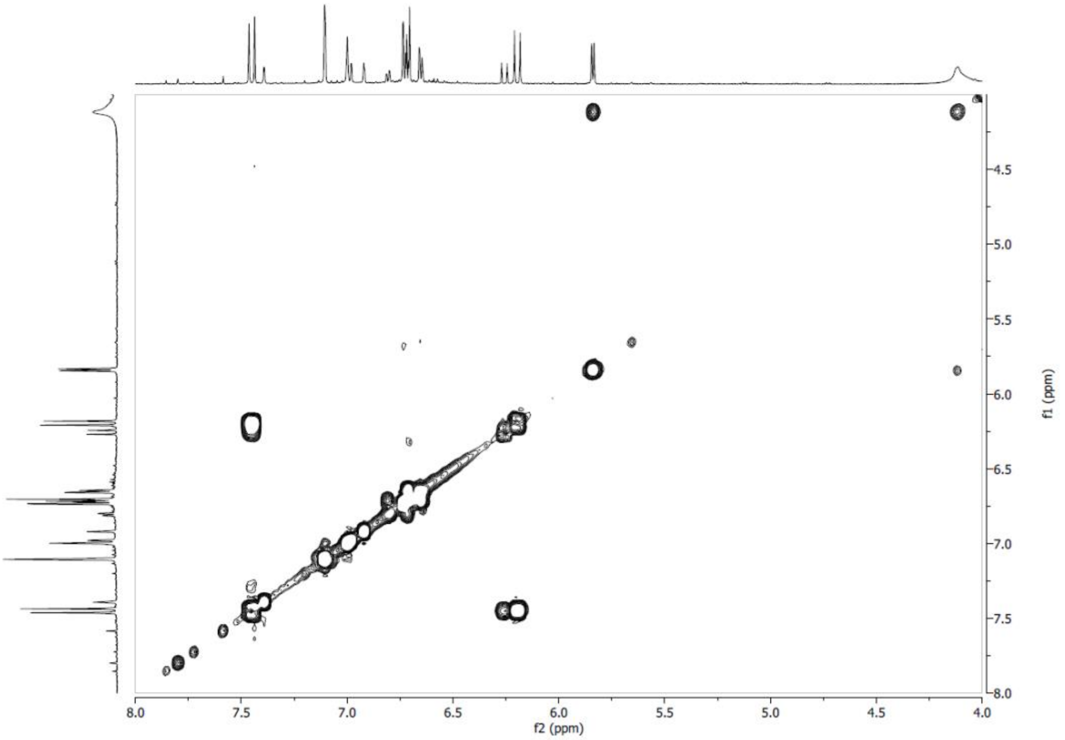

Supplementary Figure 24. COSY NMR spectrum of compound **4** in DMSO-d_6

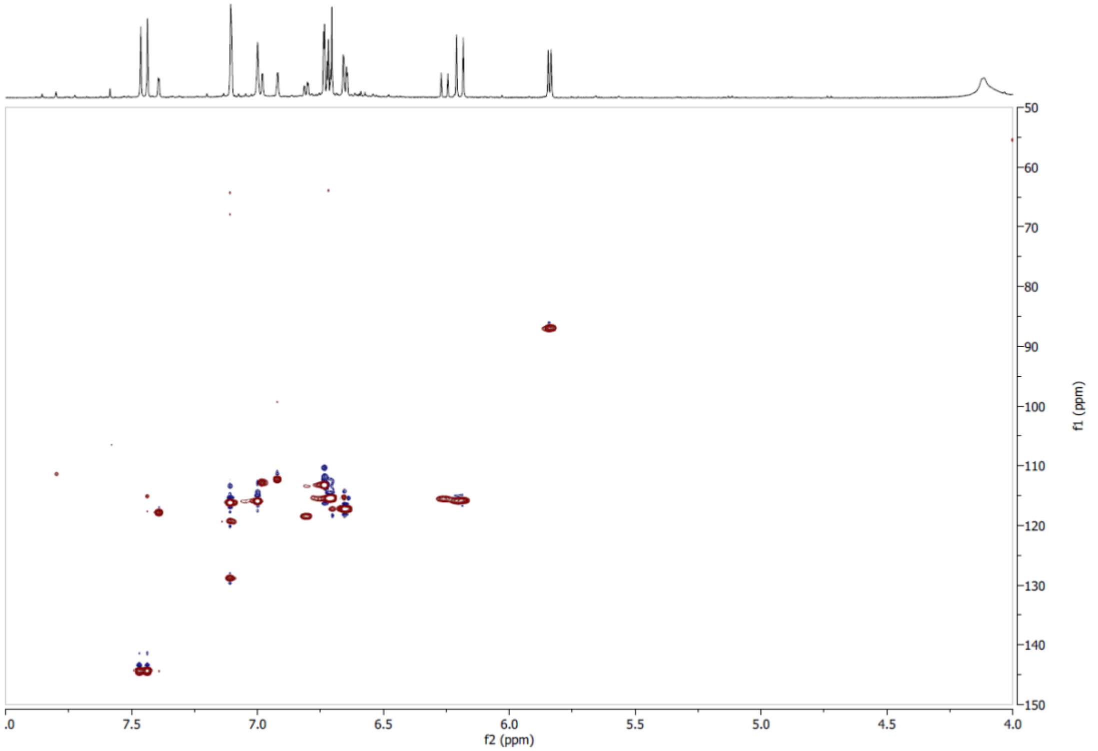

Supplementary Figure 25. Edited-HSQC NMR spectrum of compound **4** in DMSO-d_6

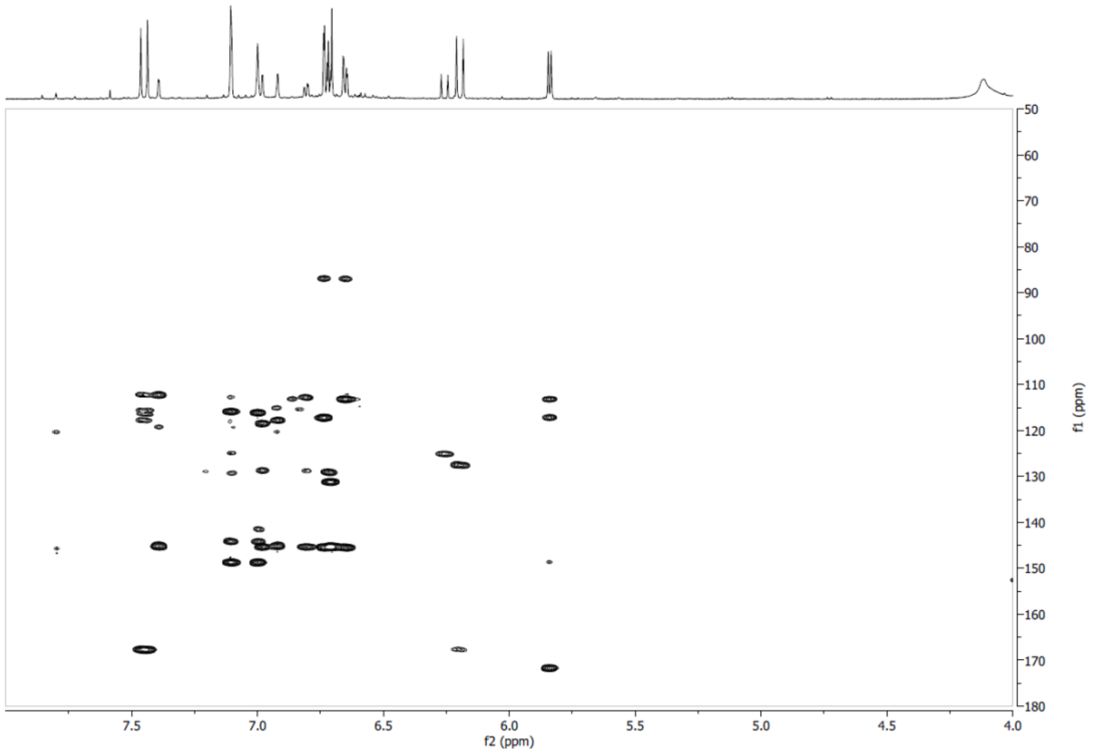

Supplementary Figure 26. HMBC NMR spectrum of compound **4** in DMSO-d_6

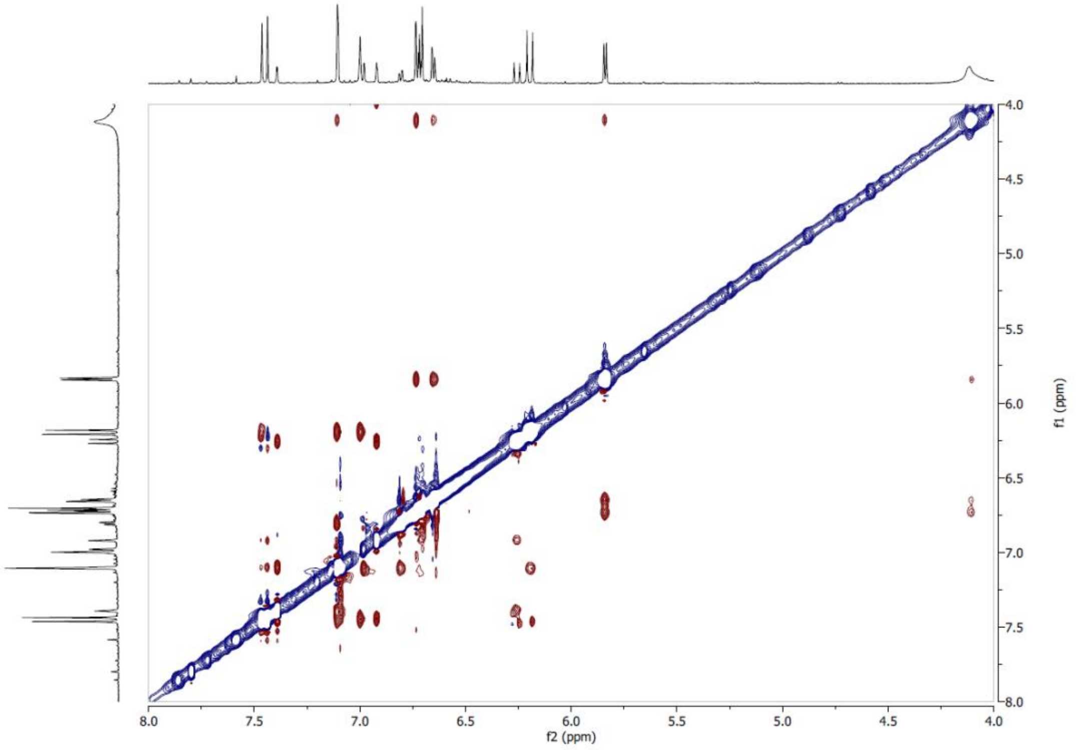

Supplementary Figure 27. ROESY NMR spectrum of compound **4** in DMSO-d_6

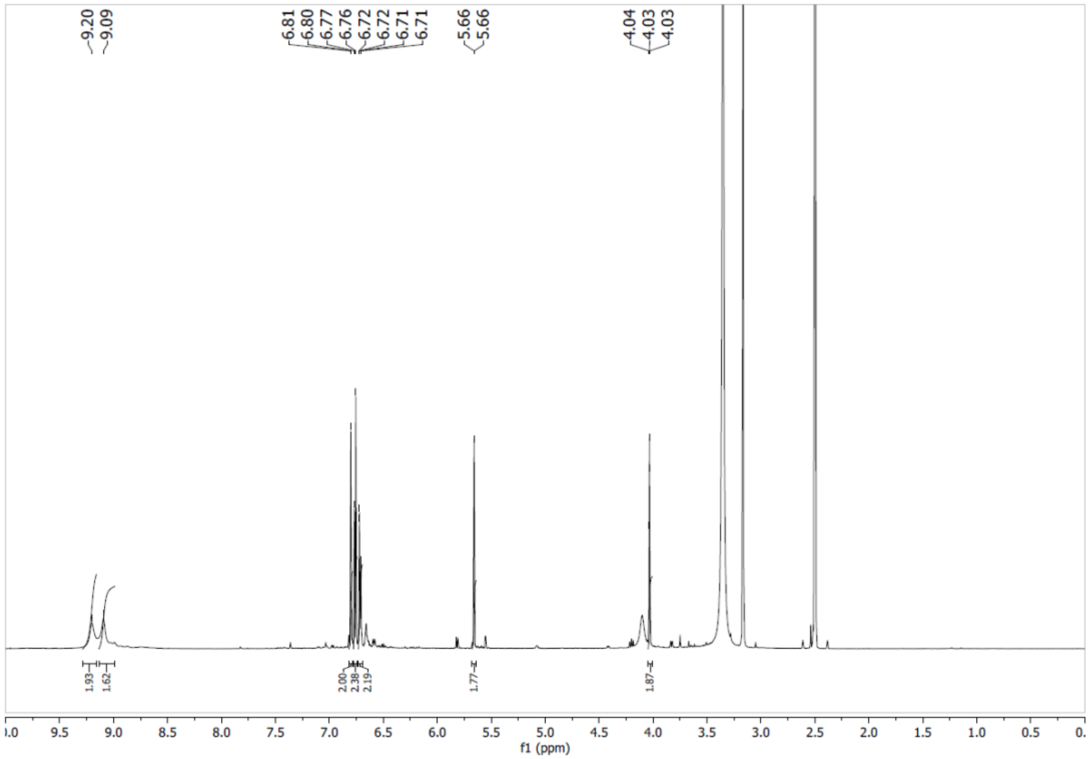

Supplementary Figure 28. ^{1}H NMR spectrum of compound **5** in DMSO-d_6 at 600 MHz

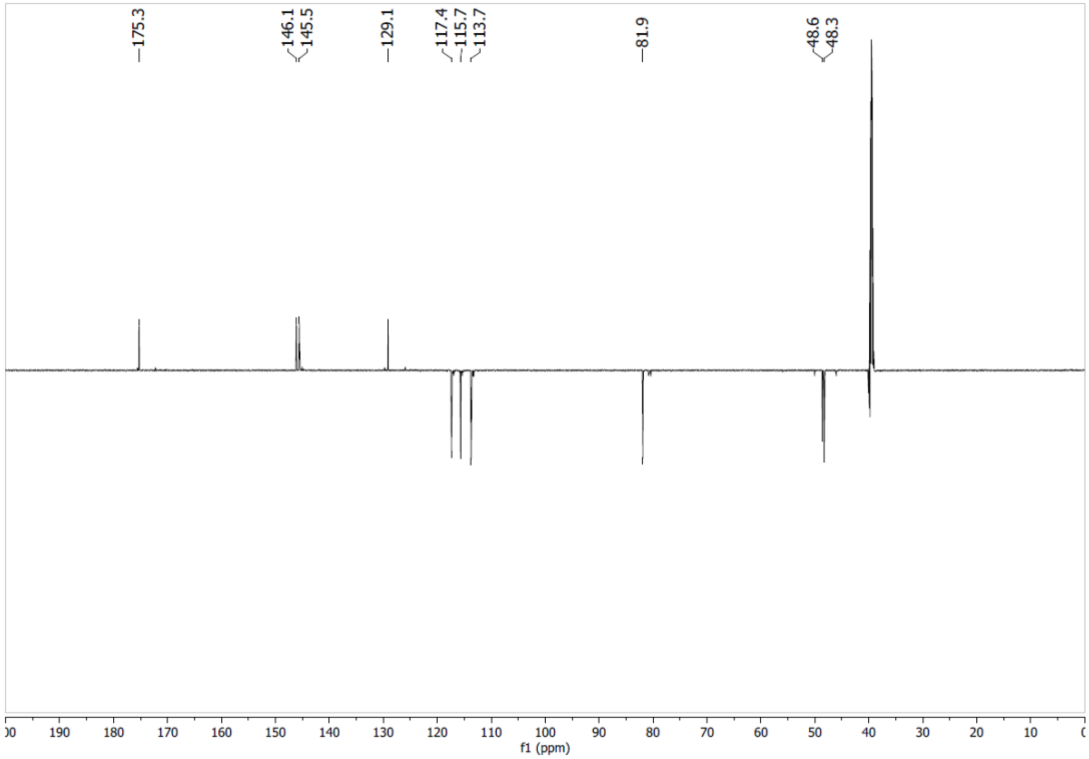

Supplementary Figure 29. ^{13}C-DEPTQ NMR spectrum of compound **5** in DMSO-d_6 at 151 MHz

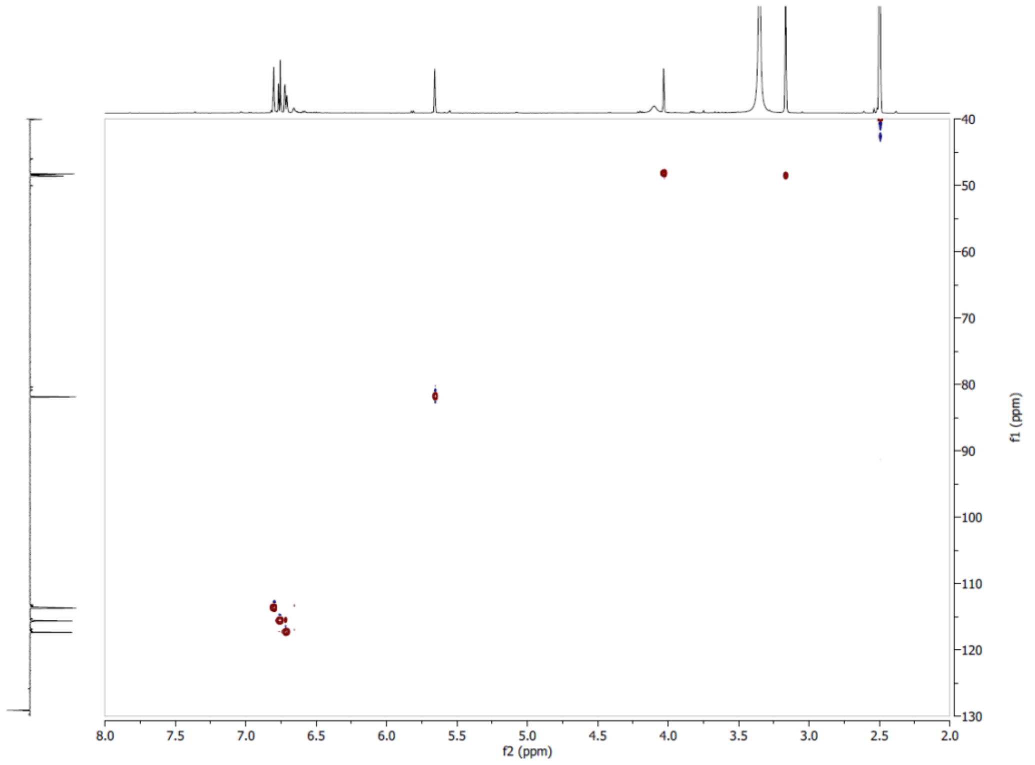

Supplementary Figure 30. Edited-HSQC NMR spectrum of compound **5** in DMSO-d_6

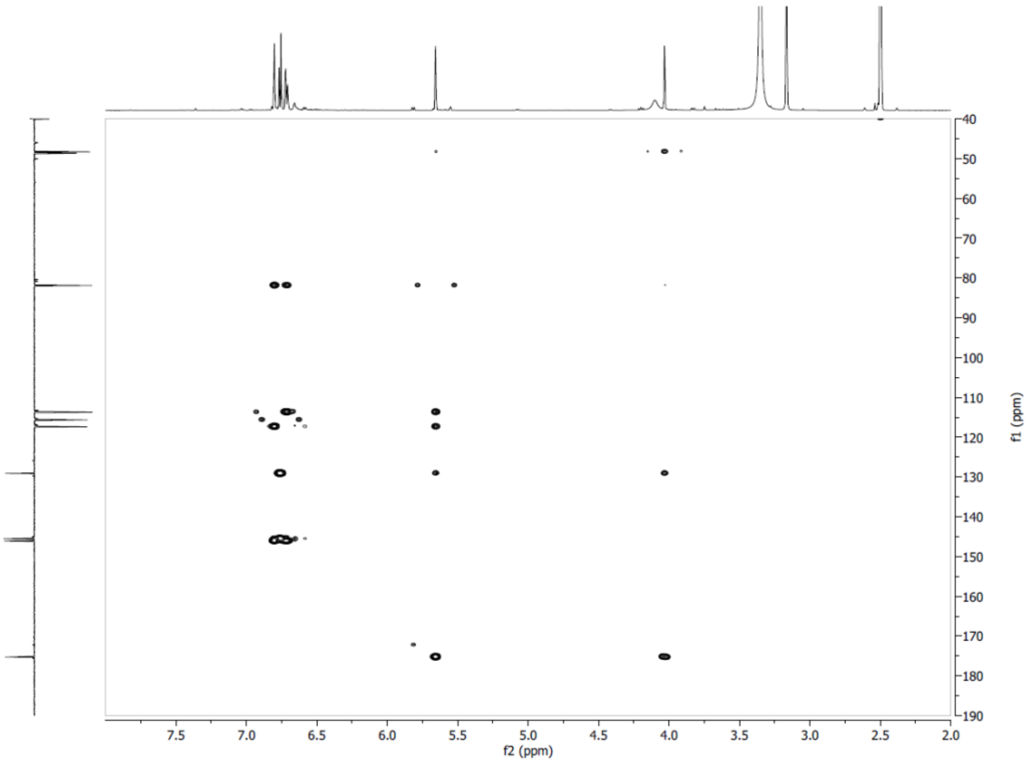

Supplementary Figure 31. HMBC NMR spectrum of compound **5** in DMSO-d_6

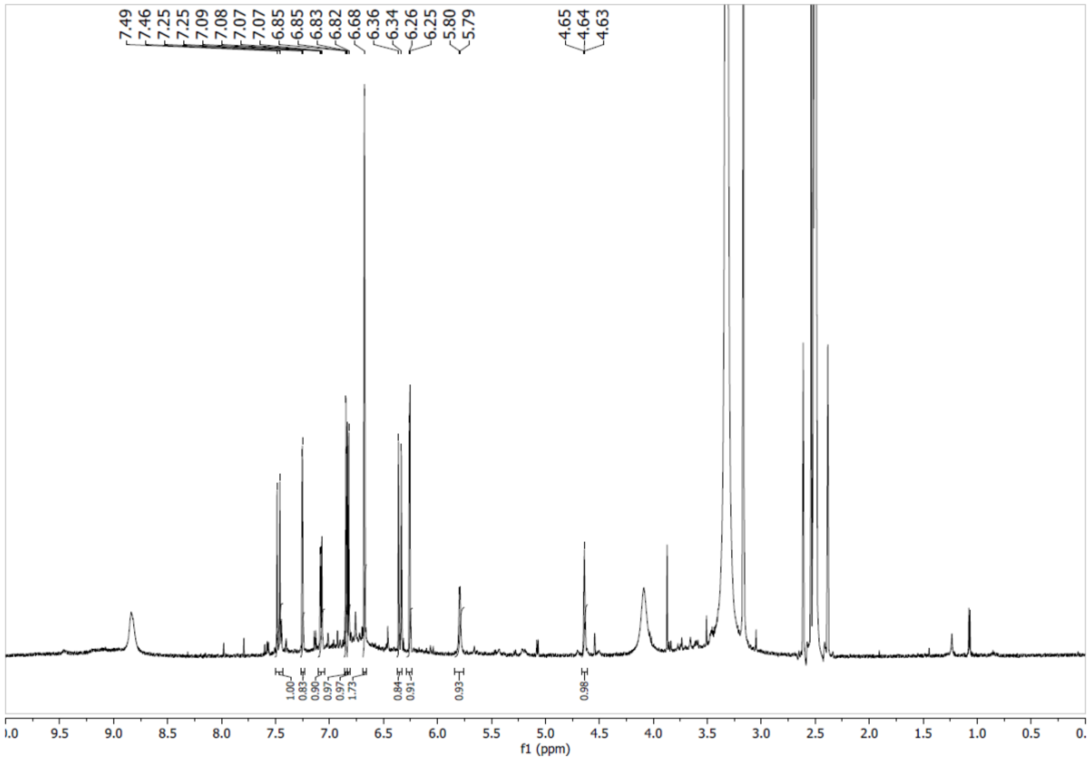

Supplementary Figure 32. ^{1}H NMR spectrum of compound **6** in DMSO-d_6 at 600 MHz

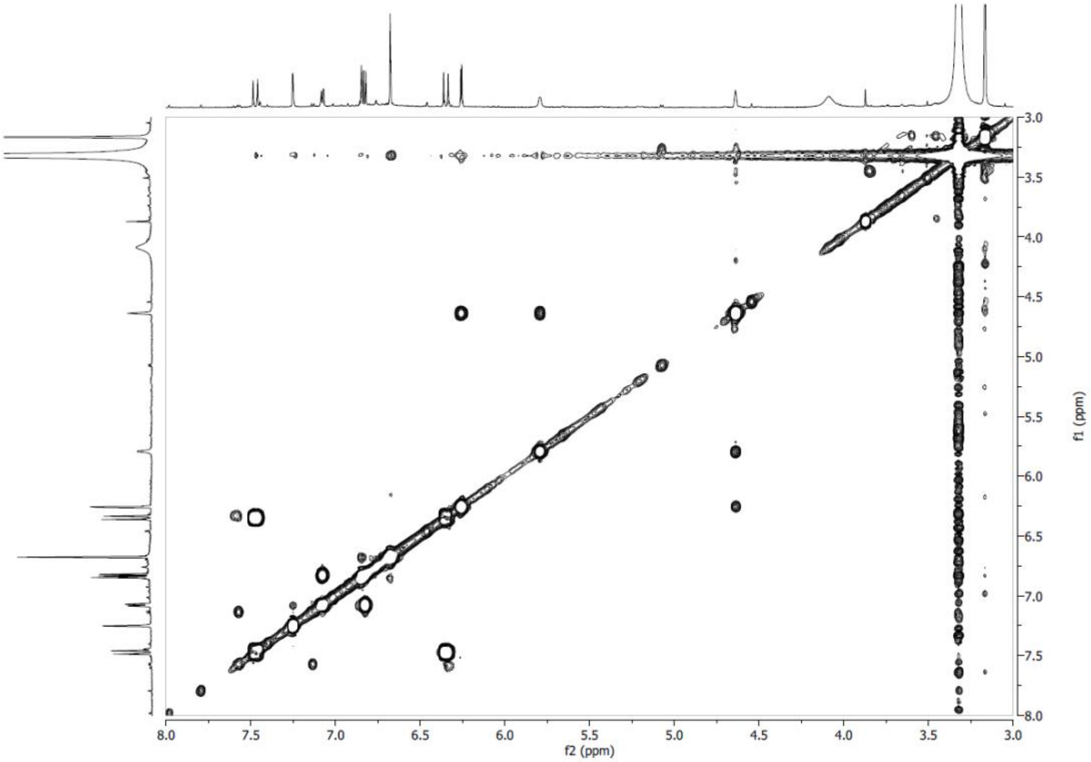

Supplementary Figure 33. COSY NMR spectrum of compound **6** in DMSO-d_6

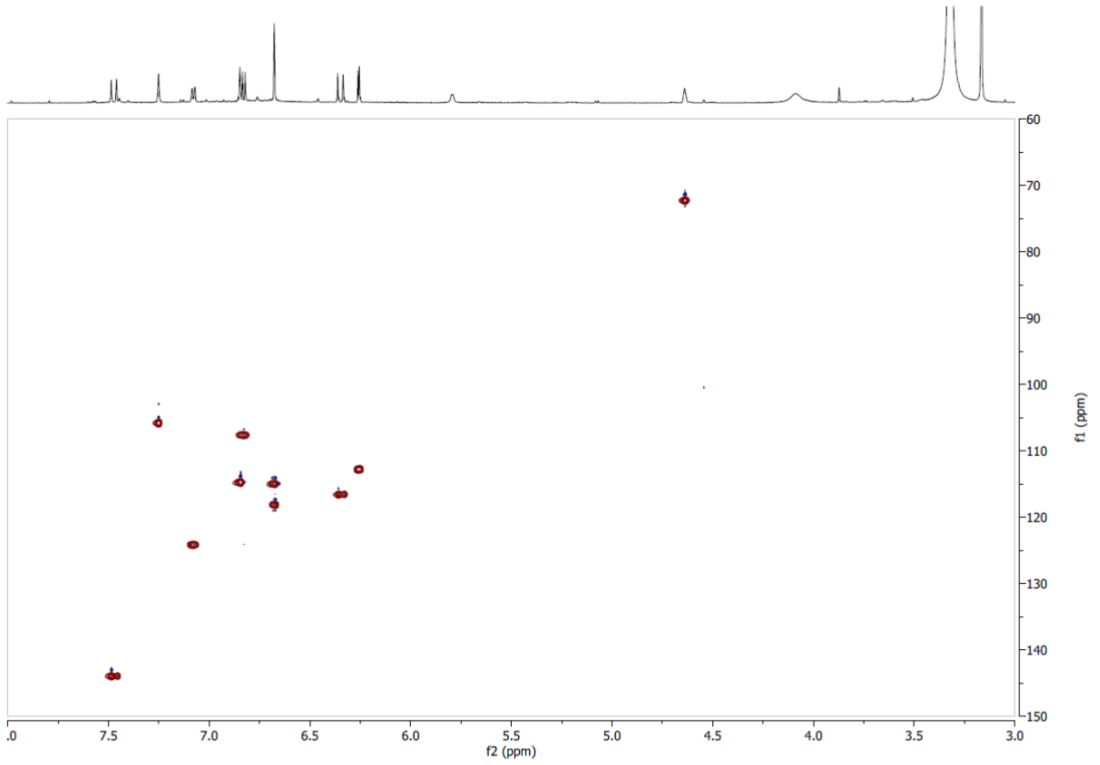

Supplementary Figure 34. Edited-HSQC NMR spectrum of compound **6** in DMSO-*d*6

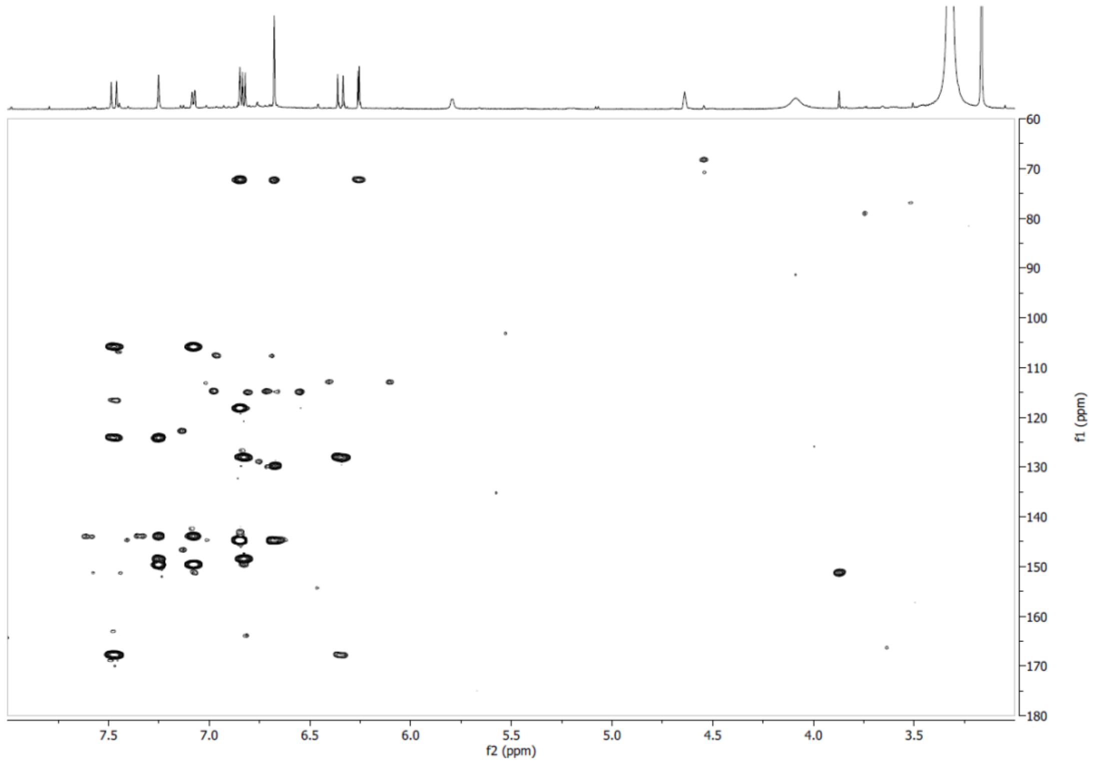

Supplementary Figure 35. HMBC NMR spectrum of compound **6** in DMSO-*d*6

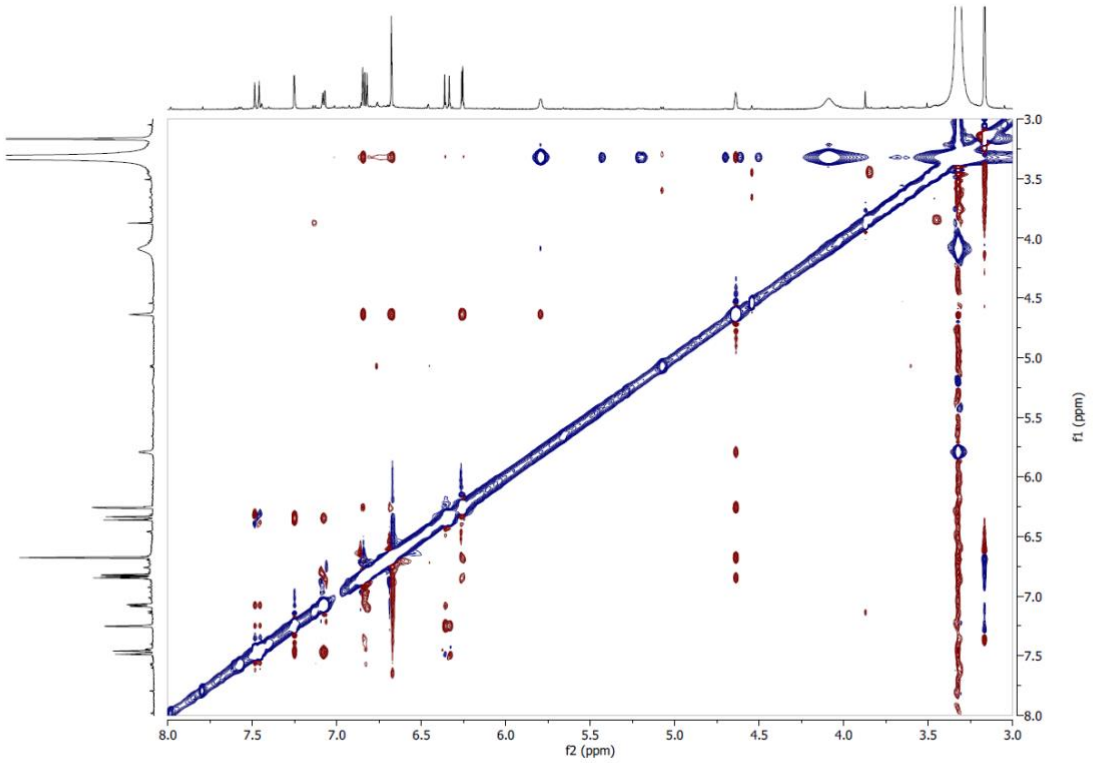

Supplementary Figure 36. ROESY NMR spectrum of compound **6** in DMSO-d_6

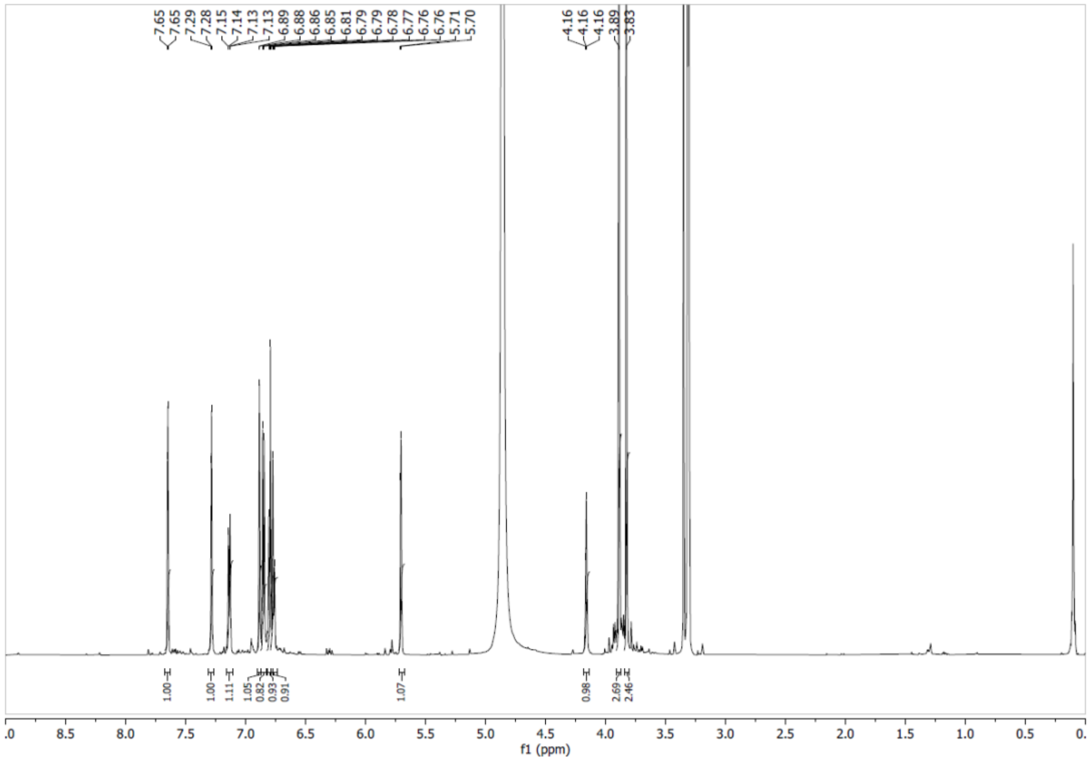

Supplementary Figure 37. ^{1}H NMR spectrum of compound **7** in DMSO-d_6 at 600 MHz

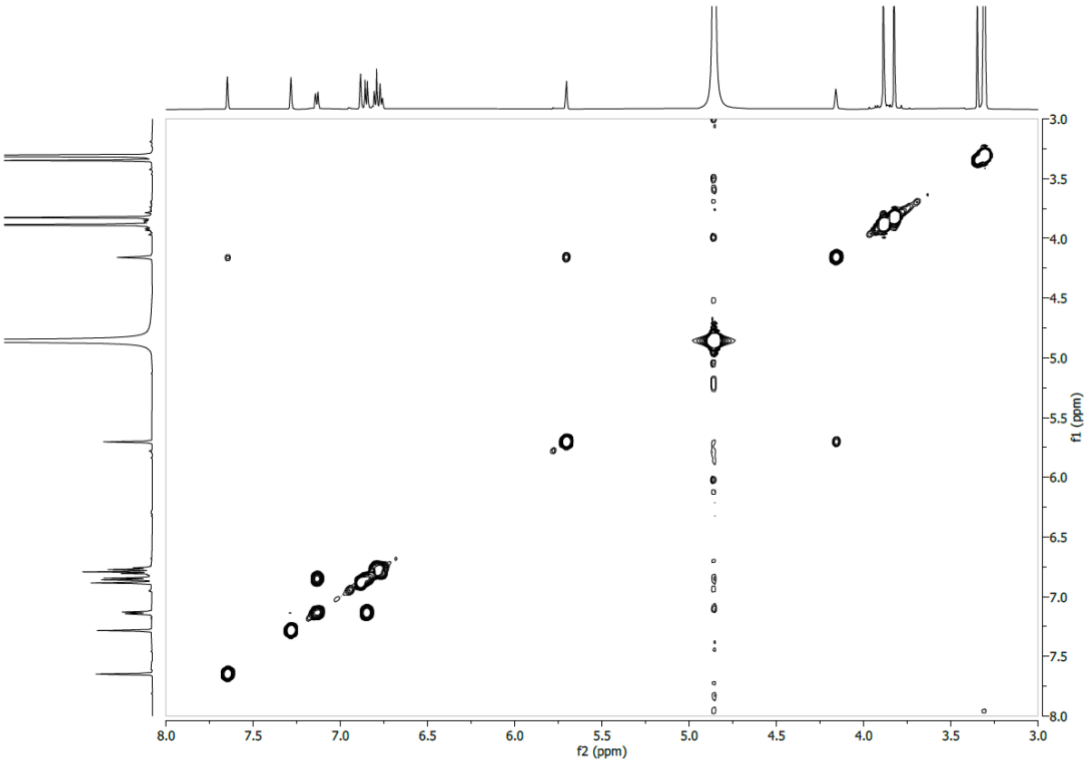

Supplementary Figure 38. COSY NMR spectrum of compound **7** in DMSO-d_6

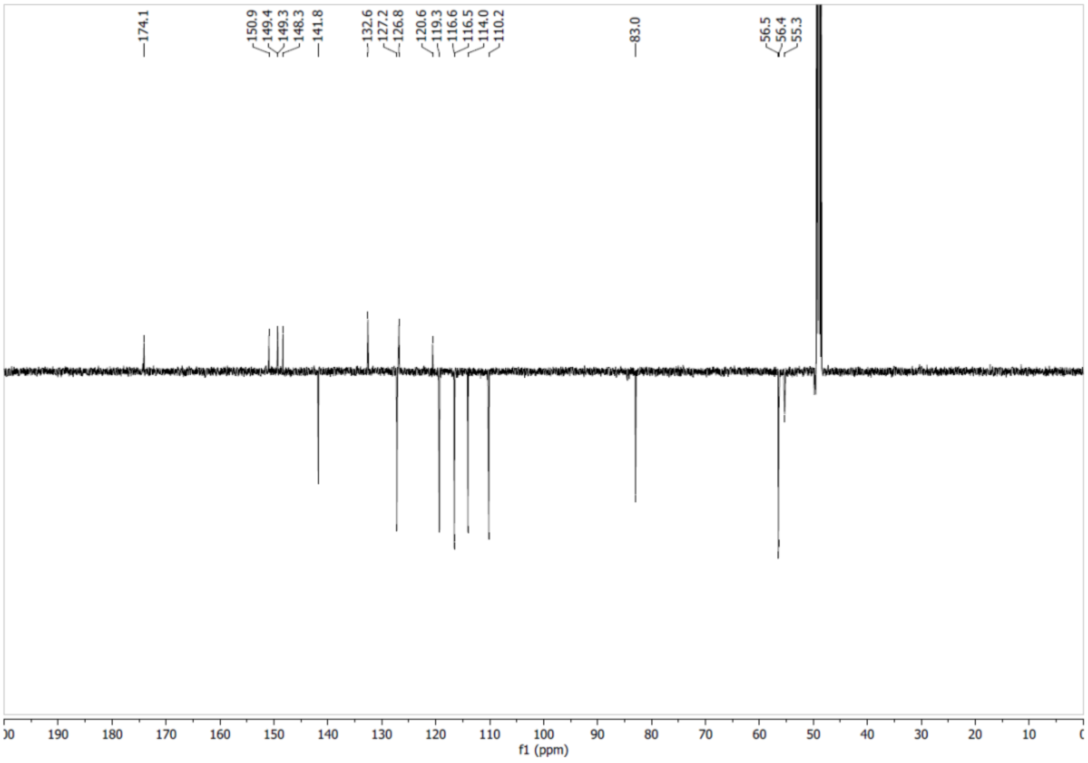

Supplementary Figure 39. ^{13}C NMR spectrum of compound **7** in DMSO-d_6 at 151 MHz

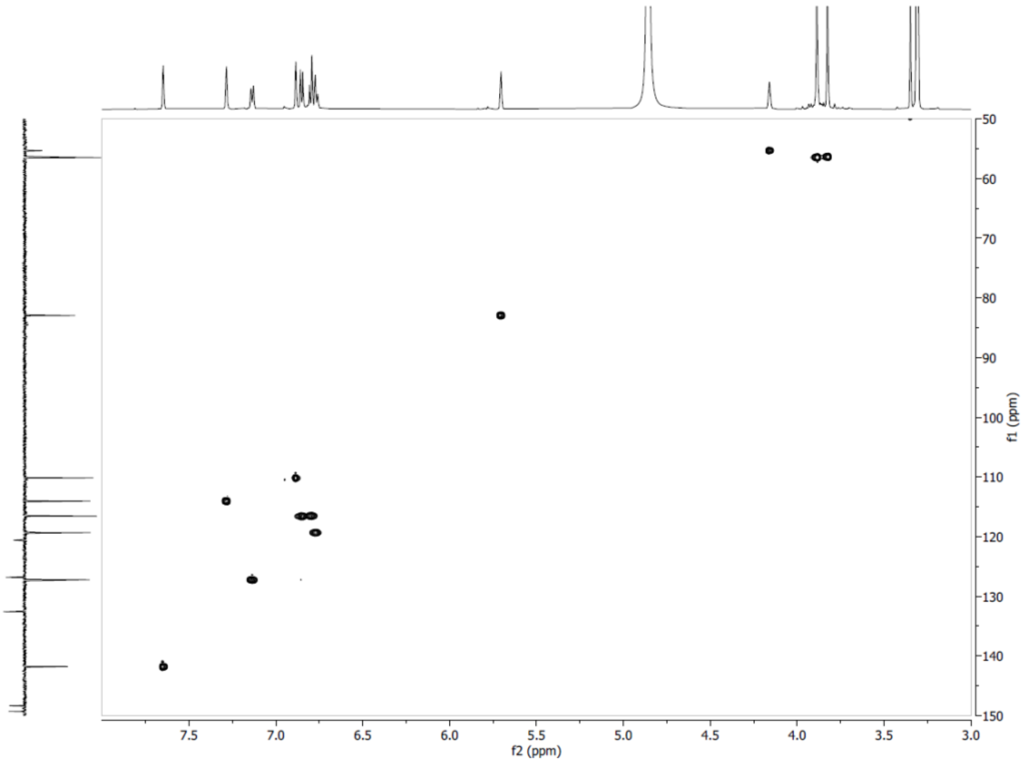

Supplementary Figure 40. Edited-HSQC NMR spectrum of compound **7** in DMSO-d_6

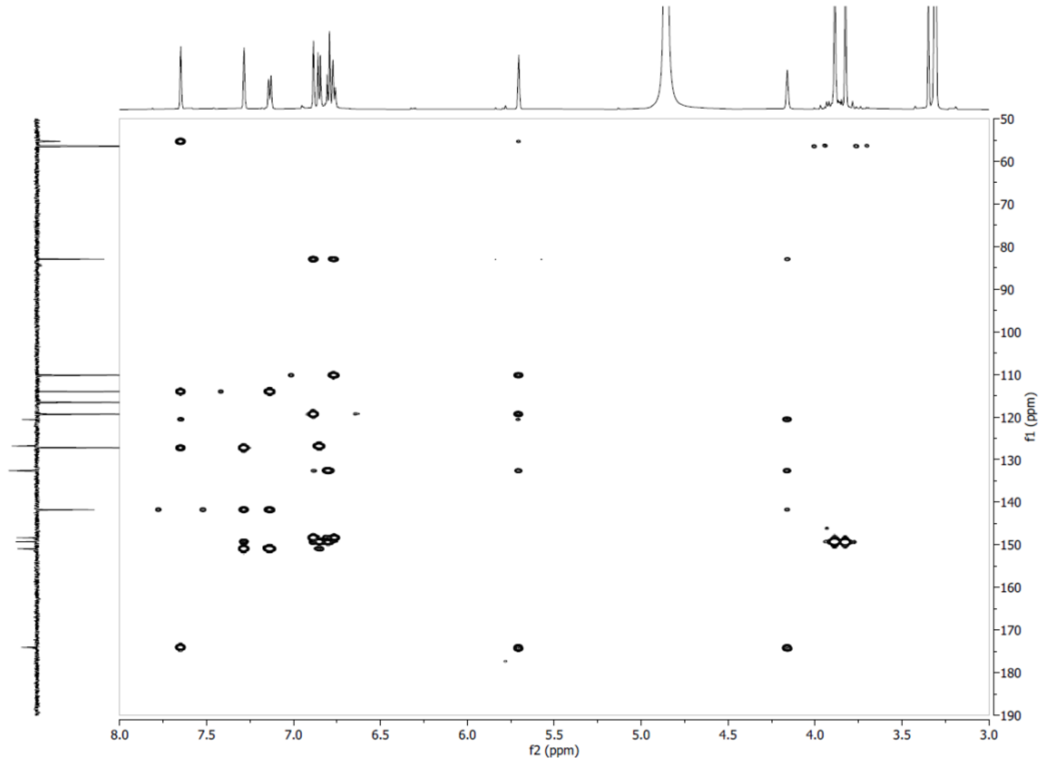

Supplementary Figure 41. HMBC NMR spectrum of compound **7** in DMSO-d_6

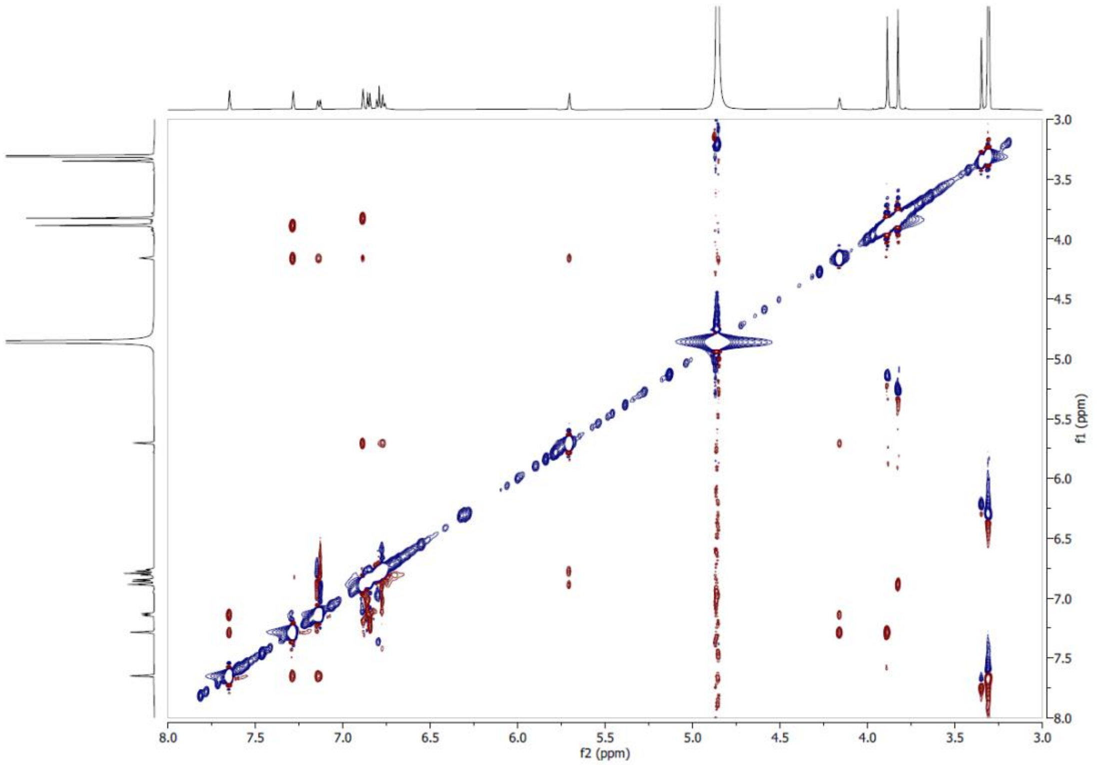

Supplementary Figure 42. ROESY NMR spectrum of compound **7** in DMSO-d_6

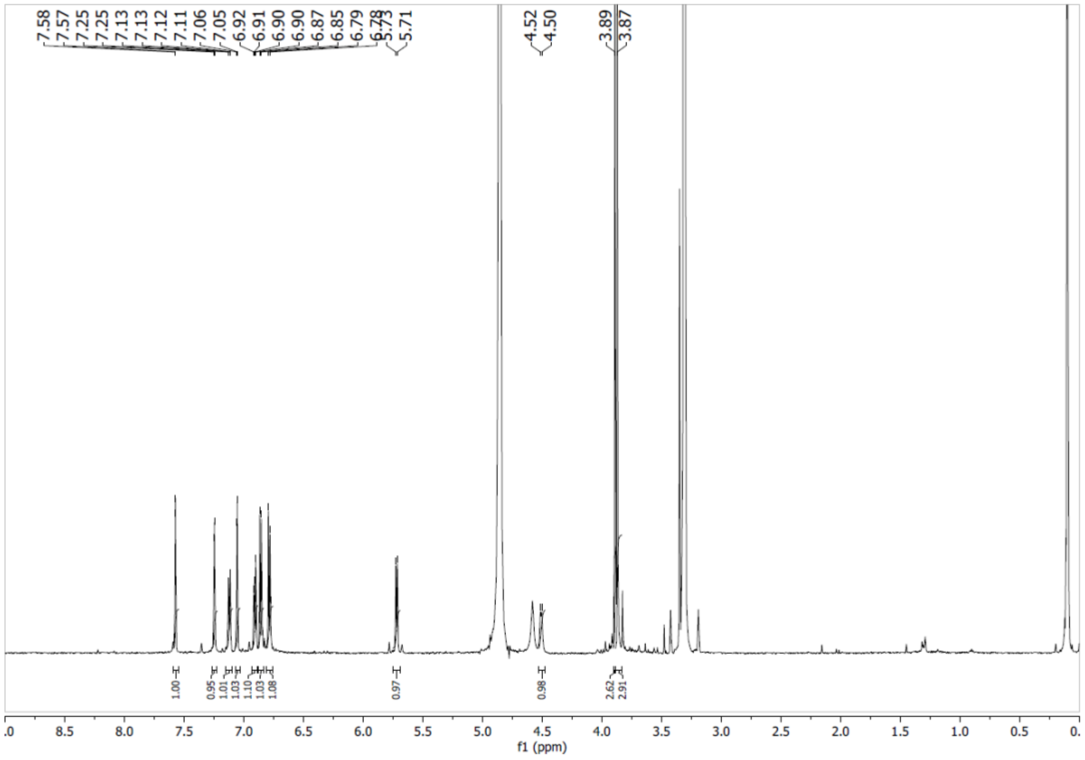

Supplementary Figure 43. ^{1}H NMR spectrum of compound **8** in DMSO-d_6 at 600 MHz

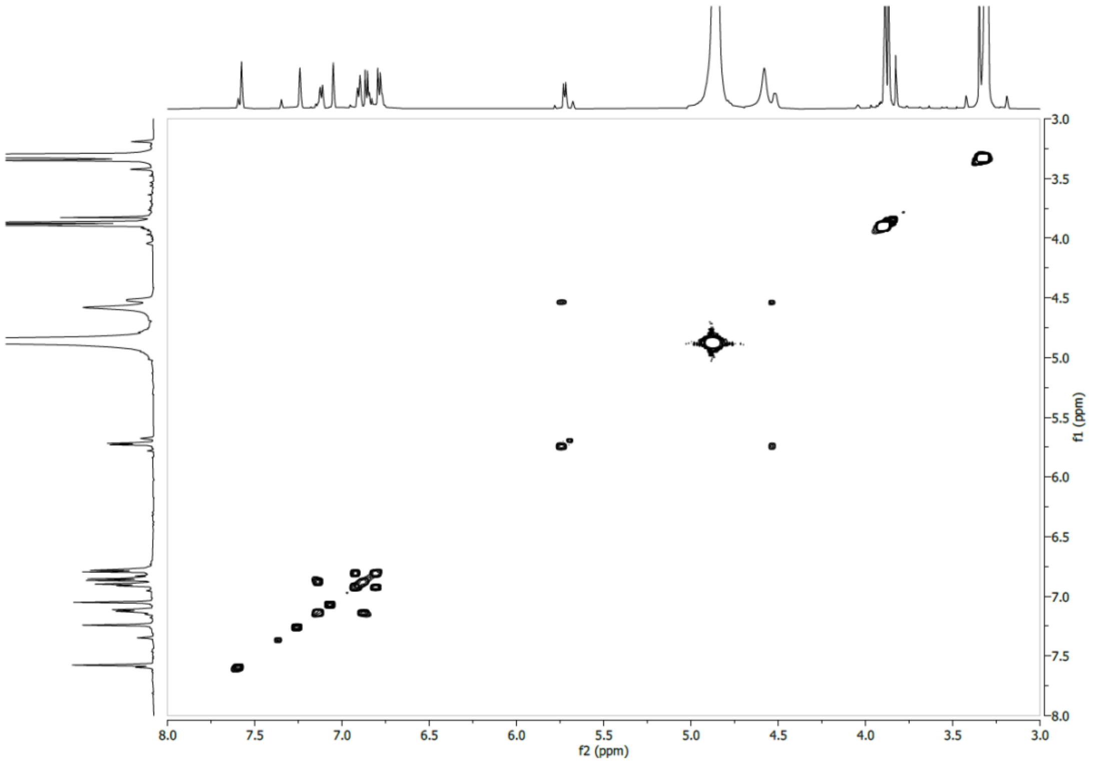

Supplementary Figure 44. COSY NMR spectrum of compound **8** in DMSO-d_6

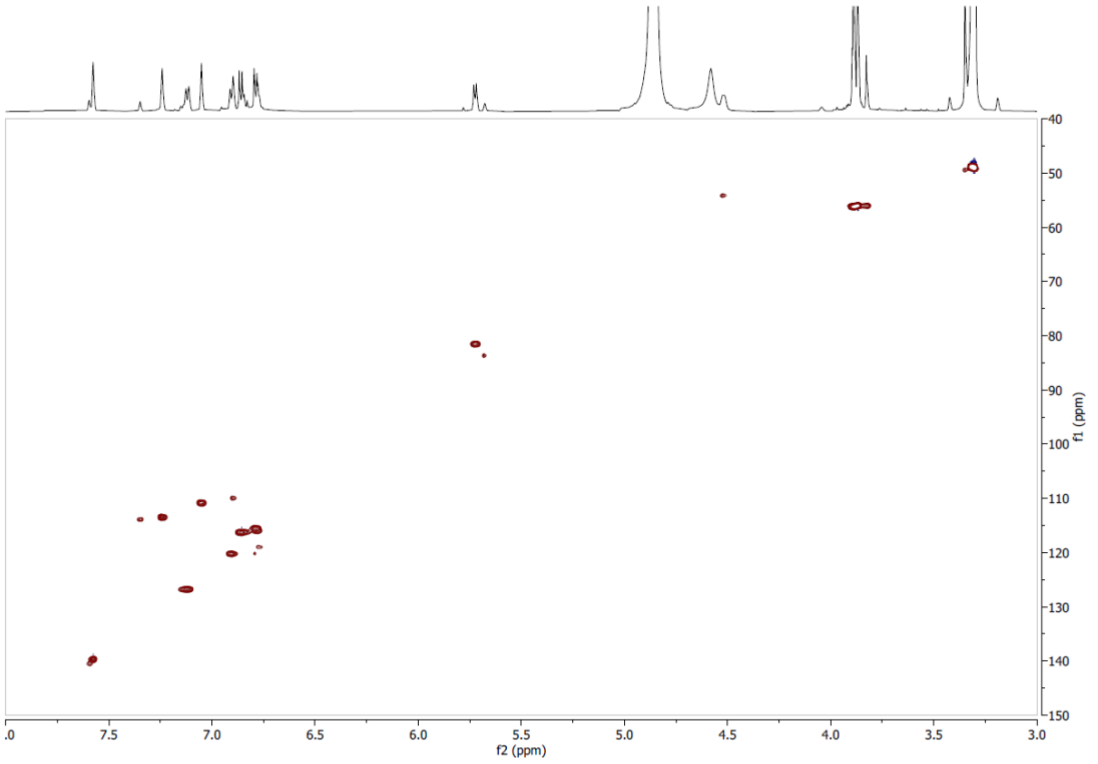

Supplementary Figure 45. Edited-HSQC NMR spectrum of compound **8** in DMSO-d_6

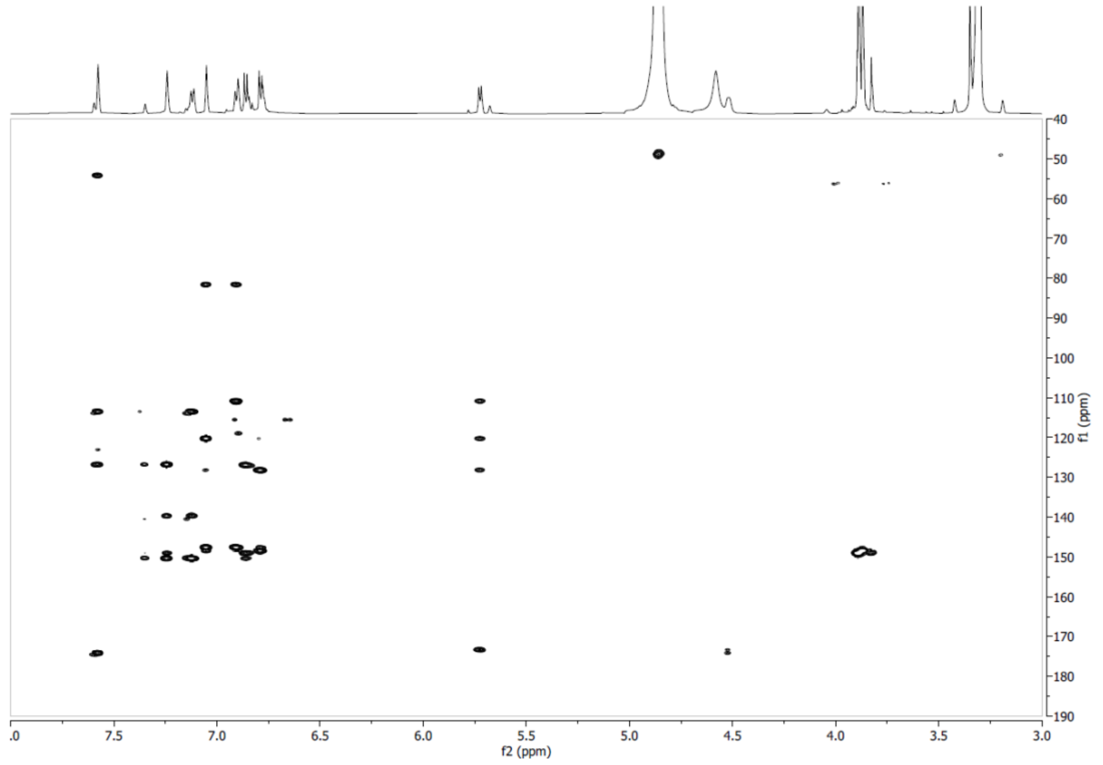

Supplementary Figure 46. HMBC NMR spectrum of compound **8** in DMSO-d_6

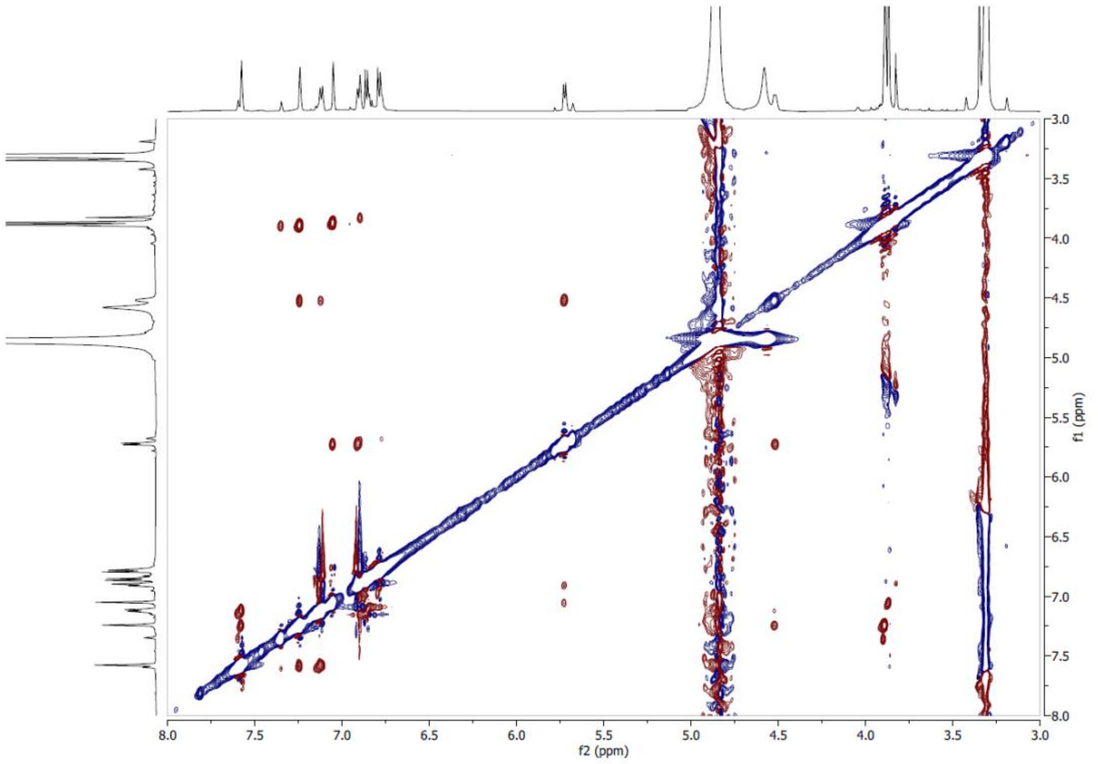

Supplementary Figure 47. ROESY NMR spectrum of compound **8** in DMSO-d_6

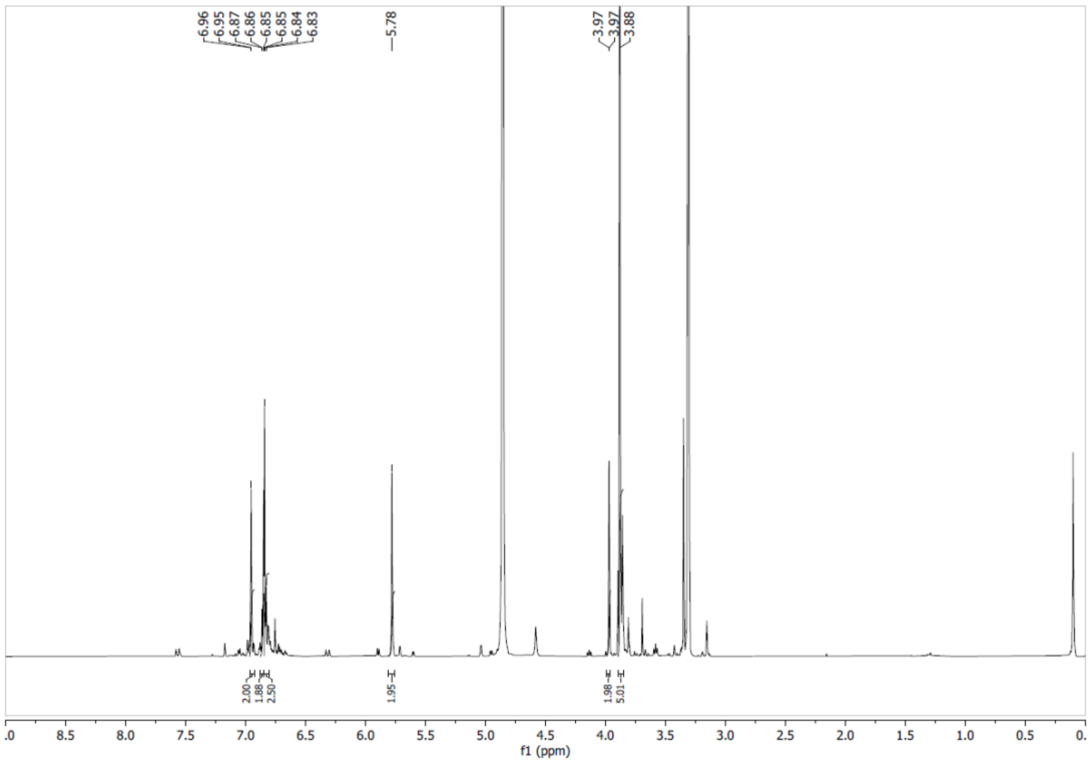

Supplementary Figure 48. ^{1}H NMR spectrum of compound **9** in DMSO-d_6 at 600 MHz

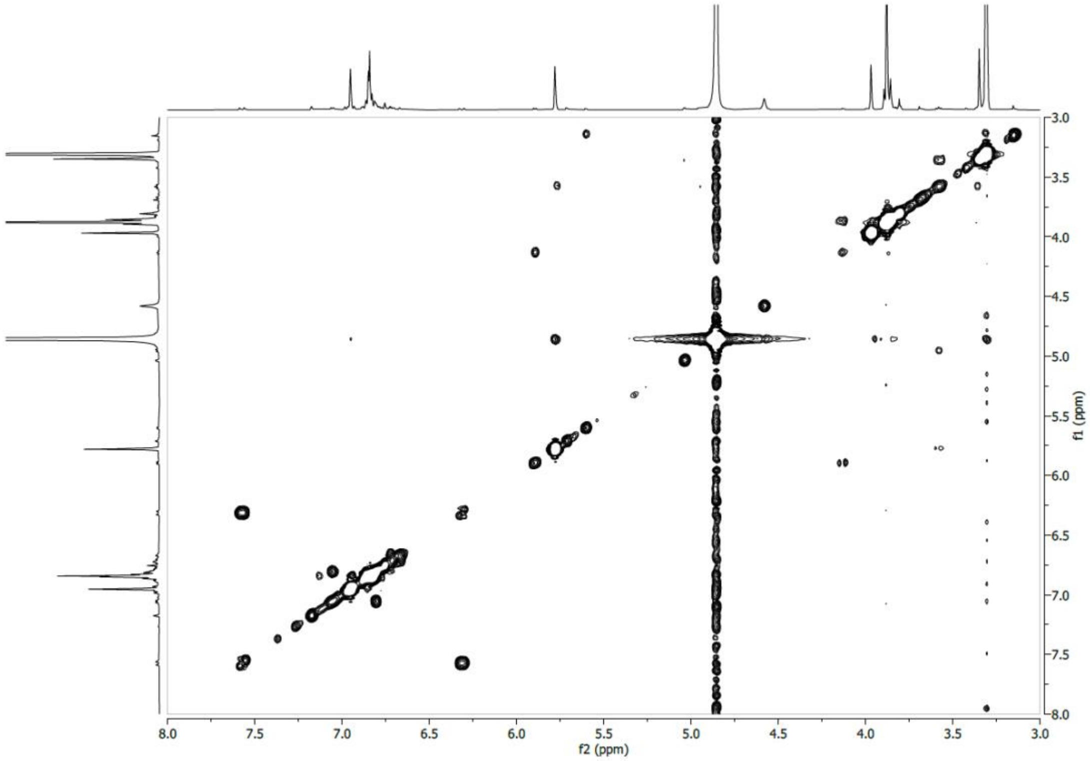

Supplementary Figure 49. COSY NMR spectrum of compound **9** in DMSO-d_6

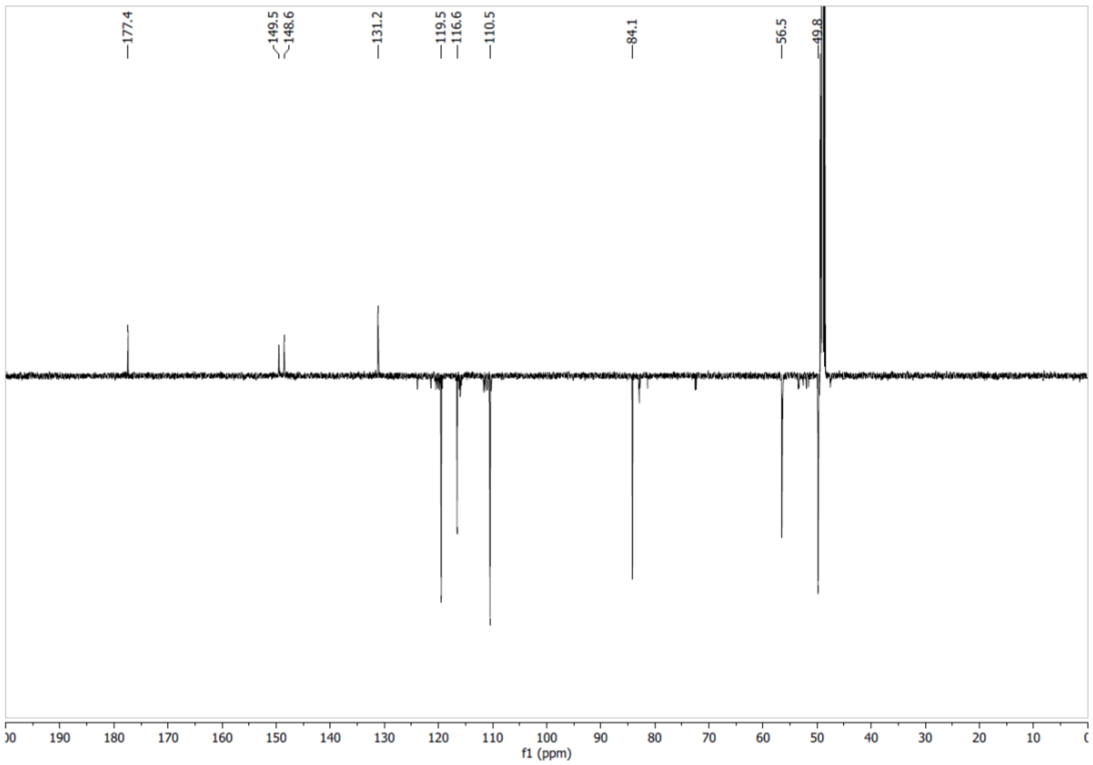

Supplementary Figure 50. ^{13}C-DEPTQ NMR spectrum of compound **9** in DMSO-d_6 at 151 MHz

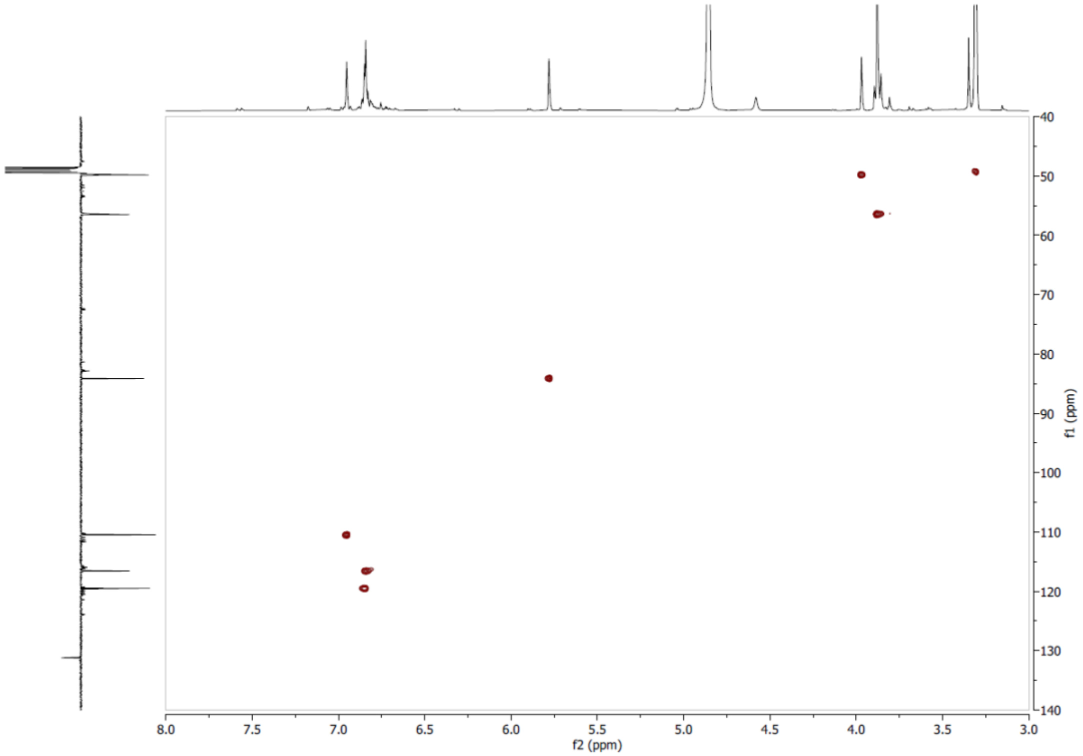

Supplementary Figure 51. Edited-HSQC NMR spectrum of compound **9** in DMSO-d_6

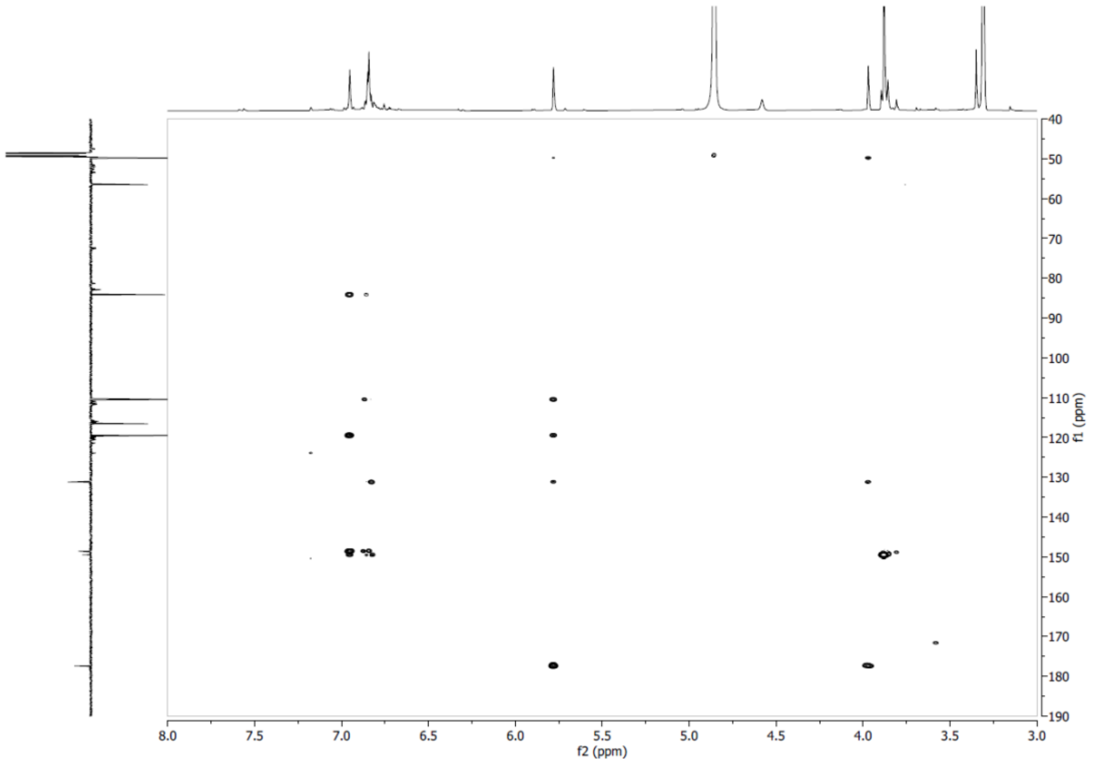

Supplementary Figure 52. HMBC NMR spectrum of compound **9** in DMSO-d_6

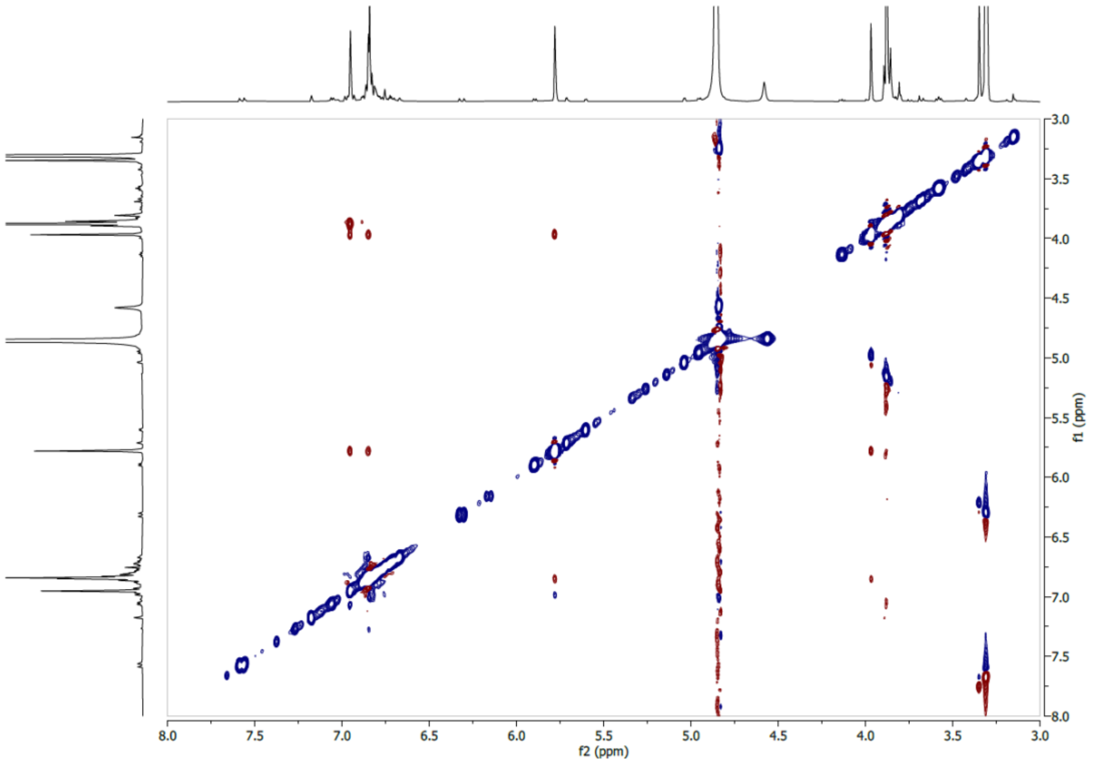

Supplementary Figure 53. ROESY NMR spectrum of compound **9** in DMSO-d_6

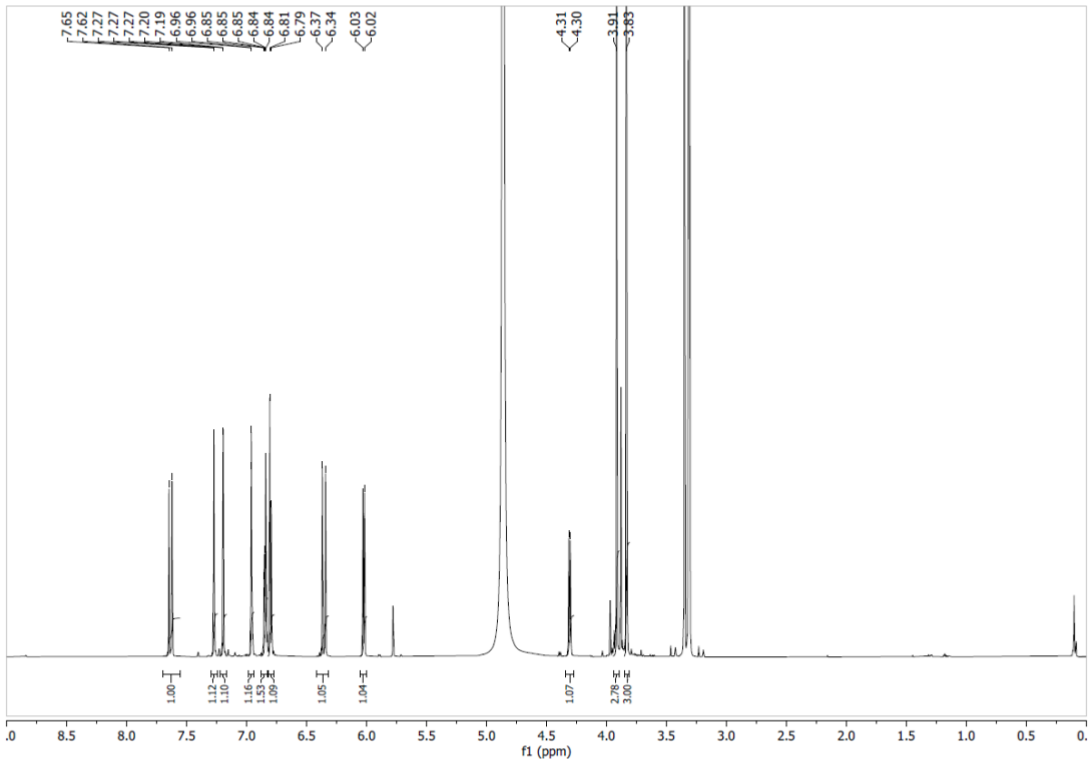

Supplementary Figure 54. ^{1}H NMR spectrum of compound **10** in DMSO-d_6 at 600 MHz

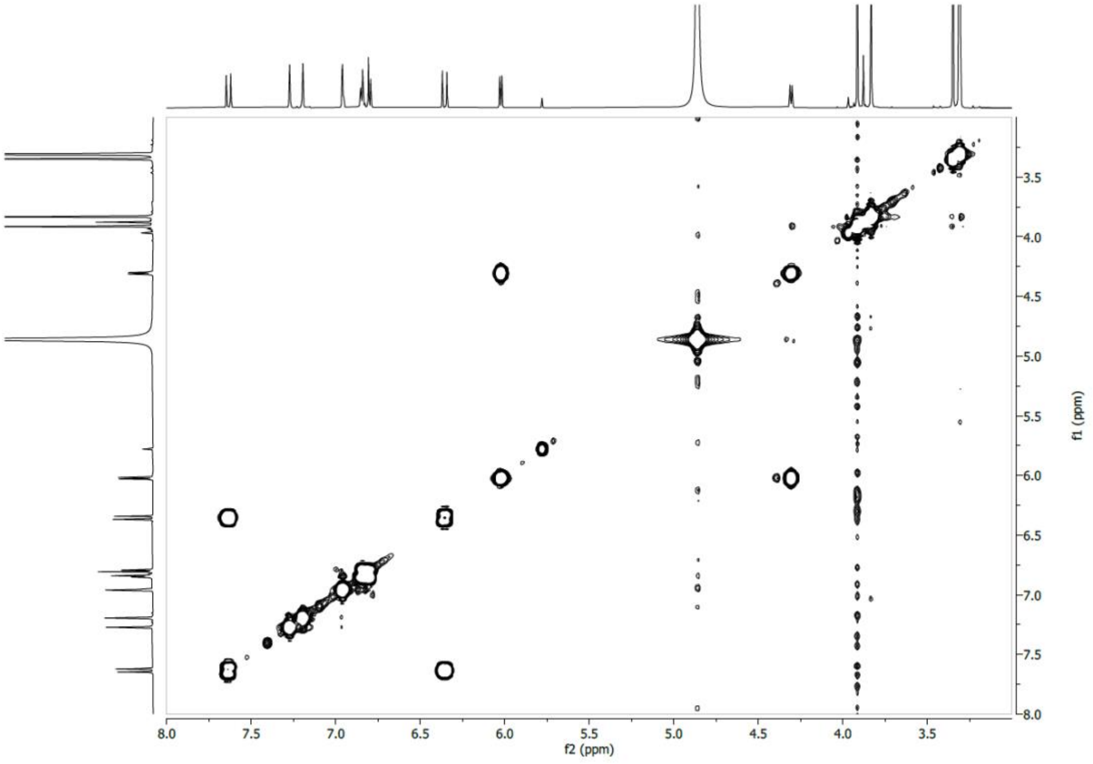

Supplementary Figure 55. COSY NMR spectrum of compound **10** in DMSO-d_6

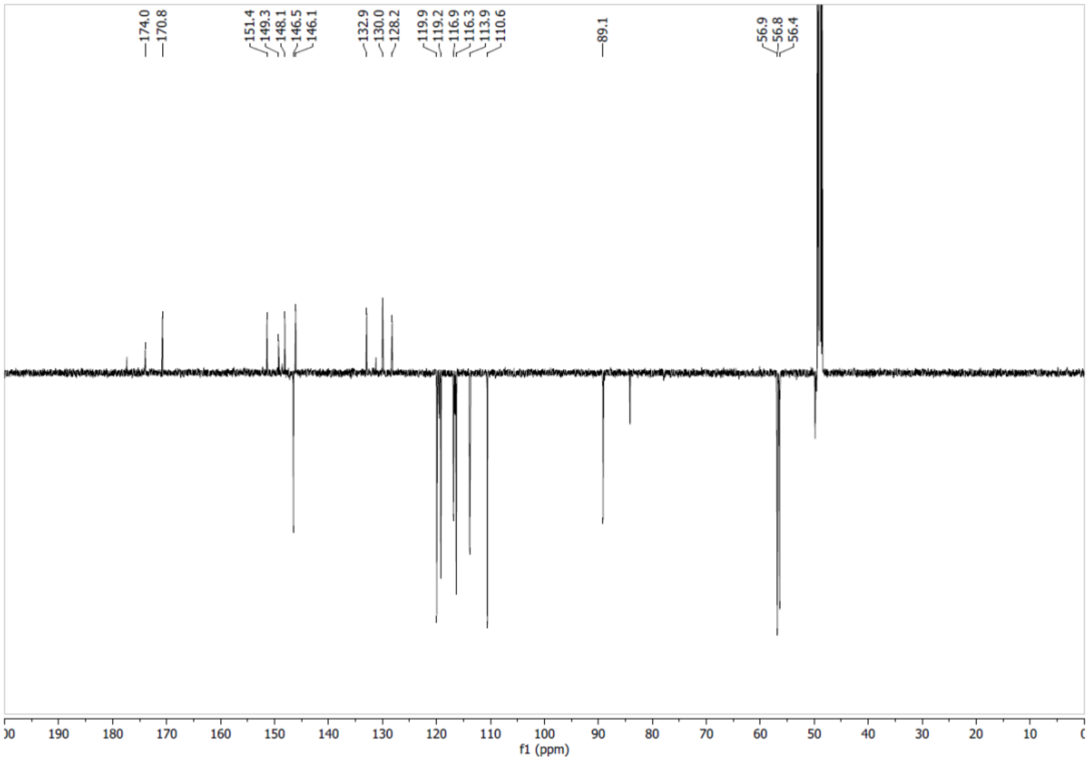

Supplementary Figure 56. ^{13}C-DEPTQ NMR spectrum of compound **10** in DMSO-d_6 at 151 MHz

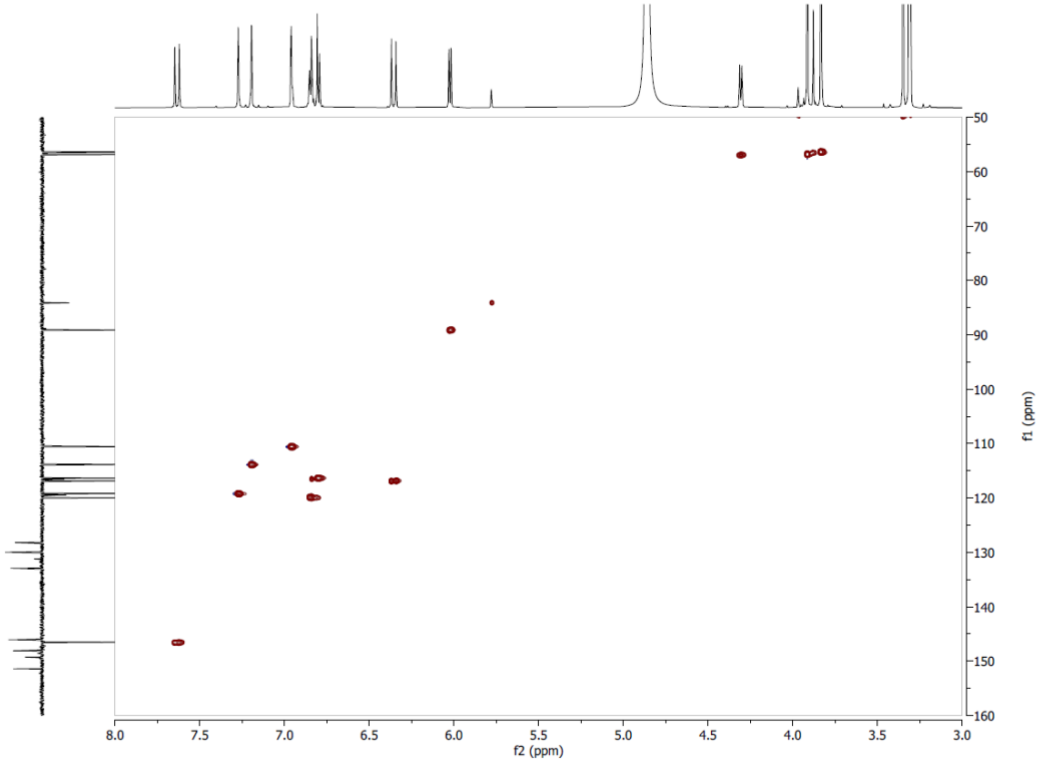

Supplementary Figure 57. Edited-HSQC NMR spectrum of compound **10** in DMSO-d_6

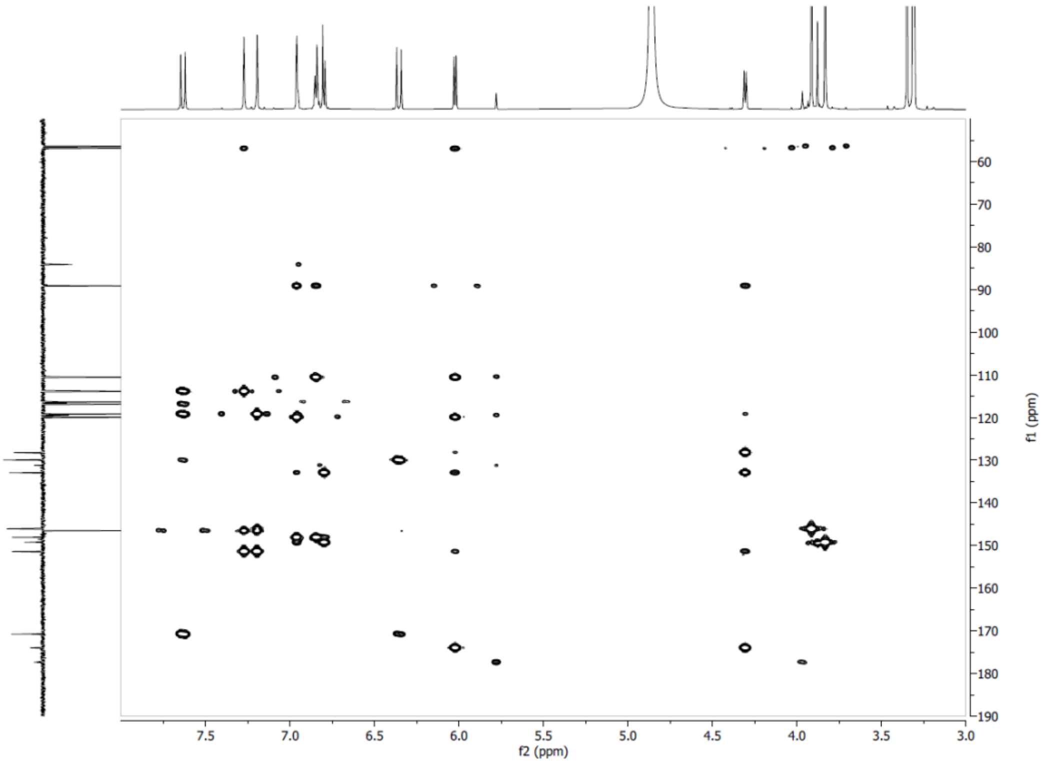

Supplementary Figure 58. HMBC NMR spectrum of compound **10** in DMSO-d_6

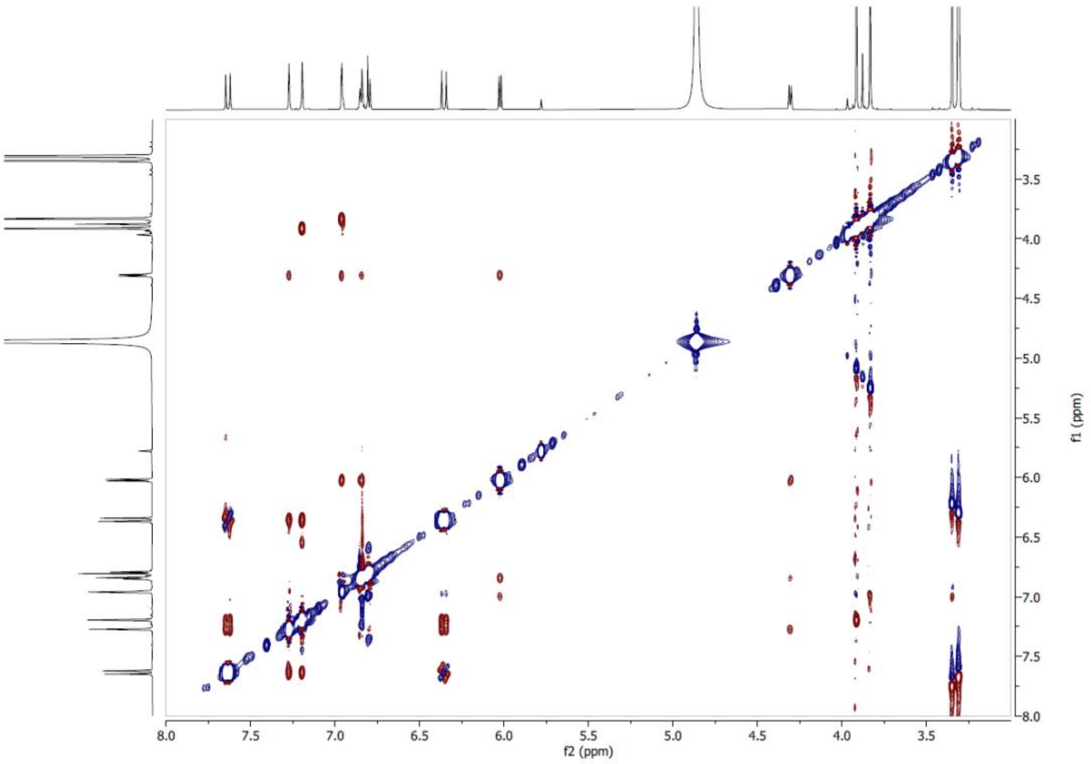

Supplementary Figure 59. ROESY NMR spectrum of compound **10** in DMSO-d_6

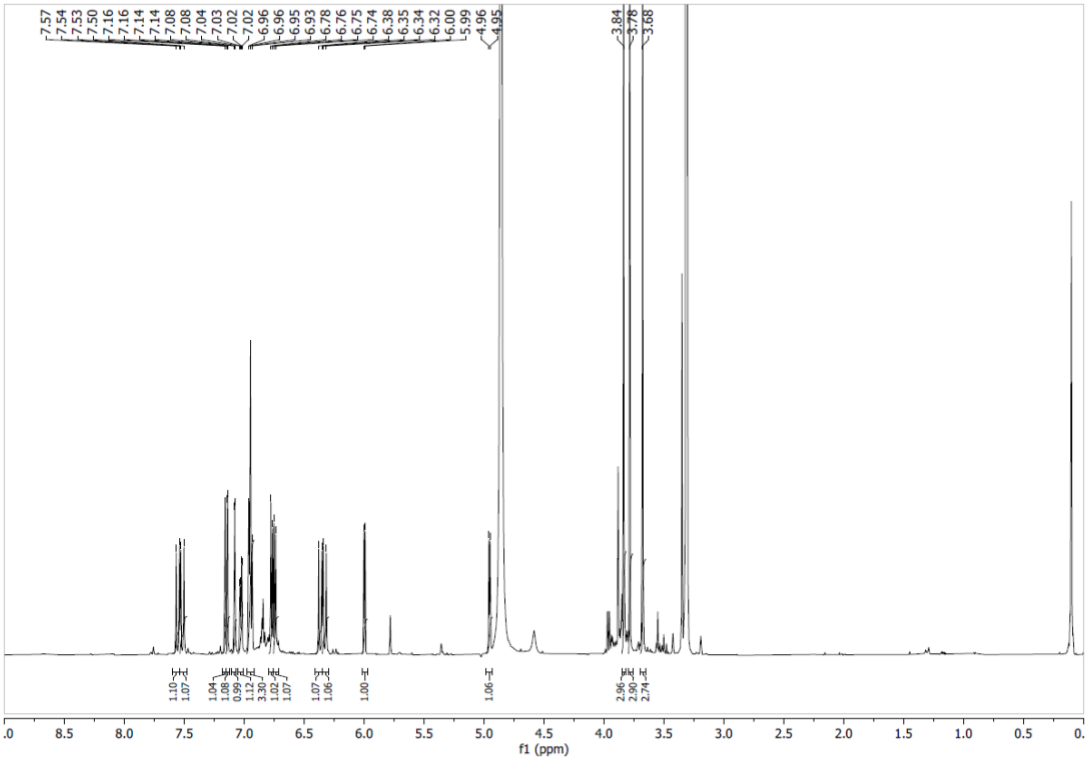

Supplementary Figure 60. ^{1}H NMR spectrum of compound **11** in DMSO-d_6 at 600 MHz

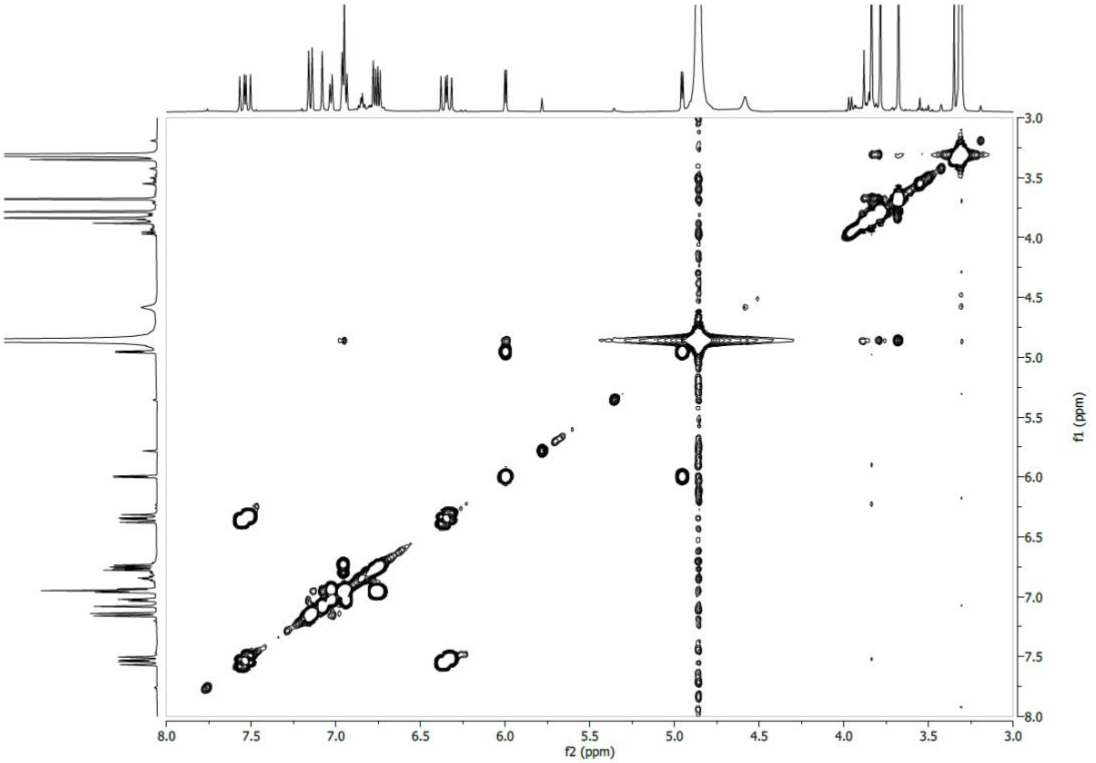

Supplementary Figure 61. COSY NMR spectrum of compound **11** in DMSO-d_6

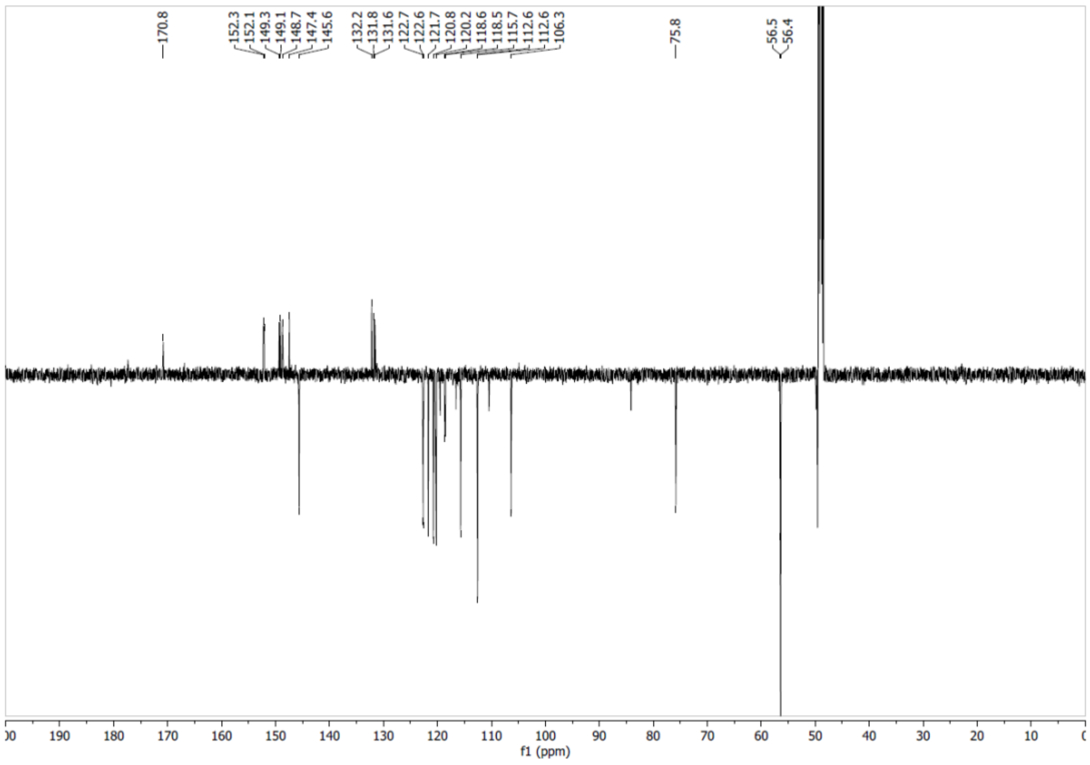

Supplementary Figure 62. ^{13}C-DEPTQ NMR spectrum of compound **11** in DMSO-d_6 at 151 MHz

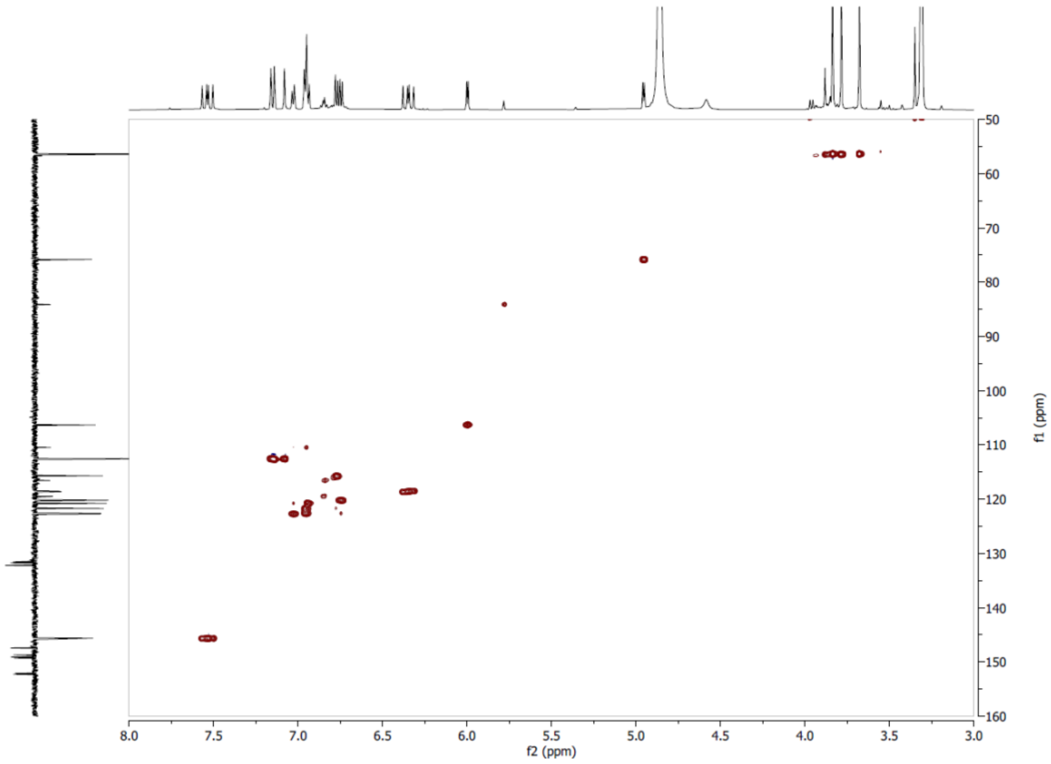

Supplementary Figure 63. Edited-HSQC NMR spectrum of compound **11** in DMSO-d_6

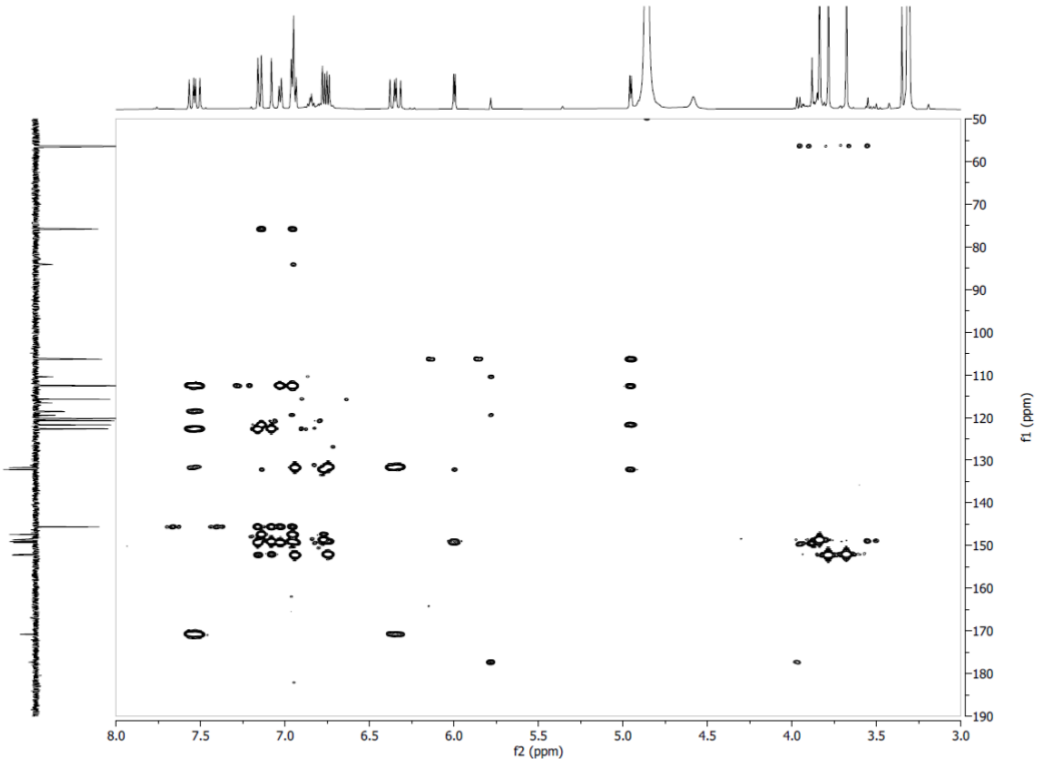

Supplementary Figure 64. HMBC NMR spectrum of compound **11** in DMSO-d_6

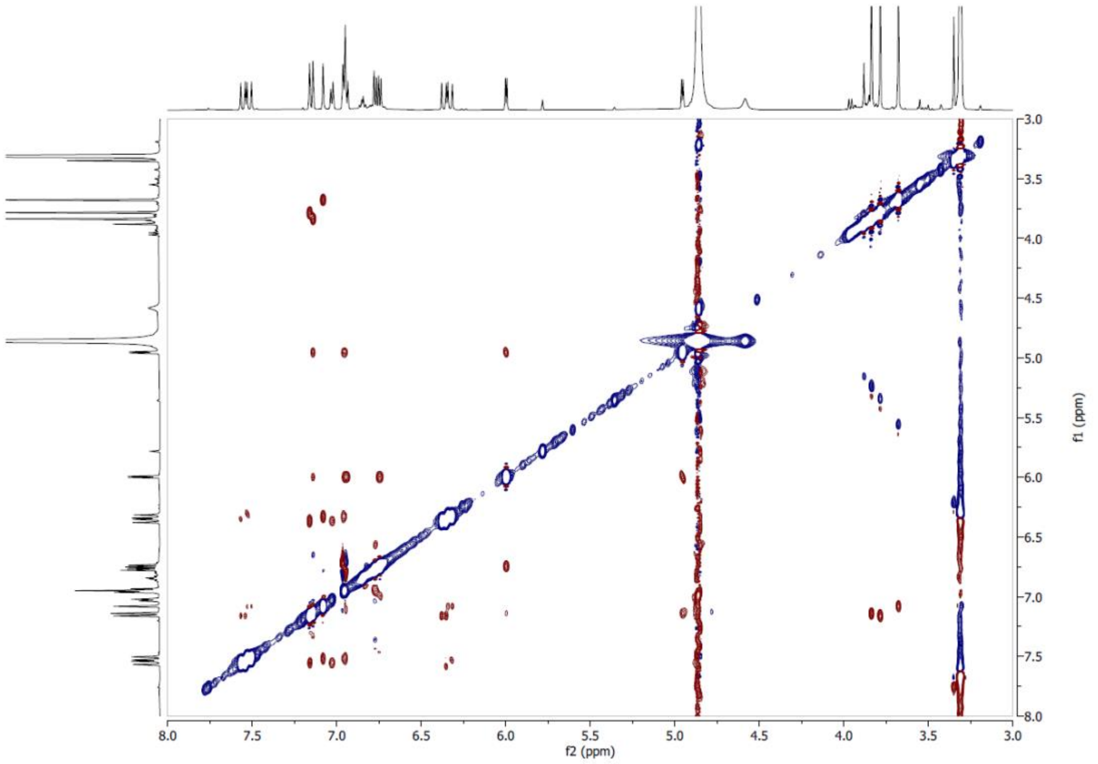

Supplementary Figure 65. ROESY NMR spectrum of compound **11** in DMSO-d_6

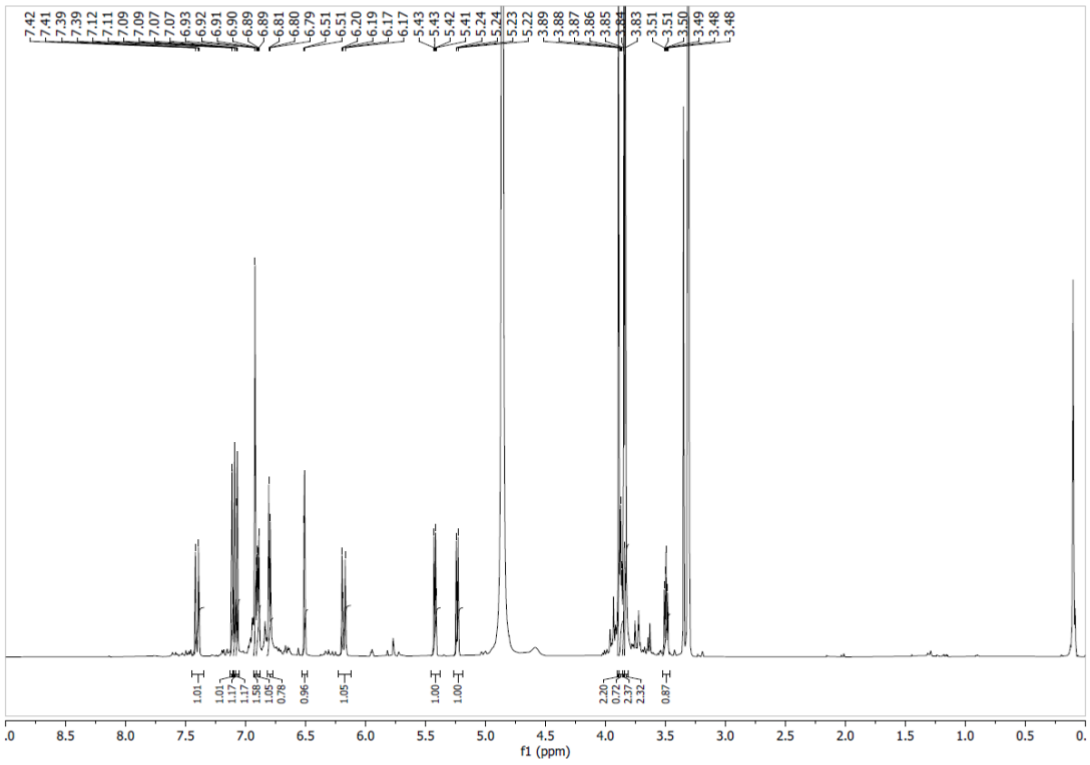

Supplementary Figure 66. ^{1}H NMR spectrum of compound **12** in DMSO-d_6 at 600 MHz

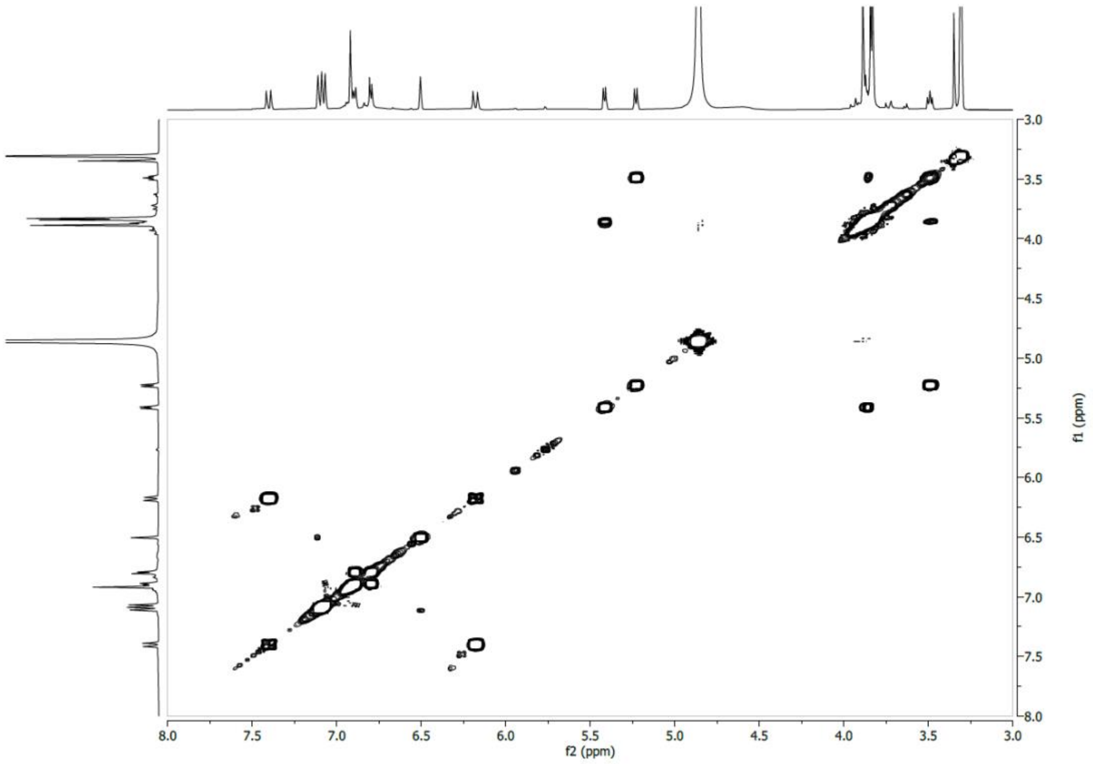

Supplementary Figure 67. COSY NMR spectrum of compound **12** in DMSO-d_6

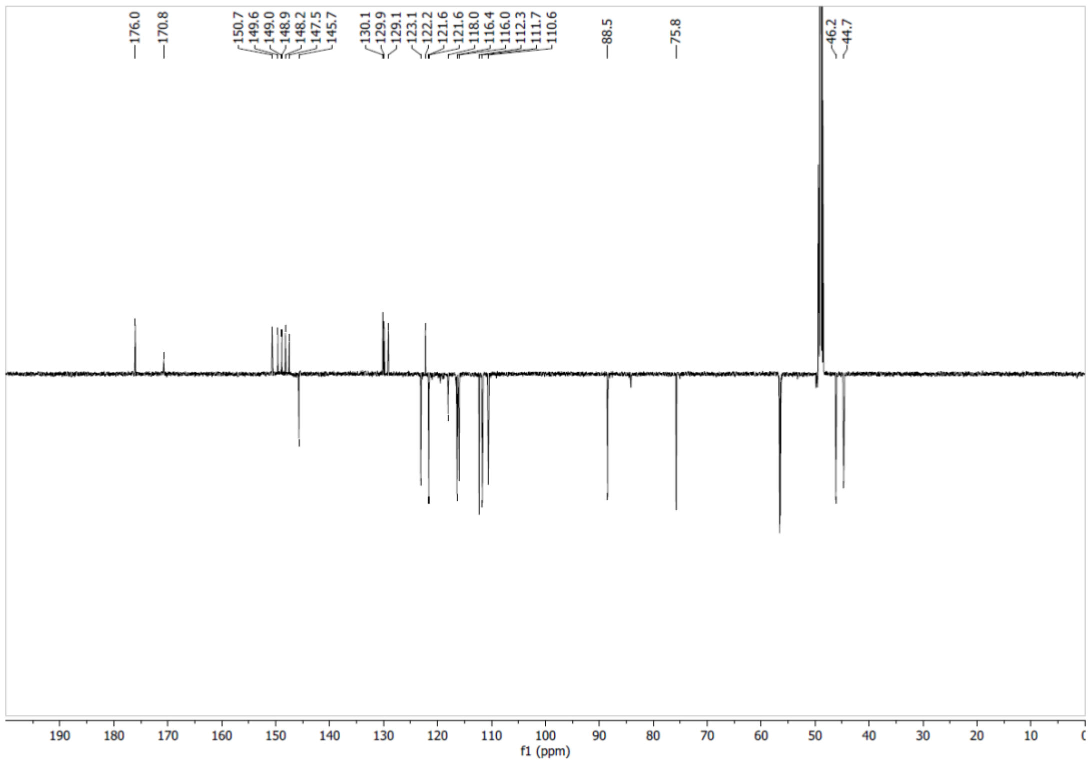

Supplementary Figure 68. ^{13}C-DEPTQ NMR spectrum of compound **12** in DMSO-d_6 at 151 MHz

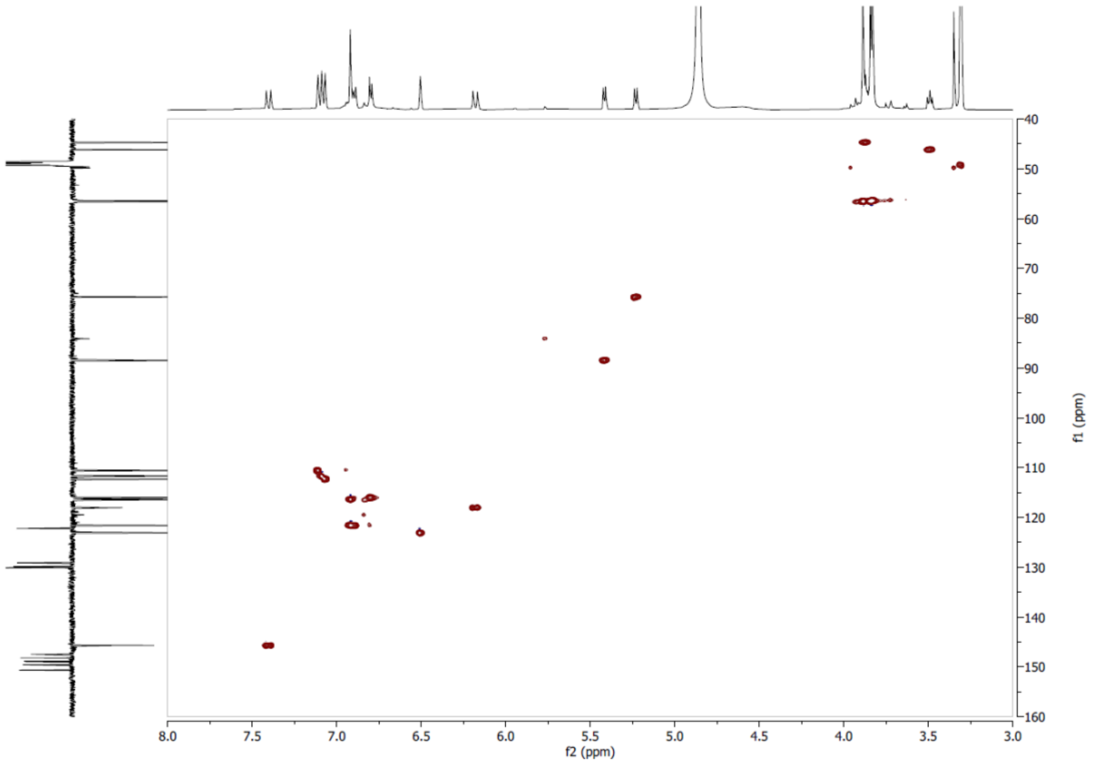

Supplementary Figure 69. Edited-HSQC NMR spectrum of compound **12** in DMSO-d_6

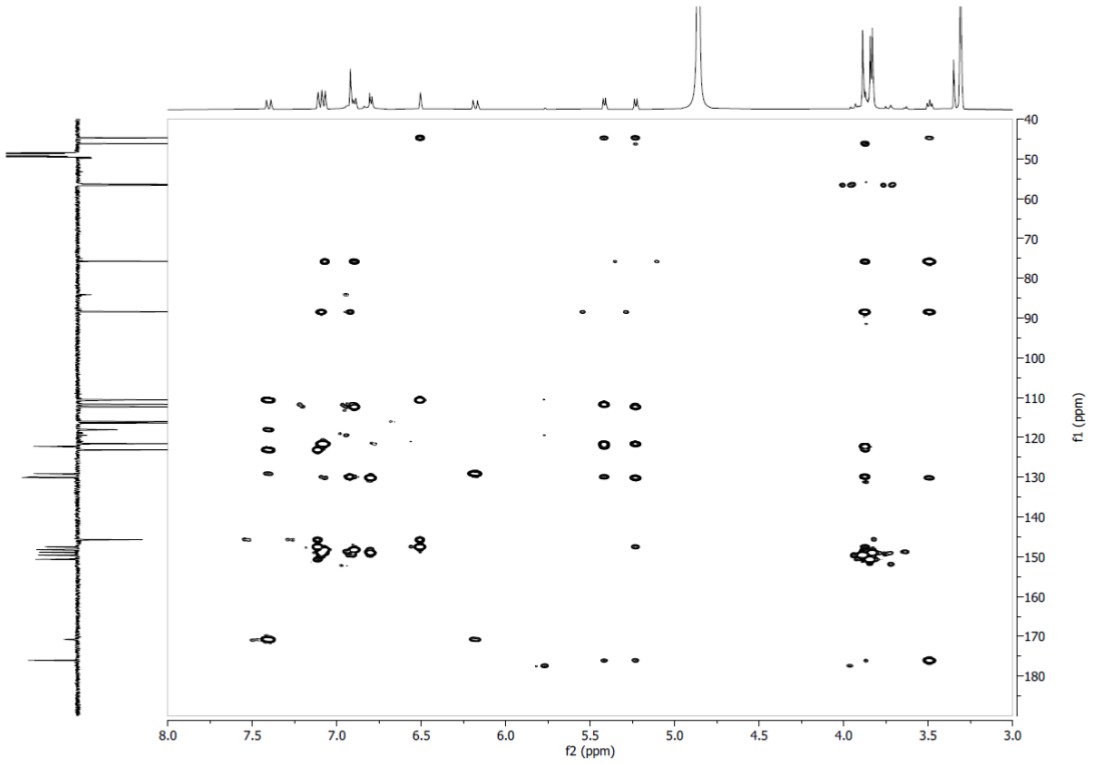

Supplementary Figure 70. HMBC NMR spectrum of compound **12** in DMSO-*d*6

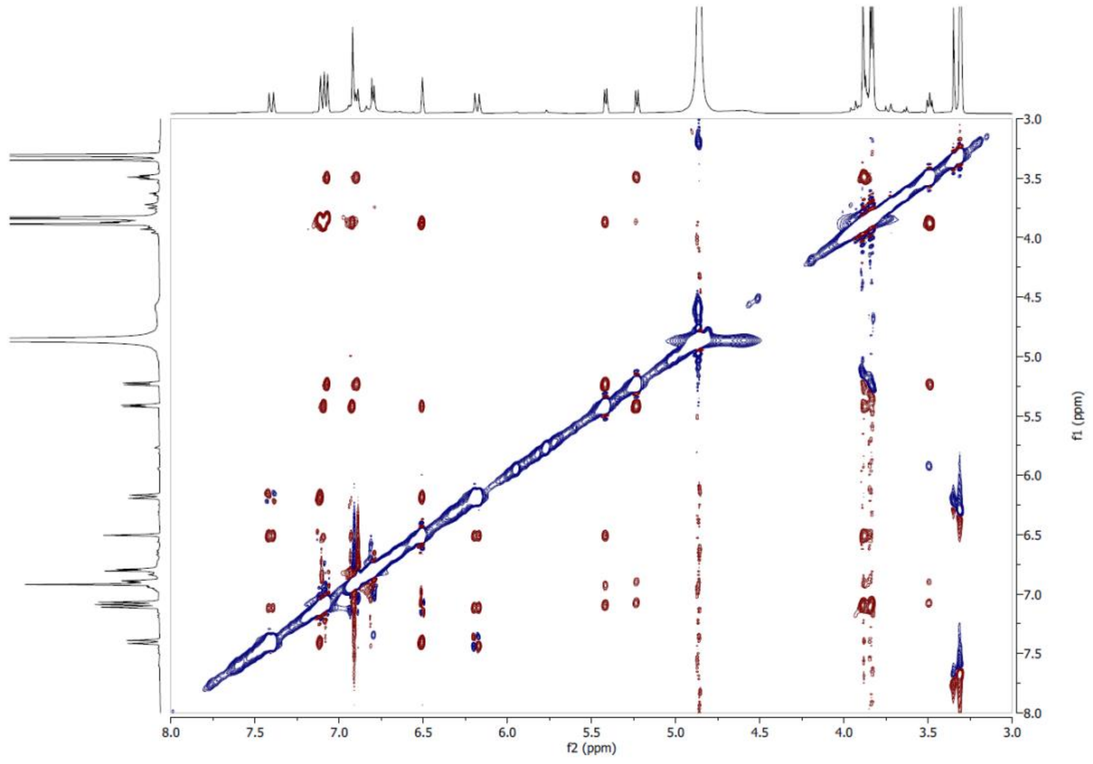

Supplementary Figure 71. ROESY NMR spectrum of compound **12** in DMSO-*d*6

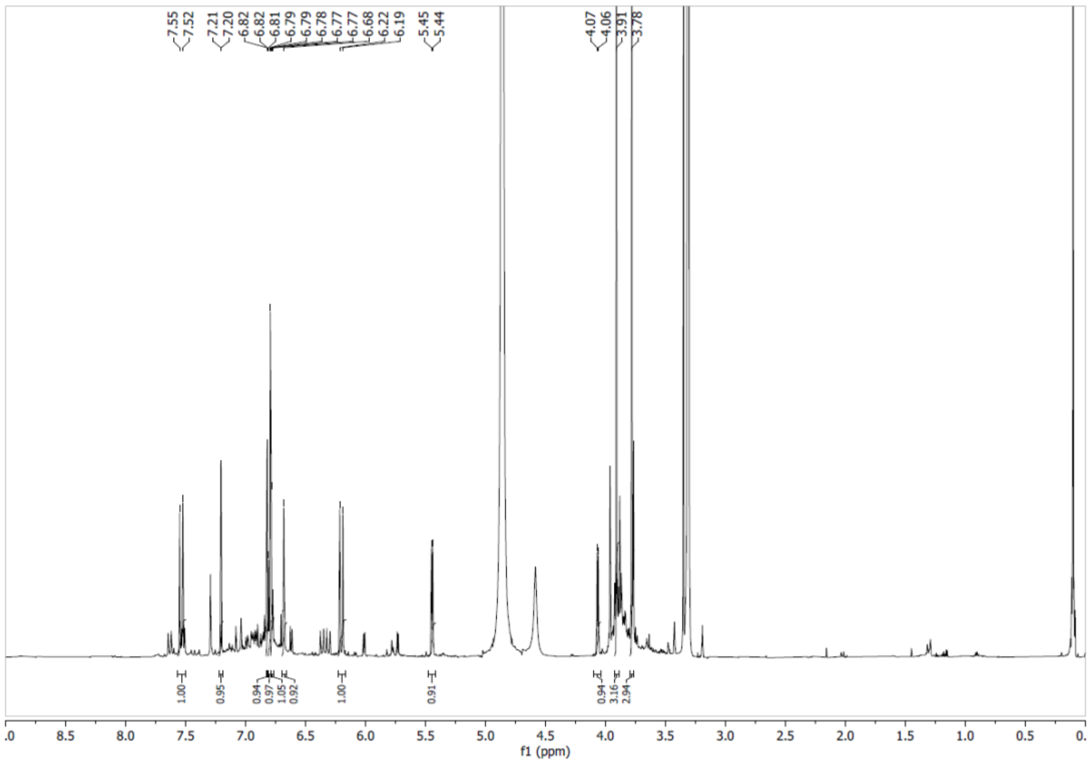

Supplementary Figure 72. ^{1}H NMR spectrum of compound **13** in DMSO-d_6 at 600 MHz

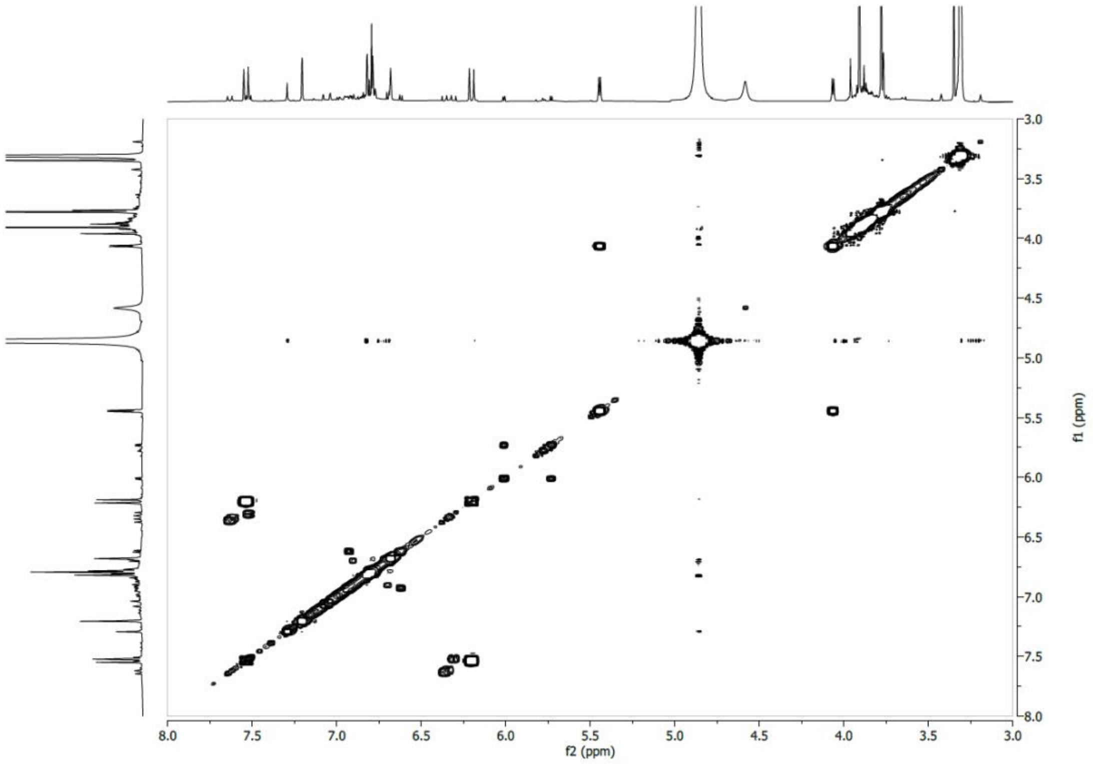

Supplementary Figure 73. COSY NMR spectrum of compound **13** in DMSO-d_6

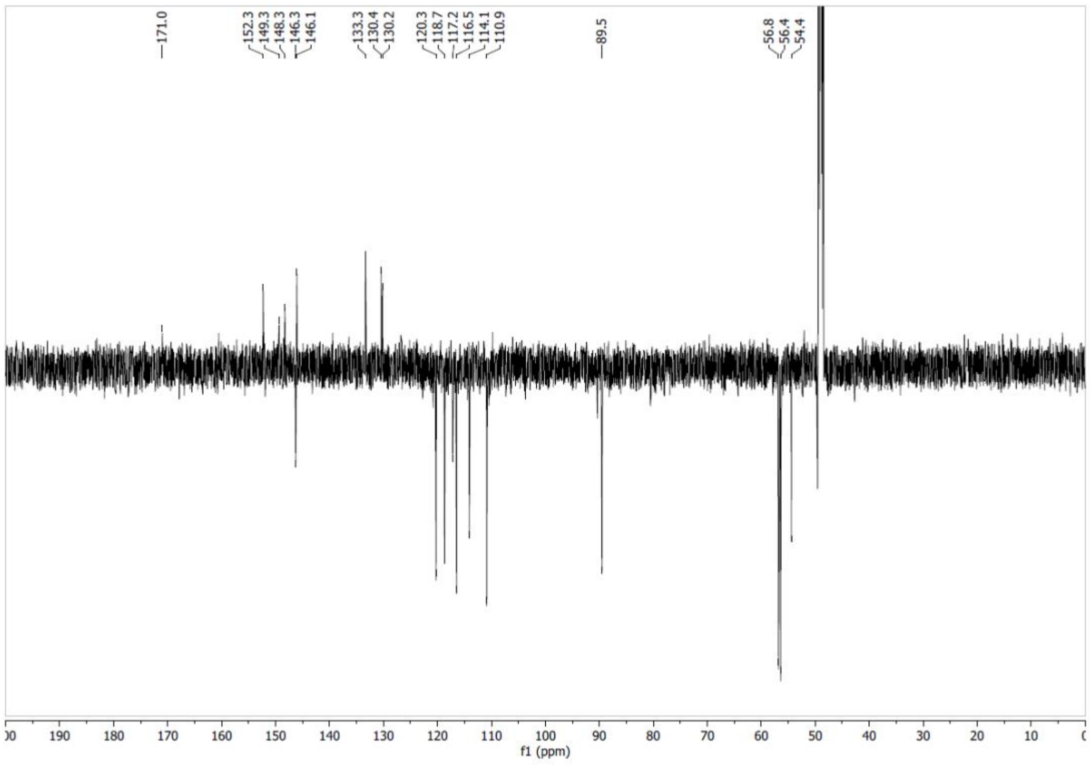

Supplementary Figure 74. ^{13}C-DEPTQ NMR spectrum of compound **13** in DMSO-d_6 at 151 MHz

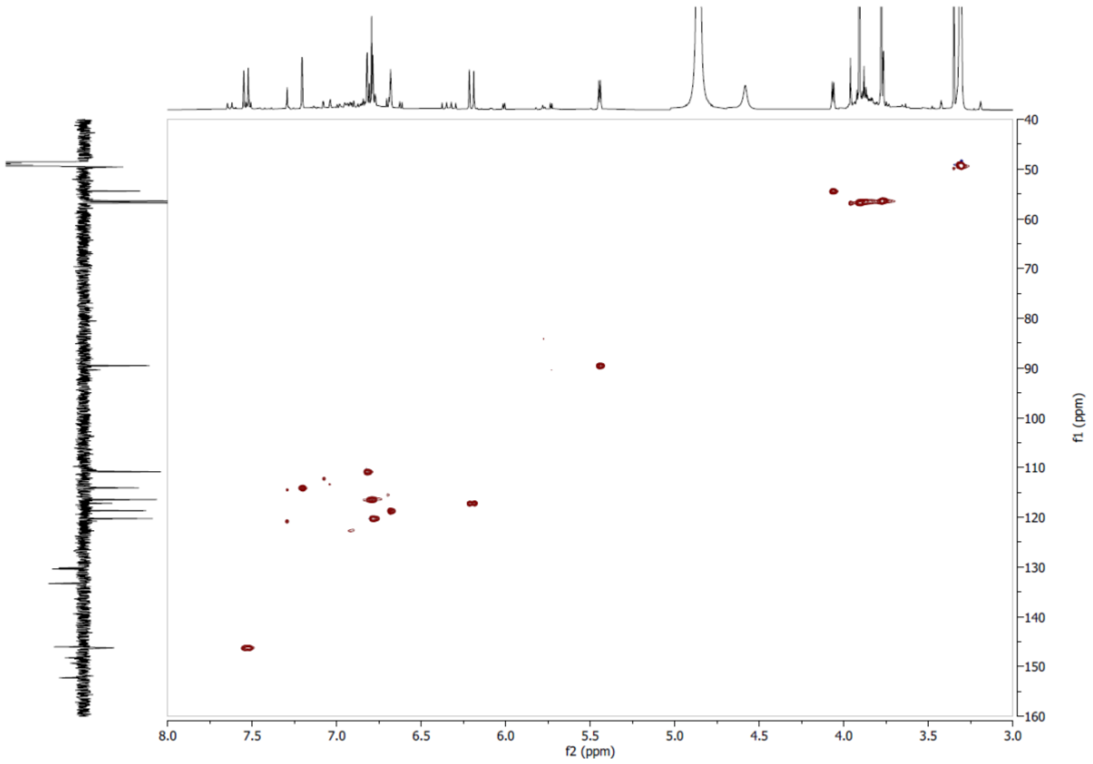

Supplementary Figure 75. Edited-HSQC NMR spectrum of compound **13** in DMSO-d_6

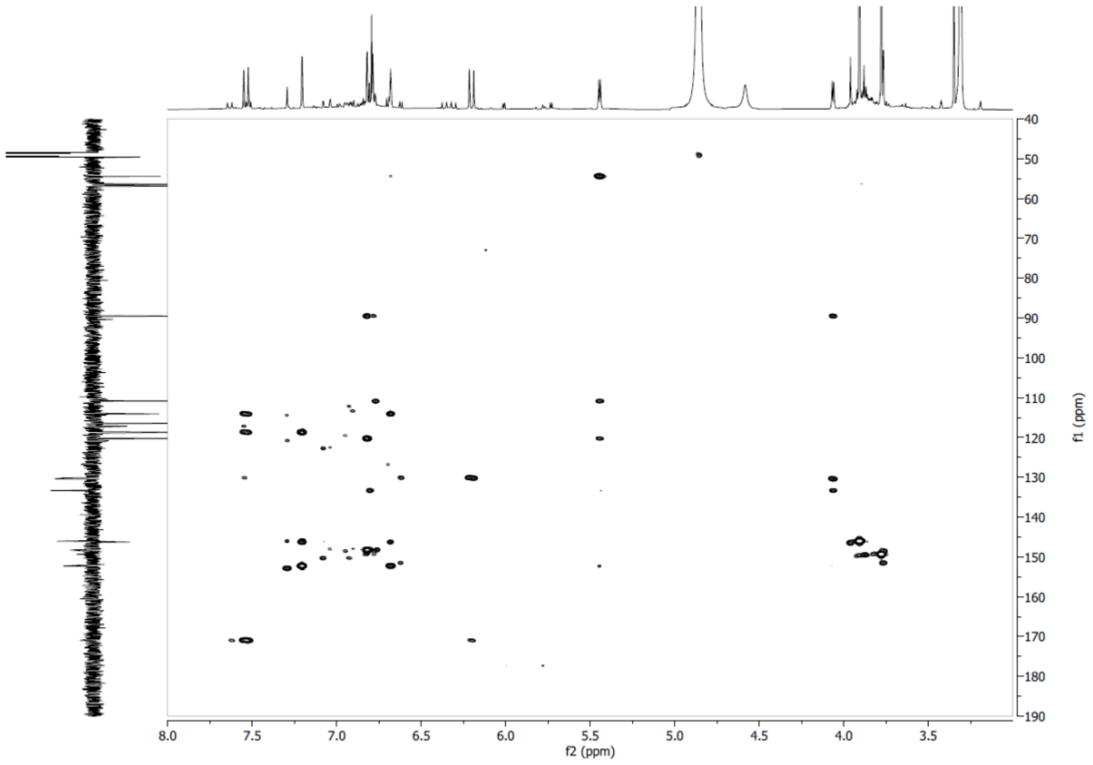

Supplementary Figure 76. HMBC NMR spectrum of compound **13** in DMSO-d_6

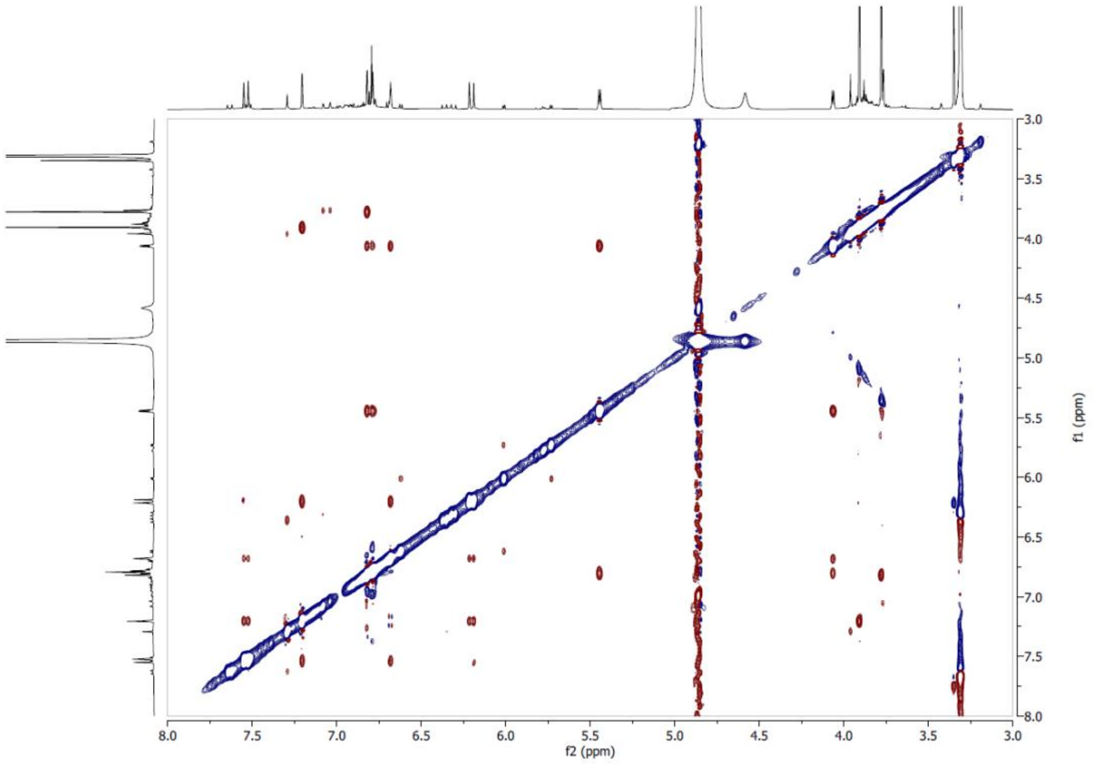

Supplementary Figure 77. ROESY NMR spectrum of compound **13** in DMSO-d_6

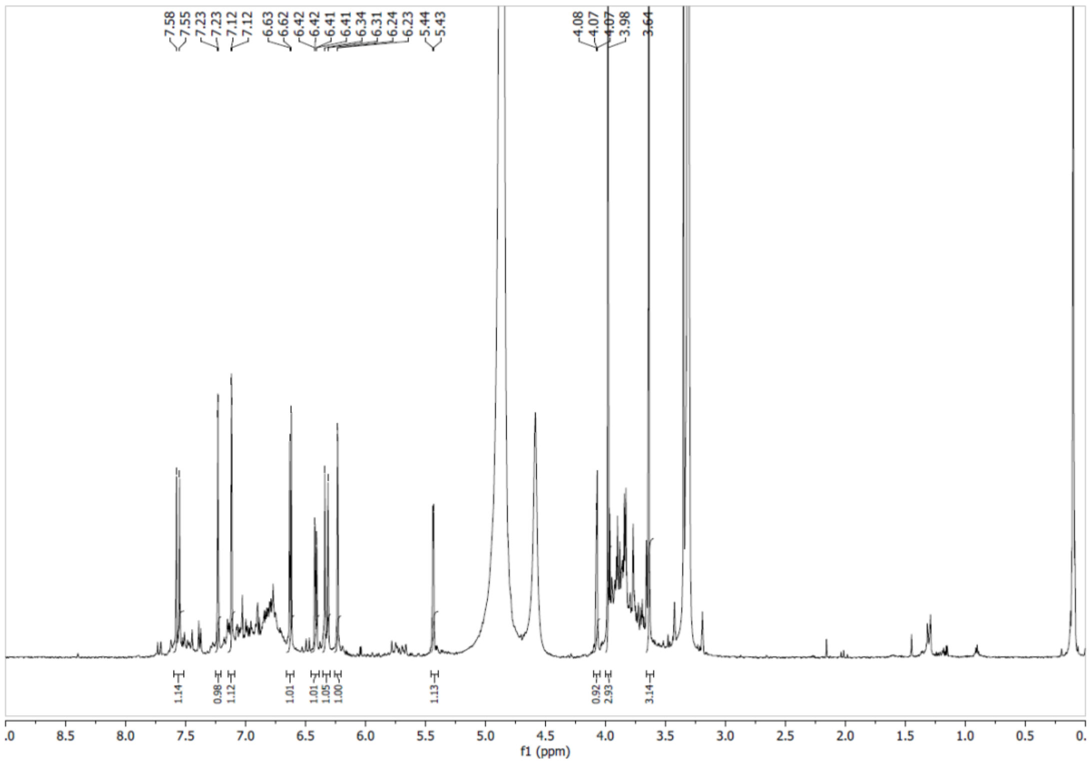

Supplementary Figure 78. ^{1}H NMR spectrum of compound **14** in DMSO-d_6 at 600 MHz

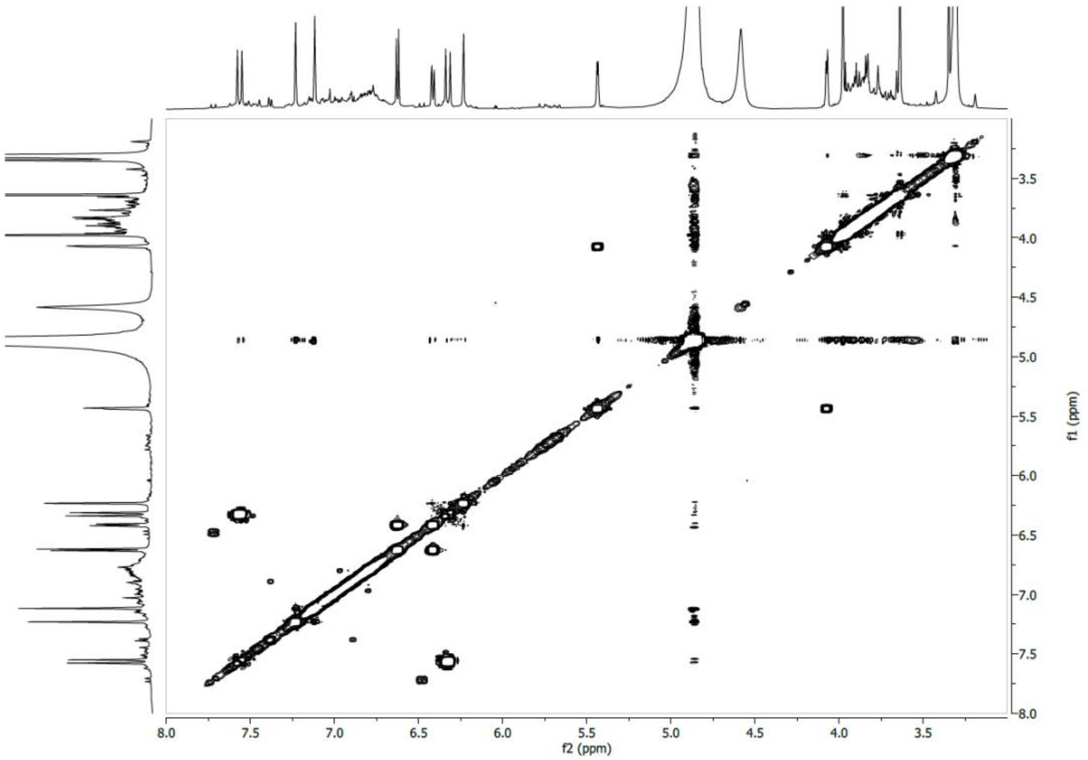

Supplementary Figure 79. COSY NMR spectrum of compound **14** in DMSO-d_6

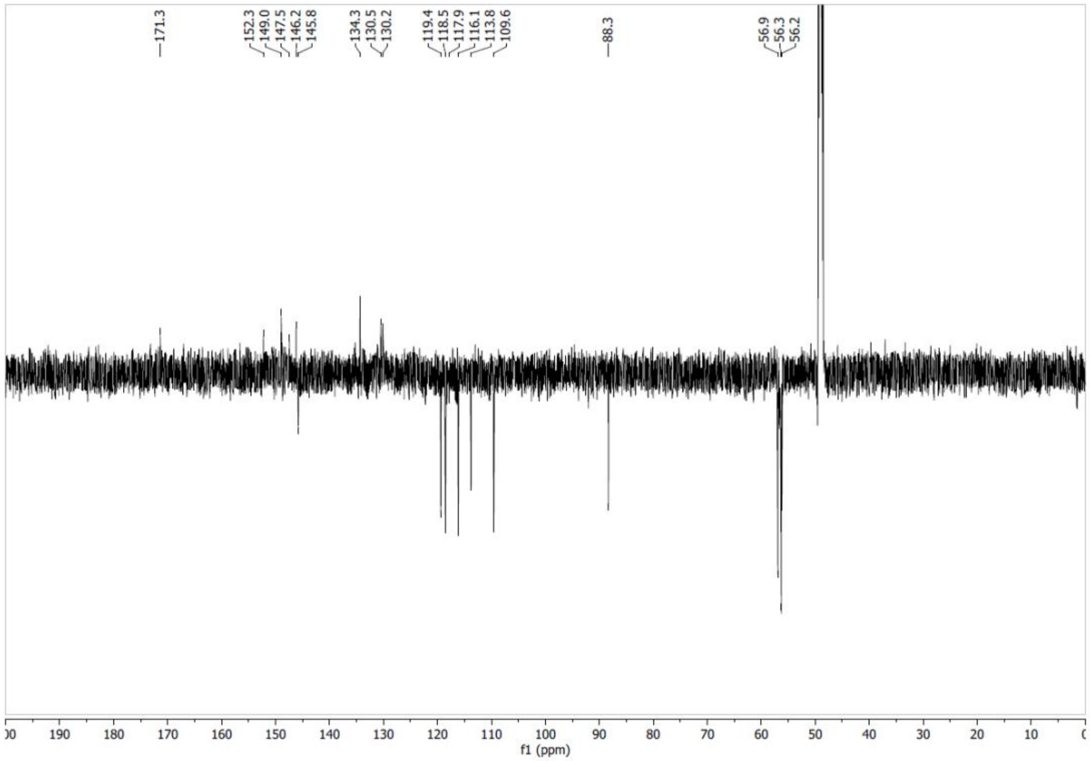

Supplementary Figure 80. ^{13}C-DEPTQ NMR spectrum of compound **14** in DMSO-d_6 at 151 MHz

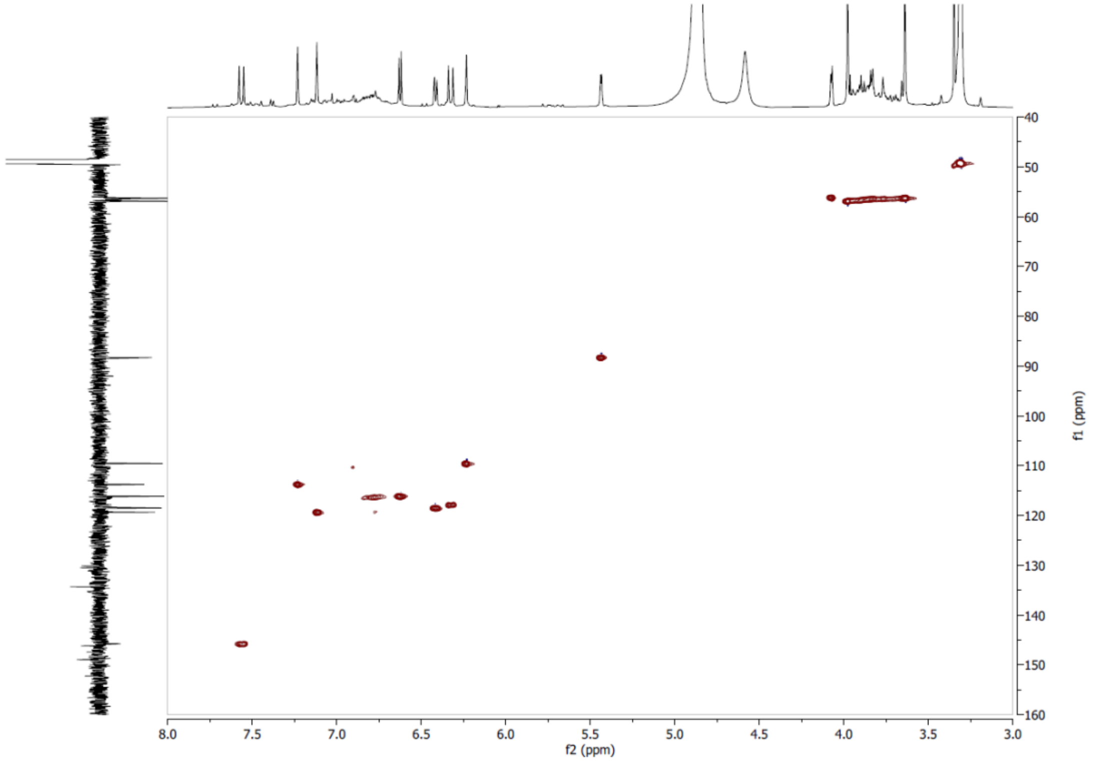

Supplementary Figure 81. Edited-HSQC NMR spectrum of compound **14** in DMSO-d_6

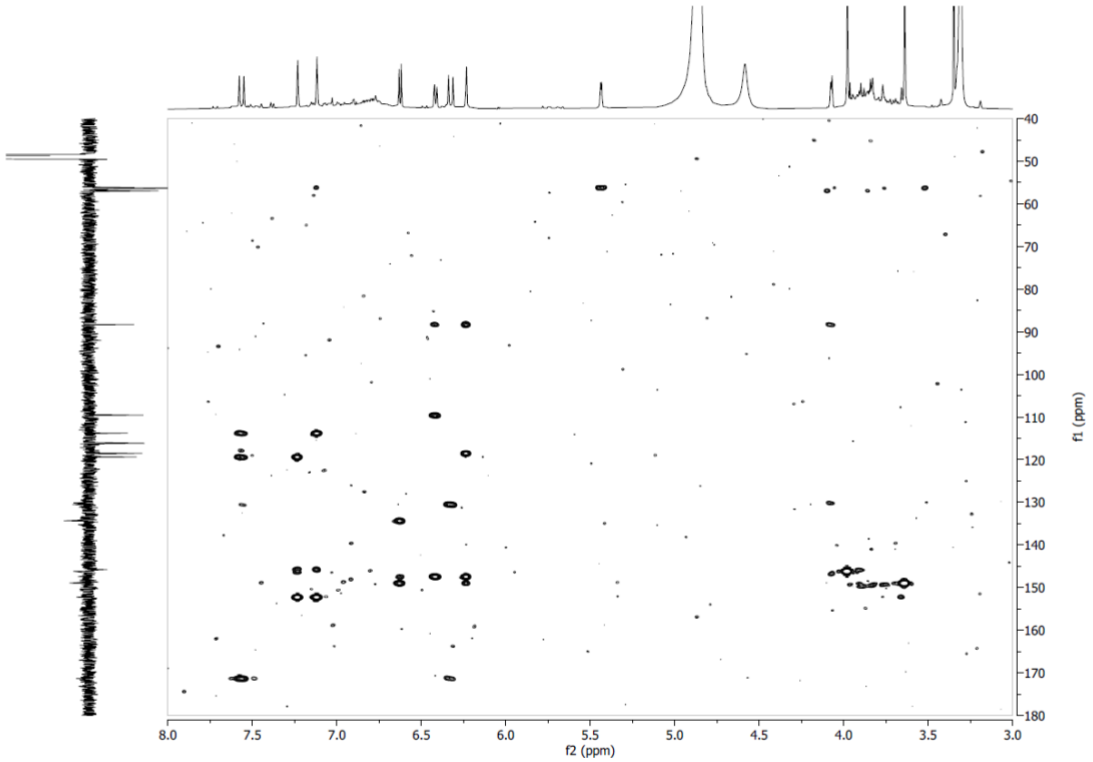

Supplementary Figure 82. HMBC NMR spectrum of compound **14** in DMSO-d_6

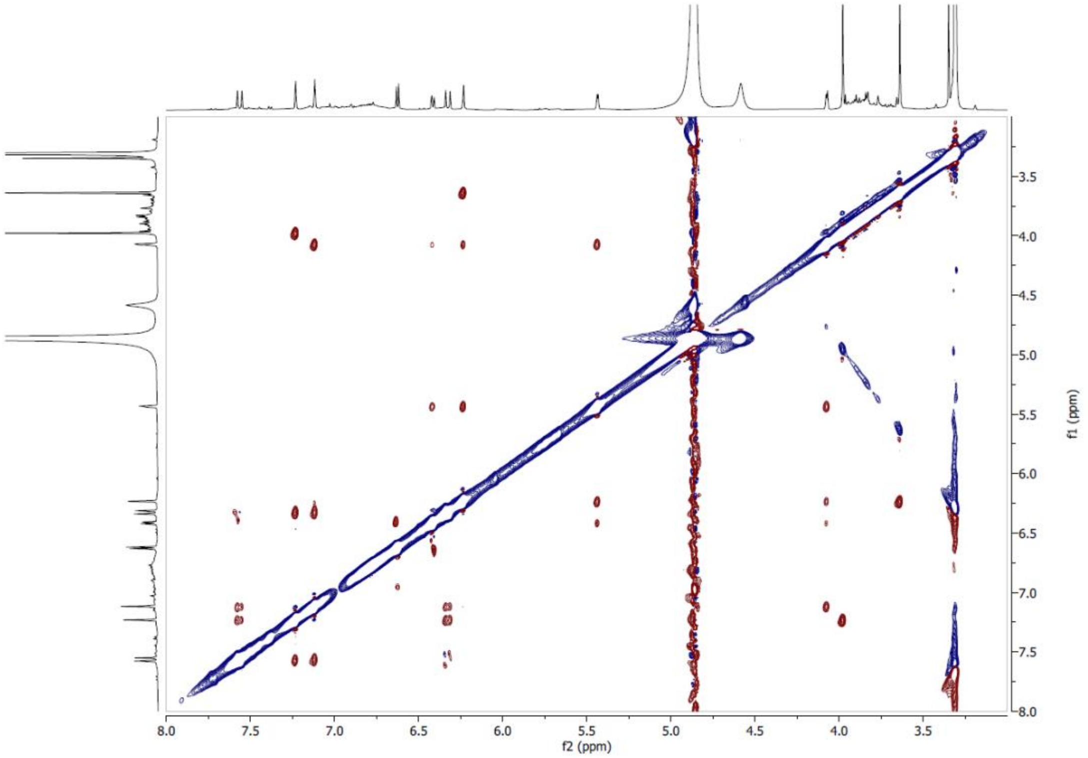

Supplementary Figure 83. ROESY NMR spectrum of compound **14** in DMSO-d_6

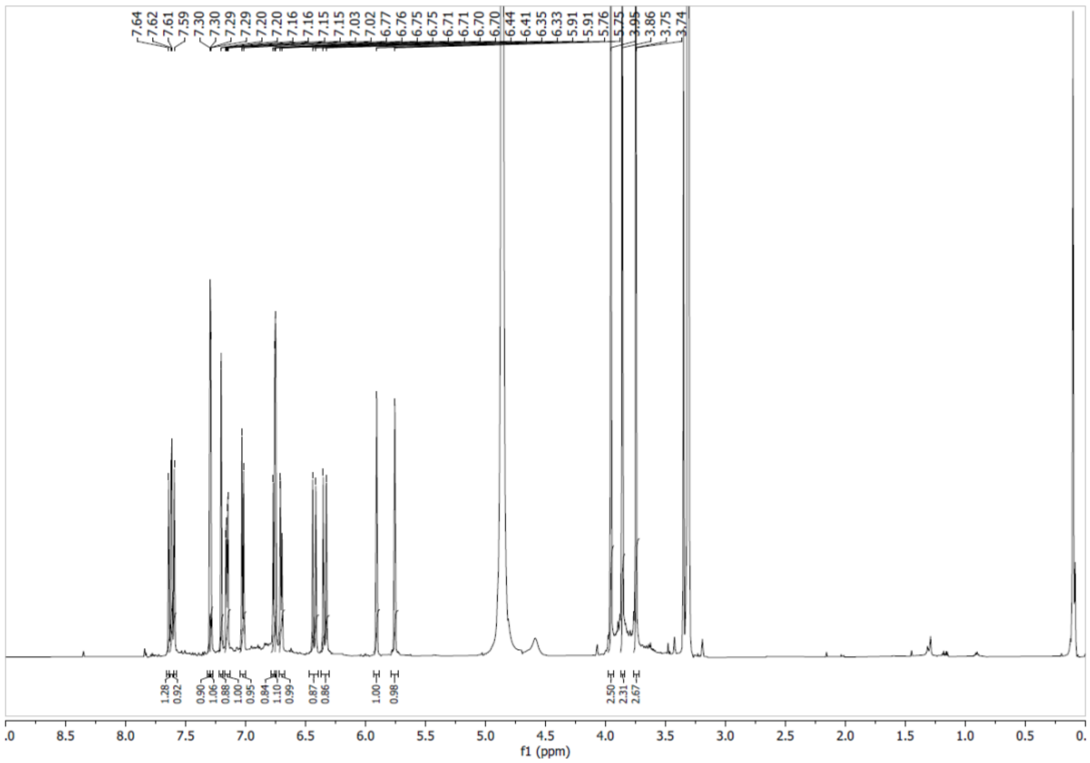

Supplementary Figure 84. ^{1}H NMR spectrum of compound **15** in DMSO-d_6 at 600 MHz

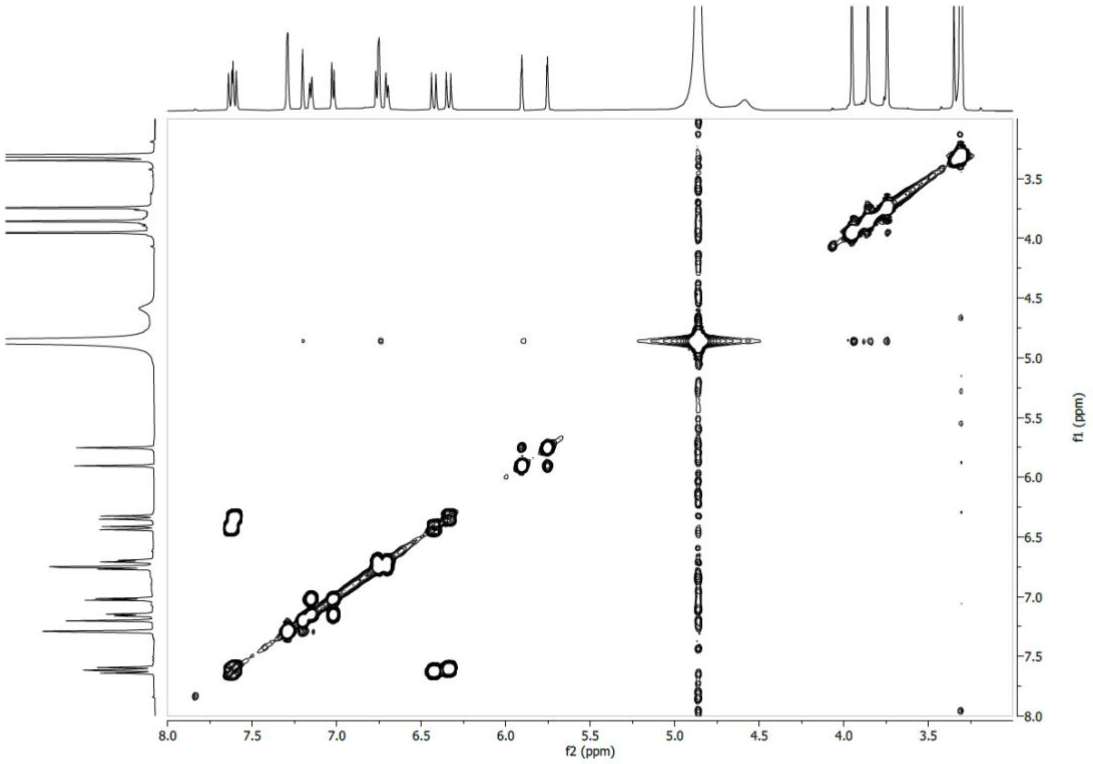

Supplementary Figure 85. COSY NMR spectrum of compound **15** in DMSO-d_6

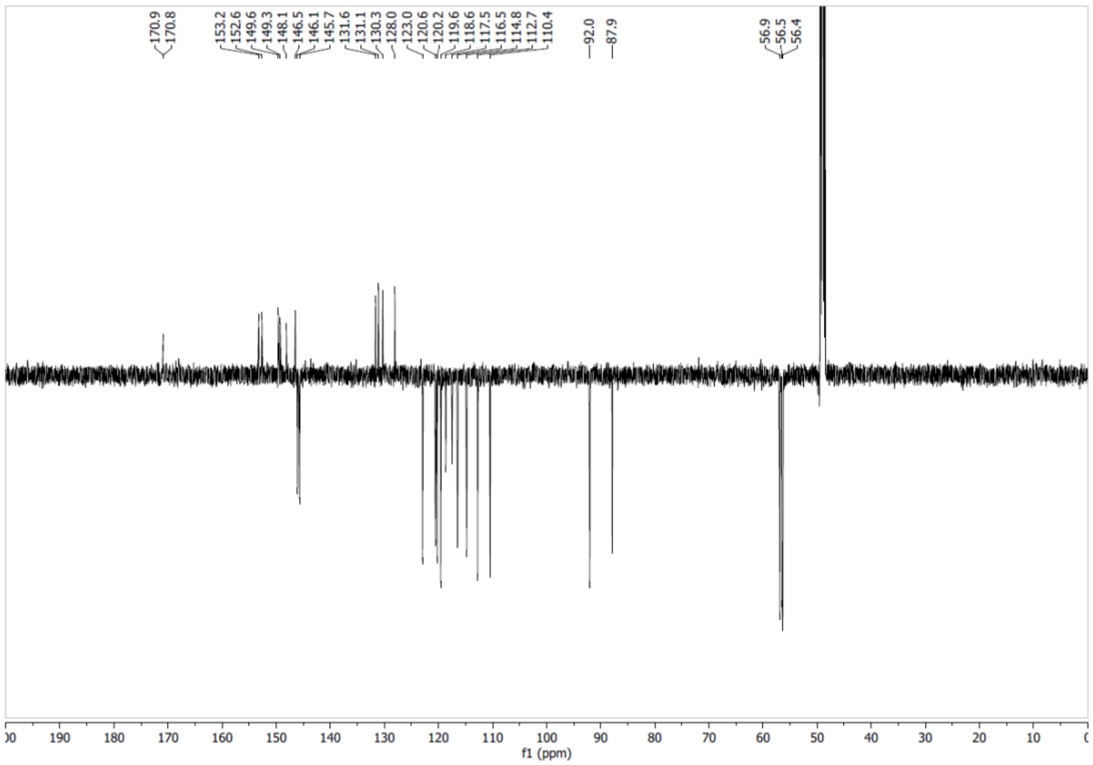

Supplementary Figure 86. ^{13}C-DEPTQ NMR spectrum of compound **15** in DMSO-d_6 at 151 MHz

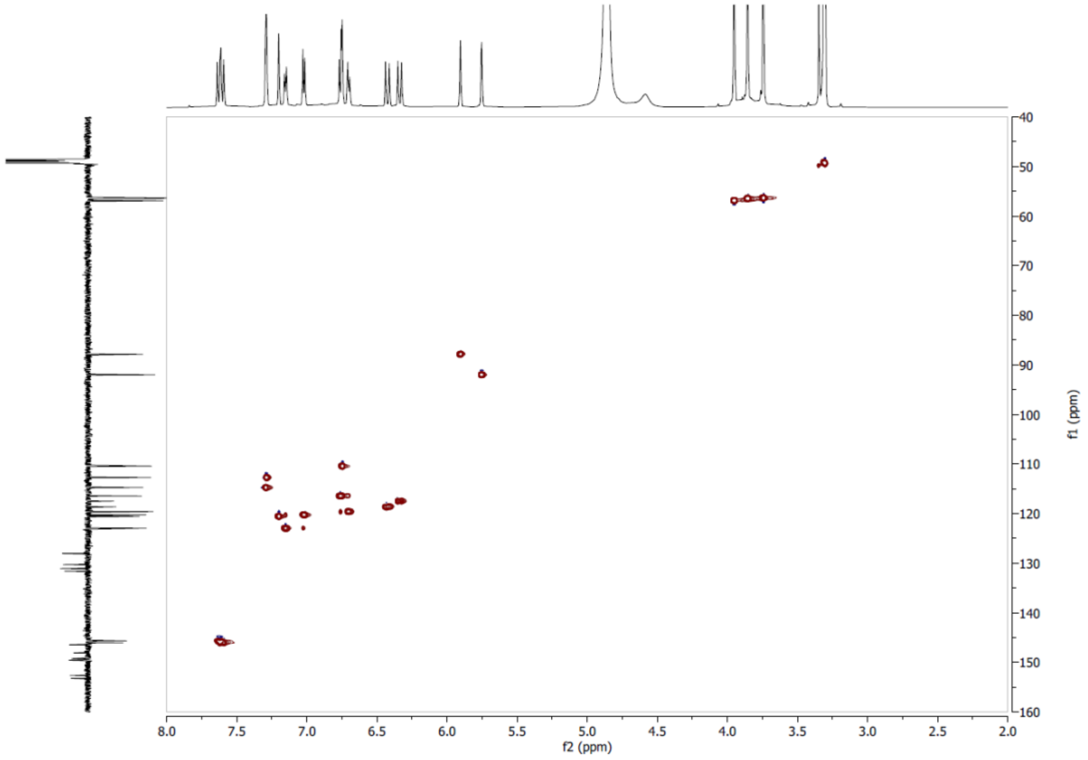

Supplementary Figure 87. Edited-HSQC NMR spectrum of compound **15** in DMSO-d_6

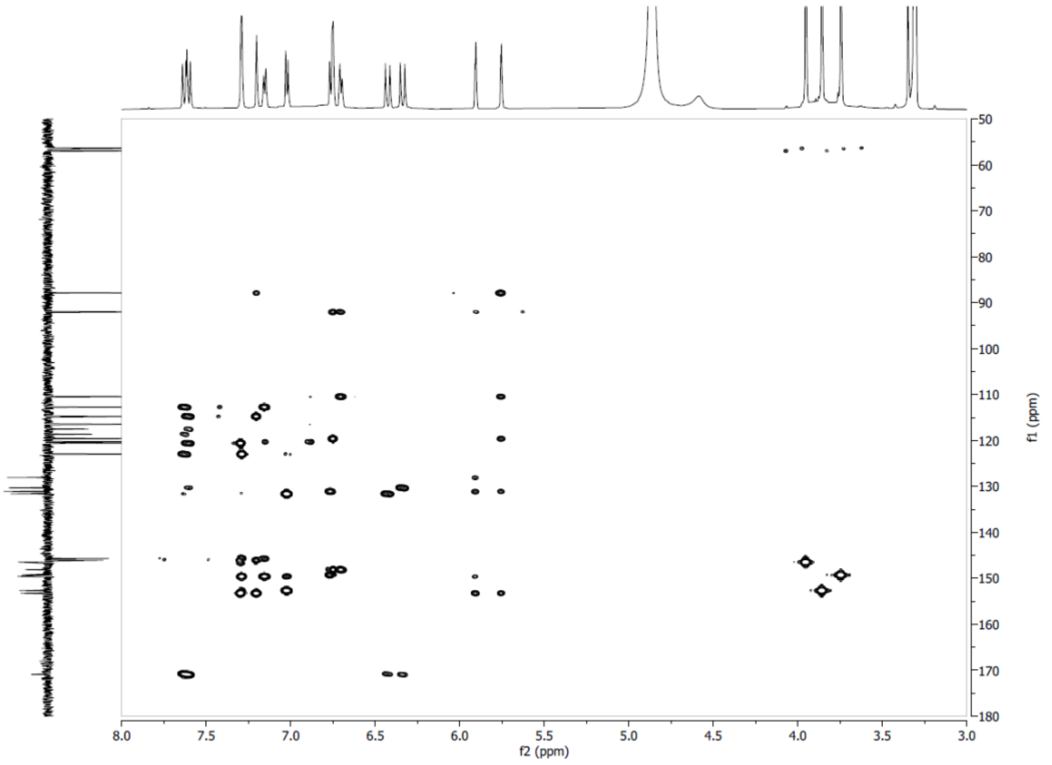

Supplementary Figure 88. HMBC NMR spectrum of compound **15** in DMSO-d_6

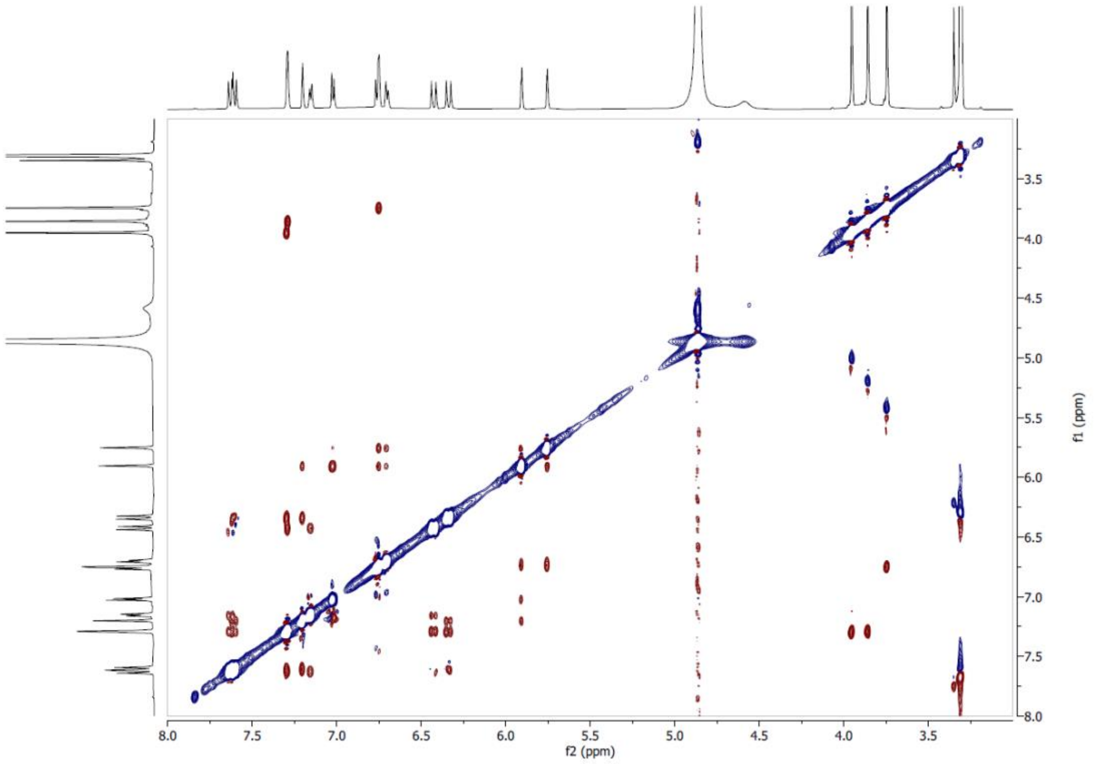

Supplementary Figure 89. ROESY NMR spectrum of compound **15** in DMSO-d_6

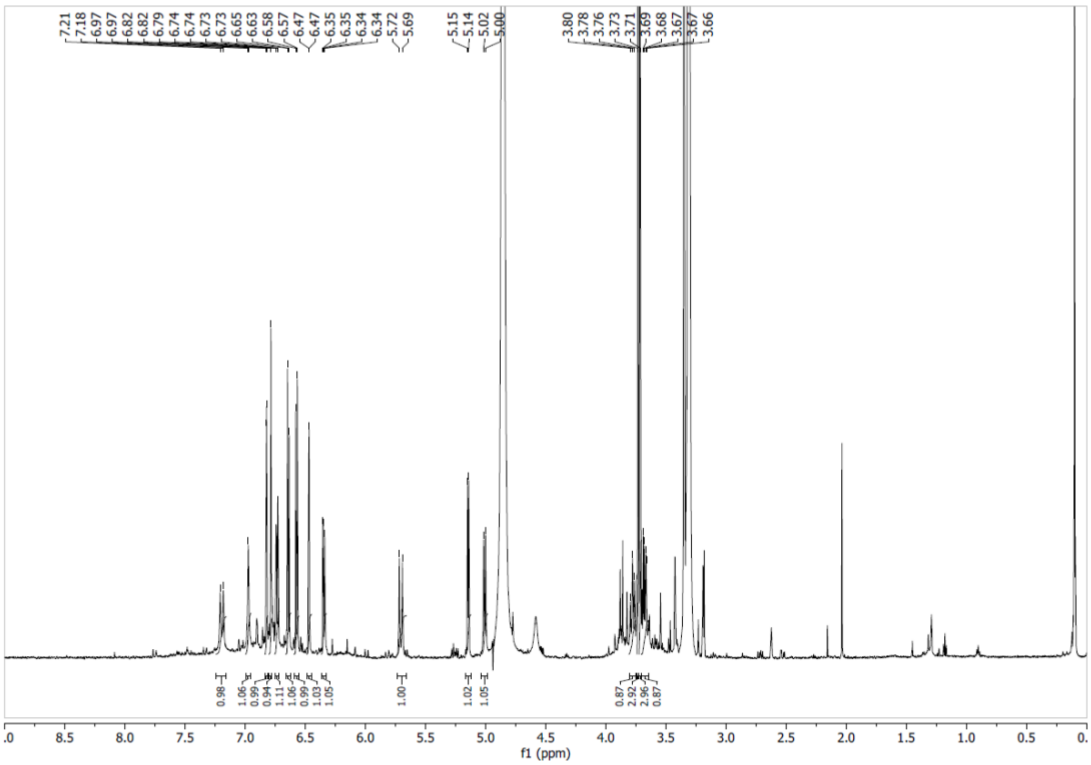

Supplementary Figure 90. ^{1}H NMR spectrum of compound **16** in DMSO-d_6 at 600 MHz

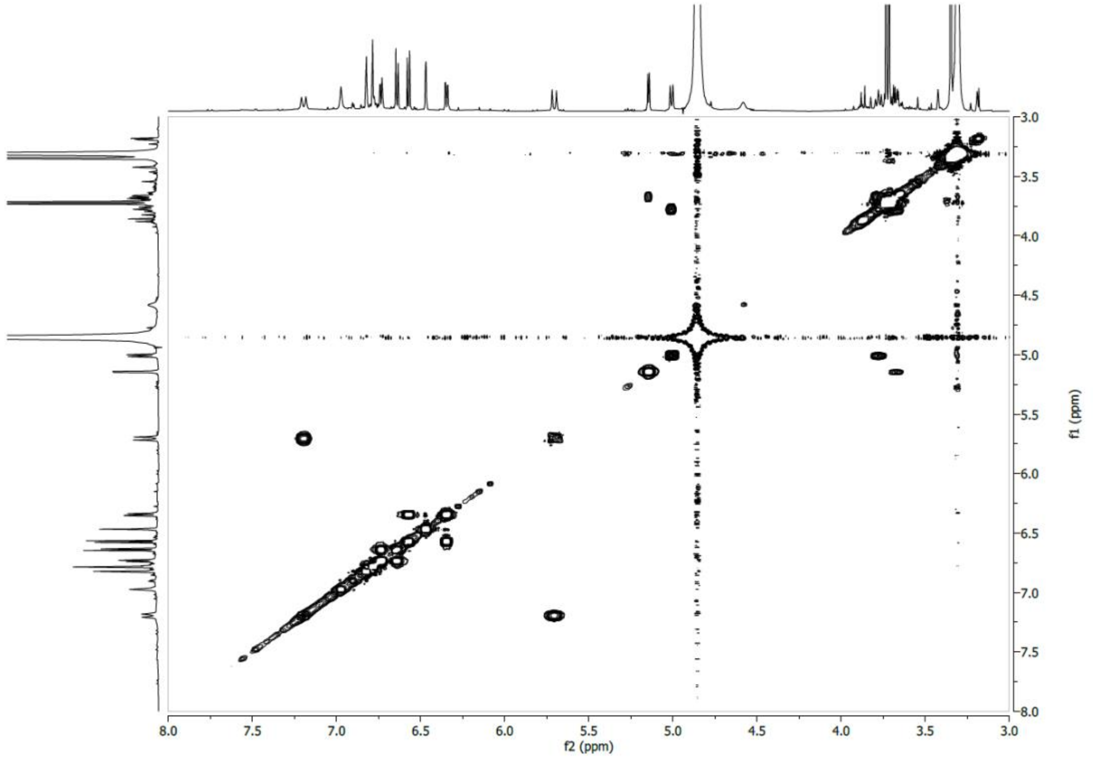

Supplementary Figure 91. COSY NMR spectrum of compound **16** in DMSO-d_6

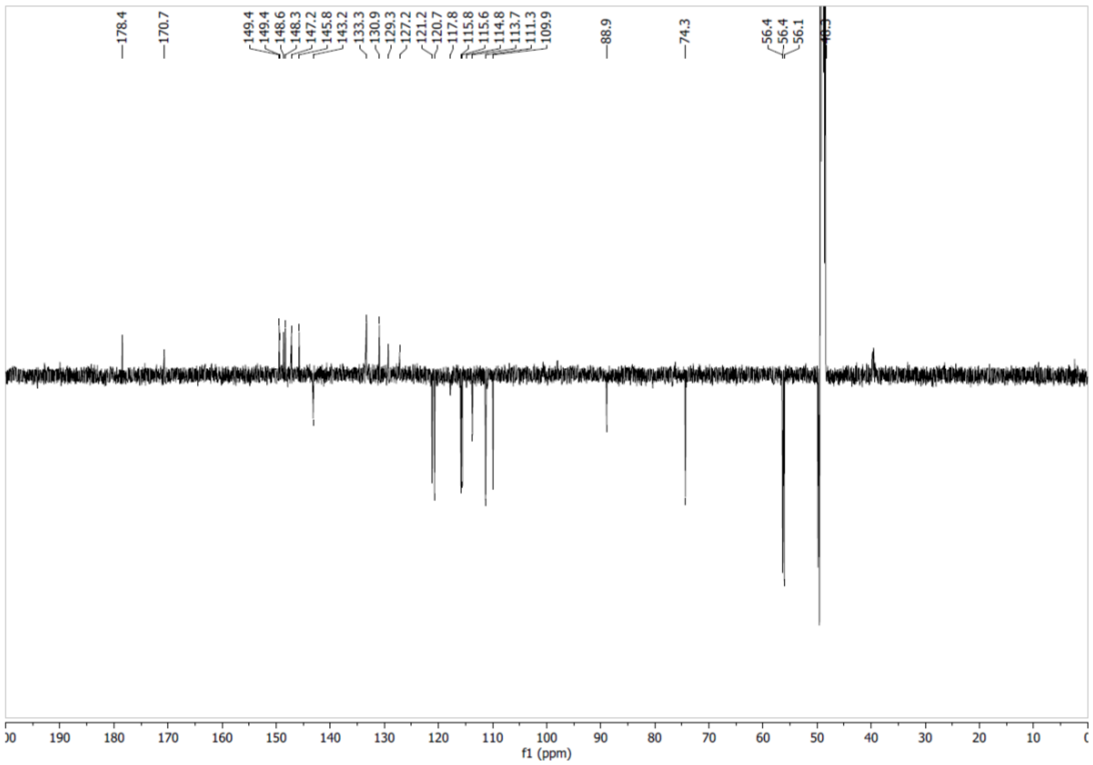

Supplementary Figure 92. ^{13}C-DEPTQ NMR spectrum of compound **16** in DMSO-d_6 at 151 MHz

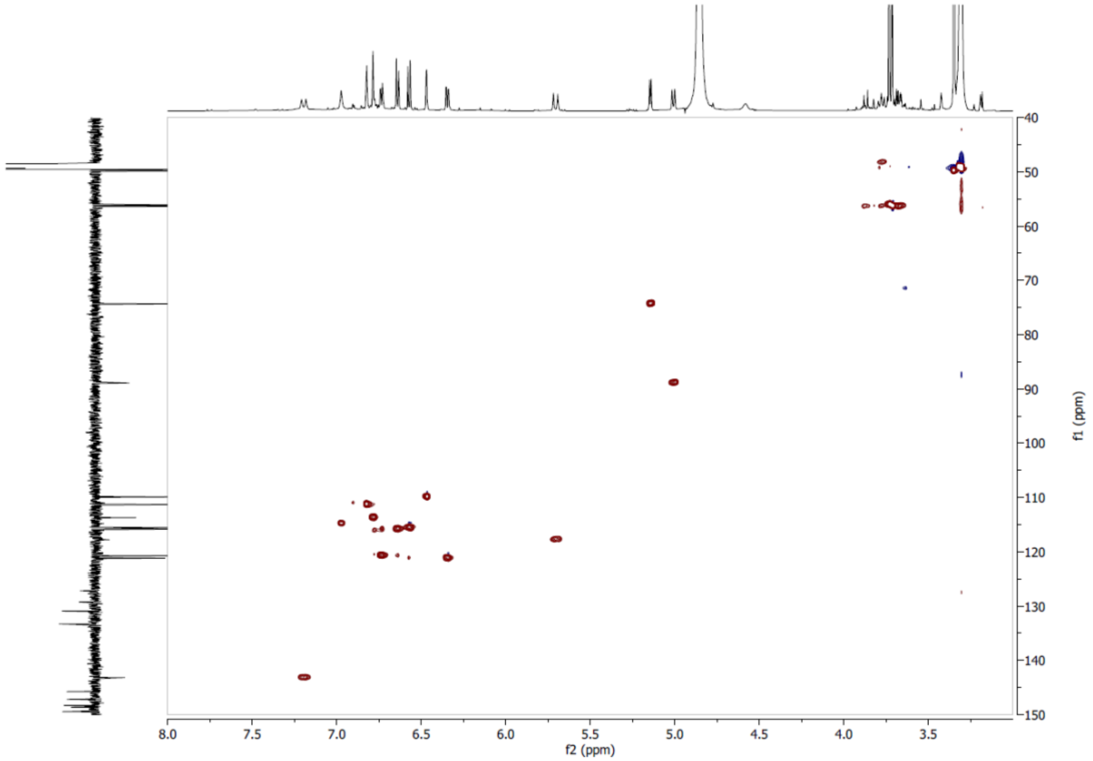

Supplementary Figure 93. Edited-HSQC NMR spectrum of compound **16** in DMSO-d_6

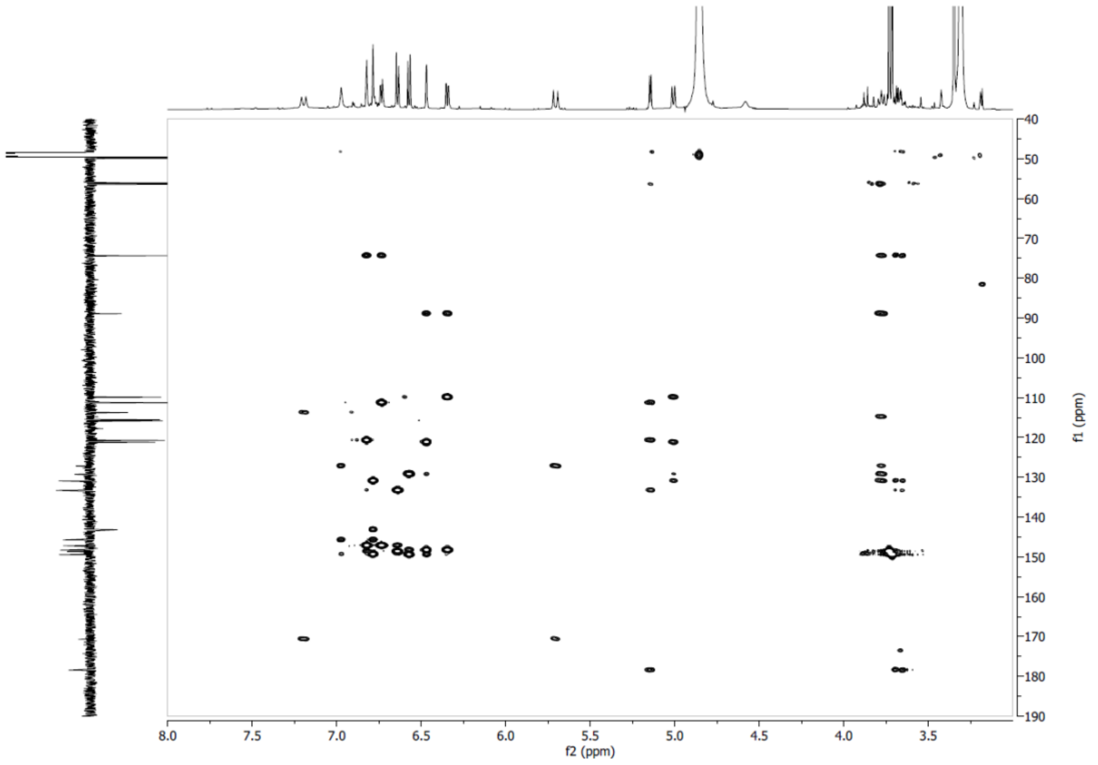

Supplementary Figure 94. HMBC NMR spectrum of compound **16** in DMSO-*d*6

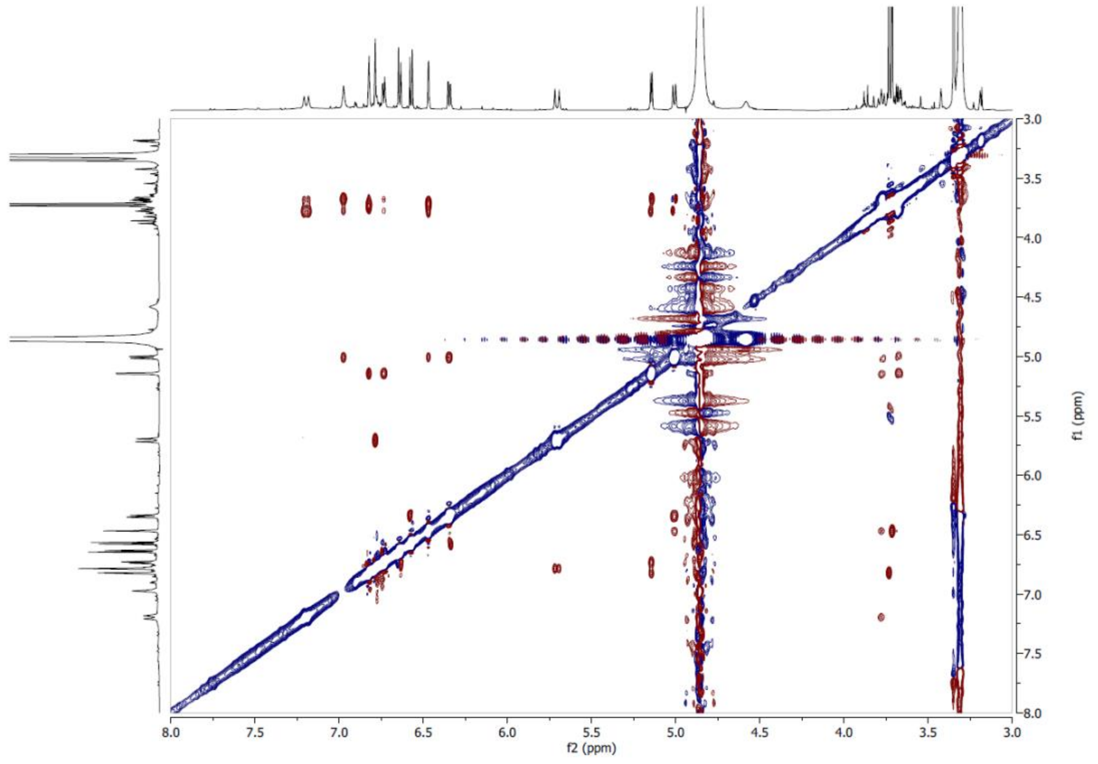

Supplementary Figure 95. ROESY NMR spectrum of compound **16** in DMSO-*d*6

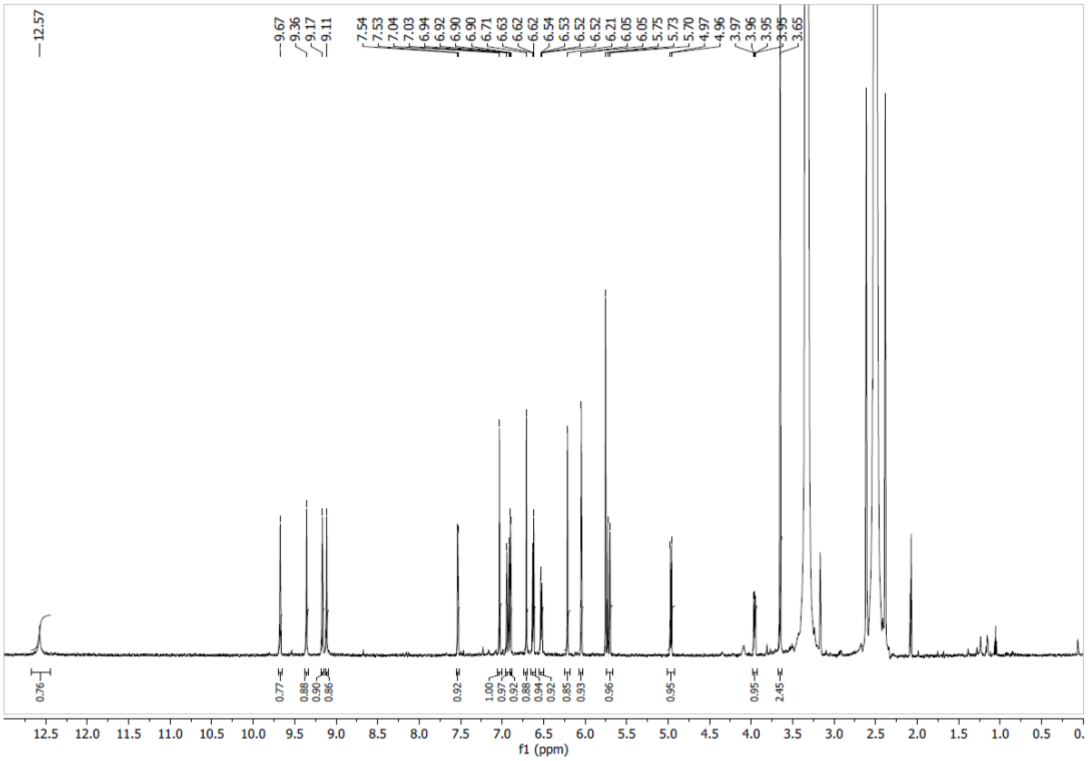

Supplementary Figure 96. ^{1}H NMR spectrum of compound **17** in DMSO-d_6 at 600 MHz

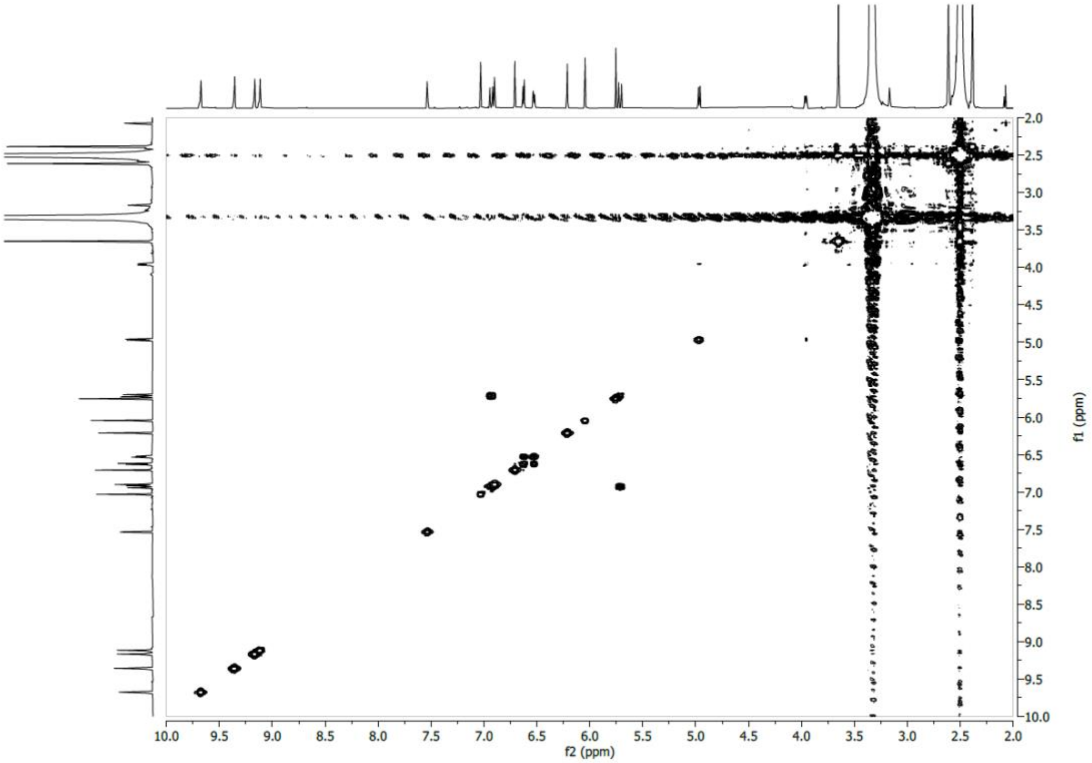

Supplementary Figure 97. COSY NMR spectrum of compound **17** in DMSO-d_6

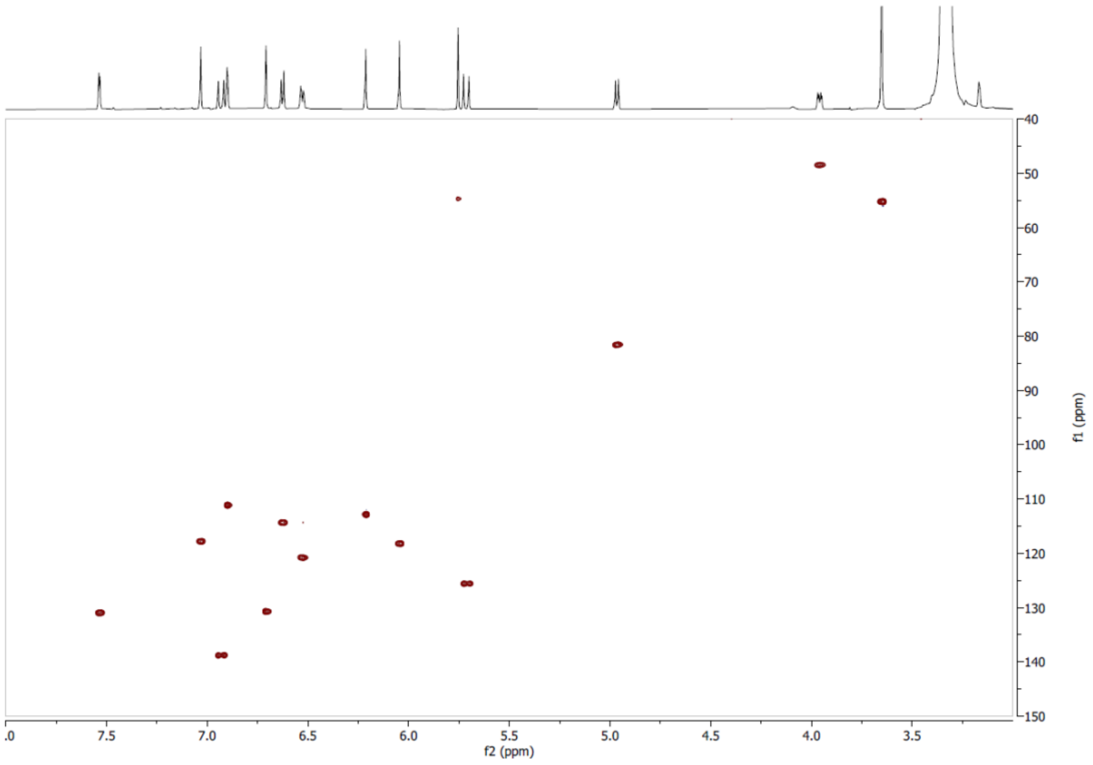

Supplementary Figure 98. Edited-HSQC NMR spectrum of compound **17** in DMSO-d_6

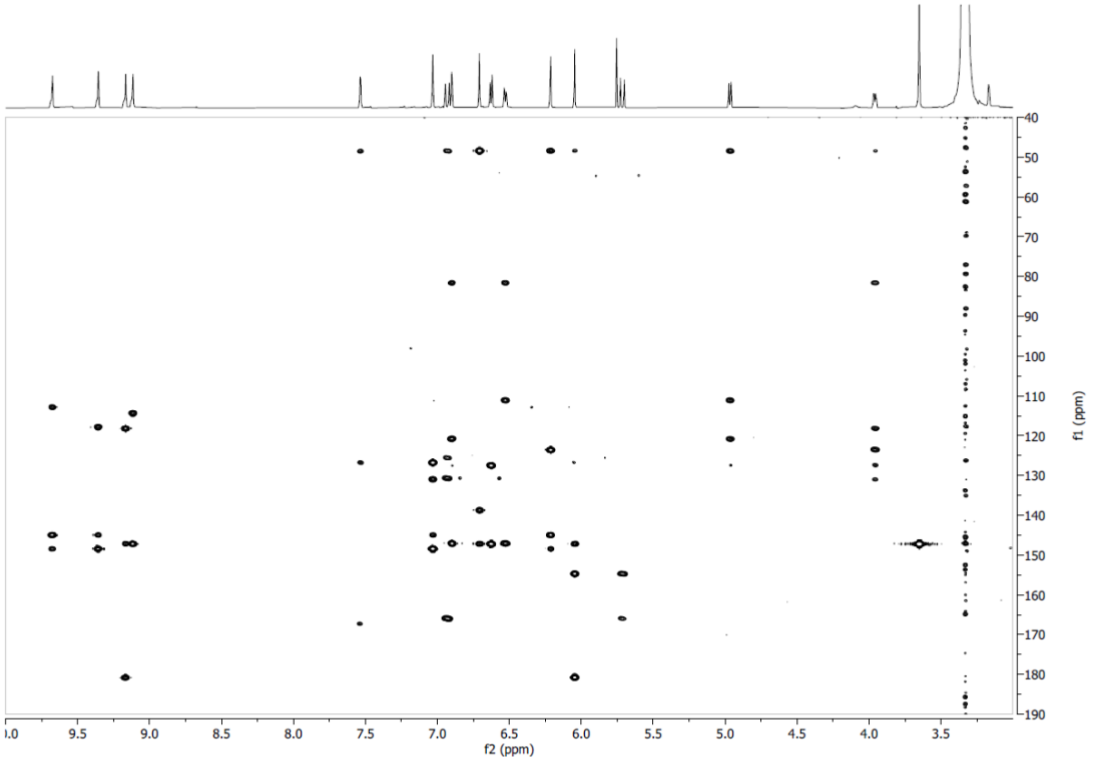

Supplementary Figure 99. HMBC NMR spectrum of compound **17** in DMSO-d_6

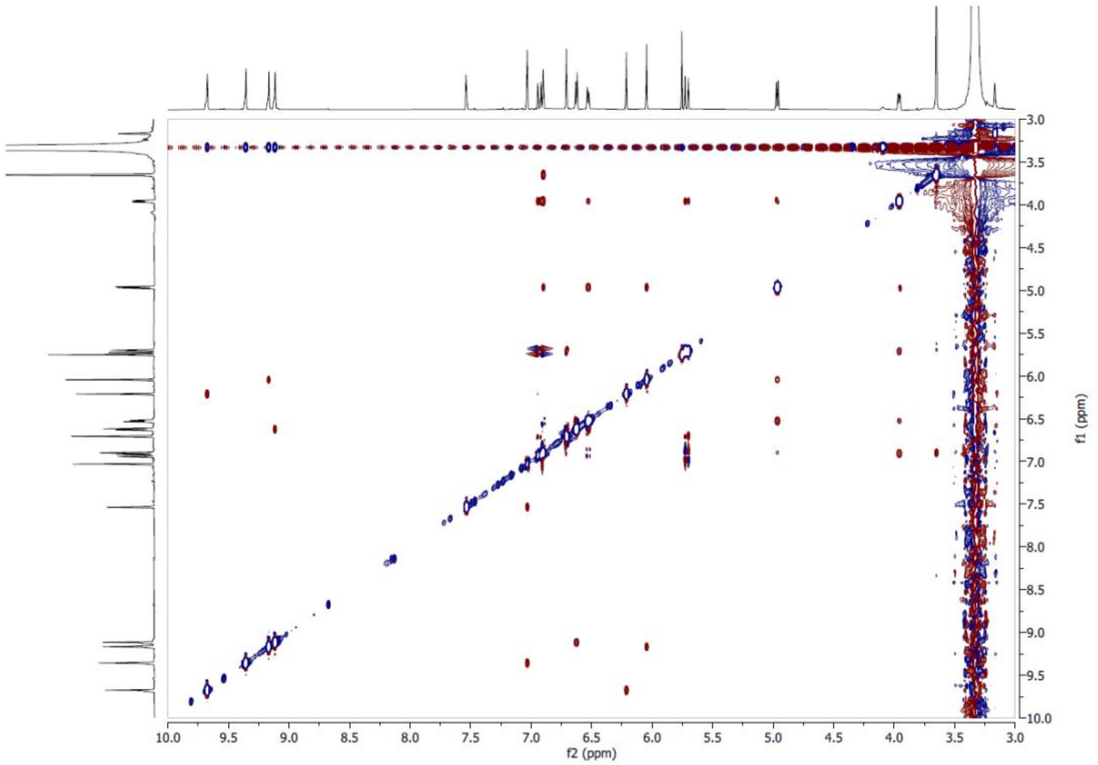

Supplementary Figure 100. ROESY NMR spectrum of compound **17** in DMSO-d_6

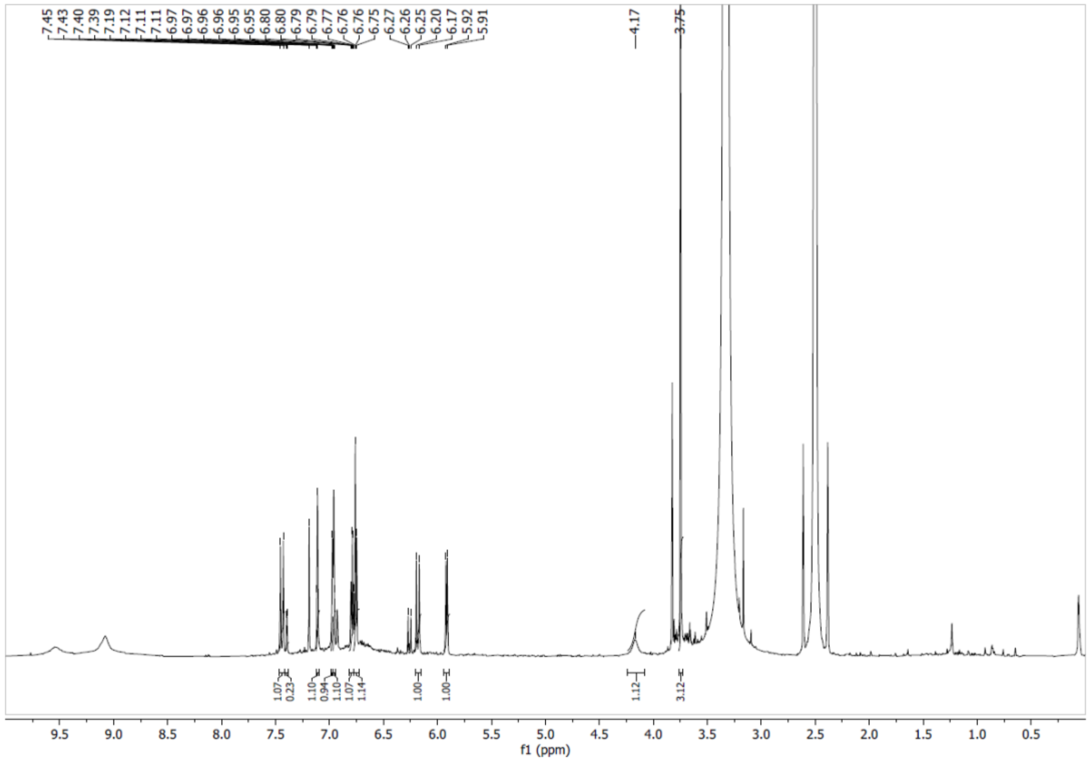

Supplementary Figure 101. ^{1}H NMR spectrum of compound **18** in DMSO-d_6 at 600 MHz

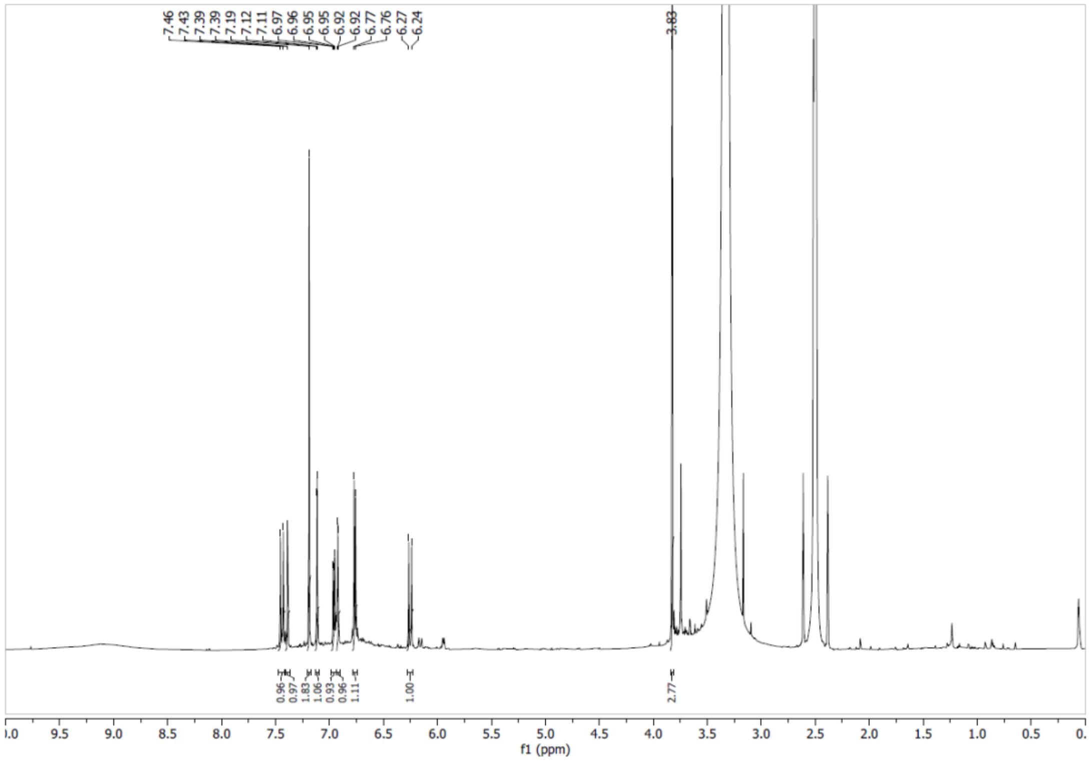

Supplementary Figure 102. ^{1}H NMR spectrum of compound **19** in DMSO-d_6 at 600 MHz

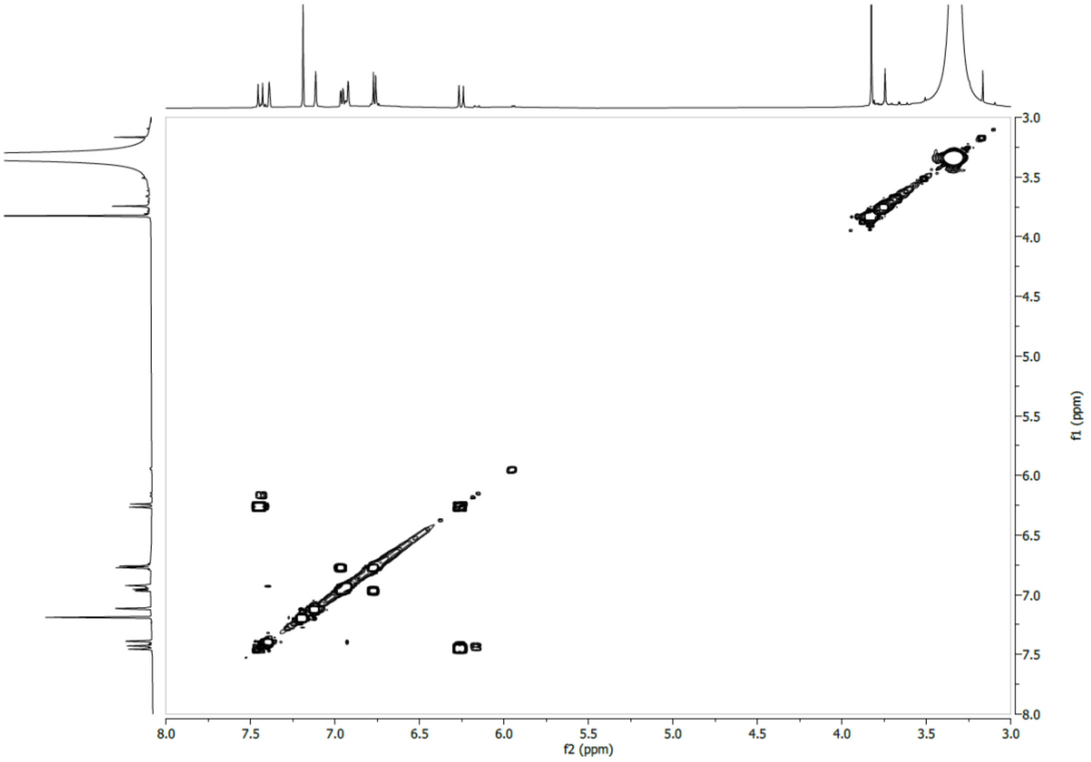

Supplementary Figure 103. COSY NMR spectrum of compound **19** in DMSO-d_6

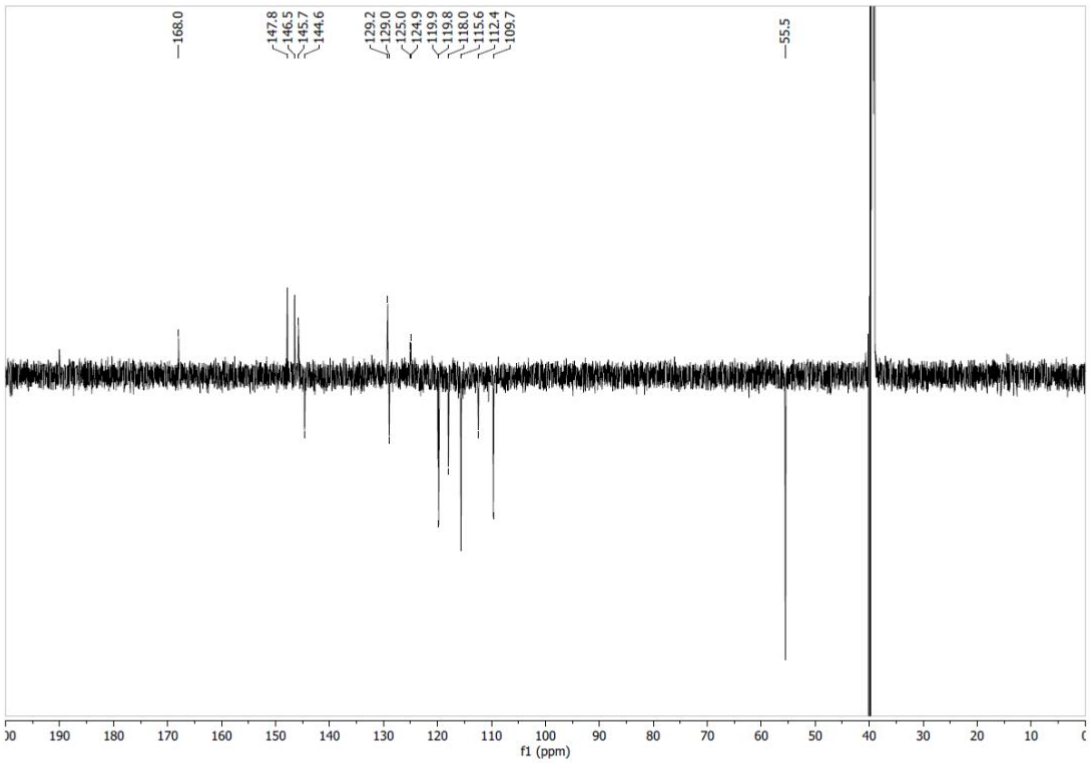

Supplementary Figure 104. ^{13}C-DEPTQ NMR spectrum of compound **19** in DMSO-d_6 at 151 MHz

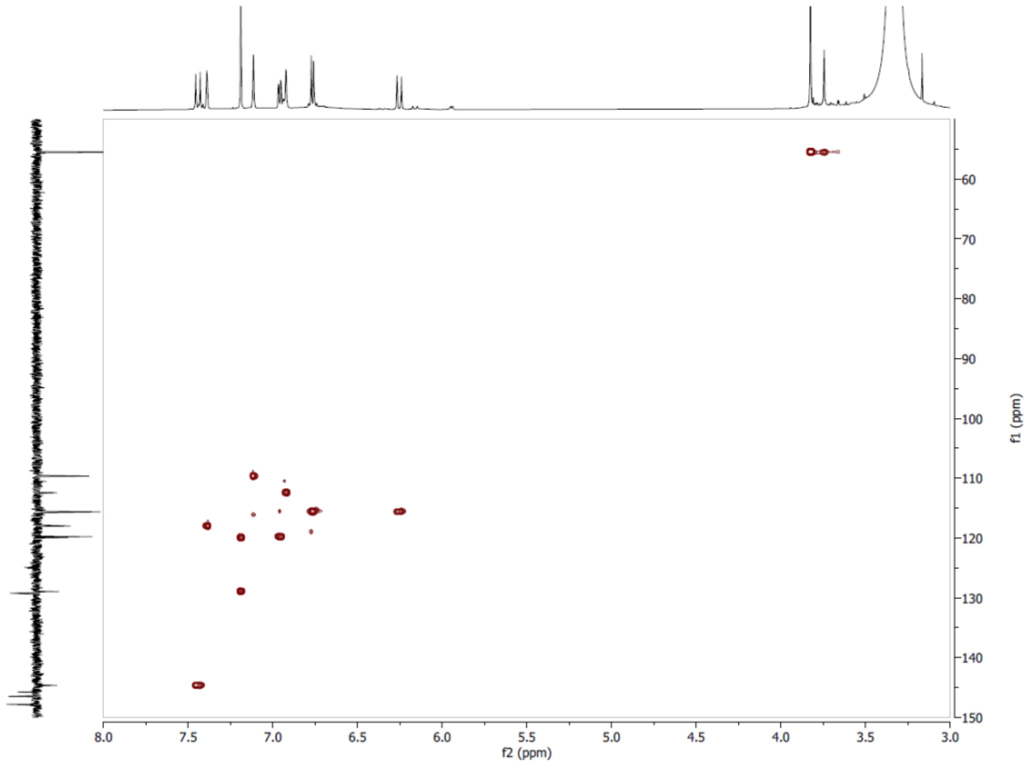

Supplementary Figure 105. Edited-HSQC NMR spectrum of compound **19** in DMSO-d_6

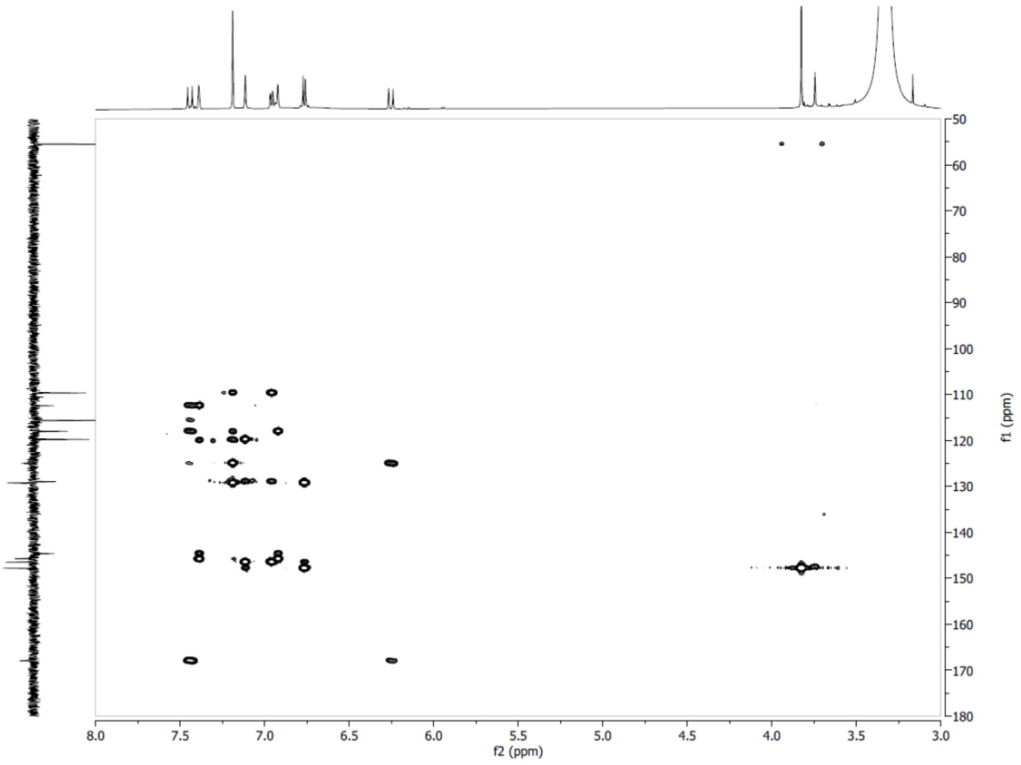

Supplementary Figure 106. HMBC NMR spectrum of compound **19** in DMSO-d_6

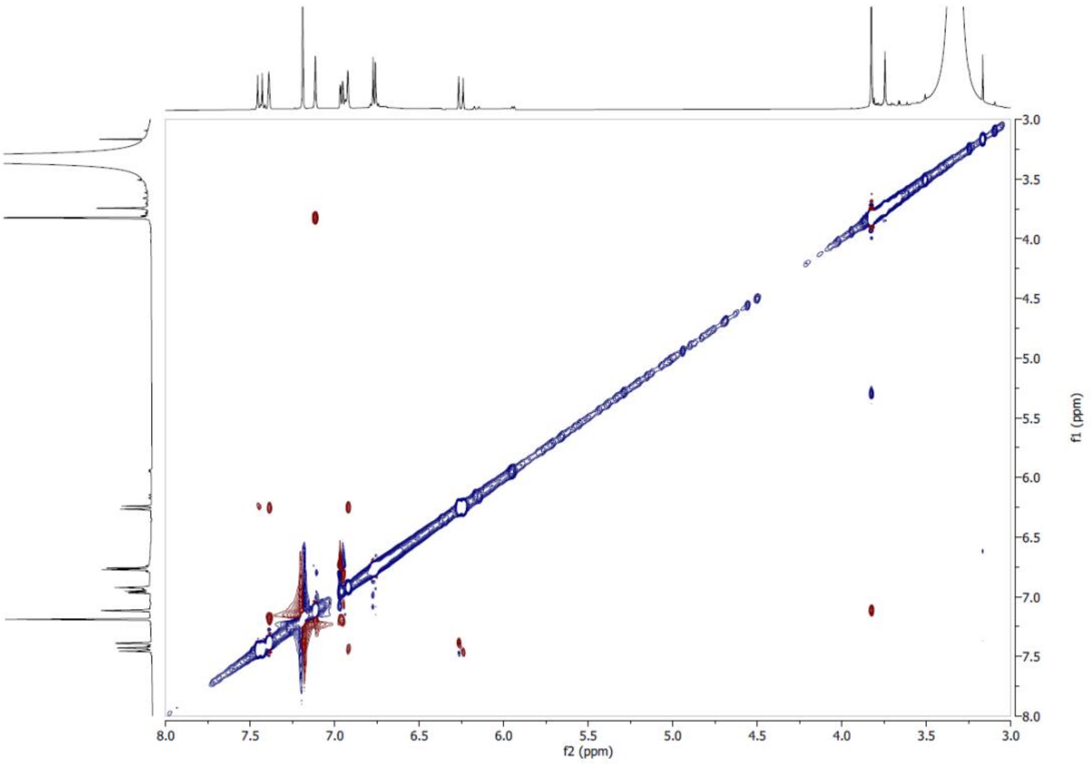

Supplementary Figure 107. ROESY NMR spectrum of compound **19** in DMSO-d_6

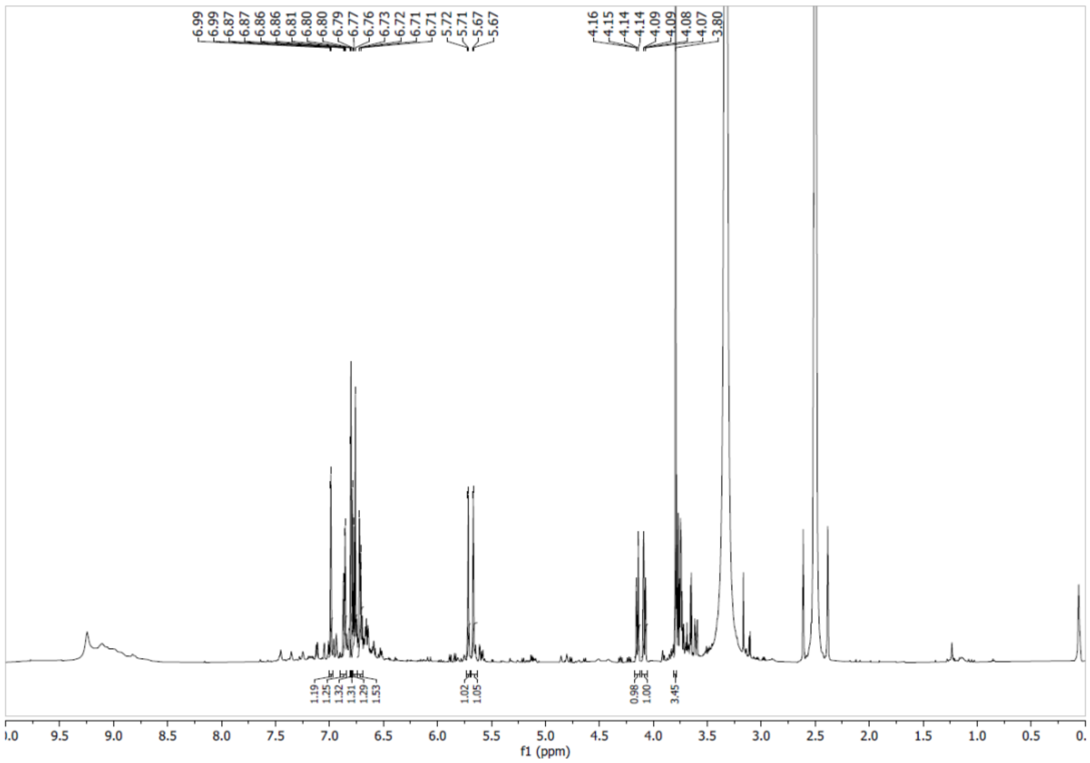

Supplementary Figure 108. ^{1}H NMR spectrum of compound **20** in DMSO-d_6 at 600 MHz

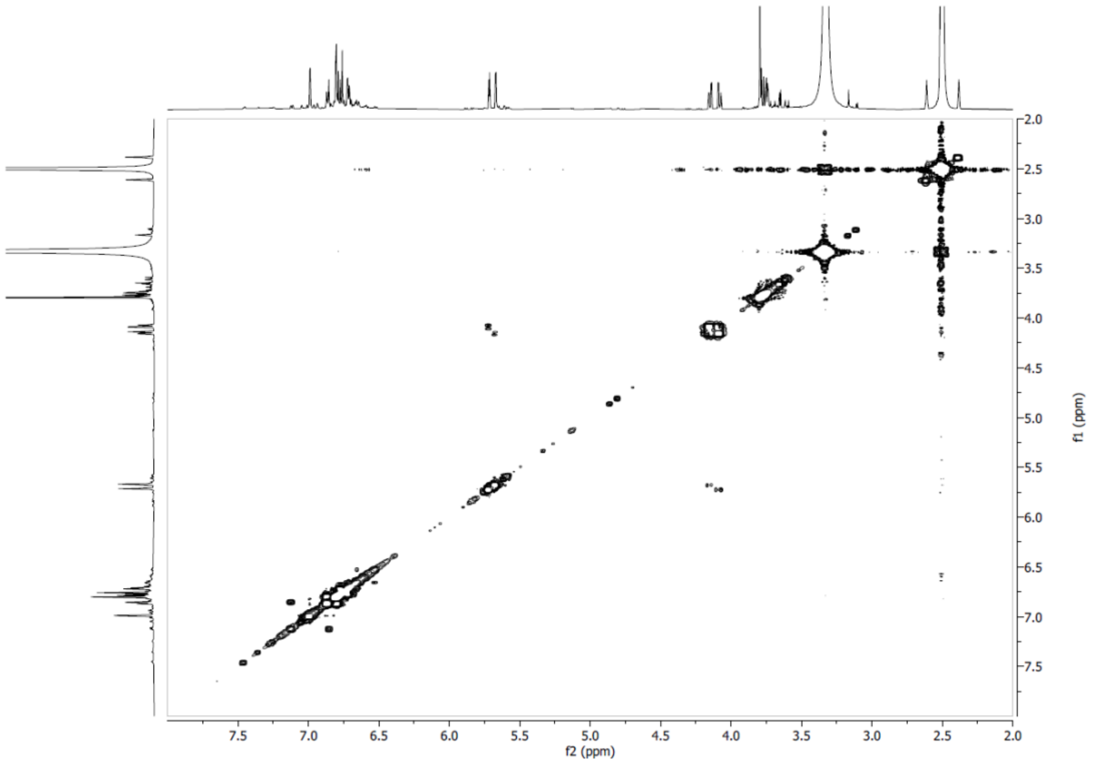

Supplementary Figure 109. COSY NMR spectrum of compound **20** in DMSO-d_6

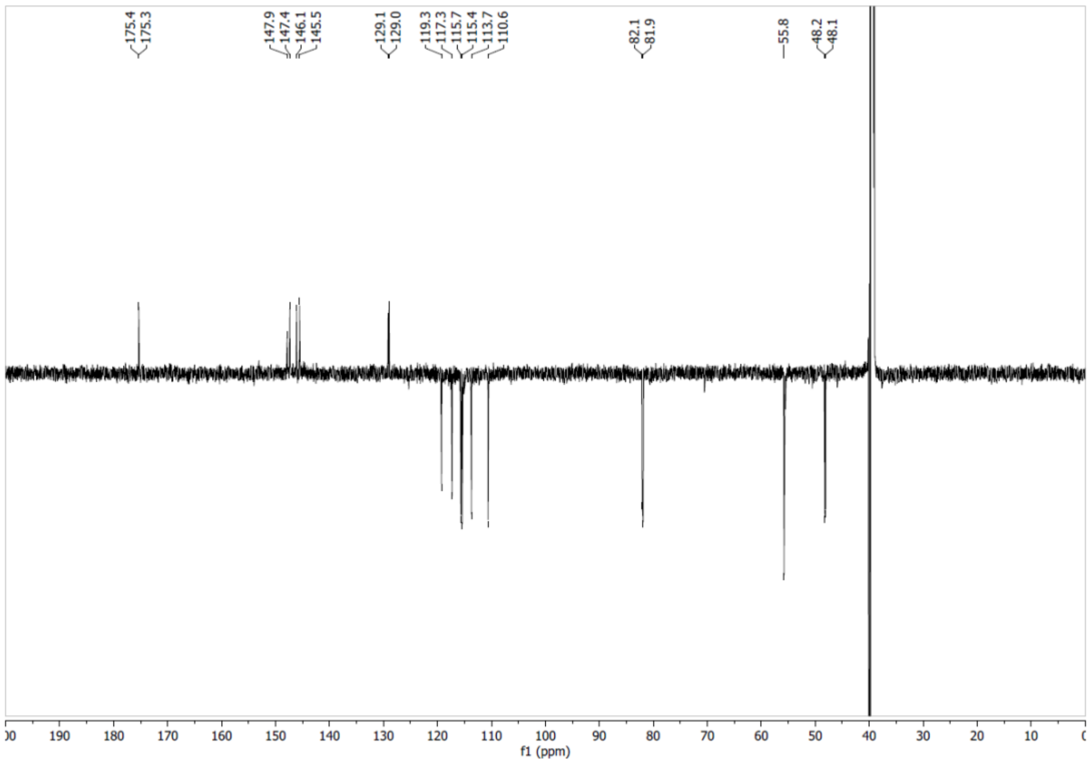

Supplementary Figure 110. ^{13}C-DEPTQ NMR spectrum of compound **20** in DMSO-d_6 at 151 MHz

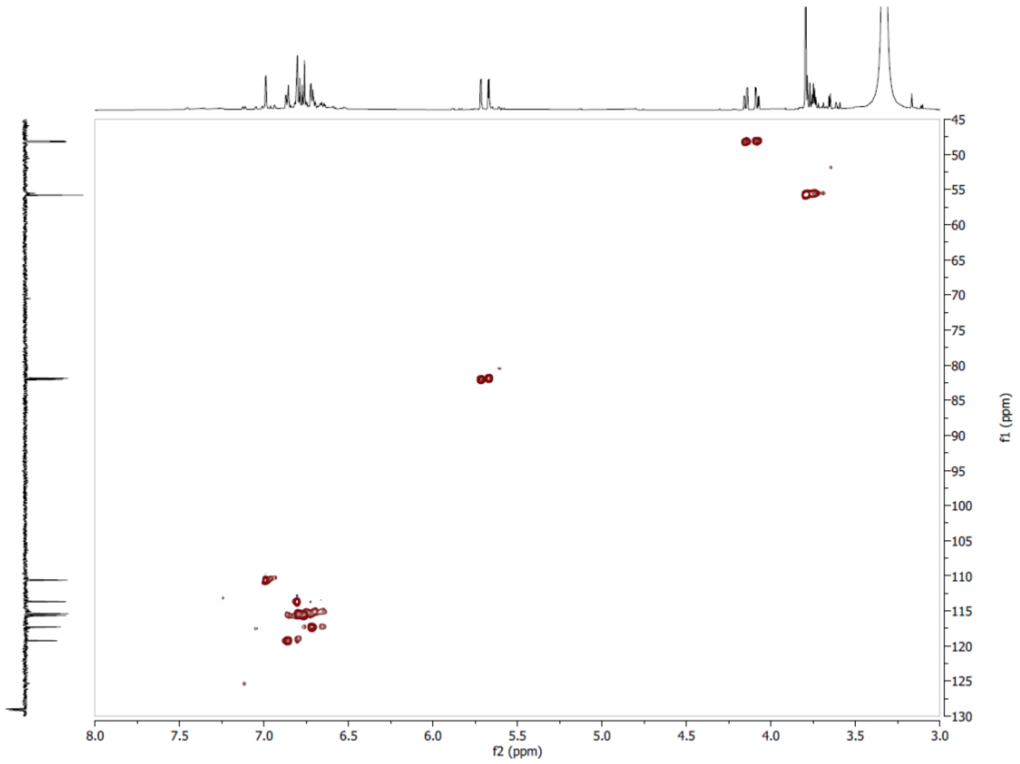

Supplementary Figure 111. Edited-HSQC NMR spectrum of compound **20** in DMSO-d_6

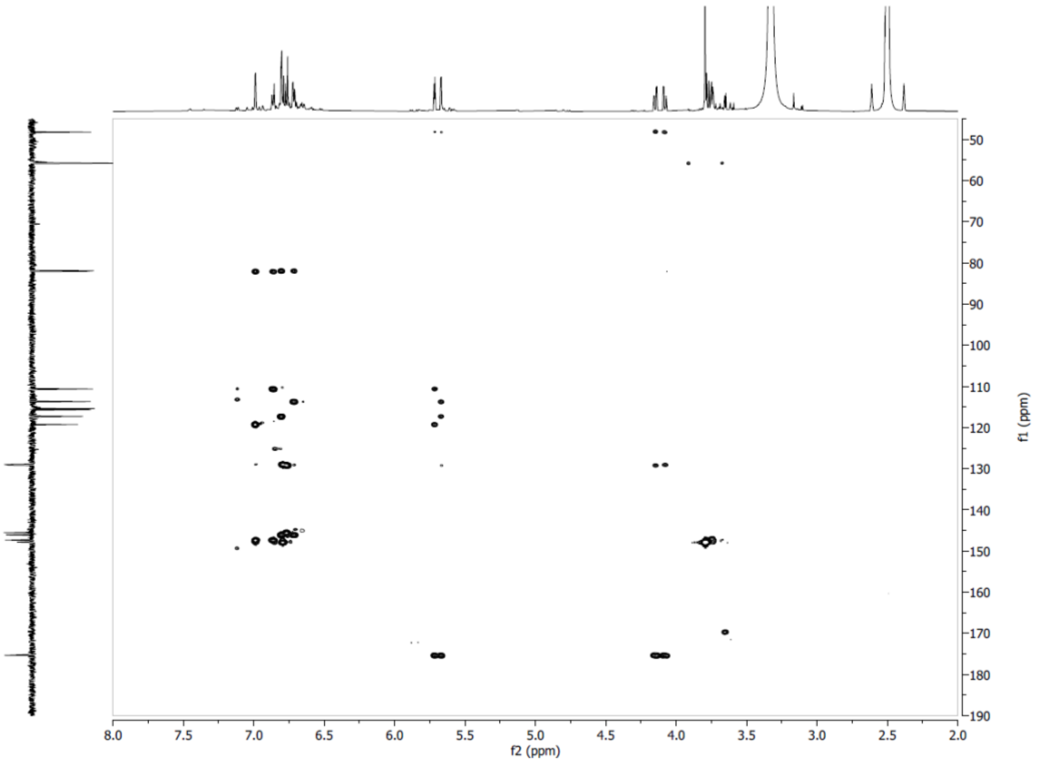

Supplementary Figure 112. HMBC NMR spectrum of compound **20** in DMSO-d_6

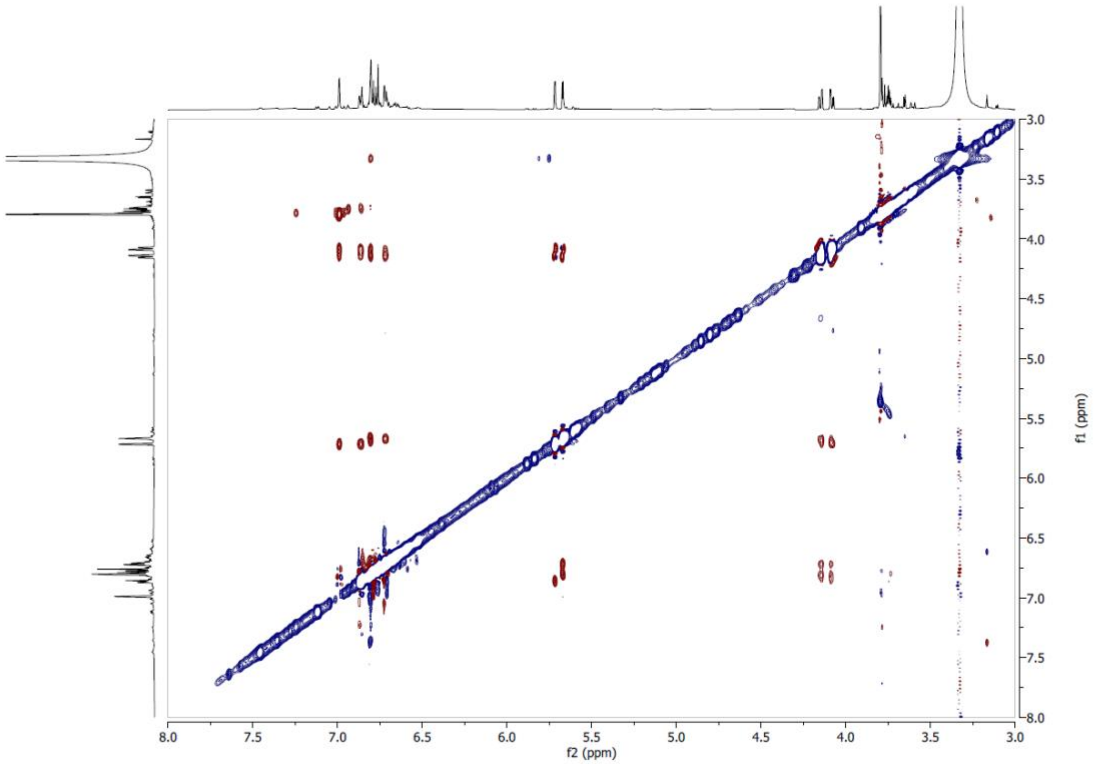

Supplementary Figure 113. ROESY NMR spectrum of compound **20** in DMSO-d_6

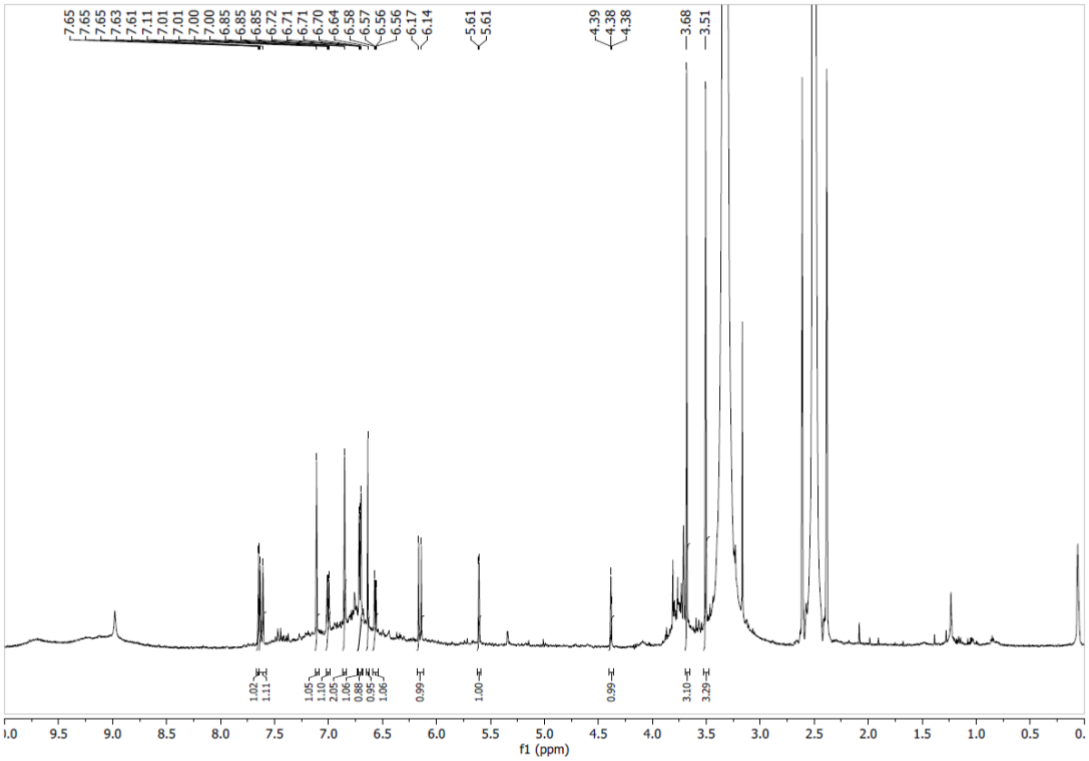

Supplementary Figure 114. ^{1}H NMR spectrum of compound **21** in DMSO-d_6 at 600 MHz

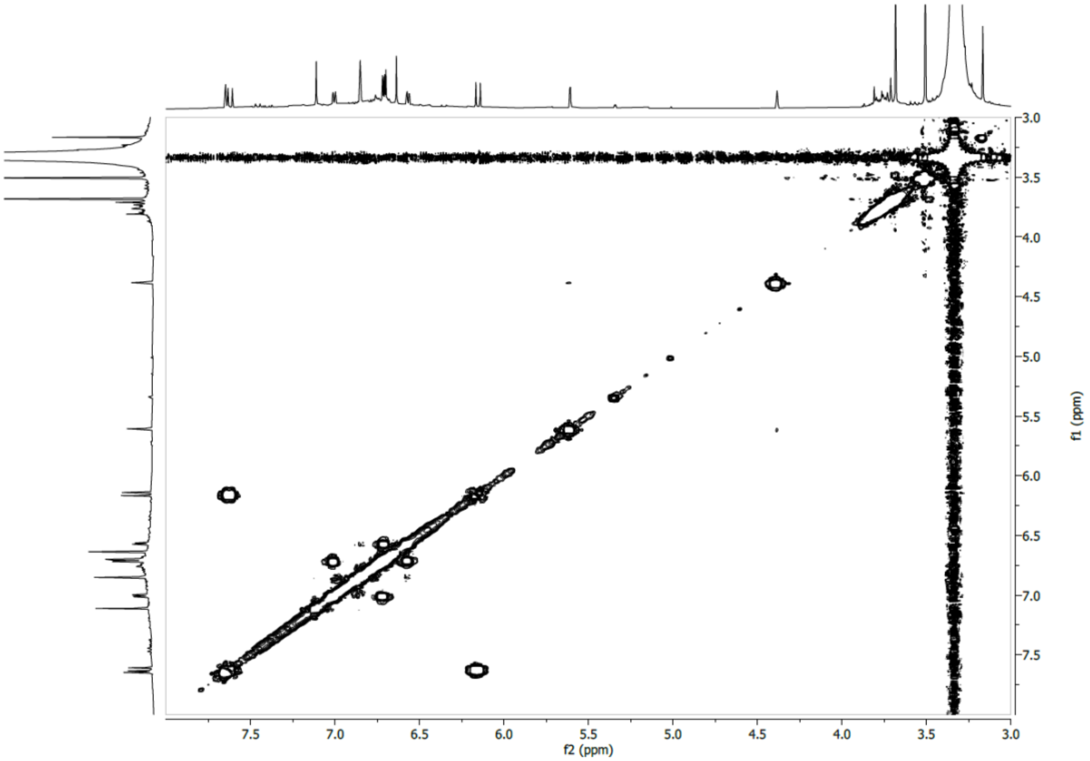

Supplementary Figure 115. COSY NMR spectrum of compound **21** in DMSO-d_6

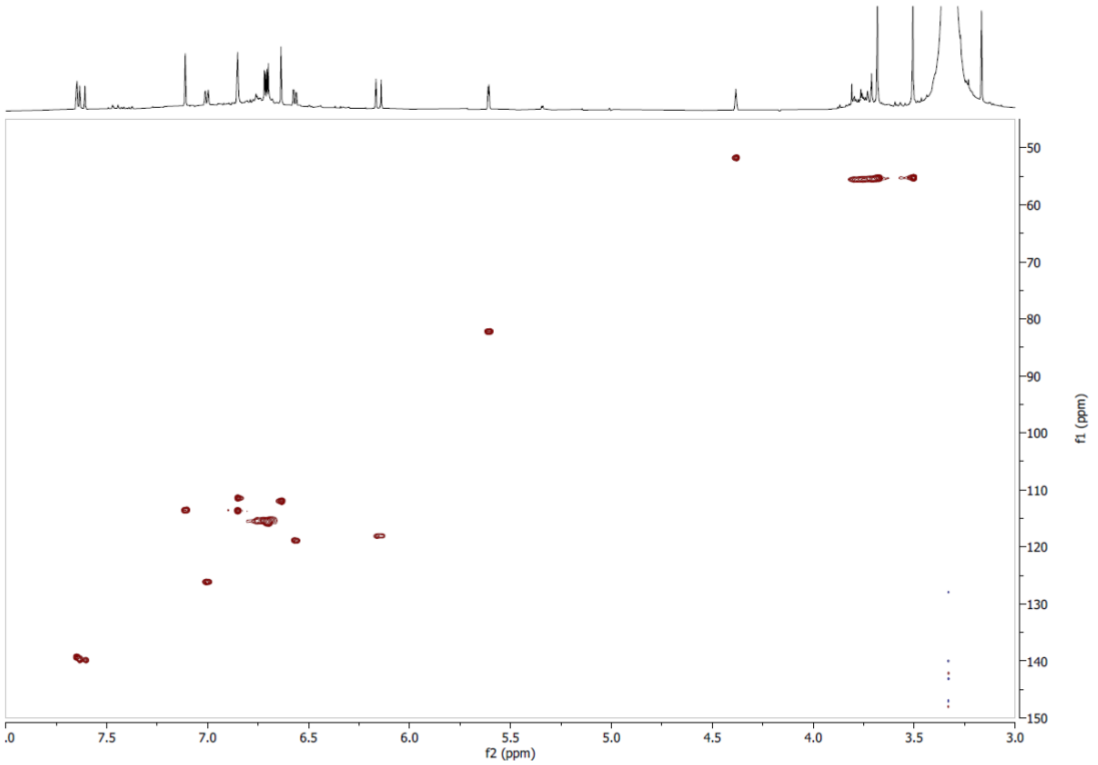

Supplementary Figure 116. Edited-HSQC NMR spectrum of compound **21** in DMSO-d_6

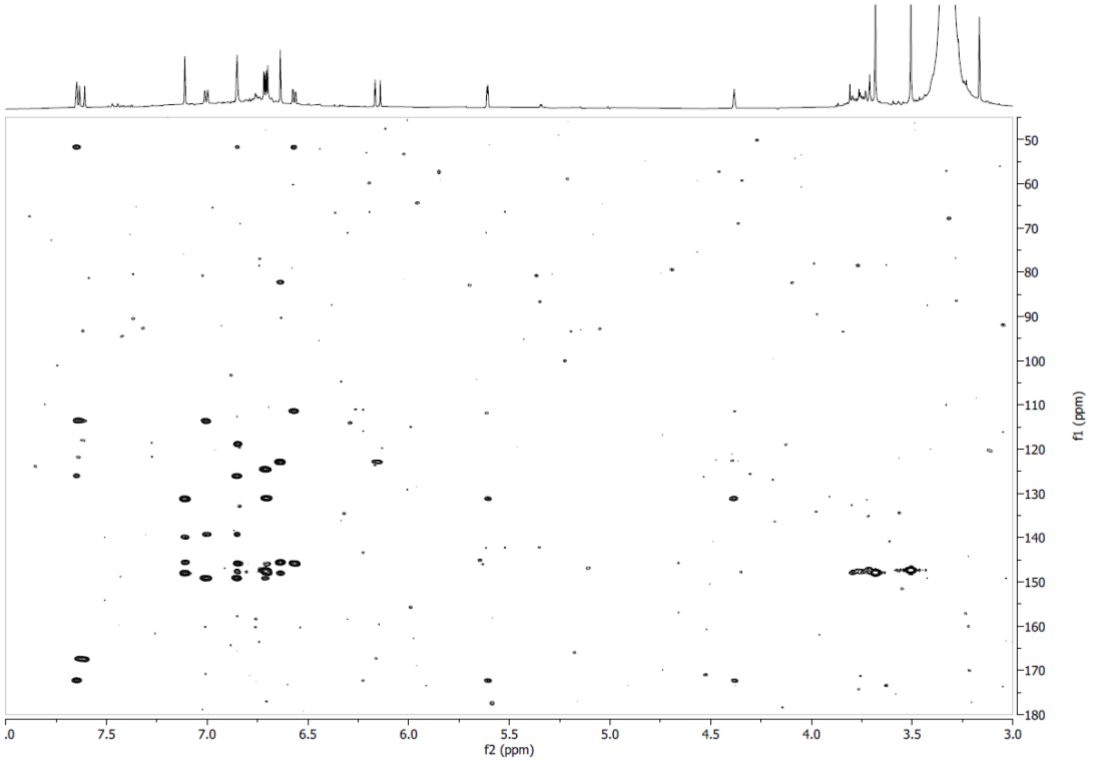

Supplementary Figure 117. HMBC NMR spectrum of compound **21** in DMSO-d_6

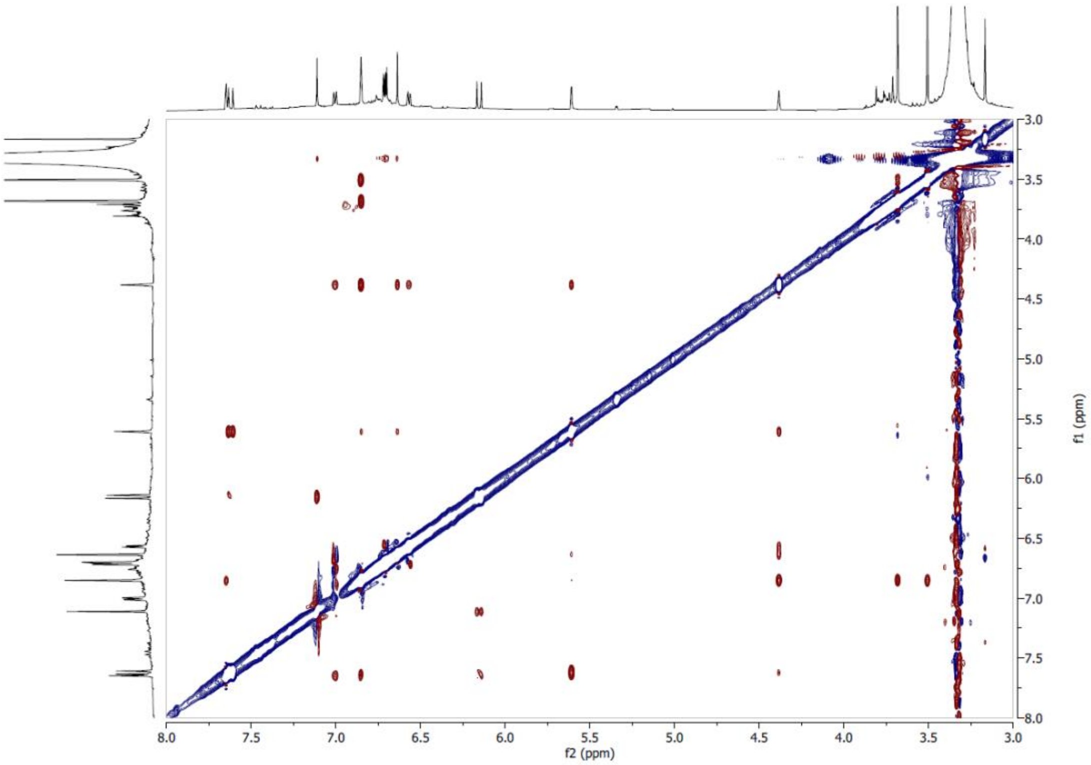

Supplementary Figure 118. ROESY NMR spectrum of compound **21** in DMSO-d_6

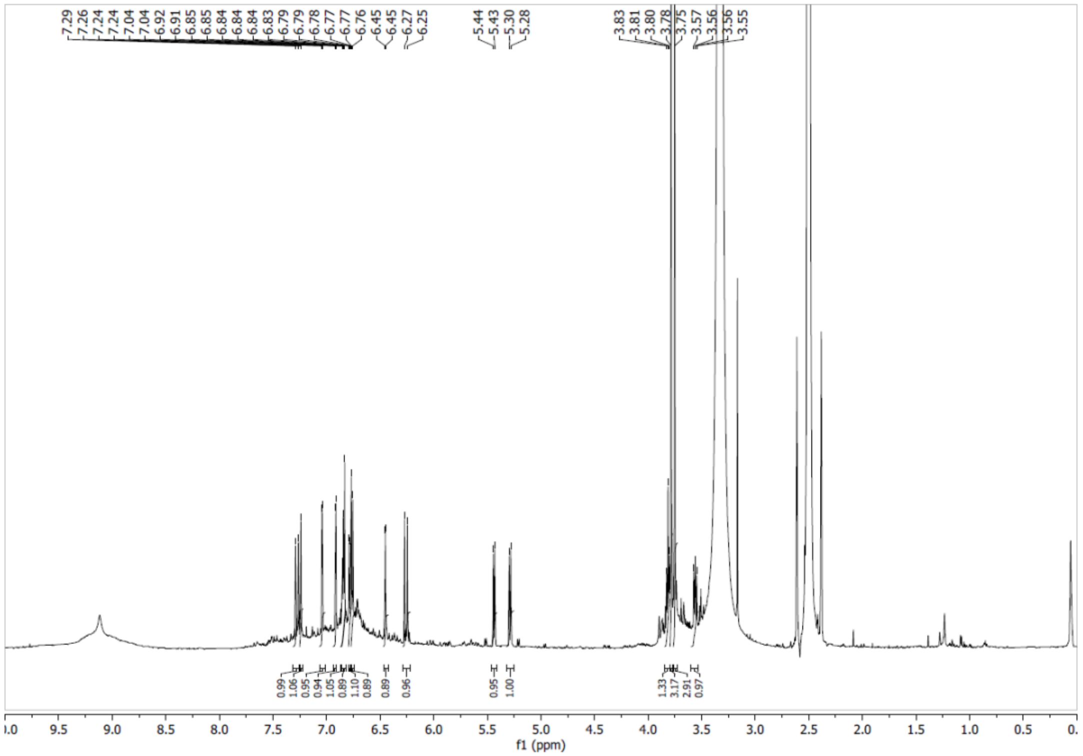

Supplementary Figure 119. ^{1}H NMR spectrum of compound **22** in DMSO-d_6 at 600 MHz

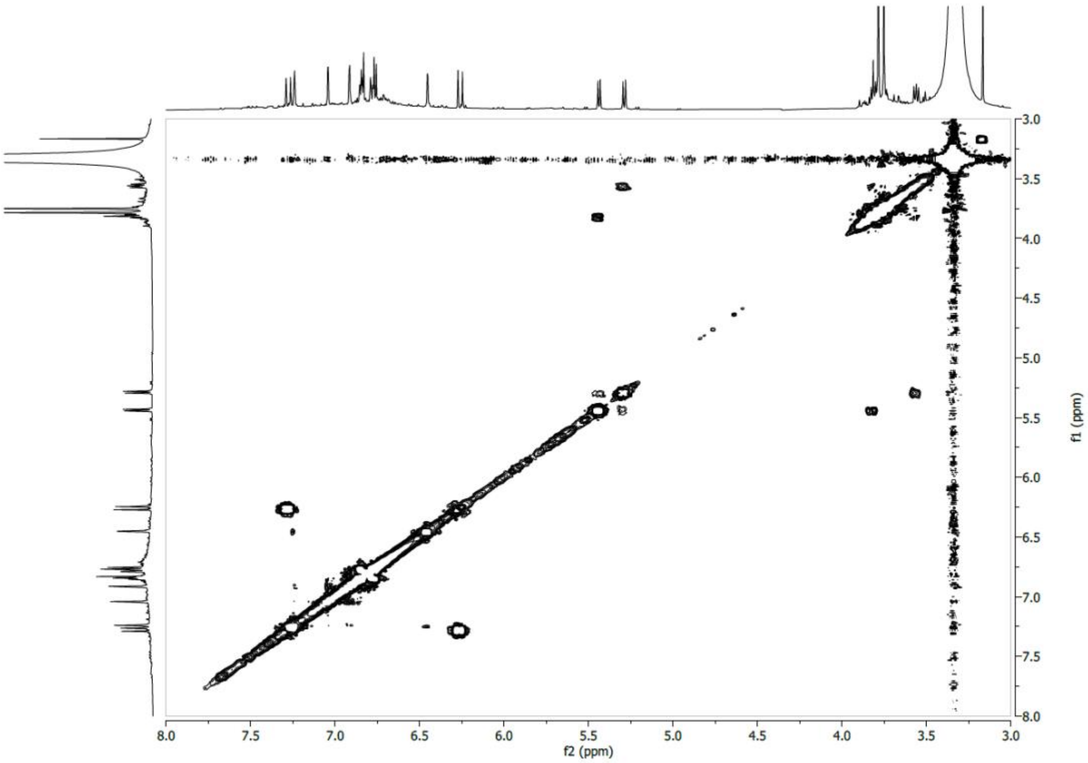

Supplementary Figure 120. COSY NMR spectrum of compound **22** in DMSO-d_6

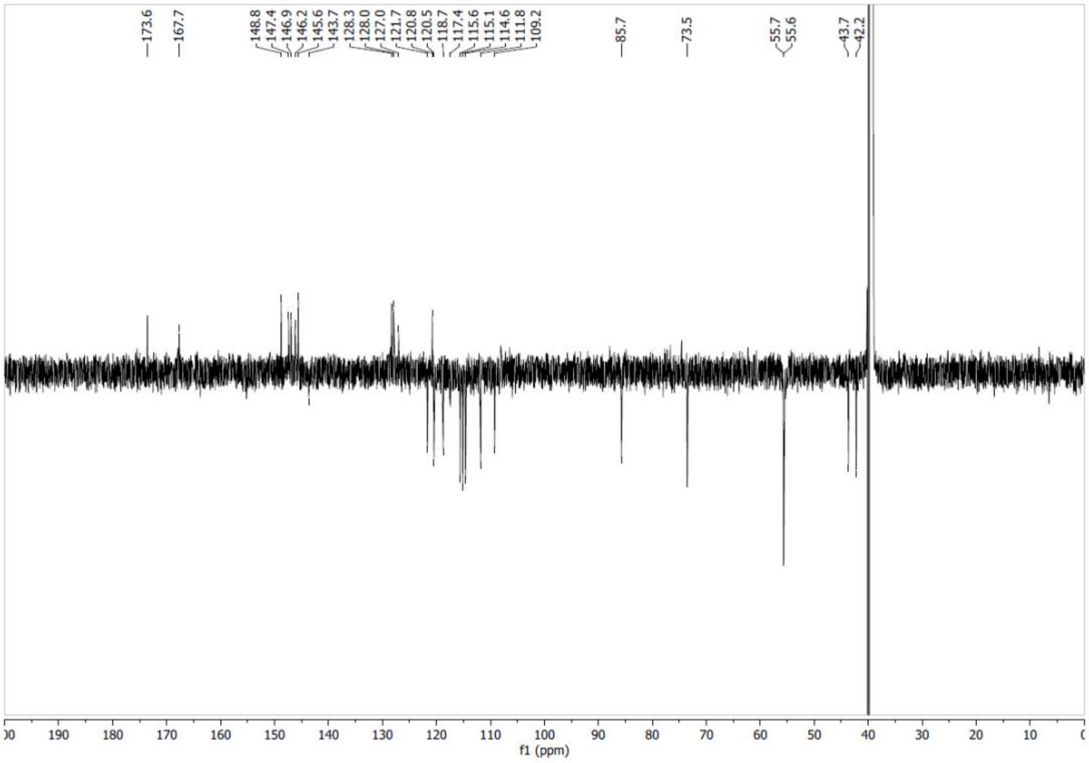

Supplementary Figure 121. ^{13}C-DEPTQ NMR spectrum of compound **22** in DMSO-d_6 at 151 MHz

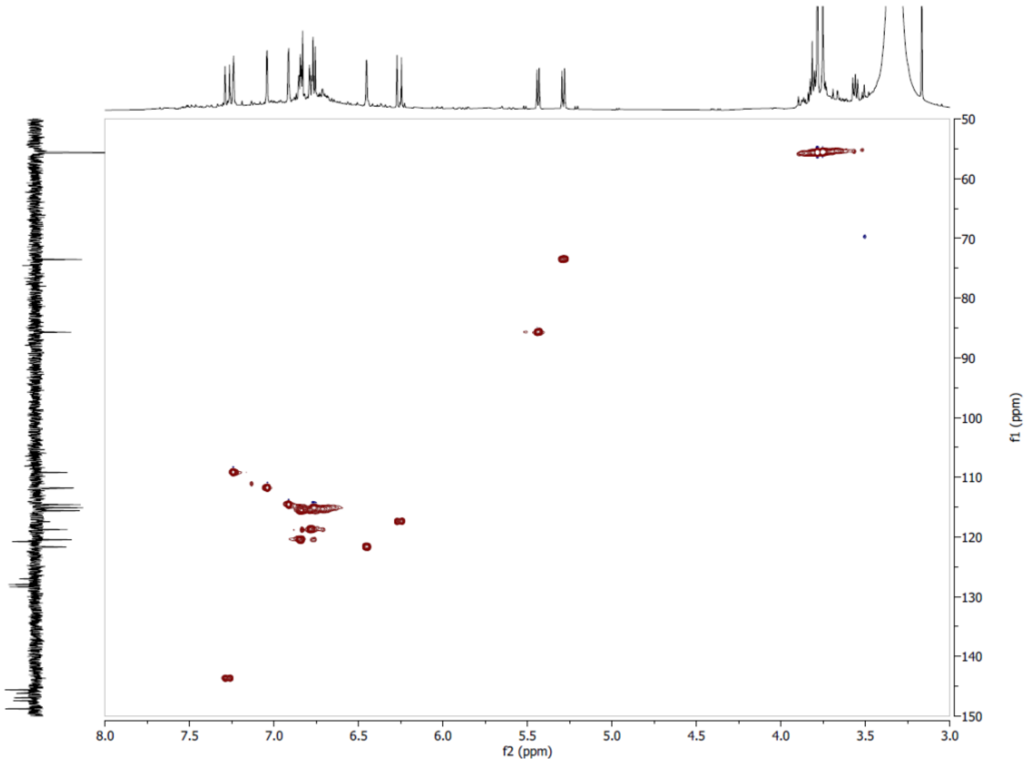

Supplementary Figure 122. Edited-HSQC NMR spectrum of compound **22** in DMSO-d_6

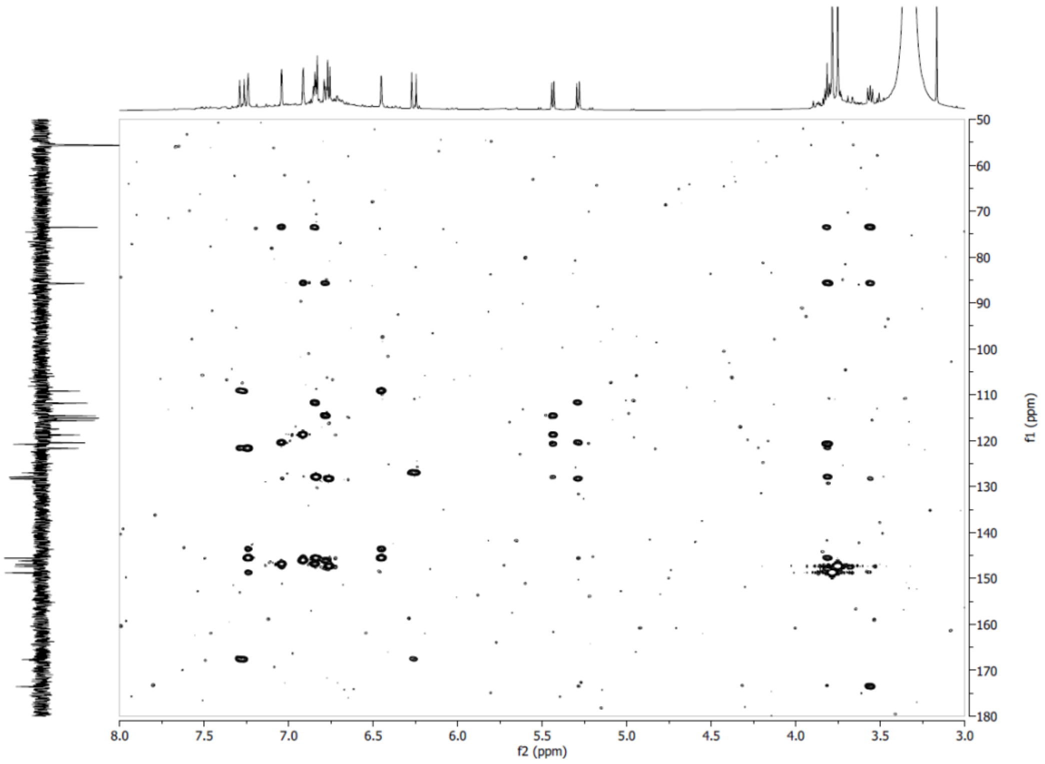

Supplementary Figure 123. HMBC NMR spectrum of compound **22** in DMSO-d_6

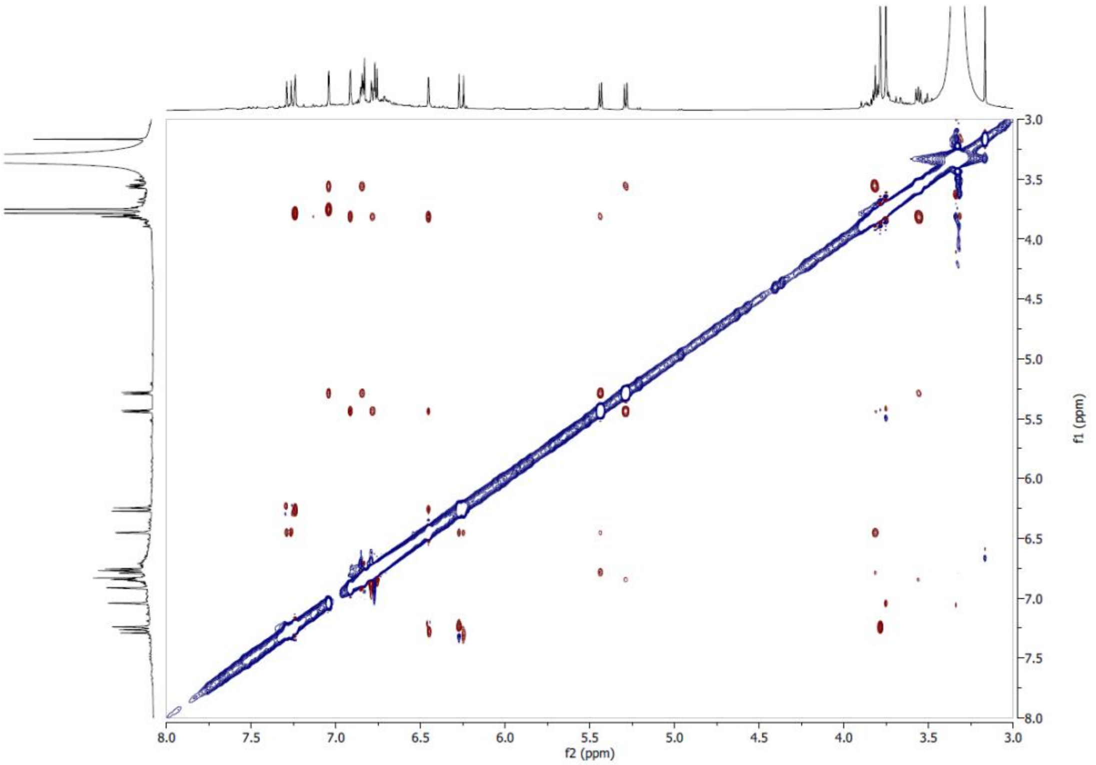

Supplementary Figure 124. ROESY NMR spectrum of compound **22** in DMSO-d_6

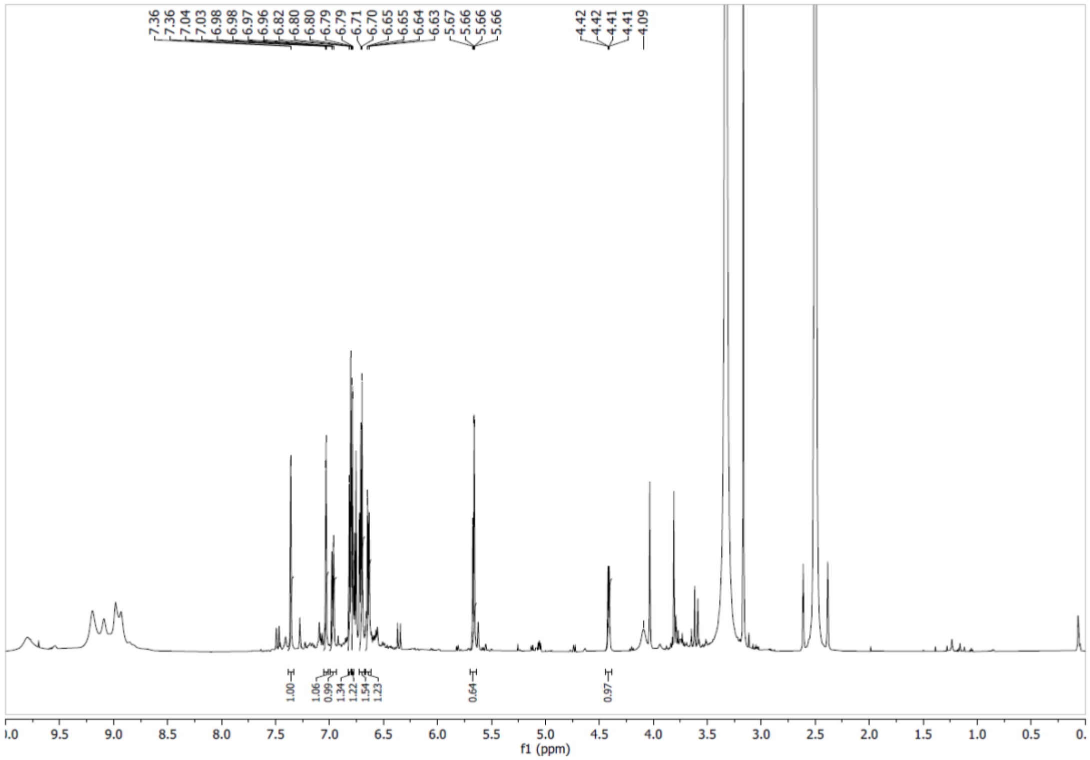

Supplementary Figure 125. ^{1}H NMR spectrum of compound **23** in DMSO-d_6 at 600 MHz

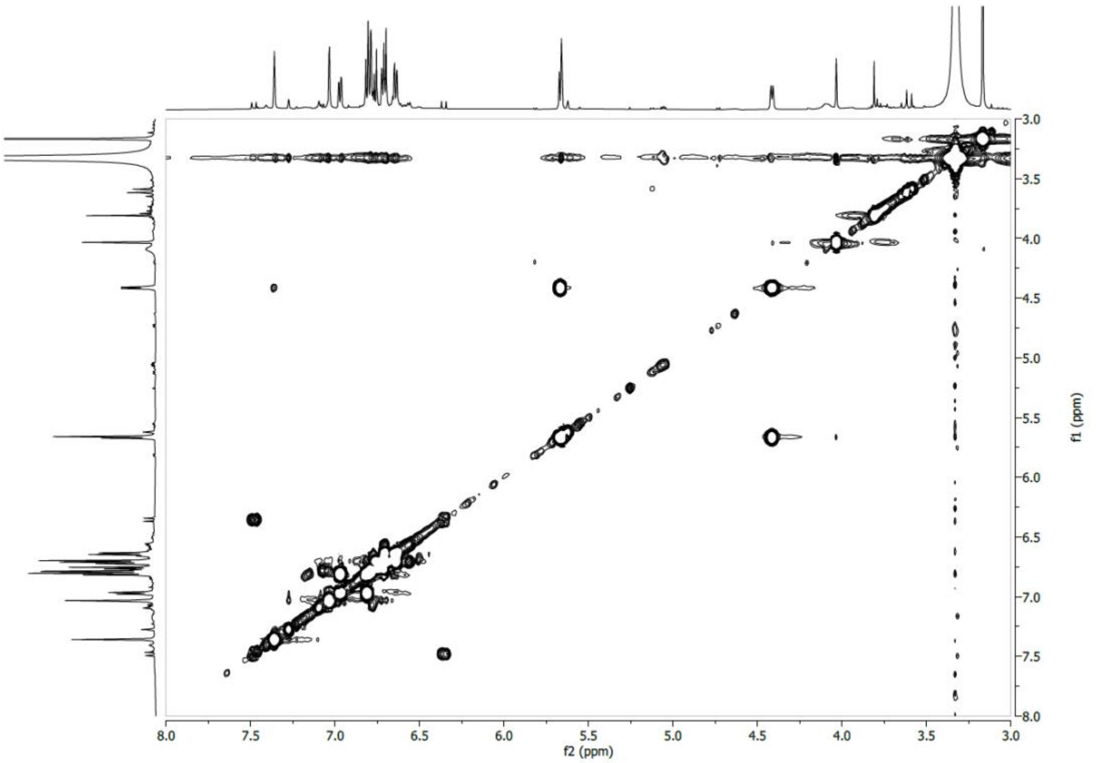

Supplementary Figure 126. COSY NMR spectrum of compound **23** in DMSO-d_6

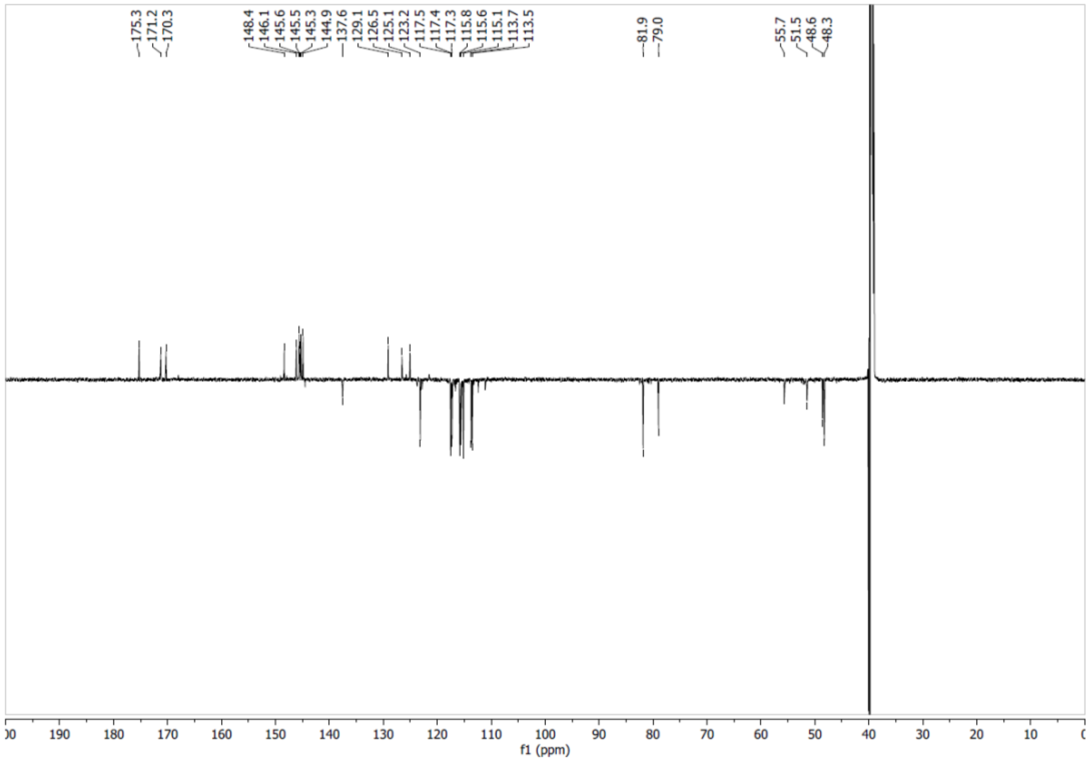

Supplementary Figure 127. ^{13}C-DEPTQ NMR spectrum of compound **23** in DMSO-d_6 at 151 MHz

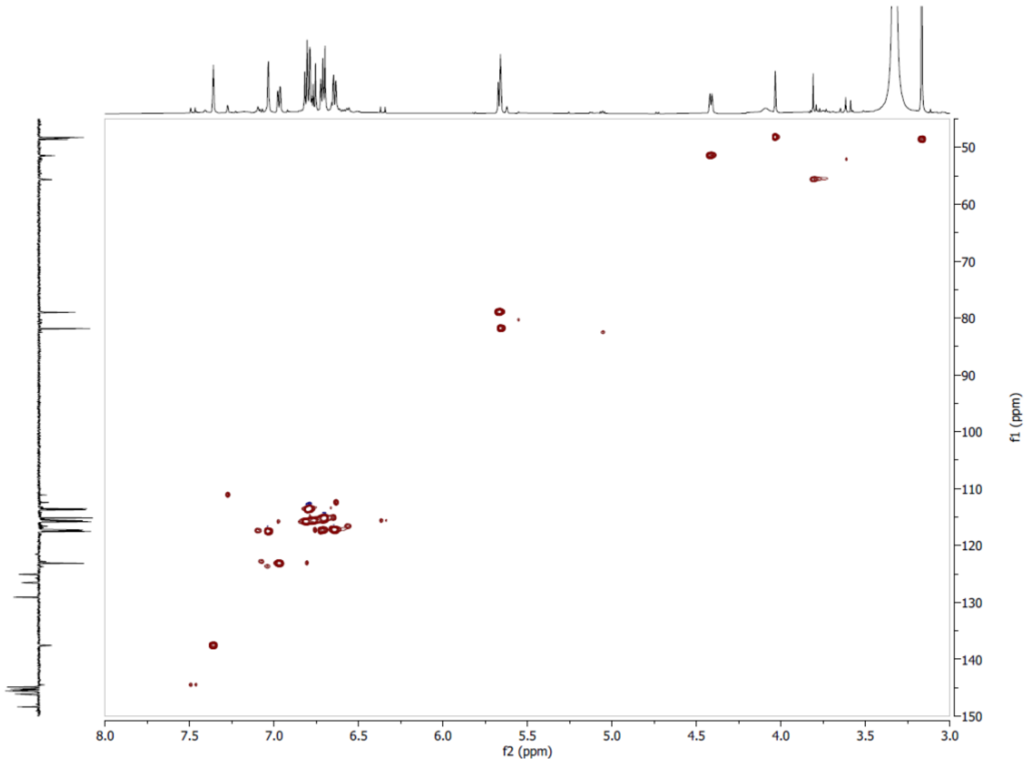

Supplementary Figure 128. Edited-HSQC NMR spectrum of compound **23** in DMSO-d_6

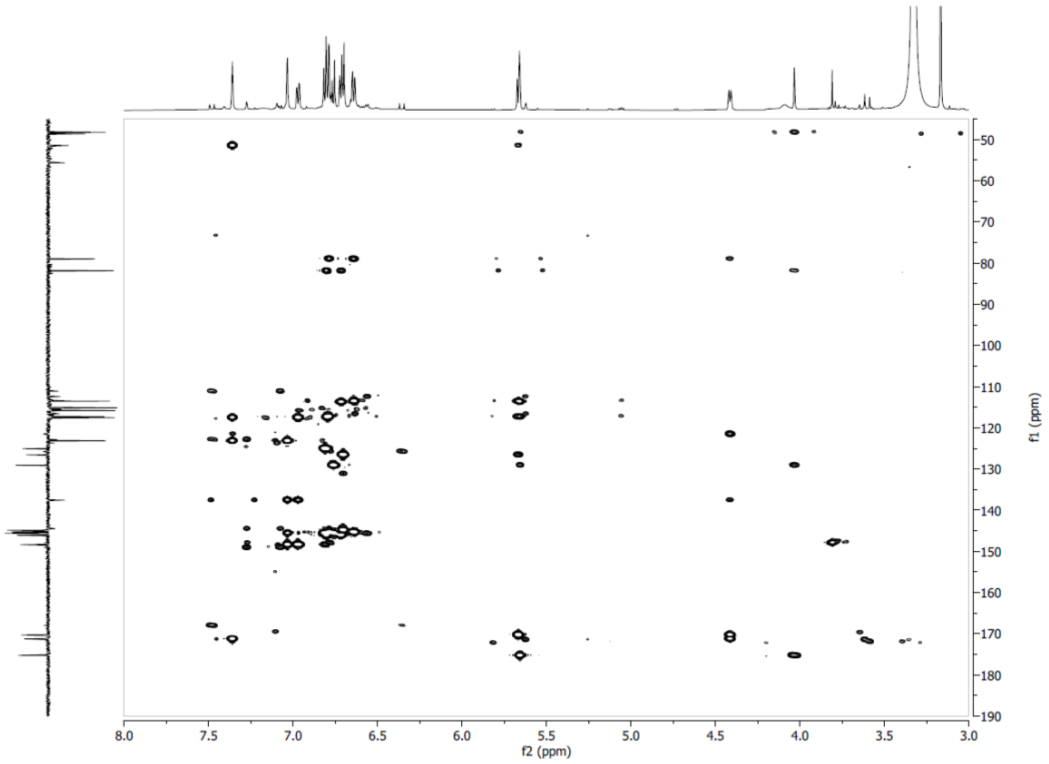

Supplementary Figure 129. HMBC NMR spectrum of compound **23** in DMSO-d_6

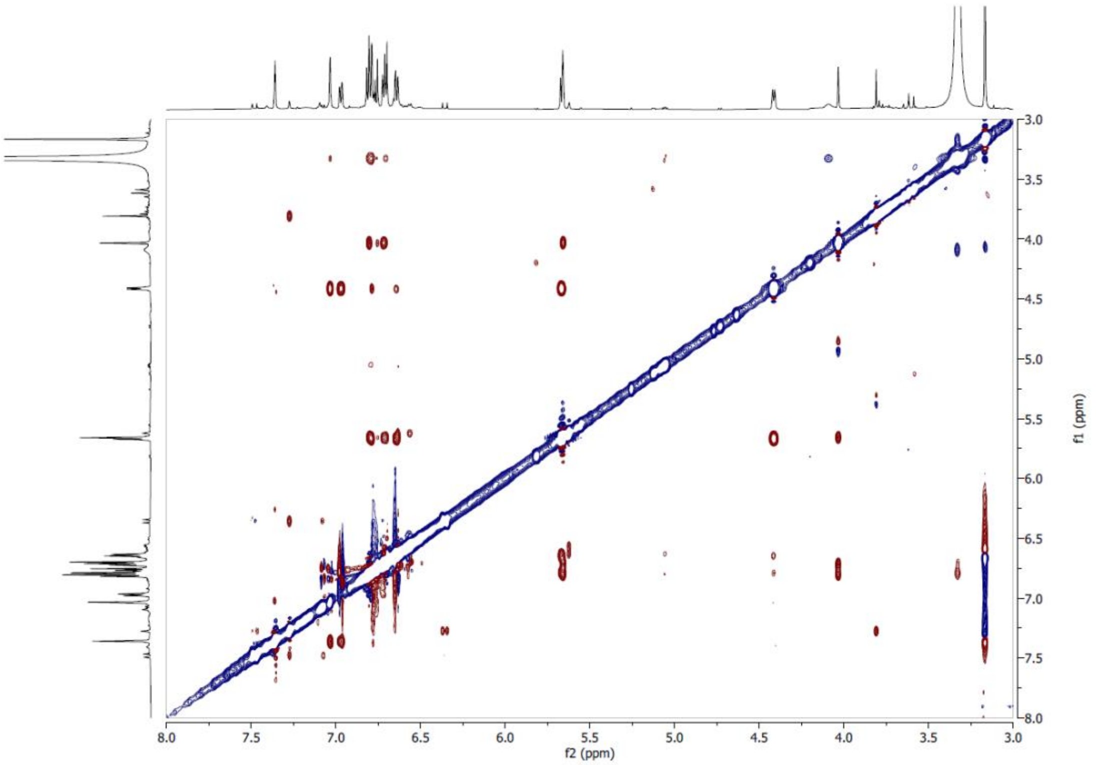

Supplementary Figure 130. ROESY NMR spectrum of compound **23** in DMSO-d_6

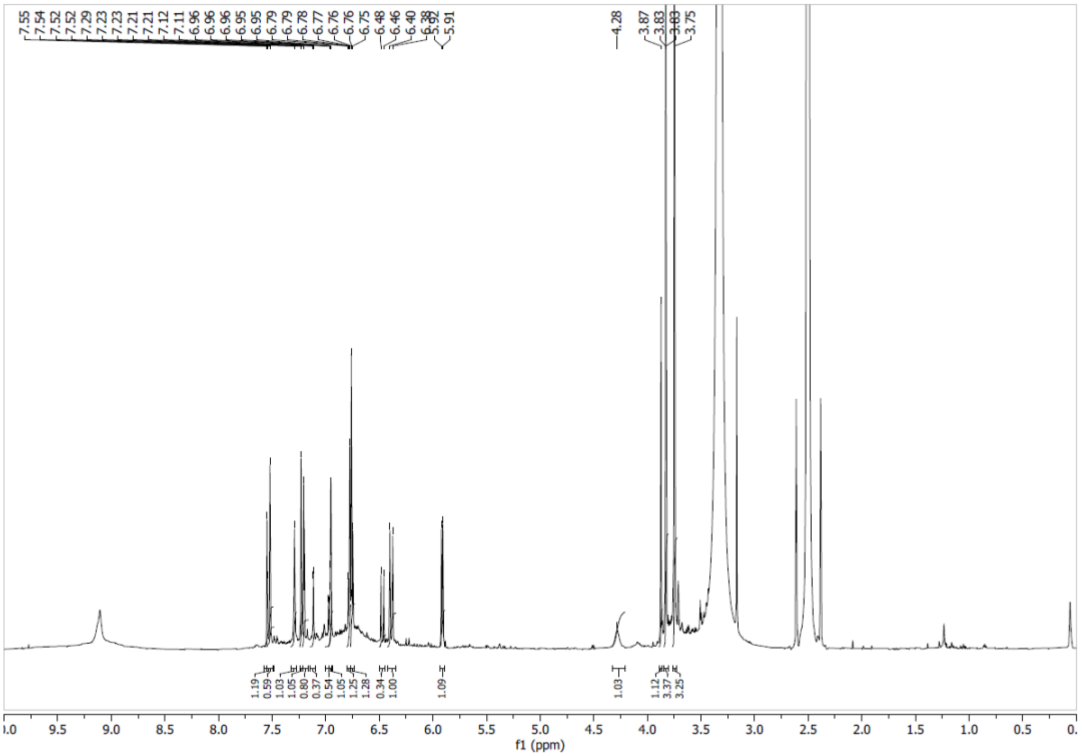

Supplementary Figure 131. ^{1}H NMR spectrum of compound **24** in DMSO-d_6 at 600 MHz

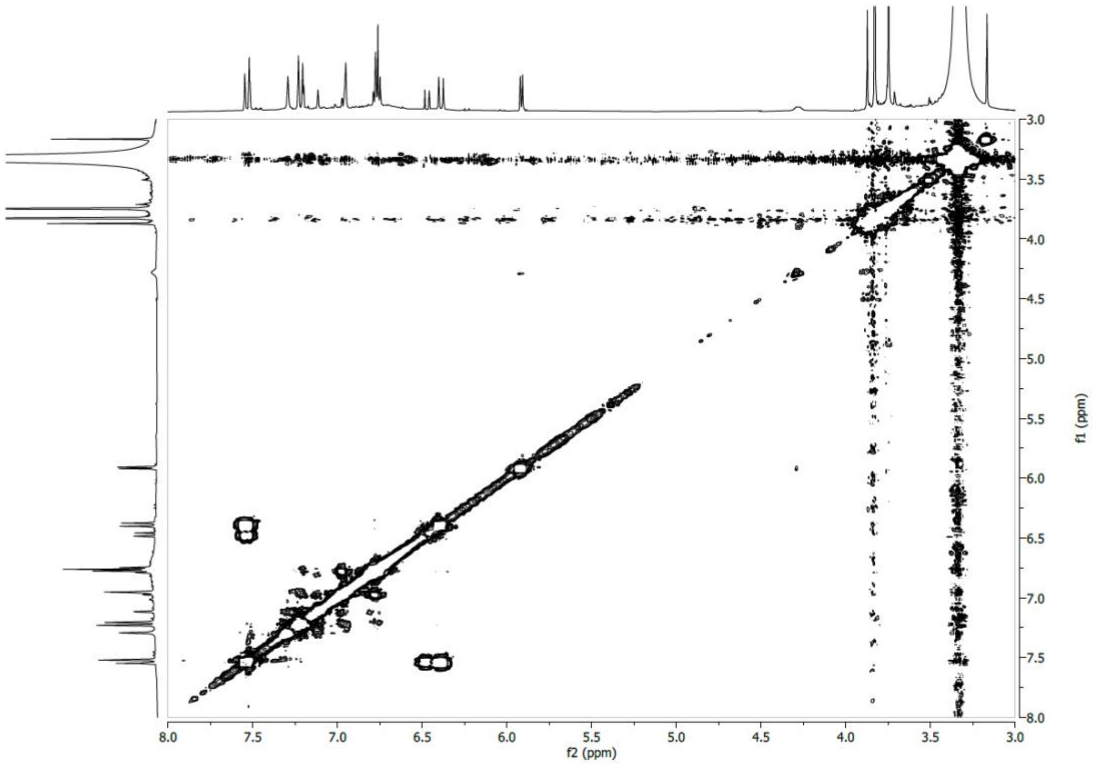

Supplementary Figure 132. COSY NMR spectrum of compound **24** in DMSO-d_6

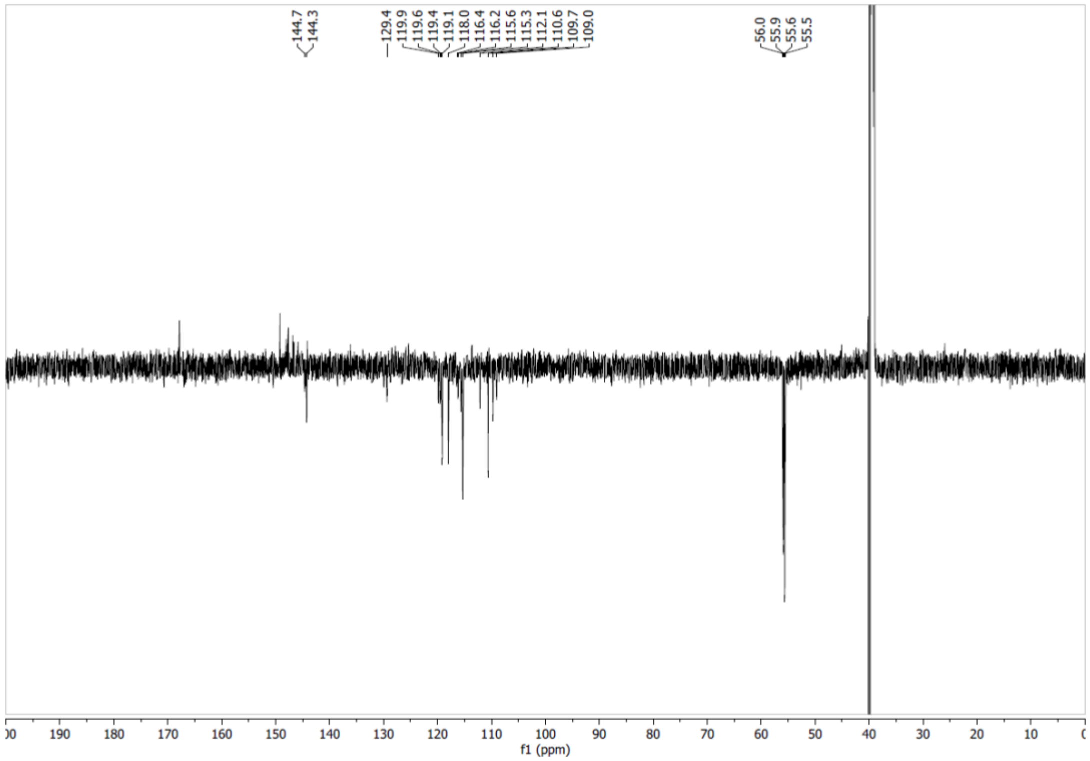

Supplementary Figure 133. ^{13}C-DEPTQ NMR spectrum of compound **24** in DMSO-d_6 at 151 MHz

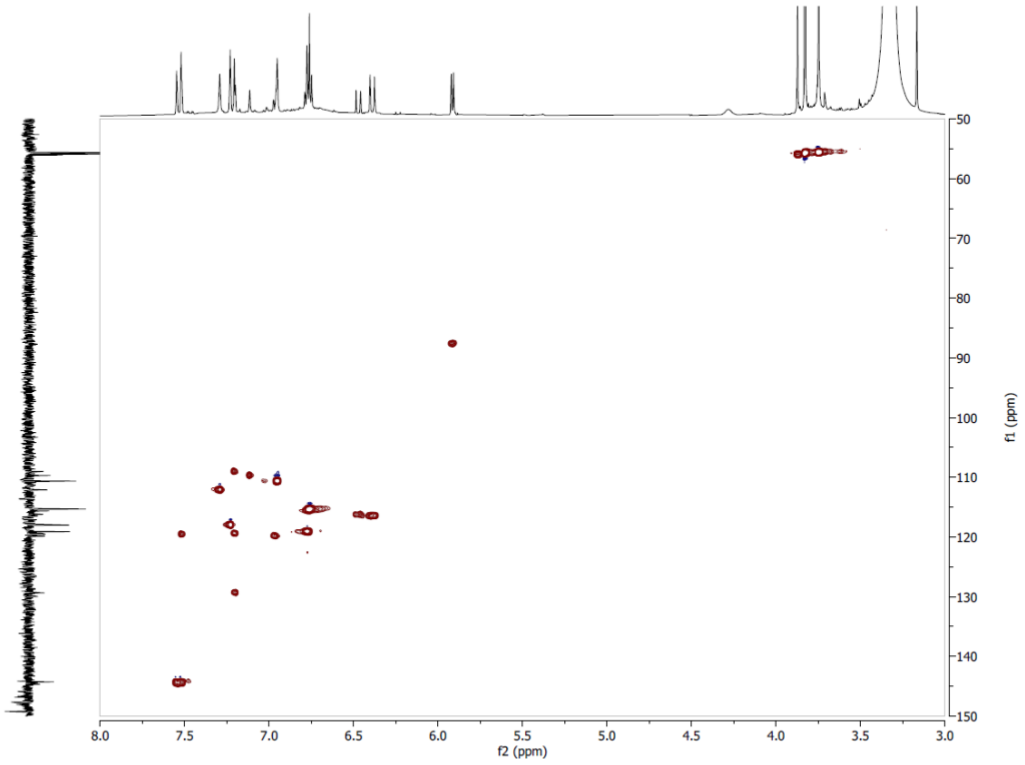

Supplementary Figure 134. Edited-HSQC NMR spectrum of compound **24** in DMSO-d_6

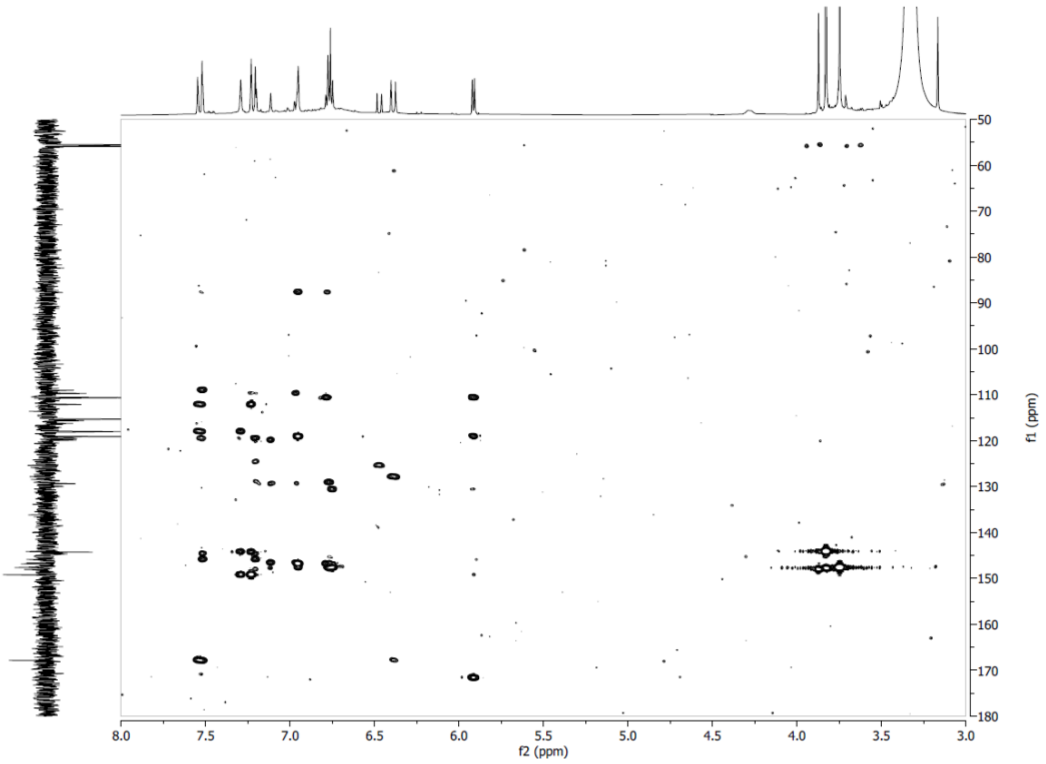

Supplementary Figure 135. HMBC NMR spectrum of compound **24** in DMSO-d_6

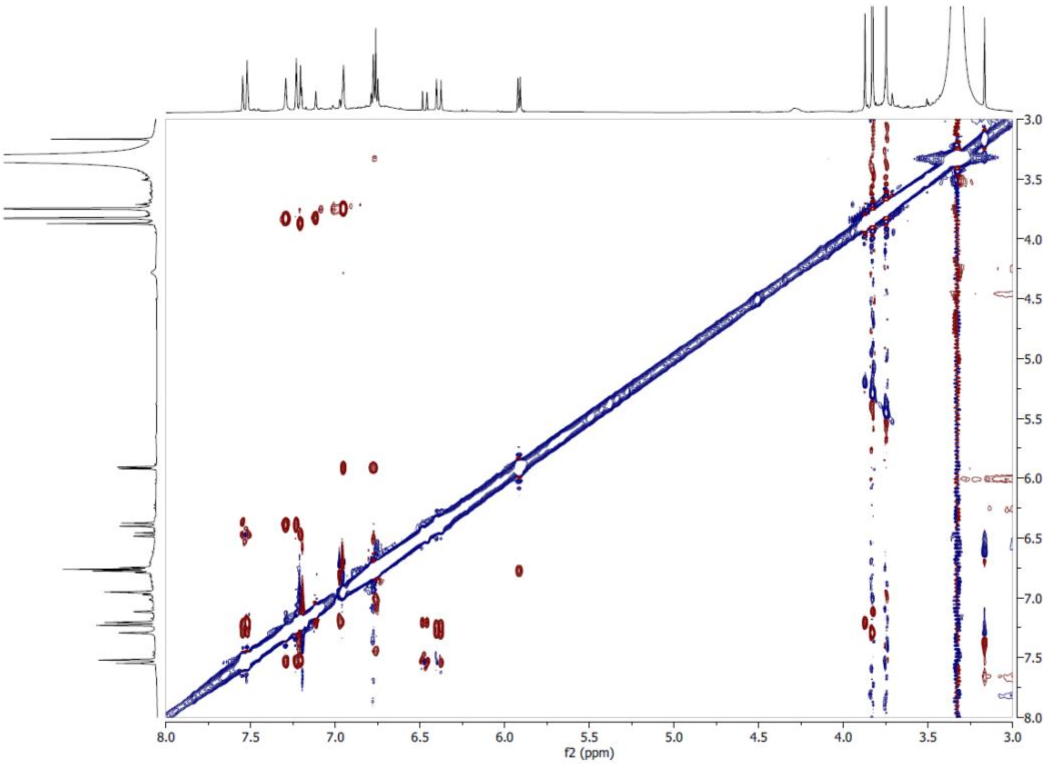

Supplementary Figure 136. ROESY NMR spectrum of compound **24** in DMSO-d_6

Supplementary Table 1. Chromatographic conditions optimized at the UHPLC level and transferred to the analytical and semipreparative HPLC scale

**Biotrans. reaction**	**UHPLC** (100 x 2.1 mm i.d., 1.7 µm)				**HPLC** (250 x 4.6 mm i.d., 5 µm)				**Semipreparative HPLC** (250 x 19 mm i.d., 5 µm)			
	Flow-rate (mL/min)	%A	%B	Time (min)	Flow-rate (mL/min)	%A	%B	Time (min)	Flow-rate (mL/min)	%A	%B	Time (min)
Caffeic acid	0.4	95	5	0	1	95	5	0	17	95	5	0
		95	5	0.05		40	60	60		40	60	60
		40	60	12.92		0	100	61		0	100	61
		0	100	13.14		0	100	70		0	100	70
		0	100	15.07								
Ferulic acid	0.4	70	30	0	1	70	30	0	17	70	30	0
		70	30	0.05		45	55	30		45	55	30
		45	55	6.49		35	65	60		35	65	60
		35	65	12.92		0	100	61		0	100	61
		0	100	13.14		0	100	70		0	100	70
		0	100	15.07								
Mixture caffeic/ferulic acid	0.4	80	20	0	1	80	20	0	17	80	20	0
		80	20	0.05		40	60	60		40	60	60
		40	60	12.92		0	100	61		0	100	61
		0	100	13.14		0	100	70		0	100	70
		0	100	15.07								
