# Supplementary figures and images for: Chemoenzymatic Synthesis of Complex Phenylpropanoid Derivatives by the Botrytis cinerea Secretome and Evaluation of Their Wnt Inhibition Activity
Source: Front Plant Sci. 2022 Jan 13;12:805610. doi: 10.3389/fpls.2021.805610 (PMC8792767; doi:10.3389/fpls.2021.805610)

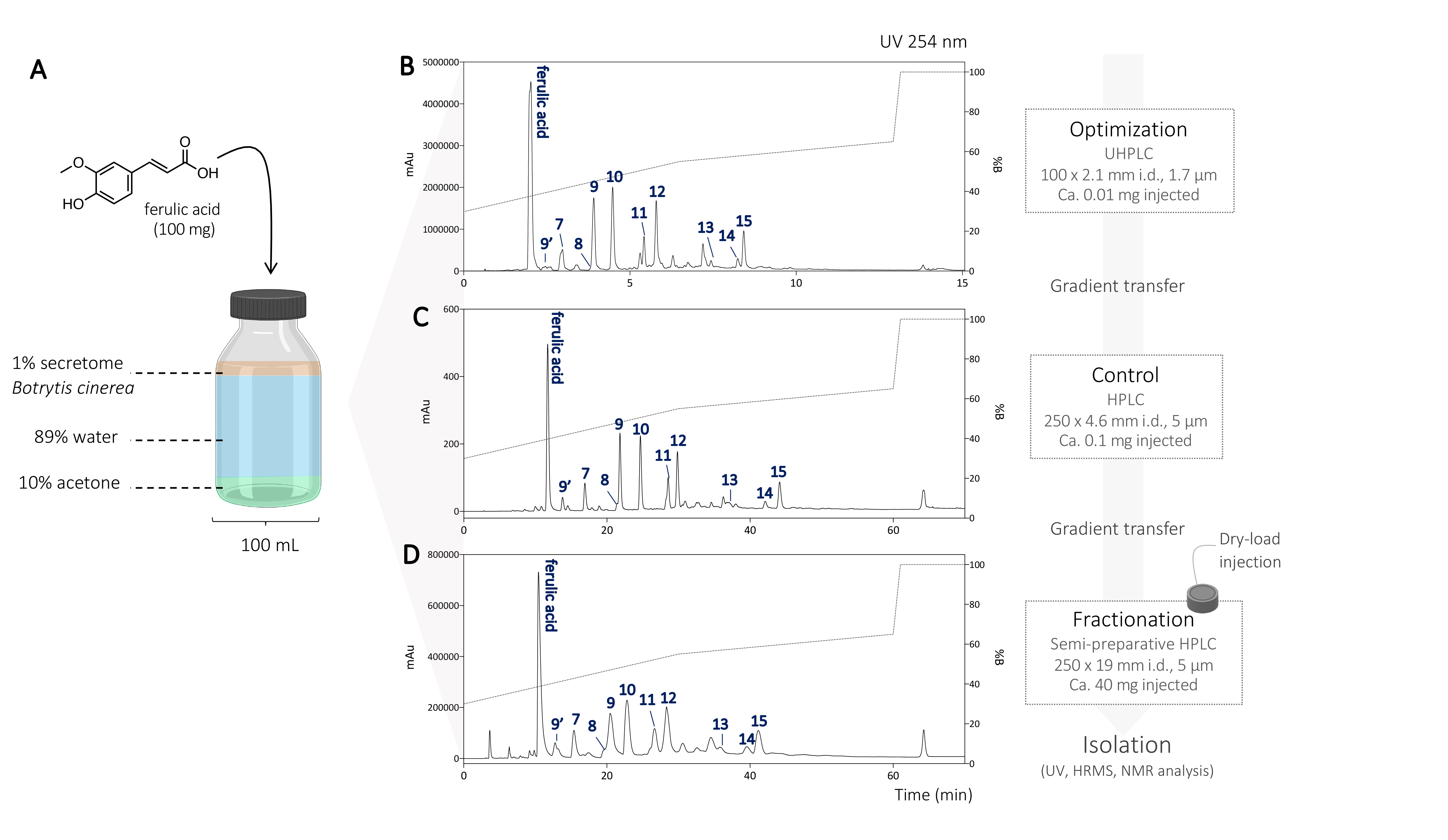

Supplement: Supplementary file 2 [file Image_1.JPEG]

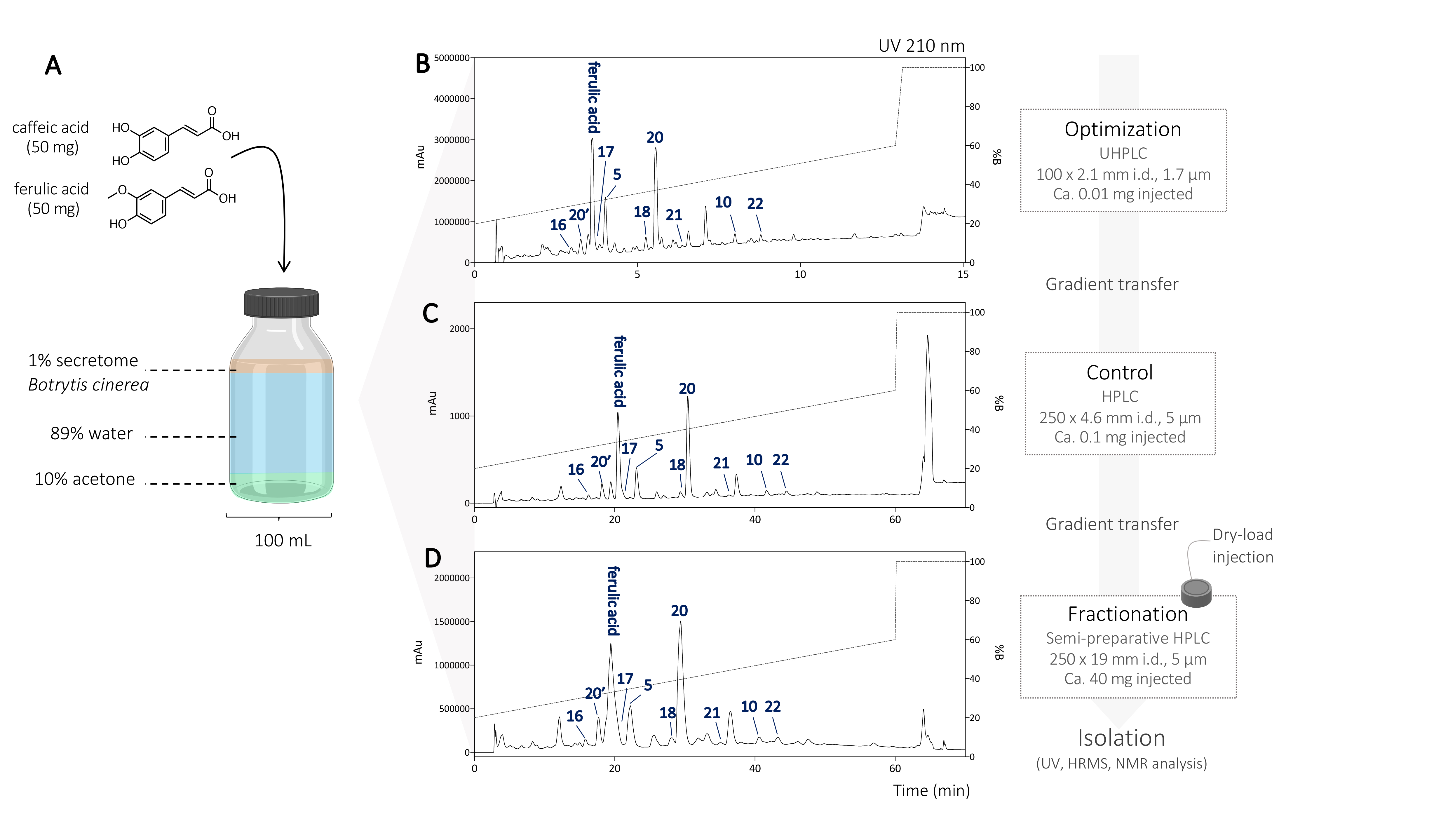

Supplement: Supplementary file 3 [file Image_2.JPEG]

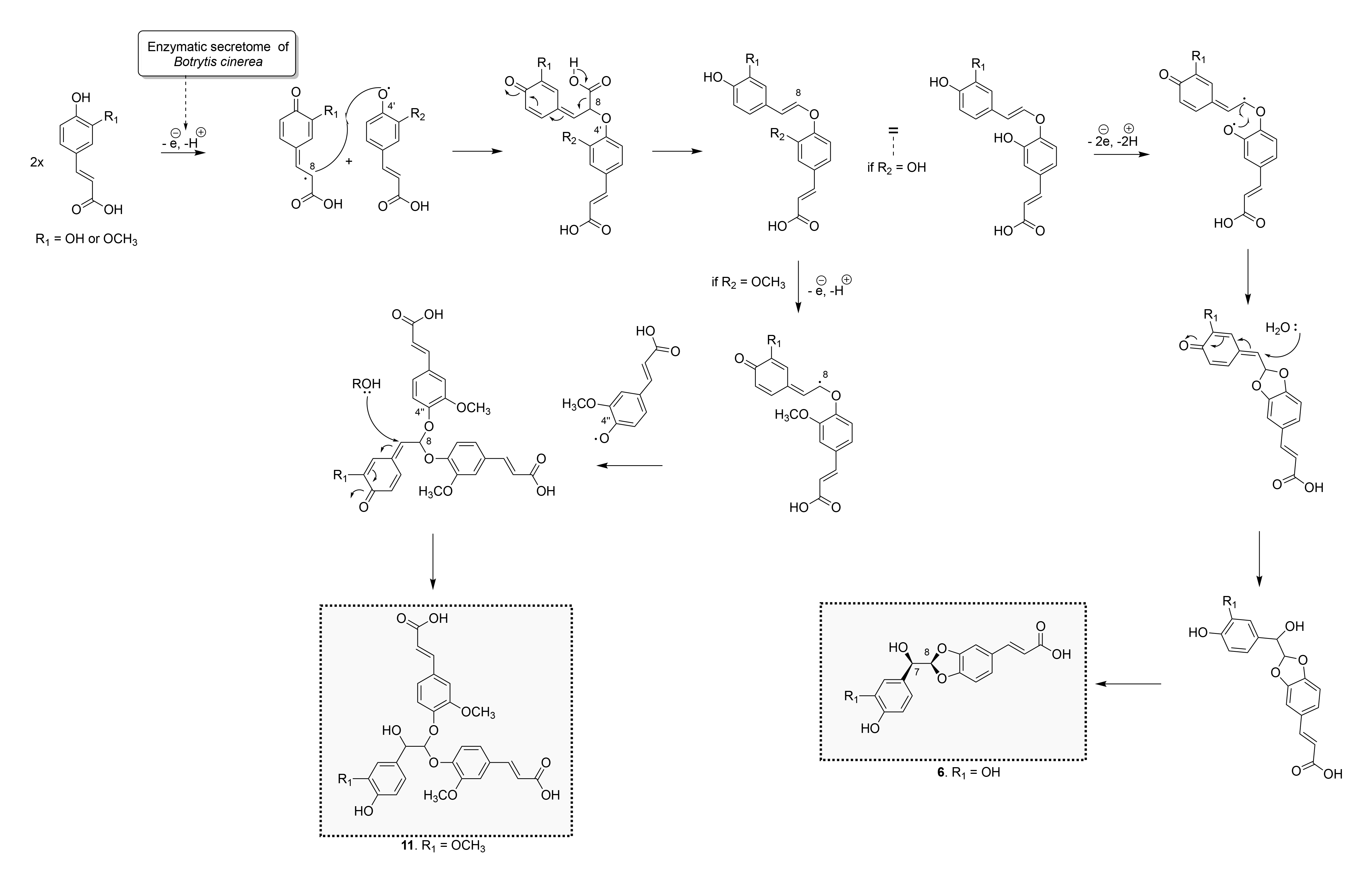

Supplement: Supplementary file 4 [file Image_3.JPEG]

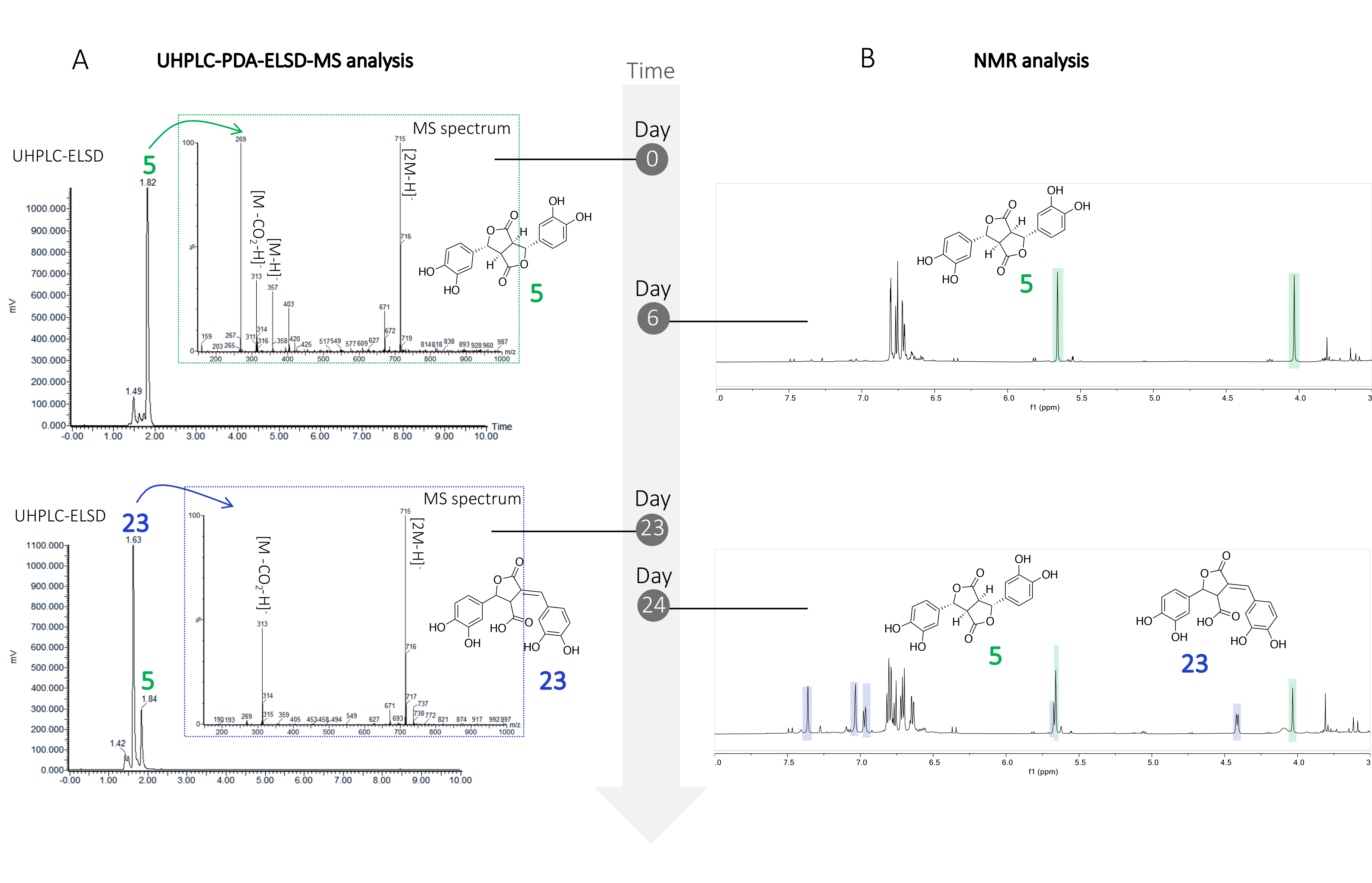

Supplement: Supplementary file 5 [file Image_4.JPEG]

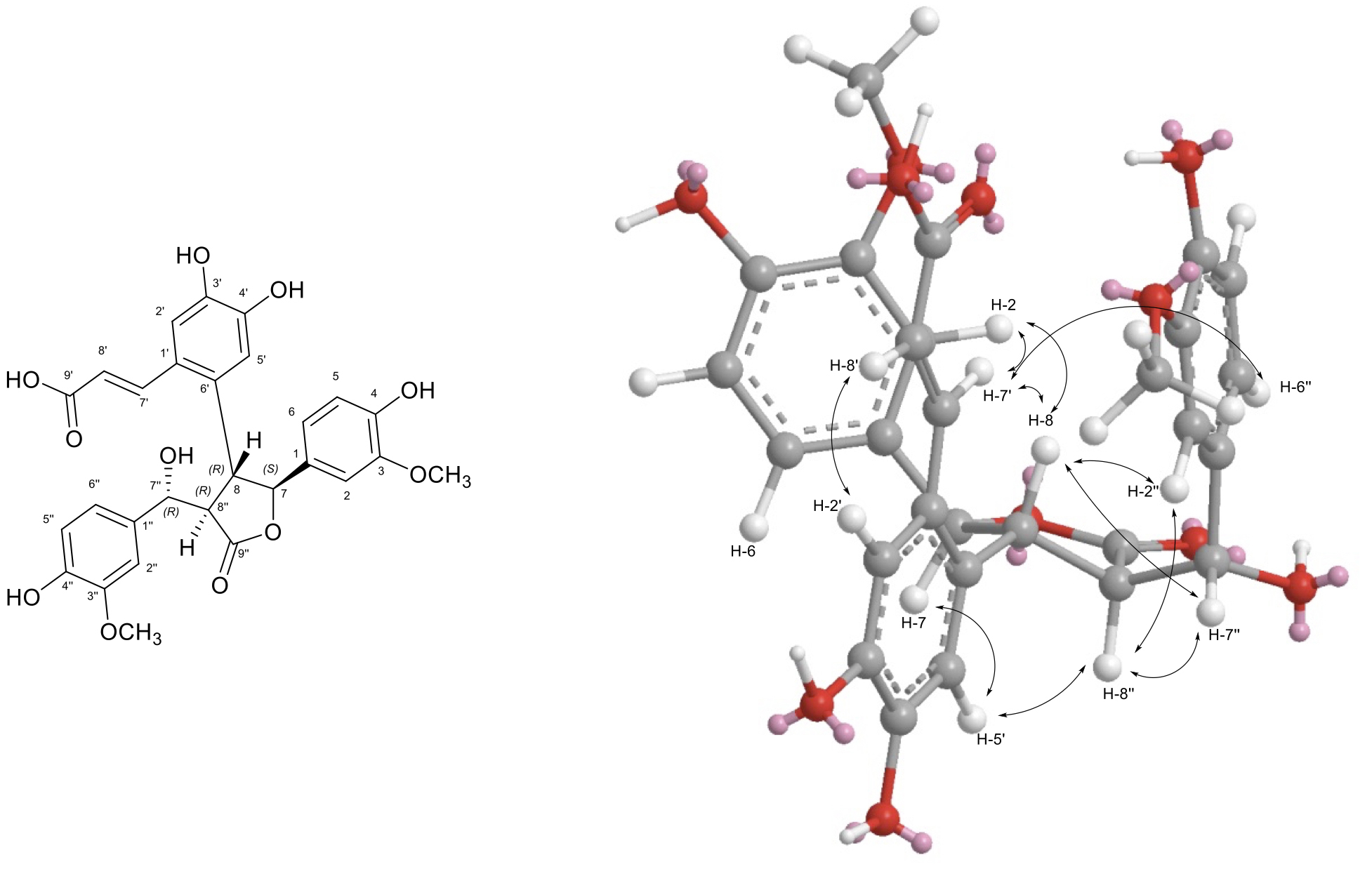

Supplement: Supplementary file 6 [file Image_5.JPEG]

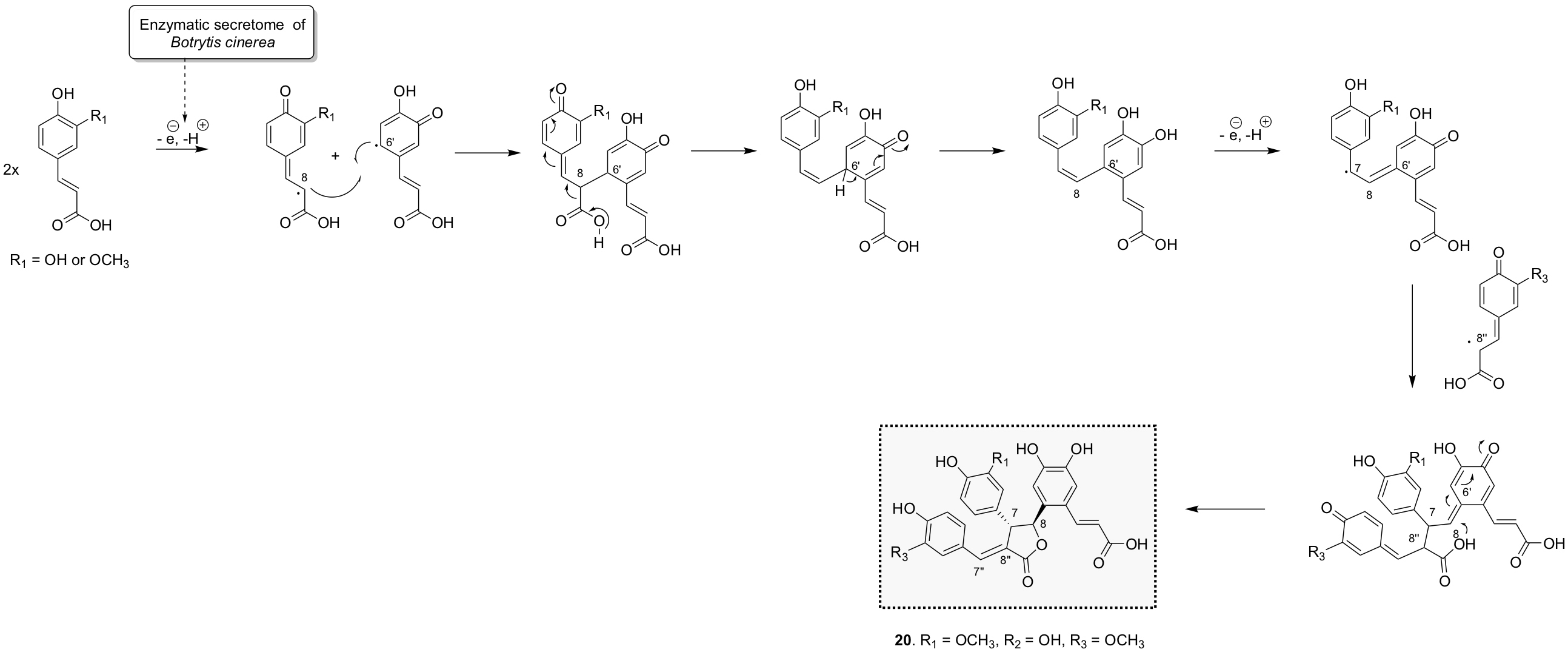

Supplement: Supplementary file 7 [file Image_6.JPEG]

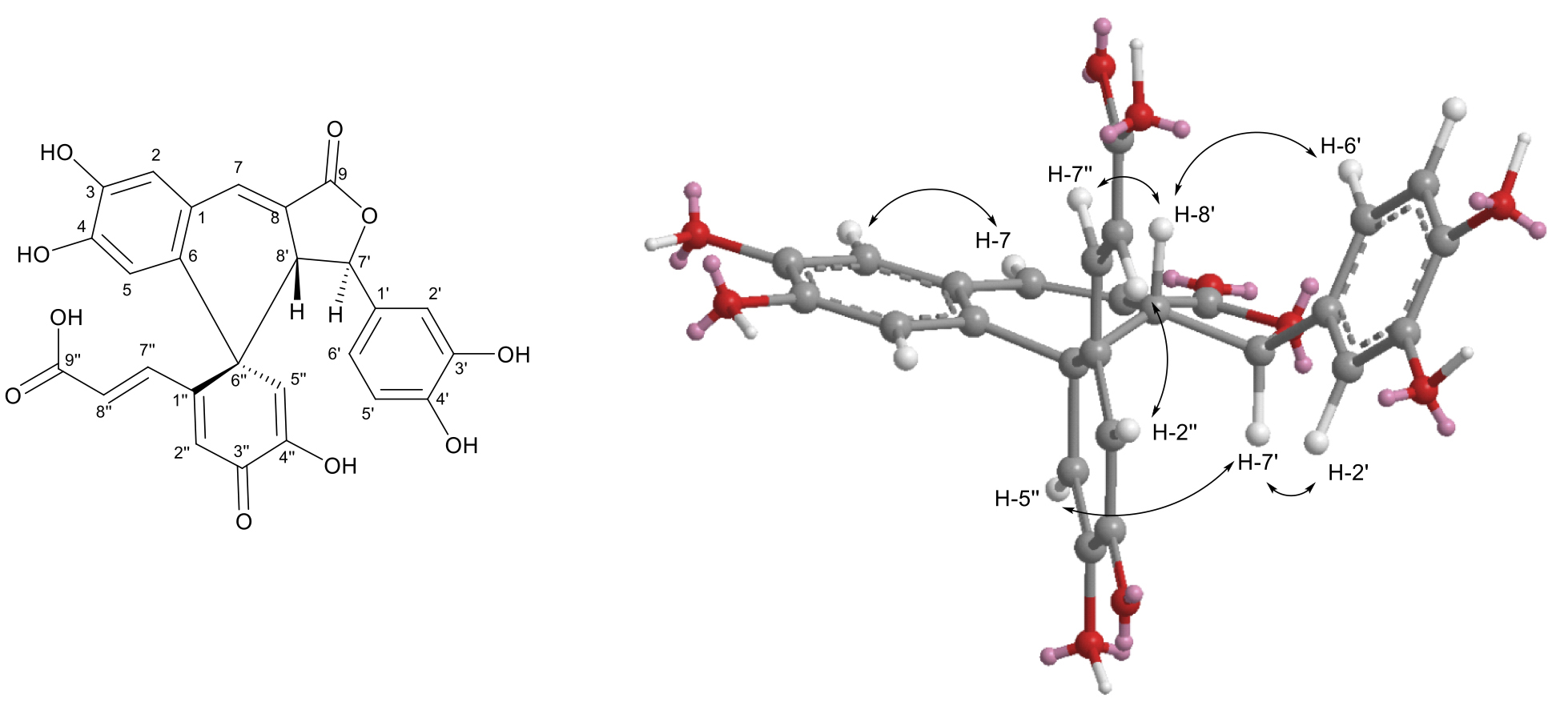

Supplement: Supplementary file 8 [file Image_7.JPEG]
